# Antioxidant Power of Vitexin and Isovitexin Against OOH Radicals: A Comparative Theoretical Investigation

**DOI:** 10.1021/acs.joc.5c01680

**Published:** 2025-10-10

**Authors:** Maciej Spiegel, Nino Russo

**Affiliations:** a Department of Organic Chemistry and Pharmaceutical Technology, Faculty of Pharmacy, 49550 Wroclaw Medical University, Borowska 211 A, Wroclaw 50-556, Poland; b Dipartimento di Chimica e Tecnologie Chimiche, Università della Calabria, Rende (CS) I-87136, Italy

## Abstract

The antioxidant activity of vitexin and isovitexin—flavonoids widely found in various plants and used in traditional medicine—has been theoretically evaluated against the OOH radical. Multiple reaction mechanisms, including hydrogen atom transfer, single electron transfer, and radical adduct formation, were considered. The study accounts for all species present in aqueous solution at physiological pH as well as in lipid-like environments. Both compounds exhibit similar antioxidant activities, with apparent rate constants of 1.45 × 10^3^ M^–^^1^ s^–^^1^ for vitexin and 4.78 × 10^3^ M^–^^1^ s^–^^1^ for isovitexin. Compared to Trolox, a commonly used reference compound, their radical scavenging capacities are slightly lower.

## Graphical abstract

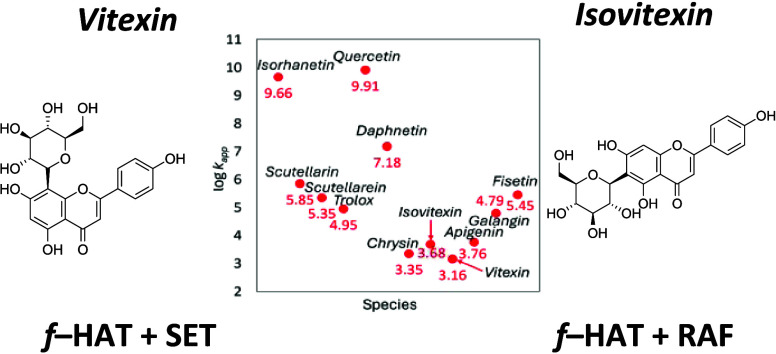

## Introduction

Vitexin (apigenin-8-C-β-d-glucopyranoside) and isovitexin (apigenin-6-C-β-d-glucopyranoside), depicted in Figure 1, are mono-C-glycosylflavones found in various natural sources, including certain insects,^1^ honey,^2^ fungi,^3^ and a wide range of plants such as pigeon pea,^4,5^ mung bean,^6^ mosses,^7^ Passiflora species,^8^ bamboo,^9^ mimosa,^10^ wheat leaves,^11^ as well as in numerous fruits, flowers,^12–15^ roots,^16^ and leaves.^17^ Growing scientific interest in vitexin and isovitexin stems from their multitarget pharmacological effects^17^ and their potential health benefits against several diseases, including diabetes mellitus,^18^ cancer,^19^ oxidative stress-related conditions,^20,21^ Alzheimer’s disease,^22^ inflammation,^23^ ischemic injury,^24,25^ cardiovascular and metabolic disorders,^26^ and neurological or psychiatric diseases.^27^

**Figure 1:**
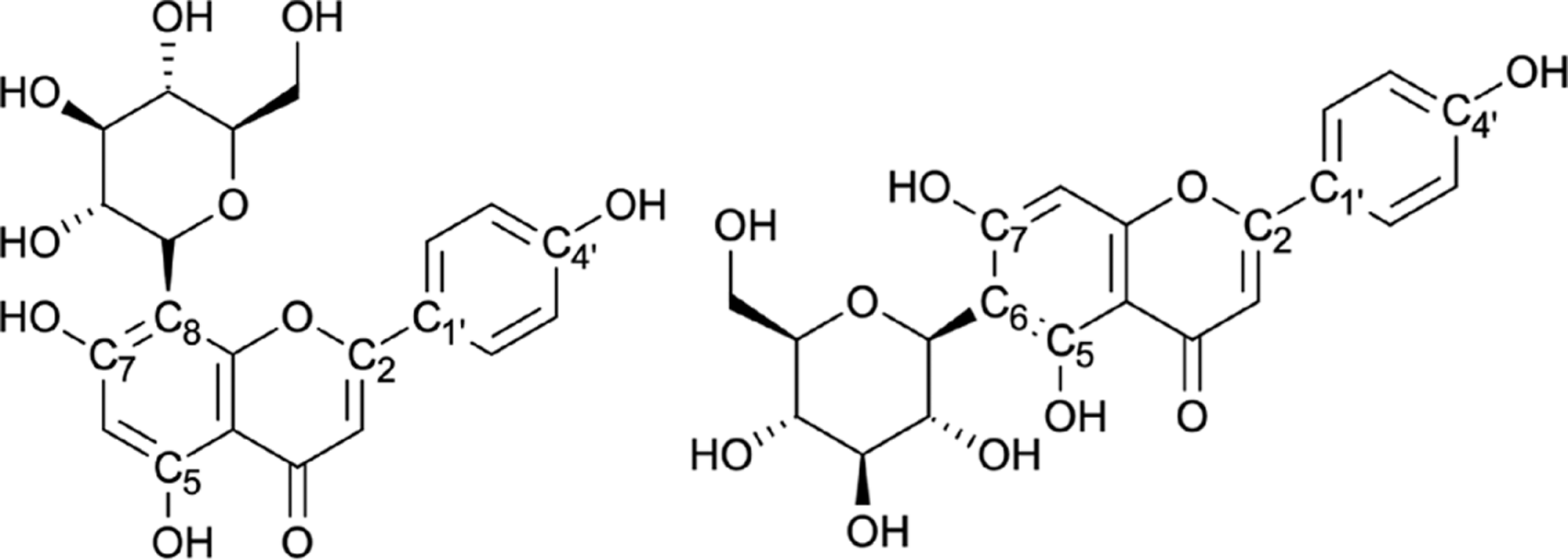
Molecular structures of vitexin (left) and isovitexin (right) with atoms labeled.

Most of these biological activities can be attributed, at least in part, to their antioxidant properties, which help reduce levels of reactive oxygen species (ROS).^28^ Recent experimental studies have shown that both isomers possess significant free radical scavenging activity against DPPH, nitric oxide, and superoxide radicals.^29^ Notably, isovitexin appears to be more effective against hydroxyl radicals than DPPH radicals,^30^ whereas vitexin has demonstrated higher scavenging activity toward DPPH radicals in other studies.^17^ Overall, the antioxidant capacities of vitexin and isovitexin appear to be comparable, with some variation depending on the radical species examined.

The primary mechanisms by which antioxidants neutralize free radicals have been previously described^31,32^ and generally involve the formation of less reactive species through electron or hydrogen transfer. These processes can be categorized into the following redox and nonredox mechanisms:

Hydrogen Atom Transfer (HAT): HA + R^•^ → HA^•^ + RH
Single Electron Transfer (SET): HA + R^•^ → HA^+•^ + R^–^;
Radical Adduct Formation (RAF): HA + R^•^ → [HA-RH]^•^

The above represents the essential initial step, and often the more important, to assess antiradical activity. However, recent studies have proposed more complex mechanisms, including sequential proton loss electron transfer (SPLET), single electron transfer–proton transfer (SET-PT), sequential proton loss hydrogen atom transfer (HAT), double and triple variants of HAT, SPLET, and SET-PT, proton-coupled electron transfer (PCET), radical adduct formation followed by hydrogen atom abstraction (RAF-HAA), hydrogen atom transfer followed by radical–radical coupling (HAT-RRC), sequential proton loss electron transfer–radical–radical coupling (SPLET-RRC), and radical adduct formation followed by radical–radical coupling (RAF-RRC). Nonetheless, to maintain consistency with previous studies—most of which follow the QM-ORSA protocol—this work focuses on the three primary mechanisms mentioned initially.

Numerous studies have highlighted that the antioxidant activity of a compound depends on the reaction environment (e.g., aqueous vs lipid phase) as well as on its molecular and electronic structure.^37–40^ However, detailed insights into the specific antioxidant mechanisms of vitexin and isovitexin are still lacking. To address this gap, we performed a comprehensive theoretical investigation of the reactivity of vitexin and isovitexin toward the OOH radical. All possible species present in aqueous solution at pH = 7.4 and in lipid-like environments were considered. Our calculations employed a density functional theory-based computational protocol, previously validated in related antioxidant studies.^33–37^

## Computational Details

The initial geometries of vitexin and isovitexin, obtained through meta-dynamics sampling as implemented in the CREST software,^38^ have been subject to full optimization using the M05–2X exchange-correlation meta-GGA functional^39^ combined with the 6–311+G­(d,p) basis set^40,41^ and the SMD implicit solvation model.^42^ At each stage of the study, frequency calculations were performed to confirm that the structures correspond to minima (ground states) or first-order saddle points (transition states), and to extract zero-point energy corrections for the evaluation of Gibbs free energies and activation barriers. Open-shell species were treated using the unrestricted formalism. Intrinsic reaction coordinate calculations were carried out for each transition state to confirm proper connectivity between reactants and products.

Solvation effects were modeled using pentyl ethanoate (ε = 4.7) to simulate lipid-like environments and water (ε = 78.4) for aqueous conditions. In aqueous solution, the p*K*_a_ values and molar fractions at physiological pH (7.4) were computed using a parameter fitting method,^43^ accounting for the fact that different protonation states of the same molecule can exhibit markedly distinct antioxidant activity.

Global reactivity indices such as bond dissociation energies (BDE), ionization potentials (IP), proton affinities (PA) and proton desorption energies (PDE) were estimated in the framework of the adiabatic approximation using the recently proposed solvation enthalpies of H^+^ (Δ*H*(H^+^) = 1055.7 kJ/mol) and electron (Δ*H* (e−) = 77.5 kJ/mol.^44^

Rate constants have been calculated in the framework of the quantum mechanics-based test for overall free radical scavenging activity (QM-ORSA) approach,^45^ based on the conventional transition state theory (TST), using the expression:

$$k=\sigma \kappa \frac{k_{B}T}{h}e^{-\Delta G^{\neq }/RT}$$

Where σ and *k* represent the reaction path degeneracy and tunneling correction in Eckart model, *k*_*B*_,h and R are the Boltzmann, Planck, and gas constants respectively, and Δ*G*‡ is the Gibbs free energy of activation at temperature *T*. For single-electron transfer (SET) mechanisms, activation free energies were estimated using Marcus theory:

$$\Delta G^{\neq }=\frac{\lambda }{4}{\left(1+\frac{\Delta G}{\lambda }\right)}^{2}$$

where λ is the reorganization energy, computed using the ΔSCF method. For reactions approaching the diffusion limit, the apparent rate constant was calculated via the Collins–Kimball expression:

$$k_{app}=\left(k_{D}k\right)/\left(k_{D}+k\right)$$

Where *k* is the thermal rate constant from TST, and *k*_*D*_ is the Smoluchowski steady-state diffusion-controlled rate constant. The energies were corrected by means of 1 M standard state and solvent cage effect.

All calculations were carried out using Gaussian 16 (Rev. C.01).^46^ 2D structure visualization was performed using MarvinSketch version 21.15.0 (ChemAxon).

## Result and Discussion

Although differing in the position of glycosylation, both vitexin and isovitexin possess seven hydroxyl groups within their flavone moieties, which can contribute to their antioxidant activity. Since their p*K*_a_ values are not well established in the literature, we first investigated their acid–base behavior in aqueous solution to determine the relevant species present at physiological pH. The deprotonation routes and corresponding molar fraction distributions as a function of pH are shown in Figure 1.

The deprotonation behaviors of vitexin and isovitexin are quite similar; however, some differences are noteworthy. Specifically, the p*K*_a_ values of the first deprotonation steps differ significantly: 6.79 for vitexin and 7.76 for isovitexin. This suggests that glucosylation at the C8 (vitexin) or C6 (isovitexin) position induces non-negligible changes in their acid–base equilibria. These different p*K*_a_ behavior accounts for both steric and electronic differences between the two considered isomers.

Despite the different p*K*_a_ values, the deprotonation sequence is the same for both compounds, proceeding in the order: C7 → C4′ → C5.

Importantly, Figure 2 also reveals the molar distribution of each species in aqueous solution at physiological pH (7.4), a crucial factor for evaluating antioxidant properties. The species distribution at pH 7.4 is as follows:

- Vitexin: H_3_Vtx^–^ (60.40%) > H_4_Vtx (30.15%) > H_2_Vtx^2^^–^ (8.45%)
- Isovitexin: H_4_iVtx (54.19%) > H_3_iVtx^–^ (44.39%) > H_2_iVtx^2^^–^ (1.41%)

**Figure 2:**
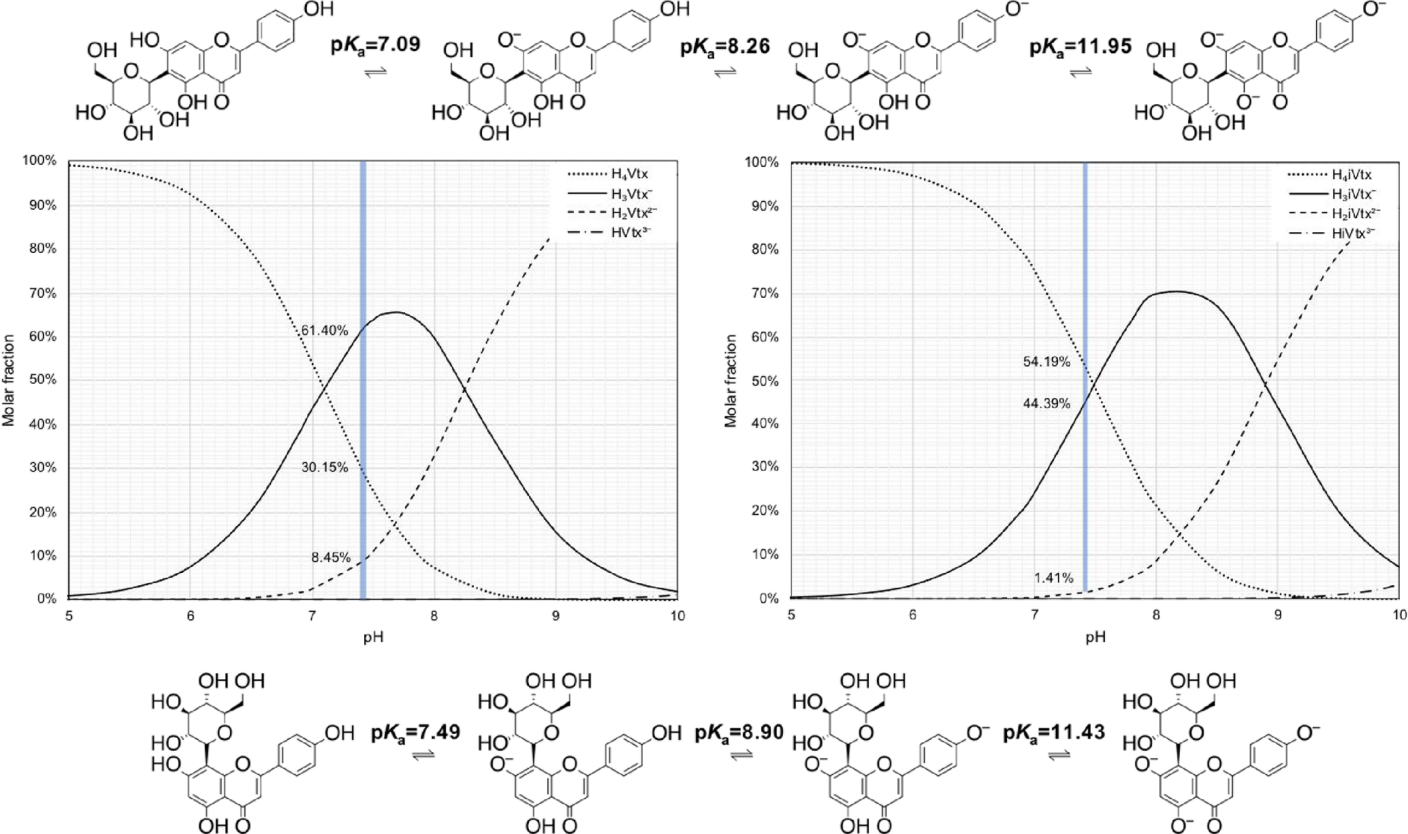
Deprotonation pathways of isovitexin (top) and vitexin (bottom), and their respective molar fraction distributions (left and right) as functions of pH.

At this pH, the neutral species dominates the isovitexin population, while the monodeprotonated species is most prevalent for vitexin. In both cases, the doubly deprotonated species is present only in minor amounts but should still be considered in computational studies. The fully deprotonated species are negligible under physiological conditions.

The antioxidant activity of a molecule can be correlated with key global reactivity descriptors, such as O–H bond dissociation enthalpies and adiabatic ionization potentials. These parameters were computed for both compounds in lipid-like (modeled using pentyl ethanoate, PET) and aqueous environments. The results are reported in Table 1.

**Table 1:** O–H Bond Dissociation Energies (BDEs, in kcal/mol) and Adiabatic Ionization Potentials (aIPs, in eV) of the H_4_Vtx and H_4_iVtx Species in Lipid-Like (PET) and Aqueous (Water) Environments

		H_4_Vtx (PET)	H_4_Vtx (w)	H_3_Vtx^–^ (w)	H_2_Vtx^2–^ (w)
BDE	C_5_	108.9	105.0	103.0	102.6
	C_7_	100.0	102.9		
	C_4**’**_	95.5	97.8	96.6	
aIP		140.7			

		**iH**_**4**_ **Vtx (PET)**	**iH**_**4**_ **Vtx (w)**	**iH**_**3**_ **Vtx** ^**–**^ **(w)**	**iH**_**2**_ **Vtx** ^**2–**^ **(w)**
BDE	C_5_	108.4	103.0	100.4	99.7
	C_7_	104.8	104.4		
	C_4**’**_	95.7	96.9	95.3	
aIP		141.4			

In both solvents and for both molecules, the most labile O–H bond corresponds to the hydroxyl group at the C4′ position, followed by those at C7 and C5. The BDE values of corresponding hydroxyl groups differ by less than 1 kcal/mol between vitexin and isovitexin, indicating similar radical scavenging potential. A similar trend is observed for the computed aIP values, further confirming the comparable antioxidant reactivity of the two isomers.

More accurate insights into the antioxidant potential of vitexin and isovitexin can be obtained by evaluating the thermodynamics of their possible O–H dissociation pathways. The computed Gibbs free energies (ΔG) for each mechanism are reported in Table 2.

**Table 2:** Gibbs Free Energies (Δ*G*, in kcal/mol) for HAT, RAF, and SET Mechanisms between Vitexin (Isovitexin) and the OOH Radical

		H_4_Vtx^PET^ (H_4_iVtx^PET^)	H_4_Vtx (H_4_iVtx)	H_3_Vtx^–^ (H_3_iVtx^–^)	H_2_Vtx^2–^ (H_2_iVtx2^–^)
*f*-HAT	C_5_	16.8 (15.1)	9.0 (6.1)	6.9 (3.9)	6.9 (2.9)
	C_7_	7.0 (11.2)	5.7 (4.7)		
	C_4’_	3.2 (3.0)	1.2 (−0.4)	0.9 (−0.7)	
RAF	C_2_	13.7 (12.1)	12.1 (10.7)	8.1 (7.2)	11.4 (7.8)
	C_3_	9.8 (9.6)	8.9 (7.8)	6.6 (5.7)	8.5 (5.8)
	C_4_	37.9 ()	35.0 ()	14.0 ()	17.6 ()
	C_4’_	28.3 (28.1)	26.8 (25.3)	22.3 (−)	- (−)
	C_5_	16.3 (16.6)	14.8 (13.9)	15.4 (14.9)	17.1 (13.0)
	C_6_	16.1 (23.7)	14.7 (21.8)	11.5 (14.6)	12.5 (13.6)
	C_7_	20.3 (19.7)	18.0 (16.7)	25.3 (21.5)	24.6 (19.2)
	C_8_	17.8 (15.5)	15.9 (13.6)	12.8 (9.8)	13.3 (8.6)
	C_8a_	17.8 (18.6)	17.7 (15.7)	17.7 (12.7)	18.2 (13.2)
	C_1’_	19.3 (21.1)	18.1 (17.9)	18.4 (17.9)	16.8 (14.1)
	C_2’_	15.9 (16.1)	13.4 (12.3)	13.1 (12.0)	16.7 (13.9)
	C_3′_	16.7 (16.3)	16.2 (14.2)	16.2 (14.5)	9.8 (6.6)
	C_4’_	12.5 (11.8)	11.1 (9.3)	10.9 (9.0)	13.9 (11.1)
	C_5′_	16.9 (16.7)	13.9 (14.0)	14.7 (13.7)	9.5 (6.8)
	C_6’_	16.3 (15.4)	13.7 (11.9)	13.3 (12.6)	16.7 (12.6)
SET			38.4 (37.5)	18.5 (18.8)	10.0 (8.5)

In the lipid-like environment, where both compounds exist predominantly in their neutral forms, the HAT mechanism is thermodynamically favored. The most reactive site is the hydroxyl group at the C4′ position, followed by the one at C7. Similarly, in aqueous solution, the HAT pathway remains the most energetically favorable for both compounds, with the same site preference (C4′ > C7). These results confirm that the OOH radical preferentially abstracts a hydrogen atom from the C4′ hydroxyl in both solvents. Although the ΔG values associated with the RAF mechanisms are slightly positive, they are small enough to suggest possible relevance under physiological conditions. In both vitexin and isovitexin, the C3 position is identified as the most energetically favorable site for radical addition. By contrast, the SET mechanism exhibits the highest free energy requirements for both molecules, suggesting that this pathway is the least likely under biological conditions.

For the lowest-energy pathways, we also characterized the corresponding transition states (TSs) and their activation energies (Table 3). In the lipid-like environment, the lowest activation barrier (ΔG‡) is associated with the HAT reaction involving OOH attack at the C4′ site for both vitexin and isovitexin, with very similar values. In aqueous solution, the lowest-energy HAT transition state corresponds to hydrogen abstraction at the C7 position.

**Table 3:** Activation Free Energies (Δ*G*‡, in kcal/mol) for the Lowest-Energy HAT, RAF, and SET Mechanisms Involving the OOH Radical

	atom	H_4_Vtx^PET^ (H_4_iVtx^PET^)	H_4_Vtx (H_4_iVtx)	H_3_Vtx^–^ (H_3_iVtx^–^)	H_2_Vtx^2–^ (H_2_iVtx2^–^)
HAT	C_4’_	17.9 (17.6)	20.6 (18.4)	20.8 (18.0)	
	C_5_		24.7 (22.9)		
	C_7_	19.9 (−)	17.9 (19.7)		
RAF	C_2_			20.5 (18.2)	- (15.9)
	C_3_	19.5 (19.4)	19.4 (17.5)	17.5 (16.2)	15.6 (12.4)
	C_3′_				15.5 (12.3)
	C_4_		- (20.7)	- (22.7)	
	C_4’_			- (21.2)	
	C_5′_				14.6 (12.7)
	C_8_			- (12.7)	- (10.7)
SET			40.1 (38.7)	19.0 (19.1)	11.7 (11.0)

For the RAF mechanism, the lowest-energy TSs in water are associated with radical addition at the C3 site in both molecules. As expected, the SET mechanism presents the highest energy barriers, with ΔG‡ values of 40.1 and 38.7 kcal/mol for neutral vitexin and isovitexin, respectively.

The optimized geometries of the intercepted transition states are illustrated in Figures S1 and S2 (Supporting Information), and the corresponding imaginary frequencies confirm that the hydrogen transfer process is in progress in these structures.

The computed rate constants and branching ratios for the most favorable reaction pathways are reported in Tables 4 and 5 for vitexin and isovitexin, respectively. These data provide a quantitative comparison of the reactivity of each species toward the OOH radical under biologically relevant conditions.

**Table 4:** Rate constants (k, M^–^^1^ s^–^^1^) and Branching Ratios (Γ) for the Most Favorable Reactions of Vitexin with the OOH Radical

	lipid		water					
	**H**_**4**_ **Vtx** ^PET^		**H**_**4**_ **Vtx** (30.15%)		**H**_**4**_ **Vtx** ^**–**^ (61.40%)		**H**_**4**_ **Vtx** ^**2–**^ (8.45%)	
mechanism	*k* (M^–1^ s^–1^)	Γ (%)	*k* (M^–1^ s^–1^)	Γ (%)	*k* (M^–1^ s^–1^)	Γ (%)	*k* (M^–1^ s^–1^)	Γ (%)
HAT-C_5_			1.30 × 10^–2^	0.04				
HAT-C_7_	1.34 × 10^0^	0.96	2.02 × 10^1^	58.94				
HAT-C_4’_	1.39 × 10^2^	99.01	1.40 × 10^1^	40.86	1.34 × 10^1^	90.83		
RAF-C_2_					9.16 × 10^–3^	0.06		
RAF-C_3_	4.74 × 10^–2^	0.03	5.55 × 10^–2^	0.16	1.27 × 10^0^	8.59	2.64 × 10^1^	0.16
RAF-C_3′_							3.10 × 10^1^	0.18
RAF-C_5′_							1.30 × 10^2^	0.76
SET			2.46 × 10^–17^	0.00	7.56 × 10^–2^	0.51	1.69 × 10^4^	98.90
total	1.40 × 10^2^		3.43 × 10^1^		1.48 × 10^1^		1.70 × 10^4^	
overall			1.03 × 10^1^		9.08 × 10^0^		1.43 × 10^3^	
apparent			1.45 × 10^3^					

**Table 5:** Rate Constants (k, M^–^^1^ s^–^^1^) and Branching Ratios (Γ) for the Most Favorable Reactions of Isovitexin with the OOH Radical

	lipid		water					
	H_4_iVtx^PET^		**H**_**4**_ **iVtx** (54.19%)		**H**_**4**_ **iVtx** ^–^ (44.39%)		**H**_**4**_ **iVtx** ^**2–**^ (1.41%)	
mechanism	*k* (M^–1^ s^–1^)	Γ (%)	*k* (M^–1^ s^–1^)	Γ (%)	*k* (M^–1^ s^–1^)	Γ (%)	*k* (M^–1^ s^–1^)	Γ (%)
HAT-C_5_			2.08 × 10^–1^	0.04%				
HAT-C_7_			1.23 × 10^1^	2.55%				
HAT-C_4’_	1.74 × 10^2^	100.00	4.67 × 10^2^	97.15%	7.85 × 10^2^	17.1%		
RAF-C_2_					4.74 × 10^1^	0.01%	2.04 × 10^1^	0.01%
RAF-C_3_	6.15 × 10^–2^	0.00	1.22 × 10^0^	0.25%	1.17 × 10^1^	0.25%	5.51 × 10^3^	3.15%
RAF-C_4_					1.81 × 10^–4^	0.00%		
RAF-C_8_					3.79 × 10^3^	82.64%	1.01 × 10^5^	57.67%
RAF-C_3′_							6.95 × 10^3^	3.97%
RAF-C_4’_			6.71 × 10^–3^	0.00%	3.05 × 10^–3^	0.00%		
RAF-C_5′_							3.69 × 10^3^	2.11%
SET			2.52 × 10^–16^	0.00%	6.50 × 10^–2^	0.00%	5.80 × 10^4^	33.09%
total	1.74 × 10^2^		4.81 × 10^2^		4.59 × 10^3^		1.75 × 10^5^	
overall			2.61 × 10^2^		2.04 × 10^3^		2.48 × 10^3^	
apparent			4.78 × 10^3^					

From Table 4, it is evident that in a lipid-like environment, vitexin exerts its antioxidant activity predominantly through a HAT mechanism, with the hydroxyl group at the C4′ position serving as the principal reactive site. In aqueous solution, the neutral form of vitexin (H_4_Vtx; molar fraction: 30.15%) contributes primarily via HAT-type reactions at both the C7 and C4′ positions, with comparable contributions of 58.94% and 40.86%, respectively. For the monodeprotonated form (H_3_Vtx^–^; molar fraction: 60.40%), the observed antioxidant activity results from both the HAT reaction at C4′ and the RAF mechanism at C3, with the HAT pathway being dominant (branching ratios: 90.83% HAT, 8.59% RAF). Although the dianionic species (H_2_Vtx^2^^–^) is present in lower concentration (8.45%), it significantly contributes to the overall kinetics, predominantly via the SET mechanism (98.90%).

The apparent overall rate constant (*k*_app_) for vitexin is computed to be 1.45 × 10^3^ M^–^^1^ s^–^^1^, a value comparable to that of the H_2_Vtx^2^^–^ species alone, highlighting the non-negligible role of even minor solution species. The computed kinetic constants for isovitexin are presented in Table 5. In the lipid-like medium, its antioxidant action is exclusively attributed to the HAT mechanism at the C4′ position, yielding a rate constant of 1.74 × 10^2^ M^–^^1^ s^–^^1^, slightly higher than that of vitexin under the same conditions (1.40 × 10^2^ M^–^^1^ s^–^^1^). In aqueous solution, the neutral species H_4_iVtx (molar fraction: 54.19%) shows a kinetic constant of 4.67 × 10^2^ M^–^^1^ s^–^^1^, nearly entirely arising from the HAT mechanism at C4′ (97.15%). The monodeprotonated form H_3_iVtx^–^ (molar fraction: 44.39%) exhibits its highest reactivity via RAF at the C8 position (3.79 × 10^3^ M^–^^1^ s^–^^1^, 82.64% contribution), with additional contributions from HAT at C4′ (7.85 × 10^2^ M^–^^1^ s^–^^1^) and minor SET/RAF processes involving the dianionic H_2_iVtx^2^^–^ species.

The resulting overall rate constant for isovitexin is predicted to be 4.78 × 10^3^ M^–^^1^ s^–^^1^, notably higher than that of vitexin. This suggests that isovitexin may possess slightly superior antioxidant activity under physiological conditions. However, the difference is modest, and our results support the conclusion that both compounds exhibit comparable antioxidant capacities against the OOH radical. This theoretical prediction aligns with available experimental data, which show similar antioxidant behavior for vitexin and isovitexin against various radical species such as DPPH, ABTS, and CO_3_•^–^.^48^

A broader comparison of antioxidant efficiency against the OOH radical can be drawn from Figure 3, which plots the logarithmic values of the apparent rate constants (log *k*_app_) for vitexin, isovitexin, and other well-known antioxidant molecules. These results provide a useful benchmark for contextualizing the reactivity of these naturally occurring C-glycosylflavones within the antioxidant landscape.

**Figure 3:**
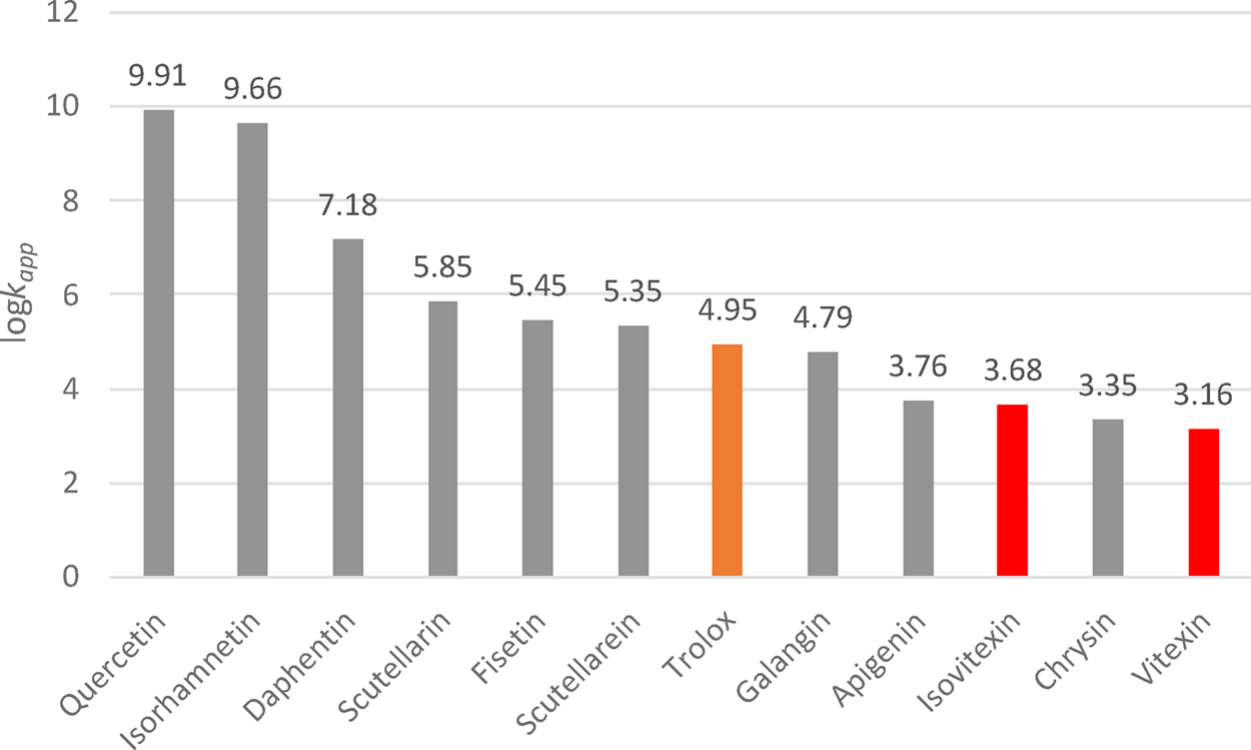
Comparison of computed apparent kinetic constant values (*k*_app_) in aqueous solution for vitexin, isovitexin, and selected reference antioxidants against the OOH radical.

As evident from Figure 3, the antioxidant capacities of vitexin and isovitexin are markedly lower than those of other well-established antioxidants. Specifically, their apparent rate constants are significantly smaller than those of isorhamnetin (*k*_app_ = 4.60 × 10^9^ M^–^^1^ s^–^^1^),^47^ quercetin (8.11 × 10^9^ M^–^^1^ s^–^^1^)^48^ daphnetin (1.51 × 10^7^ M^–^^1^ s^–^^1^),^49^ fisetin (2.81 × 10^5^ M^–^^1^ s^–^^1^)^50^ and galangin (6.21 × 10^4^ M^–^^1^ s^–^^1^).^51^ Moreover, their activities are approximately 2 orders of magnitude lower than those of scutellarin (7.09 × 10^5^ M^–^^1^ s^–^^1^)^52^ and scutellarein (2.23 × 10^5^ M^–^^1^ s^–^^1^).^53^ Vitexin and isovitexin exhibit antioxidant potencies comparable to chrysin (2.24 × 10^3^ M^–^^1^ s^–^^1^)^54^ and apigenin (5.78 × 10^3^ M^–^^1^ s^–^^1^).^54^ When compared to Trolox (8.96 × 10^4^ M^–^^1^ s^–^^1^),^55^ a widely used reference antioxidant, both vitexin and isovitexin display substantially lower reactivity toward the OOH radical, underscoring their relatively modest radical-scavenging efficiency in aqueous environments.

## Conclusions

In this study, we investigated the primary antioxidant properties of vitexin and its isomer isovitexin against the OOH radical, employing the ORSA quantum mechanical protocol. The key findings are summarized below:

- In aqueous solution, the deprotonation behavior of the two isomers is similar, although isovitexin exhibits slightly higher p*K*_a_ values for the first two dissociation steps.
- At physiological pH (7.4), species distribution differs: the neutral form predominates for isovitexin, while the monodeprotonated form is more abundant for vitexin.
- In a lipid-like environment, HAT mechanism is the preferred antioxidant pathway for both compounds.
- In aqueous solution, vitexin primarily follows HAT and single-electron transfer SET mechanisms, whereas isovitexin favors HAT and RAF mechanisms.
- The apparent kinetic constants are of the same order of magnitude, with isovitexin displaying slightly higher reactivity.

Overall, both vitexin and isovitexin demonstrate antioxidant activity toward the OOH radical, although with lower potency compared to Trolox, a widely used reference antioxidant.

## Supplementary Material



## Data Availability

The data underlying this study are available in the published article and its Supporting Information.

## Notes

Additional Figures—transition states, and *XYZ*-coordinates with energies (PDF)
