# Supplementary material for: Antioxidant Power of Vitexin and Isovitexin Against OOH Radicals: A Comparative Theoretical Investigation
Source: J Org Chem. 2025 Oct 10;90(42):14957–64. doi: 10.1021/acs.joc.5c01680 (PMC12570259; doi:10.1021/acs.joc.5c01680)
Supplement: Supplementary file 1 [file jo5c01680_si_001.pdf]

Supplementary information

Antioxidant Power of Vitexin and Isovitexin Against OOH Radicals: A Comparative Theoretical Investigation

Maciej Spiegel [a], Nino Russo *[b]

[a] M. Spiegel

Department of Organic Chemistry and Pharmaceutical Technology, Wroclaw

Medical University, Borowska 211 A, 50-556 Wroclaw, Poland

[b] N. Russo

Dipartimento di Chimica e Tecnologie Chimiche, Università della Calabria, I-

87136 Rende (CS), Italy

E-mail: nino.russo@unical.it

Table of Contents

Figure S1. Transition state structures and imaginary frequences for the HAT reactions between OOH and vitexin

Figure S2. Transition state structures and imaginary frequences for the HAT reactions between OOH and isovitexin.

Cartesian Coordinates

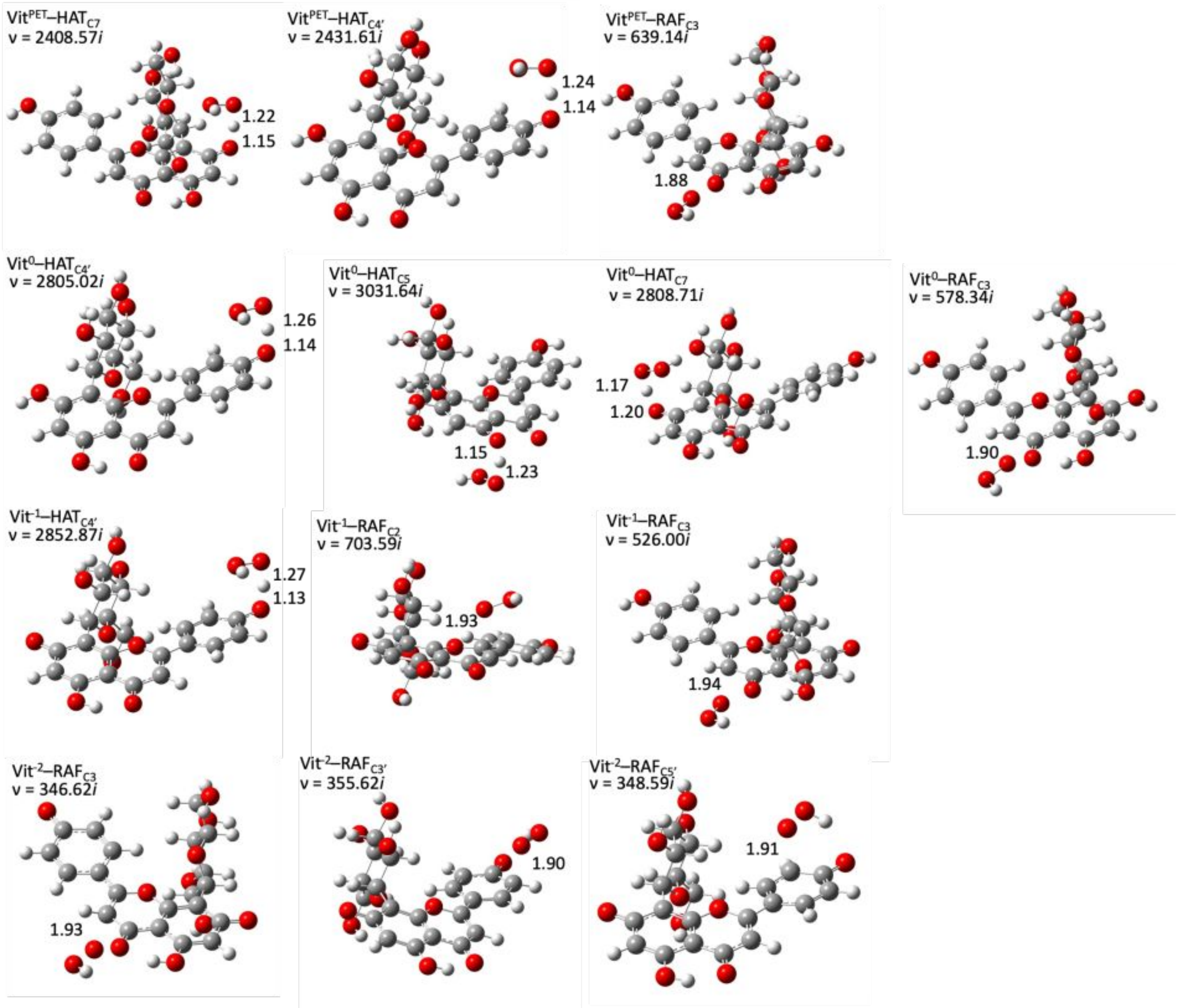

Figure S1. Transition state structures and imaginary frequences for the HAT reactions between OOH and vitexin

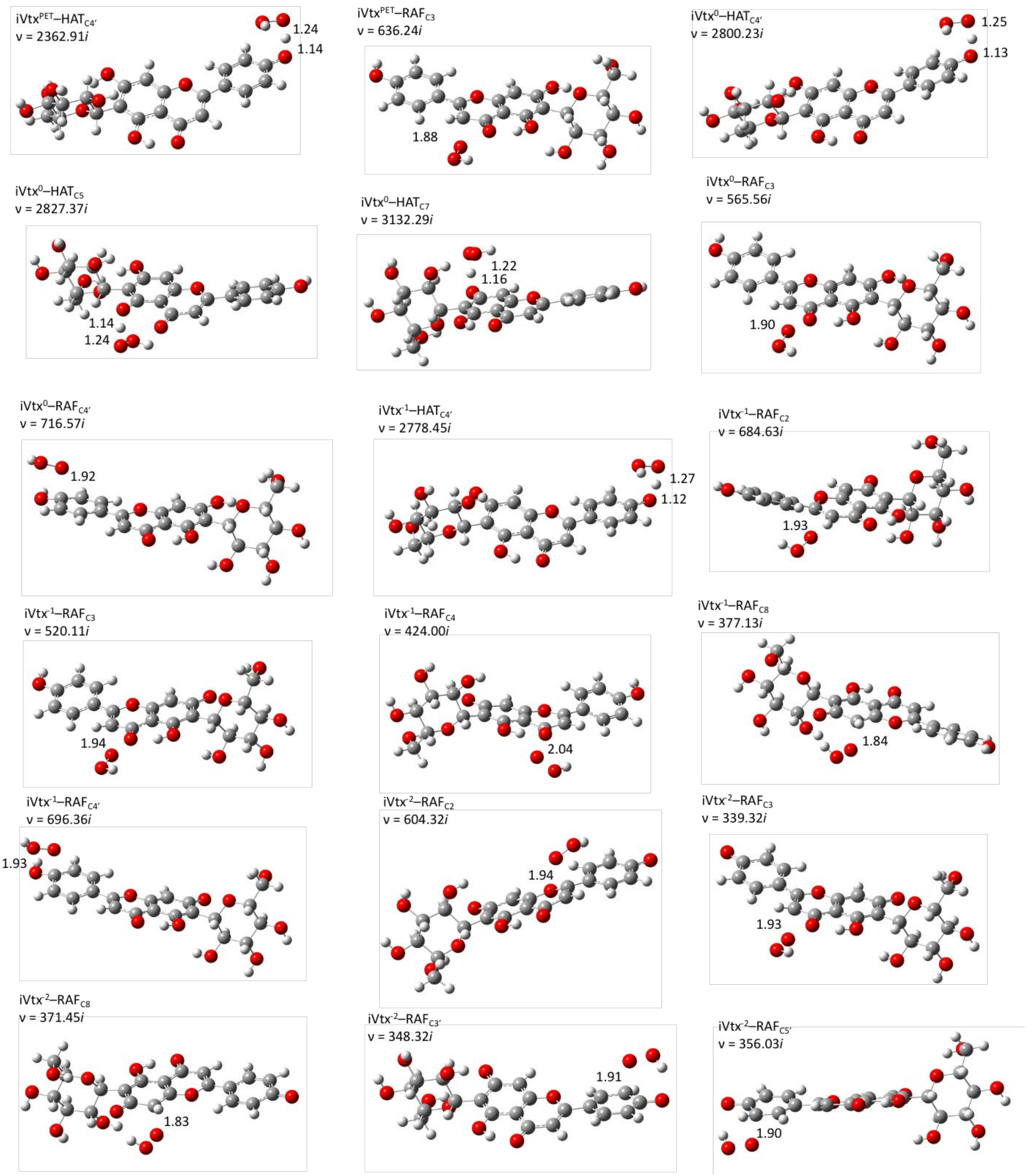

Figure S2. Transition state structures and imaginary frequences for the HAT reactions between OOH and isovitexin.

XYZ-Coordinates

==> ISOVITEXIN/LIPID <==
51
XYZ-COORDINATES + ENERGIES

O	3.470026	0.747896	0.824022
O	2.891569	-1.935350	-1.616859
O	5.489748	-1.052510	-2.278221
O	6.910992	0.160175	-0.177186
O	5.072485	3.032251	0.866321
O	0.897926	-2.312241	1.133667
O	1.660650	2.049969	-0.600832
O	-2.725028	0.507420	-0.152296
O	-1.536962	-3.058938	1.379662
O	-8.917865	1.294602	-0.609566
C	3.397464	-0.719051	-1.095420
C	4.898932	-0.881736	-0.998869
C	2.828537	-0.426184	0.300620
C	5.521349	0.351350	-0.378130
C	4.887600	0.609042	0.981500
C	1.347214	-0.170749	0.249498
C	5.385832	1.879539	1.638023
C	0.446509	-1.155419	0.640708
C	0.835145	1.048493	-0.239723
C	-0.947571	-0.950696	0.512200
C	-0.528674	1.275844	-0.376622
C	-1.393937	0.266427	-0.001975
C	-1.902695	-1.965843	0.910067
C	-3.642071	-0.418327	0.212737
C	-3.287276	-1.614542	0.735824
C	-5.015805	0.040606	-0.014284
C	-6.068420	-0.875910	-0.113906
C	-5.295123	1.404032	-0.130232
C	-7.365590	-0.444279	-0.312564
C	-6.593495	1.844645	-0.324968
C	-7.630610	0.920171	-0.415306
H	3.169501	0.113233	-1.769707
H	5.118329	-1.749004	-0.364799
H	3.035693	-1.276234	0.952842
H	5.340475	1.214362	-1.026599
H	5.094845	-0.239510	1.642505
H	6.469064	1.828290	1.719720
H	4.958967	1.956361	2.639641
H	1.949657	-1.838209	-1.788846
H	5.079783	-1.818876	-2.691692
H	7.278209	-0.147128	-1.012292
H	-0.895004	2.219495	-0.751908
H	4.163316	3.278758	1.050611
H	0.094999	-2.860816	1.343208
H	2.543171	1.866710	-0.232693
H	-4.041301	-2.317673	1.052449
H	-5.873152	-1.937523	-0.056871
H	-4.497021	2.128212	-0.056881
H	-8.180679	-1.149057	-0.399628
H	-6.803230	2.904131	-0.404381
H	-8.977833	2.253798	-0.669458

ENERGIES
SCF ENERGY: -1564.86288516
SUM OF ELECTRONIC AND ZERO-POINT ENERGIES: -1564.462991
SUM OF ELECTRONIC AND THERMAL ENERGIES: -1564.435898
SUM OF ELECTRONIC AND THERMAL ENTHALPIES: -1564.434954
SUM OF ELECTRONIC AND THERMAL FREE ENERGIES: -1564.520249

==> ISOVITEXIN/LIPID/AIP <==
51
XYZ-COORDINATES + ENERGIES

O	3.462060	0.719656	0.847318
O	2.891721	-1.889672	-1.671416
O	5.500194	-0.992712	-2.289783
O	6.909571	0.146944	-0.134015
O	5.052642	3.005928	0.937031
O	0.898879	-2.360734	1.008188
O	1.650945	2.078781	-0.470134
O	-2.734114	0.544502	-0.116619
O	-1.580175	-3.117360	1.218264
O	-8.864457	1.291021	-0.551649
C	3.402351	-0.692321	-1.112653
C	4.903657	-0.861876	-1.009831
C	2.830056	-0.440508	0.290470
C	5.525335	0.351800	-0.349077
C	4.881351	0.582664	1.010576
C	1.347684	-0.183086	0.246381
C	5.371141	1.843107	1.692243
C	0.444821	-1.184298	0.581953
C	0.838151	1.060657	-0.172375
C	-0.954081	-0.968513	0.467362
C	-0.534363	1.299576	-0.302113
C	-1.396148	0.282374	0.016631
C	-1.898859	-1.995879	0.808294
C	-3.658752	-0.383041	0.182349
C	-3.300680	-1.632457	0.636372
C	-5.008160	0.048932	-0.009547
C	-6.108884	-0.822254	0.246739
C	-5.261540	1.375183	-0.465020
C	-7.385603	-0.394827	0.061856
C	-6.536734	1.811671	-0.649168
C	-7.614597	0.928001	-0.388152
H	3.183771	0.161468	-1.762494
H	5.117343	-1.748445	-0.401097
H	3.037742	-1.308518	0.918443
H	5.356998	1.231195	-0.978816
H	5.085174	-0.277076	1.657510
H	6.454288	1.792683	1.777251
H	4.941277	1.900686	2.693519
H	1.986352	-1.746243	-1.961606
H	5.115196	-1.763376	-2.718872
H	7.292912	-0.121129	-0.975462
H	-0.892710	2.264773	-0.626756
H	4.174948	3.300079	1.189388
H	0.124264	-2.938483	1.197248
H	2.544599	1.873909	-0.130975
H	-4.038991	-2.377135	0.883168
H	-5.945615	-1.832111	0.588397
H	-4.434193	2.038012	-0.665427
H	-8.234541	-1.035976	0.250082
H	-6.740967	2.818473	-0.990235
H	-8.942855	2.206956	-0.860490

ENERGIES
SCF ENERGY: -1564.61521305
SUM OF ELECTRONIC AND ZERO-POINT ENERGIES: -1564.215569
SUM OF ELECTRONIC AND THERMAL ENERGIES: -1564.188410
SUM OF ELECTRONIC AND THERMAL ENTHALPIES: -1564.187465
SUM OF ELECTRONIC AND THERMAL FREE ENERGIES: -1564.273764

==> ISOVITEXIN/LIPID/BDE/C4' <==
50
XYZ-COORDINATES + ENERGIES

O	3.425219	-0.729647	-0.837933
O	2.847156	1.917011	1.642158
O	5.452531	1.034440	2.279142
O	6.868711	-0.139344	0.151574
O	5.039123	-3.006626	-0.917077
O	0.846676	2.328758	-1.084198
O	1.623859	-2.061361	0.560560
O	-2.766867	-0.534798	0.148884
O	-1.607498	3.071398	-1.307843
O	-8.865771	-1.399256	0.587641
C	3.356169	0.710223	1.101922
C	4.856711	0.880522	1.000263
C	2.783123	0.434812	-0.296187
C	5.481704	-0.340688	0.358396
C	4.841899	-0.583412	-1.001109

```
C	1.302561	0.173701	-0.244739
C	5.341813	-1.843156	-1.676705
C	0.397830	1.161652	-0.614462
C	0.796024	-1.056960	0.221938
C	-0.996234	0.948688	-0.487889
C	-0.567529	-1.292622	0.358195
C	-1.437144	-0.281684	0.004566
C	-1.953524	1.966443	-0.860063
C	-3.691233	0.396151	-0.186223
C	-3.342803	1.608979	-0.678296
C	-5.056948	-0.070116	0.026767
C	-6.146506	0.837037	-0.042711
C	-5.298045	-1.437682	0.306895
C	-7.422615	0.403640	0.144300
C	-6.569727	-1.893769	0.492641
C	-7.706532	-0.994066	0.419721
H	3.135143	-0.132382	1.765522
H	5.069366	1.758043	0.378104
H	2.985680	1.294553	-0.937041
H	5.309052	-1.213444	0.996000
H	5.041723	0.274377	-1.652331
H	6.424209	-1.784837	-1.764854
H	4.909068	-1.909563	-2.676493
H	1.912261	1.805750	1.840742
H	5.044983	1.795564	2.704481
H	7.240790	0.153635	0.989692
H	-0.928212	-2.245560	0.714970
H	4.135424	-3.265704	-1.110521
H	0.051138	2.884330	-1.283518
H	2.506696	-1.867875	0.196731
H	-4.089623	2.331913	-0.963341
H	-5.966709	1.885401	-0.228118
H	-4.465856	-2.122713	0.362567
H	-8.262209	1.083597	0.102823
H	-6.771987	-2.935838	0.697997
```

ENERGIES
SCF ENERGY: -1564.21155288
SUM OF ELECTRONIC AND ZERO-POINT ENERGIES: -1563.825032
SUM OF ELECTRONIC AND THERMAL ENERGIES: -1563.798055
SUM OF ELECTRONIC AND THERMAL ENTHALPIES: -1563.797111
SUM OF ELECTRONIC AND THERMAL FREE ENERGIES: -1563.883462

==> ISOVITEXIN/LIPID/BDE/C4'/TS <==
54
XYZ-COORDINATES + ENERGIES

```
O	4.080381	-0.608052	-0.913018
O	3.433224	1.433887	2.070733
O	5.949729	0.282646	2.643541
O	7.467037	-0.440318	0.386870
O	5.632555	-2.891178	-1.413441
O	1.672080	2.582622	-0.609687
O	2.115308	-2.105150	0.032066
O	-2.156185	-0.245384	-0.277732
O	-0.714691	3.511880	-0.805925
O	-8.352588	-0.772888	-0.473643
C	3.931109	0.356134	1.297356
C	5.440785	0.461000	1.330460
C	3.451813	0.440666	-0.159290
C	6.062411	-0.611333	0.459976
C	5.509805	-0.504042	-0.954315
C	1.960985	0.268715	-0.260467
C	6.010924	-1.599399	-1.871723
C	1.134567	1.370569	-0.447375
C	1.362747	-0.999911	-0.117514
C	-0.273425	1.224447	-0.455418
C	-0.016611	-1.172557	-0.119396
C	-0.807965	-0.053807	-0.283902
C	-1.150694	2.360006	-0.643981
C	-3.002298	0.795025	-0.452367
C	-2.563079	2.059536	-0.638145
C	-4.408702	0.378552	-0.434680
C	-5.427203	1.323885	-0.217198
C	-4.742022	-0.968493	-0.634581
C	-6.745392	0.935932	-0.209102
C	-6.061282	-1.365183	-0.637938
C	-7.090412	-0.417139	-0.434459
H	3.626899	-0.602152	1.731319
H	5.732212	1.445251	0.945179
H	3.740954	1.408835	-0.571394
H	5.814311	-1.596436	0.867129
H	5.786028	0.467902	-1.377112
H	7.097816	-1.567150	-1.889248
H	5.635318	-1.421088	-2.880866
H	2.481024	1.337876	2.170995
H	5.542836	0.945950	3.209828
H	7.791372	-0.369195	1.290433
H	-0.447965	-2.156217	-0.010346
H	4.731012	-3.059262	-1.696770
H	0.918301	3.216749	-0.724988
H	3.029912	-1.891151	-0.225279
H	-3.256994	2.866984	-0.809158
H	-5.179158	2.358547	-0.030072
H	-3.962567	-1.697807	-0.795600
H	-7.540530	1.648639	-0.039301
H	-6.334201	-2.398997	-0.798746
H	-8.601029	-1.394750	0.445681
O	-8.626027	-1.850400	1.595064
O	-7.339025	-1.689473	2.004949
H	-7.356314	-0.903672	2.574441
```

ENERGIES
SCF ENERGY: -1715.77534051
SUM OF ELECTRONIC AND ZERO-POINT ENERGIES: -1715.364539
SUM OF ELECTRONIC AND THERMAL ENERGIES: -1715.334275
SUM OF ELECTRONIC AND THERMAL ENTHALPIES: -1715.333331
SUM OF ELECTRONIC AND THERMAL FREE ENERGIES: -1715.428280

==> ISOVITEXIN/LIPID/BDE/C5 <==
50
XYZ-COORDINATES + ENERGIES

```
O	3.481159	-0.756502	-0.820617
O	2.929619	1.995995	1.554114
O	5.483487	1.055319	2.279489
O	6.915295	-0.225389	0.227182
O	5.027801	-3.072783	-0.796319
O	0.958051	2.303448	-1.201581
O	1.632978	-2.036053	0.554265
O	-2.702564	-0.434059	0.106289
O	-1.704555	3.224992	-1.332789
O	-8.871394	-1.425667	0.653735
C	3.405516	0.753586	1.070306
C	4.913473	0.875308	0.992980
C	2.855132	0.438446	-0.333144
C	5.519058	-0.382423	0.406070
C	4.904110	-0.649964	-0.959795
C	1.375595	0.199568	-0.289199
C	5.384265	-1.943179	-1.584392
C	0.480894	1.275016	-0.721440
C	0.859193	-0.998334	0.176302
C	-0.968193	1.081801	-0.566220
C	-0.520737	-1.172332	0.286048
C	-1.397684	-0.126937	-0.077336
C	-1.968564	2.109689	-0.915940
C	-3.665329	0.467324	-0.223172
C	-3.345829	1.674441	-0.725776
C	-5.018141	-0.042538	0.022808
C	-6.092310	0.839690	0.180727
C	-5.257044	-1.415998	0.097044
C	-7.372841	0.365203	0.390204
C	-6.538608	-1.899699	0.305148
C	-7.598450	-1.008689	0.448378
H	3.145612	-0.057027	1.759021
H	5.162696	1.725889	0.347686
H	3.073746	1.275176	-0.997732
H	5.306744	-1.229567	1.066012
H	5.138853	0.182065	-1.632031
H	6.469801	-1.916636	-1.646432
H	4.975005	-2.029294	-2.592240
H	2.004250	1.917050	1.803954
H	5.101941	1.851904	2.661995
H	7.274681	0.090954	1.062388
H	-0.924630	-2.104528	0.653371
H	4.139032	-3.341578	-1.038683
H	2.530725	-1.883080	0.208025
H	-4.124558	2.362943	-1.016075
```

H -5.925809 1.907559 0.160041
H -4.441947 -2.115318 -0.019759
H -8.204343 1.043678 0.520604
H -6.717802 -2.966788 0.353873
H -8.904048 -2.387676 0.678241

ENERGIES
SCF ENERGY: -1564.19131266
SUM OF ELECTRONIC AND ZERO-POINT ENERGIES: -1563.805299
SUM OF ELECTRONIC AND THERMAL ENERGIES: -1563.777845
SUM OF ELECTRONIC AND THERMAL ENTHALPIES: -1563.776901
SUM OF ELECTRONIC AND THERMAL FREE ENERGIES: -1563.864230

==> ISOVITEXIN/LIPID/BDE/C7 <==
50
XYZ-COORDINATES + ENERGIES
O 3.409806 -0.612962 -0.905879
O 3.125253 1.573977 2.035007
O 5.790299 0.654380 2.222710
O 6.948163 -0.158224 -0.209615
O 4.842284 -2.869712 -1.388861
O 0.931696 2.298445 -0.982436
O 1.604486 -1.909110 1.033392
O -2.753622 -0.492806 0.246419
O -1.485730 3.011693 -1.358988
O -8.964730 -1.230525 0.418188
C 3.570493 0.470986 1.254105
C 5.047889 0.686028 1.011551
C 2.862039 0.431780 -0.104988
C 5.591007 -0.410268 0.120929
C 4.801537 -0.459309 -1.181422
C 1.381531 0.212089 0.006221
C 5.169491 -1.651470 -2.041479
C 0.498814 1.154093 -0.449280
C 0.866505 -1.022312 0.575925
C -0.929993 0.939356 -0.375836
C -0.569932 -1.228231 0.626285
C -1.418324 -0.248119 0.156130
C -1.876591 1.941548 -0.867468
C -3.649263 0.411985 -0.199646
C -3.259628 1.589644 -0.751517
C -5.031733 -0.030040 -0.027815
C -6.082519 0.895463 -0.046195
C -5.324680 -1.384408 0.155413
C -7.389931 0.480470 0.104749
C -6.633884 -1.808260 0.301079
C -7.668162 -0.875182 0.275431
H 3.418101 -0.470858 1.782117
H 5.186618 1.653791 0.514248
H 3.035760 1.392566 -0.596373
H 5.493588 -1.369466 0.637485
H 4.976609 0.467736 -1.739908
H 6.240917 -1.655612 -2.227594
H 4.640329 -1.572204 -2.994681
H 2.247534 1.382413 2.376916
H 5.408769 1.311073 2.813662
H 7.414882 -0.001184 0.617215
H -0.946191 -2.150227 1.044359
H 3.940519 -2.772603 -1.064444
H 0.133273 2.825759 -1.246255
H -3.994909 2.279734 -1.133950
H -5.878639 1.951256 -0.155233
H -4.528700 -2.114493 0.172173
H -8.205120 1.190297 0.102275
H -6.856306 -2.860136 0.432975
H -9.038270 -2.184136 0.532492

ENERGIES
SCF ENERGY: -1564.19703791
SUM OF ELECTRONIC AND ZERO-POINT ENERGIES: -1563.811390
SUM OF ELECTRONIC AND THERMAL ENERGIES: -1563.784086
SUM OF ELECTRONIC AND THERMAL ENTHALPIES: -1563.783142
SUM OF ELECTRONIC AND THERMAL FREE ENERGIES: -1563.870500

==> ISOVITEXIN/LIPID/RAF/C1' <==
54
XYZ-COORDINATES + ENERGIES
O 3.767765 -0.887460 -0.764771
O 3.363300 2.226429 1.140904
O 6.021602 1.478878 1.737559
O 7.273397 -0.119853 -0.208981
O 5.414091 -3.137518 -0.514492
O 1.146324 2.060332 -1.454009
O 2.098388 -1.871038 1.041640
O -2.329643 -0.442248 0.646636
O -1.318989 2.752536 -1.644905
O -8.161890 -0.932000 -1.613726
C 3.839686 0.929167 0.828574
C 5.325328 1.065111 0.571977
C 3.157804 0.369096 -0.428482
C 5.910204 -0.266108 0.148829
C 5.163829 -0.781912 -1.073342
C 1.688911 0.135706 -0.204751
C 5.626913 -2.153116 -1.518100
C 0.747810 1.025491 -0.708935
C 1.233678 -0.959553 0.558044
C -0.630184 0.853860 -0.432529
C -0.111396 -1.150175 0.849757
C -1.017125 -0.235372 0.350863
C -1.623787 1.774523 -0.943960
C -3.271021 0.404331 0.184296
C -2.991860 1.473821 -0.579814
C -4.663225 -0.046310 0.586263
C -5.712079 0.957383 0.239884
C -4.928631 -1.403447 0.013435
C -6.827396 0.631577 -0.450734
C -6.061325 -1.685961 -0.682268
C -7.026357 -0.687337 -0.925003
H 3.681787 0.247849 1.671132
H 5.476846 1.792762 -0.234073
H 3.298936 1.073853 -1.249566
H 5.801125 -0.986230 0.965828
H 5.295883 -0.075950 -1.900370
H 6.696456 -2.113117 -1.710721
H 5.111154 -2.423539 -2.441282
H 2.449131 2.166031 1.435313
H 5.637688 2.311776 2.029520
H 7.709036 0.339743 0.516042
H -0.434152 -1.996734 1.436821
H 4.495044 -3.411909 -0.552923
H 0.329078 2.562885 -1.705426
H 2.945403 -1.769679 0.572259
H -3.781829 2.113287 -0.941532
H -5.569323 1.958746 0.622305
H -4.180865 -2.164309 0.189175
H -7.592216 1.370226 -0.650750
H -6.224627 -2.686537 -1.067133
H -8.194640 -1.855661 -1.886402
O -4.532074 -0.132114 2.050127
O -5.771205 -0.551287 2.588266
H -5.697102 -1.515420 2.563781

ENERGIES
SCF ENERGY: -1715.77825004
SUM OF ELECTRONIC AND ZERO-POINT ENERGIES: -1715.361463
SUM OF ELECTRONIC AND THERMAL ENERGIES: -1715.331423
SUM OF ELECTRONIC AND THERMAL ENTHALPIES: -1715.330478
SUM OF ELECTRONIC AND THERMAL FREE ENERGIES: -1715.422764

==> ISOVITEXIN/LIPID/RAF/C2 <==
54
XYZ-COORDINATES + ENERGIES
O 3.766008 -0.942934 -0.723153
O 3.119794 2.139636 1.166203
O 5.747689 1.471774 1.955161
O 7.194126 -0.065167 0.094002
O 5.474401 -3.139881 -0.361811
O 1.137126 1.959163 -1.579048
O 1.956962 -2.014391 0.883807
O -2.458114 -0.676190 0.157695
O -1.336185 2.575070 -2.015086
O -8.630516 -1.679735 0.018068
C 3.659536 0.861746 0.878450
C 5.155171 1.047187 0.737196
C 3.094809 0.293537 -0.432086
C 5.814032 -0.260318 0.349700

C	5.176970	-0.790773	-0.926782
C	1.619133	0.022954	-0.328333
C	5.717245	-2.141952	-1.344922
C	0.695729	0.909935	-0.879138
C	1.125406	-1.096609	0.360886
C	-0.691656	0.717175	-0.699953
C	-0.241887	-1.316538	0.541858
C	-1.126511	-0.404283	0.019011
C	-1.659801	1.627687	-1.273988
C	-3.369733	0.390730	0.122970
C	-3.033701	1.392751	-0.929609
C	-4.764522	-0.179584	0.039334
C	-5.845234	0.600763	-0.375015
C	-5.003537	-1.486781	0.462756
C	-7.133267	0.088628	-0.376122
C	-6.286856	-2.008546	0.459708
C	-7.354730	-1.219400	0.039937
H	3.459117	0.165524	1.699584
H	5.342956	1.787008	-0.050079
H	3.283023	1.006927	-1.236127
H	5.670013	-0.990455	1.152128
H	5.345693	-0.074750	-1.738420
H	6.795064	-2.062856	-1.466668
H	5.273233	-2.423972	-2.301321
H	2.188219	2.046756	1.389622
H	5.314833	2.288410	2.223641
H	7.559019	0.403195	0.851762
H	-0.587036	-2.190038	1.074302
H	4.573900	-3.454546	-0.468405
H	0.344210	2.439829	-1.917304
H	2.843717	-1.876147	0.505508
H	-3.784139	2.034614	-1.359763
H	-5.703152	1.621776	-0.698269
H	-4.182946	-2.105681	0.793110
H	-7.970612	0.691064	-0.699534
H	-6.459750	-3.027870	0.782874
H	-8.656161	-2.593828	0.319248
O	-3.145118	1.004894	1.431687
O	-3.971573	2.146918	1.535880
H	-4.765583	1.791923	1.961717

ENERGIES
SCF ENERGY: -1715.79232566
SUM OF ELECTRONIC AND ZERO-POINT ENERGIES: -1715.375168
SUM OF ELECTRONIC AND THERMAL ENERGIES: -1715.345159
SUM OF ELECTRONIC AND THERMAL ENTHALPIES: -1715.344215
SUM OF ELECTRONIC AND THERMAL FREE ENERGIES: -1715.437073

==> ISOVITEXIN/LIPID/RAF/C2' <==
54
XYZ-COORDINATES + ENERGIES

O	3.820152	-0.434234	-1.050900
O	3.208093	1.290726	2.133279
O	5.771837	0.167121	2.520786
O	7.239762	-0.282781	0.162232
O	5.418554	-2.601992	-1.826191
O	1.343942	2.645616	-0.395535
O	1.915463	-2.069994	-0.185119
O	-2.403769	-0.299515	-0.281799
O	-1.056255	3.528345	-0.458305
O	-8.641207	-0.996870	-0.410218
C	3.711298	0.308139	1.245092
C	5.217815	0.456382	1.246180
C	3.185405	0.518210	-0.182275
C	5.843365	-0.505259	0.257361
C	5.243212	-0.278186	-1.122944
C	1.697584	0.316486	-0.263733
C	5.748249	-1.259767	-2.159326
C	0.841109	1.409204	-0.336356
C	1.132309	-0.974514	-0.223307
C	-0.562155	1.227971	-0.338372
C	-0.241464	-1.181580	-0.217816
C	-1.063425	-0.072925	-0.272396
C	-1.469638	2.355311	-0.400616
C	-3.281186	0.731992	-0.329706
C	-2.869445	2.025139	-0.376911
C	-4.660836	0.284293	-0.320035
C	-5.687353	1.087014	-0.771183
C	-4.944607	-1.075560	0.245987
C	-7.006024	0.649304	-0.763339
C	-6.366633	-1.507696	0.132596
C	-7.325028	-0.669302	-0.315782
H	3.447940	-0.696276	1.592655
H	5.468522	1.482426	0.951730
H	3.437744	1.529572	-0.505353
H	5.637562	-1.532973	0.571912
H	5.475680	0.739814	-1.454281
H	6.832936	-1.194091	-2.200957
H	5.339772	-0.991840	-3.135507
H	2.260414	1.162511	2.241852
H	5.361409	0.757162	3.160835
H	7.588939	-0.287722	1.059310
H	-0.648253	-2.181138	-0.189001
H	4.504961	-2.757310	-2.076551
H	0.566411	3.264433	-0.434982
H	2.816982	-1.811319	-0.444337
H	-3.586302	2.830885	-0.371890
H	-5.471615	2.065410	-1.176875
H	-4.281652	-1.822770	-0.194568
H	-7.803439	1.282023	-1.123575
H	-6.609113	-2.518971	0.434831
H	-8.778005	-1.895848	-0.093074
O	-4.541251	-0.954280	1.641076
O	-4.471078	-2.267169	2.180452
H	-5.250145	-2.291696	2.751048

ENERGIES
SCF ENERGY: -1715.78578797
SUM OF ELECTRONIC AND ZERO-POINT ENERGIES: -1715.368580
SUM OF ELECTRONIC AND THERMAL ENERGIES: -1715.338462
SUM OF ELECTRONIC AND THERMAL ENTHALPIES: -1715.337518
SUM OF ELECTRONIC AND THERMAL FREE ENERGIES: -1715.430766

==> ISOVITEXIN/LIPID/RAF/C3 <==
54
XYZ-COORDINATES + ENERGIES

O	3.731924	-0.878404	-0.822900
O	3.181147	1.937250	1.470460
O	5.829376	1.162716	2.072133
O	7.194467	-0.140374	-0.014654
O	5.407159	-3.118429	-0.778544
O	1.075569	2.094197	-1.186140
O	2.013839	-2.133309	0.734341
O	-2.418129	-0.761063	0.421107
O	-1.409258	2.809262	-1.269706
O	-8.587765	-2.051987	0.238393
C	3.700024	0.704413	1.003725
C	5.191340	0.898765	0.832432
C	3.082974	0.311596	-0.347058
C	5.821505	-0.354386	0.261556
C	5.138468	-0.716163	-1.049690
C	1.612070	0.023367	-0.220579
C	5.645601	-2.014054	-1.642189
C	0.666082	0.956882	-0.621765
C	1.153199	-1.174458	0.362705
C	-0.722967	0.722147	-0.427972
C	-0.199386	-1.425633	0.584434
C	-1.117470	-0.478726	0.189686
C	-1.694696	1.702395	-0.790916
C	-3.424409	0.042834	-0.031035
C	-3.149739	1.418979	-0.493958
C	-4.731411	-0.504011	0.055407
C	-5.864056	0.276176	-0.271780
C	-4.941868	-1.838286	0.463889
C	-7.133823	-0.255923	-0.209853
C	-6.215932	-2.366499	0.530194
C	-7.316842	-1.579890	0.191102
H	3.524584	-0.089790	1.736998
H	5.359858	1.730169	0.137572
H	3.243223	1.123551	-1.058103
H	5.694108	-1.178749	0.970247
H	5.293972	0.091714	-1.772700
H	6.721487	-1.935557	-1.780410
H	5.175039	-2.173289	-2.613913
H	2.266042	1.814270	1.741197
H	5.418582	1.944767	2.454294

H	7.590567	0.225090	0.783019
H	-0.515309	-2.354381	1.034948
H	4.507560	-3.422183	-0.918578
H	0.280870	2.646317	-1.363804
H	2.879453	-1.946920	0.325677
H	-5.748547	1.312127	-0.557052
H	-4.096724	-2.457656	0.724250
H	-7.999162	0.343087	-0.457730
H	-6.362148	-3.393235	0.843988
H	-8.587937	-2.967169	0.537250
H	-3.746367	1.659685	-1.375819
O	-3.536751	2.356717	0.547698
O	-3.845527	3.602284	-0.064041
H	-2.979550	3.900116	-0.386523

\# ENERGIES

SCF ENERGY: -1715.79799359

SUM OF ELECTRONIC AND ZERO-POINT ENERGIES: -1715.379561

SUM OF ELECTRONIC AND THERMAL ENERGIES: -1715.349652

SUM OF ELECTRONIC AND THERMAL ENTHALPIES: -1715.348708

SUM OF ELECTRONIC AND THERMAL FREE ENERGIES: -1715.440993

==> ISOVITEXIN/LIPID/RAF/C3/TS <==

54

XYZ-COORDINATES + ENERGIES

O	3.741577	-0.918199	-0.776833
O	3.066490	1.951955	1.413539
O	5.710660	1.248590	2.114610
O	7.163537	-0.071783	0.096717
O	5.483658	-3.113123	-0.631570
O	1.055908	1.999931	-1.333842
O	1.984420	-2.153996	0.767426
O	-2.455476	-0.821948	0.264939
O	-1.435372	2.651891	-1.579991
O	-8.630941	-1.922247	0.436164
C	3.623476	0.717616	0.997439
C	5.114934	0.939230	0.864268
C	3.056334	0.273085	-0.359269
C	5.789324	-0.312876	0.343861
C	5.149224	-0.726939	-0.973722
C	1.586192	-0.031896	-0.269570
C	5.704850	-2.025143	-1.519894
C	0.644854	0.879903	-0.733370
C	1.123696	-1.215918	0.337083
C	-0.740768	0.642081	-0.561638
C	-0.231760	-1.474983	0.521690
C	-1.138377	-0.539188	0.074215
C	-1.727909	1.590281	-1.004496
C	-3.425572	0.010313	-0.177316
C	-3.118847	1.273546	-0.678281
C	-4.765161	-0.503274	-0.005902
C	-5.876557	0.348814	-0.119275
C	-4.982029	-1.858685	0.280727
C	-7.157936	-0.138617	0.029686
C	-6.264730	-2.351244	0.429345
C	-7.355674	-1.492646	0.300725
H	3.442321	-0.059216	1.747768
H	5.285796	1.756741	0.153894
H	3.225867	1.065486	-1.090102
H	5.663998	-1.120745	1.071490
H	5.302042	0.065555	-1.714235
H	6.780436	-1.918640	-1.640500
H	5.257802	-2.224443	-2.495432
H	2.142534	1.820330	1.648249
H	5.272083	2.030767	2.464174
H	7.531268	0.318645	0.896096
H	-0.556459	-2.392596	0.988502
H	4.588934	-3.433878	-0.765190
H	0.250142	2.512200	-1.582782
H	2.858946	-1.971215	0.378920
H	-5.737595	1.405534	-0.298760
H	-4.142422	-2.531205	0.376782
H	-8.015476	0.514980	-0.047020
H	-6.425179	-3.400833	0.642967
H	-8.644771	-2.865067	0.632613
H	-3.874471	1.786891	-1.252356
O	-3.183444	2.503096	0.745238
O	-3.455429	3.725788	0.167906
H	-2.615878	3.980308	-0.247333

\# ENERGIES

SCF ENERGY: -1715.78002059

SUM OF ELECTRONIC AND ZERO-POINT ENERGIES: -1715.363403

SUM OF ELECTRONIC AND THERMAL ENERGIES: -1715.333442

SUM OF ELECTRONIC AND THERMAL ENTHALPIES: -1715.332498

SUM OF ELECTRONIC AND THERMAL FREE ENERGIES: -1715.425494

==> ISOVITEXIN/LIPID/RAF/C3' <==

54

XYZ-COORDINATES + ENERGIES

O	3.967786	-0.687318	-0.912901
O	3.482889	1.615834	1.906040
O	5.998977	0.463238	2.476605
O	7.407385	-0.491528	0.236744
O	5.431579	-3.037020	-1.288237
O	1.623619	2.562553	-0.787029
O	2.019890	-2.064031	0.236818
O	-2.229665	-0.149983	-0.051933
O	-0.749046	3.517244	-0.964160
O	-8.531780	-0.427067	0.012524
C	3.928475	0.464621	1.210641
C	5.439809	0.538038	1.173999
C	3.388057	0.433083	-0.226527
C	6.003145	-0.620009	0.376729
C	5.395578	-0.618848	-1.018971
C	1.891859	0.280713	-0.250697
C	5.835542	-1.799107	-1.859508
C	1.075746	1.378703	-0.498238
C	1.279706	-0.959358	0.024769
C	-0.333111	1.259897	-0.436421
C	-0.100655	-1.103793	0.094821
C	-0.880610	0.012802	-0.132759
C	-1.200066	2.392855	-0.684951
C	-3.064336	0.887908	-0.280695
C	-2.613606	2.121315	-0.594598
C	-4.482758	0.506872	-0.166113
C	-5.462423	1.516191	0.040065
C	-4.841412	-0.804098	-0.281075
C	-6.819008	1.180854	0.102928
C	-6.256037	-1.247167	-0.181795
C	-7.221793	-0.119345	-0.015216
H	3.627074	-0.445560	1.739377
H	5.734295	1.477771	0.692291
H	3.672960	1.357498	-0.731649
H	5.750078	-1.560799	0.875401
H	5.673301	0.307700	-1.533091
H	6.921884	-1.804267	-1.910600
H	5.434848	-1.689510	-2.868758
H	2.533929	1.549553	2.051441
H	5.629317	1.183085	2.997561
H	7.767639	-0.347354	1.117721
H	-0.543590	-2.065081	0.307900
H	4.515434	-3.193635	-1.527937
H	0.871873	3.196979	-0.924237
H	2.926030	-1.890577	-0.074253
H	-3.304989	2.922104	-0.802685
H	-5.164181	2.543929	0.174369
H	-4.106747	-1.576248	-0.450050
H	-7.567323	1.946986	0.252894
H	-6.534096	-1.865699	-1.043823
H	-8.626669	-1.384640	0.108636
O	-6.302928	-2.140775	0.969204
O	-7.525009	-2.888920	0.841326
H	-7.733271	-3.052797	1.771165

\# ENERGIES

SCF ENERGY: -1715.78659573

SUM OF ELECTRONIC AND ZERO-POINT ENERGIES: -1715.368709

SUM OF ELECTRONIC AND THERMAL ENERGIES: -1715.338773

SUM OF ELECTRONIC AND THERMAL ENTHALPIES: -1715.337828

SUM OF ELECTRONIC AND THERMAL FREE ENERGIES: -1715.430424

==> ISOVITEXIN/LIPID/RAF/C4 <==

54

XYZ-COORDINATES + ENERGIES

O	3.596181	-0.777275	-0.892981

O 3.111381 1.535301 1.919843
O 5.747594 0.614311 2.329085
O 7.076478 -0.259478 0.005606
O 5.241466 -2.998205 -1.334220
O 1.051046 2.272250 -0.622953
O 1.856451 -2.293687 0.414748
O -2.552444 -0.764976 0.350270
O -1.865415 2.756890 0.793805
O -8.701451 -1.952100 0.306596
C 3.602713 0.410545 1.212058
C 5.095841 0.612769 1.067860
C 2.970369 0.303923 -0.183522
C 5.702427 -0.507575 0.249246
C 5.001330 -0.587559 -1.099529
C 1.493645 0.025265 -0.104447
C 5.484834 -1.741312 -1.952751
C 0.567741 1.047228 -0.312620
C 1.014560 -1.253613 0.224630
C -0.810083 0.820357 -0.173960
C -0.342597 -1.501380 0.366425
C -1.227138 -0.456338 0.171802
C -1.819749 1.930403 -0.271837
C -3.510598 0.151423 0.078808
C -3.223296 1.421475 -0.271790
C -4.861711 -0.409003 0.155748
C -5.972092 0.418014 0.364383
C -5.061907 -1.784551 0.013849
C -7.247524 -0.109618 0.411487
C -6.337448 -2.320604 0.059690
C -7.432558 -1.482338 0.254155
H 3.416665 -0.510304 1.774572
H 5.271881 1.564979 0.553337
H 3.143895 1.237641 -0.720041
H 5.570680 -1.456388 0.778492
H 5.161152 0.349501 -1.644240
H 6.560104 -1.649138 -2.086533
H 5.001085 -1.690849 -2.929896
H 2.180104 1.400729 2.122796
H 5.343684 1.302447 2.867291
H 7.484450 -0.066260 0.855830
H -0.699490 -2.489920 0.614197
H 4.323943 -3.235210 -1.487274
H 0.336829 2.817189 -0.985724
H 2.720939 -2.056549 0.038248
H -4.003579 2.127233 -0.500032
H -5.838661 1.479656 0.518019
H -4.216975 -2.438953 -0.142558
H -8.107232 0.522847 0.583014
H -6.485337 -3.387316 -0.055138
H -8.705012 -2.908968 0.197912
O -1.469348 2.627372 -1.493039
O -2.359497 3.709137 -1.704616
H -2.839211 3.406241 -2.487539

ENERGIES
SCF ENERGY: -1715.75264544
SUM OF ELECTRONIC AND ZERO-POINT ENERGIES: -1715.335226
SUM OF ELECTRONIC AND THERMAL ENERGIES: -1715.305122
SUM OF ELECTRONIC AND THERMAL ENTHALPIES: -1715.304178
SUM OF ELECTRONIC AND THERMAL FREE ENERGIES: -1715.396961

==> ISOVITEXIN/LIPID/RAF/C4a <==
54
XYZ-COORDINATES + ENERGIES
O 3.624762 -0.513256 -0.894032
O 2.758275 1.171168 2.252507
O 5.325935 0.125385 2.798177
O 6.952734 -0.271913 0.537387
O 5.315946 -2.635388 -1.563752
O 0.986548 2.447608 -0.290580
O 1.766393 -2.243758 -0.168038
O -2.654774 -0.698051 -0.156661
O -1.433234 3.154714 0.223255
O -8.801148 -1.606601 0.571717
C 3.346987 0.209942 1.394373
C 4.845083 0.403856 1.491476
C 2.905824 0.410813 -0.062047
C 5.560252 -0.535094 0.542323
C 5.044495 -0.319019 -0.873295
C 1.434595 0.145471 -0.242407
C 5.640167 -1.285767 -1.874960
C 0.551410 1.187771 -0.347078
C 0.936725 -1.186122 -0.210650
C -0.910841 0.960685 -0.595774
C -0.437851 -1.445973 -0.153703
C -1.318100 -0.411554 -0.239943
C -1.829516 1.998485 0.041272
C -3.541367 0.269711 0.124075
C -3.160989 1.564884 0.305333
C -4.909841 -0.232504 0.239038
C -6.001899 0.639335 0.147324
C -5.149736 -1.595092 0.439142
C -7.294713 0.168371 0.261812
C -6.442447 -2.073919 0.559774
C -7.516985 -1.192033 0.469923
H 3.093895 -0.804267 1.721056
H 5.082751 1.438152 1.215827
H 3.139437 1.432428 -0.365669
H 5.364393 -1.569844 0.839903
H 5.271004 0.705573 -1.187731
H 6.723522 -1.195067 -1.843291
H 5.292278 -1.024945 -2.875927
H 1.803982 1.045770 2.259001
H 4.862231 0.703218 3.412376
H 7.244112 -0.271361 1.454836
H -0.789405 -2.460151 -0.025260
H 4.449081 -2.828216 -1.927890
H 0.195230 3.030302 -0.213622
H 2.661994 -1.937021 -0.400735
H -3.877299 2.301807 0.632368
H -5.843804 1.692158 -0.038992
H -4.321566 -2.284578 0.509813
H -8.141266 0.835795 0.181733
H -6.621027 -3.129489 0.723822
H -8.832418 -2.560076 0.703950
O -1.179696 1.017198 -2.073287
O -0.855354 2.307609 -2.571231
H -1.715753 2.748598 -2.595315

ENERGIES
SCF ENERGY: -1715.76718053
SUM OF ELECTRONIC AND ZERO-POINT ENERGIES: -1715.350090
SUM OF ELECTRONIC AND THERMAL ENERGIES: -1715.319984
SUM OF ELECTRONIC AND THERMAL ENTHALPIES: -1715.319039
SUM OF ELECTRONIC AND THERMAL FREE ENERGIES: -1715.411578

==> ISOVITEXIN/LIPID/RAF/C4' <==
54
XYZ-COORDINATES + ENERGIES
O 4.068631 -0.770873 -0.852718
O 3.571542 1.876498 1.643737
O 6.152863 0.918210 2.269100
O 7.530358 -0.282687 0.129876
O 5.620549 -3.091070 -0.951181
O 1.581270 2.368366 -1.072990
O 2.227877 -2.053818 0.546420
O -2.114738 -0.387173 0.154462
O -0.838857 3.191688 -1.279689
O -7.927435 -0.533782 1.612083
C 4.045622 0.659869 1.094272
C 5.550048 0.788966 0.990440
C 3.461387 0.409134 -0.303601
C 6.138579 -0.445606 0.339731
C 5.488132 -0.662613 -1.019183
C 1.973906 0.193475 -0.249012
C 5.951777 -1.931078 -1.704165
C 1.098880 1.211891 -0.609591
C 1.429968 -1.021916 0.214151
C -0.299896 1.042285 -0.479031
C 0.060359 -1.214669 0.353915
C -0.779510 -0.174831 0.008238
C -1.226299 2.094360 -0.839973
C -3.016805 0.575117 -0.171906
C -2.620673 1.785202 -0.654698
C -4.380942 0.162406 0.048999
C -5.472799 1.050210 -0.212331

C	-4.664025	-1.148967	0.542041
C	-6.750307	0.674423	-0.014308
C	-5.931104	-1.564870	0.739474
C	-7.112291	-0.690677	0.471788
H	3.802573	-0.180791	1.752739
H	5.785971	1.664197	0.373516
H	3.687824	1.265721	-0.940670
H	5.943195	-1.316601	0.973121
H	5.709505	0.193414	-1.665841
H	7.035052	-1.901550	-1.795578
H	5.514059	-1.979395	-2.702841
H	2.629168	1.797177	1.821907
H	5.765135	1.685840	2.701173
H	7.912838	-0.004398	0.968312
H	-0.328560	-2.157841	0.706957
H	4.706130	-3.317636	-1.135182
H	0.797530	2.947519	-1.263072
H	3.114586	-1.886247	0.180184
H	-3.341203	2.542350	-0.915783
H	-5.278871	2.050441	-0.569365
H	-3.846066	-1.818647	0.759283
H	-7.569464	1.352614	-0.200935
H	-6.143030	-2.562948	1.103343
H	-7.942508	-1.366399	2.099498
O	-7.854824	-1.419221	-0.522649
O	-9.021207	-0.682913	-0.856311
H	-9.586135	-0.825302	-0.083400

ENERGIES
SCF ENERGY: -1715.79265226
SUM OF ELECTRONIC AND ZERO-POINT ENERGIES: -1715.375635
SUM OF ELECTRONIC AND THERMAL ENERGIES: -1715.345653
SUM OF ELECTRONIC AND THERMAL ENTHALPIES: -1715.344708
SUM OF ELECTRONIC AND THERMAL FREE ENERGIES: -1715.437482

==> ISOVITEXIN/LIPID/RAF/C5 <==
54
XYZ-COORDINATES + ENERGIES

O	3.524658	-0.883973	-0.679630
O	2.706055	1.673499	1.846546
O	5.328053	0.917703	2.542134
O	6.896825	-0.219131	0.495145
O	5.232630	-3.116170	-0.705542
O	0.923607	2.307387	-0.217440
O	1.577097	-2.339902	0.357749
O	-2.751565	-0.647190	0.079120
O	-1.545737	3.189880	-0.359246
O	-8.934963	-1.497630	0.631154
C	3.307342	0.532529	1.262732
C	4.810395	0.742030	1.232492
C	2.823868	0.258131	-0.172187
C	5.505356	-0.456888	0.624715
C	4.945017	-0.699089	-0.768993
C	1.352080	-0.027507	-0.218813
C	5.516190	-1.930840	-1.438310
C	0.461591	1.085154	-0.694927
C	0.817634	-1.266169	0.046176
C	-0.998555	0.892114	-0.377584
C	-0.570854	-1.476305	0.035650
C	-1.441614	-0.370272	-0.118045
C	-1.919752	2.009511	-0.303665
C	-3.673473	0.346505	0.030970
C	-3.306534	1.634574	-0.152888
C	-5.042325	-0.149868	0.199596
C	-6.075941	0.714819	0.577284
C	-5.337703	-1.496824	-0.021862
C	-7.369438	0.251296	0.717251
C	-6.633136	-1.968123	0.112601
C	-7.651269	-1.093117	0.481333
H	3.079776	-0.341468	1.880779
H	5.028867	1.626952	0.622367
H	3.046940	1.124417	-0.801504
H	5.320371	-1.339709	1.244191
H	5.152542	0.173175	-1.398408
H	6.597995	-1.827440	-1.483270
H	5.125749	-2.000032	-2.455075
H	2.079193	2.074029	1.225695
H	4.855528	1.654439	2.943664
H	7.211591	0.089084	1.351061
H	-0.973905	-2.458579	0.230567
H	4.358595	-3.424327	-0.954291
H	0.177067	2.945752	-0.303950
H	2.497647	-2.132133	0.122229
H	-4.050860	2.414713	-0.190282
H	-5.870744	1.755441	0.785014
H	-4.555265	-2.183055	-0.310893
H	-8.169159	0.913648	1.017594
H	-6.854058	-3.012808	-0.069124
H	-9.008181	-2.440825	0.451178
O	0.668516	1.003351	-2.127328
O	-0.042892	2.061364	-2.753570
H	-0.821243	1.605264	-3.101430

ENERGIES
SCF ENERGY: -1715.78637655
SUM OF ELECTRONIC AND ZERO-POINT ENERGIES: -1715.368866
SUM OF ELECTRONIC AND THERMAL ENERGIES: -1715.339156
SUM OF ELECTRONIC AND THERMAL ENTHALPIES: -1715.338212
SUM OF ELECTRONIC AND THERMAL FREE ENERGIES: -1715.429856

==> ISOVITEXIN/LIPID/RAF/C5' <==
54
XYZ-COORDINATES + ENERGIES

O	-4.121903	0.801810	-0.677895
O	-3.364240	-2.098670	1.443651
O	-5.961470	-1.379083	2.287431
O	-7.502764	-0.021394	0.362044
O	-5.834049	3.011405	-0.430760
O	-1.500168	-2.132698	-1.425687
O	-2.271419	1.994789	0.803589
O	2.120239	0.626779	-0.003116
O	0.938719	-2.780499	-1.868885
O	8.297092	1.581348	0.300982
C	-3.928957	-0.854271	1.070190
C	-5.427303	-1.060803	1.011380
C	-3.427362	-0.400275	-0.308585
C	-6.115020	0.203435	0.539537
C	-5.538950	0.624057	-0.805172
C	-1.951288	-0.112145	-0.294790
C	-6.109650	1.931215	-1.313192
C	-1.049455	-1.024621	-0.830951
C	-1.442744	1.059772	0.301204
C	0.344981	-0.798099	-0.744445
C	-0.079239	1.306336	0.403402
C	0.788534	0.367663	-0.118957
C	1.299966	-1.737124	-1.297880
C	3.033261	-0.231456	-0.510479
C	2.686364	-1.367562	-1.150754
C	4.413278	0.235339	-0.290528
C	5.424487	-0.671856	-0.155952
C	4.666471	1.629519	-0.226867
C	6.838617	-0.261001	0.048651
C	5.977206	2.098730	-0.035760
C	7.010173	1.220345	0.084286
H	-3.702023	-0.087844	1.818751
H	-5.641904	-1.868802	0.301969
H	-3.642499	-1.183153	-1.037834
H	-5.943860	1.002437	1.267567
H	-5.736379	-0.160872	-1.543306
H	-7.190915	1.835991	-1.379880
H	-5.710295	2.132801	-2.308828
H	-2.422088	-1.983910	1.602665
H	-5.514217	-2.170072	2.604560
H	-7.831584	-0.419895	1.174284
H	0.283672	2.211952	0.865692
H	-4.936523	3.310508	-0.593342
H	-0.700025	-2.635178	-1.732582
H	-3.166037	1.829916	0.457310
H	3.444070	-2.004985	-1.579509
H	5.231934	-1.734939	-0.157505
H	3.854213	2.331405	-0.326274
H	6.166464	3.163863	0.018203
H	8.371051	2.539683	0.388757
H	7.496478	-0.707216	-0.704776
O	7.260981	-0.823070	1.322645
O	8.662283	-1.052924	1.284468

H 9.023849 -0.167247 1.430305

ENERGIES
SCF ENERGY: -1715.78573486
SUM OF ELECTRONIC AND ZERO-POINT ENERGIES: -1715.368105
SUM OF ELECTRONIC AND THERMAL ENERGIES: -1715.338243
SUM OF ELECTRONIC AND THERMAL ENTHALPIES: -1715.337298
SUM OF ELECTRONIC AND THERMAL FREE ENERGIES: -1715.429659

==> ISOVITEXIN/LIPID/RAF/C6 <==
54
XYZ-COORDINATES + ENERGIES
O -2.867482 0.787928 -0.577311
O -4.003572 -2.196769 1.260192
O -6.265456 -0.581040 1.235758
O -6.448712 1.225293 -0.900028
O -3.625155 3.441922 -0.713335
O -0.916424 -1.747754 -1.809719
O -1.596426 1.320617 1.746082
O 2.784586 0.237698 0.442969
O 1.468841 -1.993739 -2.681960
O 8.978686 1.103698 0.723397
C -3.878424 -0.825984 0.926527
C -5.212979 -0.463249 0.292030
C -2.772149 -0.561917 -0.111800
C -5.229288 0.954855 -0.231730
C -4.098065 1.114772 -1.233120
C -1.303918 -0.696587 0.339876
C -3.948517 2.528793 -1.753498
C -0.398855 -1.059004 -0.798443
C -0.752505 0.511431 1.066049
C 0.967454 -0.809371 -0.715142
C 0.586799 0.729057 1.150696
C 1.453885 0.011864 0.303858
C 1.885571 -1.335931 -1.710322
C 3.669373 -0.284291 -0.435173
C 3.272063 -1.035905 -1.492794
C 5.053071 0.077601 -0.114140
C 6.126644 -0.638391 -0.656659
C 5.323091 1.151102 0.738241
C 7.430611 -0.285376 -0.368908
C 6.627679 1.512491 1.029733
C 7.683672 0.795633 0.473515
H -3.733638 -0.225414 1.826911
H -5.387150 -1.146747 -0.547706
H -2.936383 -1.253275 -0.939893
H -5.079764 1.656653 0.595413
H -4.256971 0.433422 -2.075429
H -4.895909 2.848157 -2.181202
H -3.184119 2.544793 -2.532806
H -3.321343 -2.419533 1.900627
H -6.234490 -1.476436 1.588673
H -7.160339 0.950334 -0.312816
H 0.972636 1.521336 1.776916
H -2.704770 3.302449 -0.476648
H -0.138768 -1.993683 -2.390932
H -2.404616 1.415639 1.220599
H 3.993123 -1.400605 -2.206657
H 5.948178 -1.492980 -1.293575
H 4.510335 1.716095 1.170190
H 8.262259 -0.840714 -0.779474
H 6.827144 2.350183 1.686660
H 9.030034 1.858156 1.319614
O -1.141465 -1.889143 1.199393
O -1.639972 -1.618390 2.506061
H -0.836320 -1.419961 3.006760

ENERGIES
SCF ENERGY: -1715.77580607
SUM OF ELECTRONIC AND ZERO-POINT ENERGIES: -1715.358352
SUM OF ELECTRONIC AND THERMAL ENERGIES: -1715.328654
SUM OF ELECTRONIC AND THERMAL ENTHALPIES: -1715.327710
SUM OF ELECTRONIC AND THERMAL FREE ENERGIES: -1715.418515

==> ISOVITEXIN/LIPID/RAF/C6' <==
54
XYZ-COORDINATES + ENERGIES
O -3.950618 0.499574 -0.981780
O -3.306936 -1.487965 2.039228
O -5.961624 -0.604777 2.434758
O -7.384863 -0.016823 0.081624
O -5.687850 2.606128 -1.612529
O -1.240410 -2.417396 -0.509172
O -2.228233 2.191444 0.113217
O 2.226323 0.794393 0.132798
O 1.229627 -3.089481 -0.515888
O 8.376030 1.886028 0.940054
C -3.858020 -0.460794 1.233791
C -5.347281 -0.719881 1.160136
C -3.268347 -0.481296 -0.184192
C -6.011920 0.290948 0.248932
C -5.354363 0.244727 -1.123131
C -1.801459 -0.150339 -0.174717
C -5.902058 1.281067 -2.081493
C -0.850106 -1.156680 -0.302011
C -1.353142 1.172110 0.017889
C 0.530289 -0.863031 -0.199587
C -0.004034 1.488350 0.122890
C 0.913243 0.461040 0.018754
C 1.535226 -1.899591 -0.319210
C 3.195826 -0.150772 0.026995
C 2.900088 -1.458466 -0.192850
C 4.527724 0.386466 0.191220
C 5.695219 -0.548812 0.075260
C 4.733675 1.728431 0.429853
C 7.008629 0.054959 0.436360
C 6.009922 2.244120 0.641494
C 7.142578 1.380281 0.666961
H -3.687116 0.519498 1.690667
H -5.509309 -1.725434 0.754381
H -3.420476 -1.471631 -0.616371
H -5.890219 1.293729 0.670212
H -5.496090 -0.750938 -1.557543
H -6.976529 1.138786 -2.170549
H -5.443949 1.138498 -3.061890
H -2.375596 -1.301897 2.194504
H -5.529937 -1.228443 3.027121
H -7.757636 -0.134479 0.961345
H 0.311858 2.510778 0.265499
H -4.785115 2.856323 -1.822131
H -0.411804 -2.963918 -0.564709
H -3.092559 1.884848 -0.213465
H 3.685995 -2.188798 -0.298483
H 5.526391 -1.453985 0.663921
H 3.889182 2.399997 0.469754
H 7.872932 -0.592558 0.481891
H 6.140888 3.304394 0.812921
H 8.312656 2.825056 1.139660
O 5.689701 -0.965887 -1.322893
O 6.569003 -2.075155 -1.440635
H 7.345091 -1.677534 -1.857075

ENERGIES
SCF ENERGY: -1715.78626766
SUM OF ELECTRONIC AND ZERO-POINT ENERGIES: -1715.369514
SUM OF ELECTRONIC AND THERMAL ENERGIES: -1715.339234
SUM OF ELECTRONIC AND THERMAL ENTHALPIES: -1715.338290
SUM OF ELECTRONIC AND THERMAL FREE ENERGIES: -1715.431856

==> ISOVITEXIN/LIPID/RAF/C7 <==
54
XYZ-COORDINATES + ENERGIES
O 3.256696 0.030930 -1.152000
O 3.195515 0.357457 2.502059
O 5.879362 -0.322079 1.995939
O 6.835460 0.326050 -0.586216
O 4.885441 -1.496475 -2.908027
O 0.858840 2.626169 0.040522
O 1.429295 -1.969219 -0.577222
O -2.848392 -0.405154 -0.015386
O -1.559103 3.442698 0.026045
O -9.049178 -1.144064 -0.384469
C 3.591849 -0.094436 1.220684
C 5.042395 0.296588 1.030234
C 2.770723 0.559810 0.102860
C 5.523495 -0.151100 -0.334958

C	4.613098	0.413765	-1.417075
C	1.290797	0.324165	0.168152
C	4.962067	-0.081868	-2.803977
C	0.422553	1.361123	0.111339
C	0.784805	-1.084868	0.277498
C	-1.017579	1.148369	0.090440
C	-0.686292	-1.251545	0.095909
C	-1.508595	-0.156114	0.061044
C	-1.950914	2.262687	0.025972
C	-3.736894	0.609626	-0.067123
C	-3.338798	1.905597	-0.054736
C	-5.120755	0.138955	-0.146128
C	-6.187404	0.994814	0.154615
C	-5.398110	-1.177688	-0.523547
C	-7.492661	0.553399	0.071121
C	-6.704601	-1.626082	-0.614271
C	-7.753808	-0.759732	-0.317487
H	3.508362	-1.181863	1.155952
H	5.130572	1.387210	1.102773
H	2.947952	1.636104	0.122732
H	5.496987	-1.243740	-0.380402
H	4.681582	1.507347	-1.408707
H	5.988969	0.197823	-3.026828
H	4.299171	0.396778	-3.528103
H	2.380034	-0.105980	2.722172
H	5.514471	-0.120395	2.863638
H	7.380025	0.064903	0.163138
H	-1.082942	-2.253133	0.030862
H	3.959281	-1.747742	-2.872978
H	0.060048	3.214086	0.025549
H	2.241024	-1.550708	-0.901830
H	-4.060745	2.702677	-0.135354
H	-6.001404	2.009306	0.477120
H	-4.590436	-1.855061	-0.758535
H	-8.319175	1.207505	0.310716
H	-6.912365	-2.645506	-0.915474
H	-9.108713	-2.065511	-0.658364
O	1.092940	-1.419050	1.669592
O	0.754296	-2.776114	1.926526
H	0.005724	-2.676075	2.529643

ENERGIES
SCF ENERGY: -1715.78045120
SUM OF ELECTRONIC AND ZERO-POINT ENERGIES: -1715.363281
SUM OF ELECTRONIC AND THERMAL ENERGIES: -1715.333289
SUM OF ELECTRONIC AND THERMAL ENTHALPIES: -1715.332345
SUM OF ELECTRONIC AND THERMAL FREE ENERGIES: -1715.424946

==> ISOVITEXIN/LIPID/RAF/C8 <==
54
XYZ-COORDINATES + ENERGIES

O	3.495215	-0.403555	-1.082439
O	2.999495	1.700883	1.886593
O	5.599266	0.677304	2.284334
O	6.963903	-0.064504	-0.063001
O	5.070017	-2.631946	-1.657597
O	0.961643	2.696673	-0.623437
O	1.690629	-1.980268	0.024805
O	-2.704901	-0.311818	-0.040754
O	-1.486753	3.481961	-0.739063
O	-8.903213	-1.200493	0.064742
C	3.481467	0.623783	1.103254
C	4.982651	0.791575	1.011135
C	2.881174	0.638560	-0.309761
C	5.577038	-0.284035	0.125920
C	4.910497	-0.240584	-1.241762
C	1.396519	0.384464	-0.269597
C	5.382639	-1.341460	-2.168065
C	0.491688	1.462566	-0.402059
C	0.907078	-0.881510	-0.071440
C	-0.909444	1.234814	-0.317241
C	-0.524345	-1.204664	0.169306
C	-1.384024	-0.029224	-0.081266
C	-1.866845	2.319312	-0.524241
C	-3.621773	0.659733	-0.227927
C	-3.249506	1.941643	-0.471864
C	-4.997254	0.161480	-0.145395
C	-6.072101	1.046619	-0.001397
C	-5.258057	-1.209711	-0.206439
C	-7.369729	0.579049	0.066662
C	-6.556554	-1.685775	-0.141781
C	-7.614594	-0.791069	-0.006480
H	3.257827	-0.333205	1.586151
H	5.200547	1.773271	0.574252
H	3.082381	1.604980	-0.774391
H	5.400176	-1.264885	0.578470
H	5.110949	0.729353	-1.709730
H	6.464680	-1.279547	-2.258407
H	4.936997	-1.196884	-3.153586
H	2.069969	1.555530	2.089308
H	5.204390	1.337637	2.862650
H	7.353455	0.055128	0.809173
H	-0.846705	-2.073165	-0.410674
H	4.169204	-2.845486	-1.910479
H	0.169108	3.285718	-0.713543
H	2.582578	-1.749117	-0.295936
H	-3.991410	2.703441	-0.650458
H	-5.899745	2.110517	0.077762
H	-4.444847	-1.912609	-0.311142
H	-8.201699	1.258950	0.185677
H	-6.749766	-2.750098	-0.194377
H	-8.948292	-2.161219	0.015938
O	-0.697064	-1.586392	1.573893
O	-0.478948	-2.984313	1.694464
H	0.486745	-3.049954	1.685656

ENERGIES
SCF ENERGY: -1715.78712137
SUM OF ELECTRONIC AND ZERO-POINT ENERGIES: -1715.369195
SUM OF ELECTRONIC AND THERMAL ENERGIES: -1715.339035
SUM OF ELECTRONIC AND THERMAL ENTHALPIES: -1715.338091
SUM OF ELECTRONIC AND THERMAL FREE ENERGIES: -1715.431658

==> ISOVITEXIN/LIPID/RAF/C8a <==
54
XYZ-COORDINATES + ENERGIES

O	3.475736	-0.814805	-0.944582
O	3.225073	1.950088	1.463167
O	5.846602	0.981407	1.900006
O	7.015466	-0.322120	-0.304030
O	5.005586	-3.155869	-1.072752
O	1.019584	2.255632	-1.293968
O	1.760208	-2.010790	0.683570
O	-2.572116	-0.430645	0.121544
O	-1.361582	3.106031	-1.459338
O	-8.734809	-1.492724	-0.153270
C	3.640395	0.701501	0.937111
C	5.130909	0.805245	0.688537
C	2.935946	0.395440	-0.397047
C	5.648694	-0.462587	0.040800
C	4.876612	-0.732105	-1.243003
C	1.463564	0.205906	-0.195844
C	5.266138	-2.035286	-1.908175
C	0.543189	1.231052	-0.602917
C	0.946768	-0.968581	0.391394
C	-0.796381	1.135225	-0.323106
C	-0.384544	-1.097922	0.702418
C	-1.307167	0.057054	0.551736
C	-1.740794	2.081556	-0.852375
C	-3.486065	0.487683	-0.295514
C	-3.134191	1.728353	-0.705115
C	-4.853993	-0.041155	-0.265838
C	-5.897168	0.604040	-0.942537
C	-5.139680	-1.207853	0.448237
C	-7.185552	0.110640	-0.893998
C	-6.429807	-1.710131	0.501678
C	-7.455284	-1.049039	-0.168096
H	3.449328	-0.105061	1.651081
H	5.315935	1.650322	0.014679
H	3.114440	1.222187	-1.086277
H	5.507189	-1.303315	0.727092
H	5.045186	0.091195	-1.945414
H	6.335730	-2.017269	-2.104309
H	4.734276	-2.126402	-2.856775
H	2.377525	1.848077	1.906850
H	5.514418	1.778666	2.324923

H	7.478519	-0.021807	0.484726
H	-0.755987	-2.003691	1.155872
H	4.082457	-3.399145	-1.172176
H	0.223748	2.838922	-1.490883
H	2.589122	-1.900408	0.187221
H	-3.880921	2.438681	-1.021345
H	-5.702120	1.491318	-1.527771
H	-4.348216	-1.728486	0.967259
H	-7.991702	0.603248	-1.419371
H	-6.640762	-2.613315	1.061275
H	-8.799054	-2.299951	0.367911
O	-1.505450	0.707230	1.827073
O	-1.992998	-0.244806	2.763851
H	-2.949031	-0.107037	2.715189

ENERGIES
SCF ENERGY: -1715.78291039
SUM OF ELECTRONIC AND ZERO-POINT ENERGIES: -1715.366156
SUM OF ELECTRONIC AND THERMAL ENERGIES: -1715.336351
SUM OF ELECTRONIC AND THERMAL ENTHALPIES: -1715.335407
SUM OF ELECTRONIC AND THERMAL FREE ENERGIES: -1715.426659

==> ISOVITEXIN/WATER <==
51
XYZ-COORDINATES + ENERGIES

O	3.463185	-0.756194	-0.810539
O	2.790749	1.921271	1.555238
O	5.465096	1.037722	2.313790
O	6.942516	-0.166651	0.200396
O	5.175508	-3.030541	-0.830612
O	0.912115	2.322228	-1.082369
O	1.647331	-2.075920	0.609834
O	-2.733599	-0.525016	0.127841
O	-1.545413	3.065542	-1.356817
O	-8.937613	-1.275111	0.607568
C	3.398421	0.721363	1.093775
C	4.908774	0.882620	1.011461
C	2.824503	0.417396	-0.291709
C	5.540900	-0.337494	0.367552
C	4.878022	-0.610883	-0.981006
C	1.343759	0.157430	-0.241197
C	5.371528	-1.877303	-1.645950
C	0.445821	1.146132	-0.617567
C	0.826041	-1.062051	0.236751
C	-0.949609	0.945505	-0.509315
C	-0.537706	-1.290956	0.359327
C	-1.399373	-0.278545	-0.011858
C	-1.908619	1.956624	-0.897170
C	-3.649910	0.405822	-0.225654
C	-3.288768	1.608242	-0.734555
C	-5.024427	-0.046183	-0.000222
C	-6.072660	0.878290	0.073637
C	-5.308475	-1.407223	0.144381
C	-7.372295	0.455342	0.276671
C	-6.609270	-1.838855	0.343363
C	-7.639774	-0.905461	0.409011
H	3.164137	-0.104930	1.770311
H	5.135987	1.764154	0.401899
H	3.032888	1.258351	-0.955065
H	5.409762	-1.199373	1.021567
H	5.068423	0.235347	-1.651698
H	6.439727	-1.793753	-1.827437
H	4.858565	-1.996515	-2.601107
H	2.886032	1.960662	2.511988
H	5.274018	1.928336	2.624602
H	7.089647	0.595201	-0.372789
H	-0.909555	-2.235713	0.726660
H	4.230689	-3.204530	-0.773892
H	0.121305	2.876830	-1.299231
H	2.542654	-1.902298	0.266164
H	-4.039779	2.320000	-1.038815
H	-5.874931	1.937537	-0.008083
H	-4.512727	-2.135559	0.088993
H	-8.184860	1.165189	0.343713
H	-6.833801	-2.892624	0.447390
H	-8.998469	-2.233824	0.691832

ENERGIES
SCF ENERGY: -1564.87913096
SUM OF ELECTRONIC AND ZERO-POINT ENERGIES: -1564.479813
SUM OF ELECTRONIC AND THERMAL ENERGIES: -1564.452772
SUM OF ELECTRONIC AND THERMAL ENTHALPIES: -1564.451828
SUM OF ELECTRONIC AND THERMAL FREE ENERGIES: -1564.537158

==> ISOVITEXIN/WATER/AIP <==
51
XYZ-COORDINATES + ENERGIES

O	3.456156	-0.726162	-0.847150
O	2.783975	1.862568	1.613530
O	5.464471	0.961983	2.333001
O	6.937452	-0.165172	0.174946
O	5.170397	-3.001259	-0.935805
O	0.910340	2.365969	-0.976194
O	1.636291	-2.100429	0.494728
O	-2.739579	-0.539053	0.120521
O	-1.578509	3.125255	-1.199143
O	-8.861250	-1.305473	0.519836
C	3.395176	0.682741	1.108393
C	4.904809	0.850540	1.027927
C	2.821053	0.429324	-0.287290
C	5.537147	-0.346167	0.341660
C	4.871776	-0.577885	-1.013633
C	1.339582	0.169499	-0.241716
C	5.363603	-1.824143	-1.716896
C	0.442002	1.175411	-0.564969
C	0.821692	-1.069582	0.182835
C	-0.956721	0.970566	-0.456905
C	-0.544894	-1.302114	0.309482
C	-1.405805	-0.278595	-0.009353
C	-1.906682	1.996018	-0.791830
C	-3.661224	0.399260	-0.183987
C	-3.303792	1.638237	-0.629383
C	-5.017938	-0.044342	0.004577
C	-6.118169	0.826095	-0.257318
C	-5.262997	-1.372518	0.459241
C	-7.392319	0.393907	-0.081711
C	-6.534546	-1.814898	0.640921
C	-7.619510	-0.935048	0.368167
H	3.163497	-0.170061	1.752480
H	5.128650	1.752578	0.447742
H	3.028915	1.292838	-0.921177
H	5.410952	-1.229423	0.967385
H	5.061907	0.288363	-1.658139
H	6.431309	-1.734073	-1.898261
H	4.848726	-1.915240	-2.674042
H	2.880440	1.868829	2.570902
H	5.264964	1.837909	2.678329
H	7.078989	0.622088	-0.364350
H	-0.912992	-2.263317	0.635208
H	4.227317	-3.188314	-0.895703
H	0.137709	2.944703	-1.167068
H	2.533145	-1.912708	0.158983
H	-4.038802	2.385801	-0.877092
H	-5.952710	1.837032	-0.593978
H	-4.431025	-2.028822	0.659605
H	-8.245556	1.028046	-0.272226
H	-6.747083	-2.817971	0.985205
H	-8.939763	-2.221911	0.830786

ENERGIES
SCF ENERGY: -1564.64923930
SUM OF ELECTRONIC AND ZERO-POINT ENERGIES: -1564.250071
SUM OF ELECTRONIC AND THERMAL ENERGIES: -1564.222967
SUM OF ELECTRONIC AND THERMAL ENTHALPIES: -1564.222023
SUM OF ELECTRONIC AND THERMAL FREE ENERGIES: -1564.308452

==> ISOVITEXIN/WATER/BDE/C4'/TS <==
54
XYZ-COORDINATES + ENERGIES

O	4.096231	-0.696276	-0.845721
O	3.404141	1.698539	1.851112
O	5.949064	0.647205	2.584352
O	7.484398	-0.330715	0.415774
O	5.792816	-2.995768	-1.031035
O	1.651890	2.489313	-0.831428
O	2.146232	-2.129105	0.243688

O	-2.145260	-0.360960	-0.255626
O	-0.768869	3.364174	-1.112837
O	-8.333840	-1.006408	-0.471540
C	3.936338	0.535327	1.233187
C	5.445727	0.665037	1.252892
C	3.453439	0.422889	-0.219583
C	6.076032	-0.498374	0.514682
C	5.522836	-0.571207	-0.901975
C	1.965510	0.212633	-0.292643
C	6.040575	-1.755478	-1.688402
C	1.121817	1.279654	-0.568055
C	1.382658	-1.042157	-0.023269
C	-0.284009	1.120488	-0.564419
C	0.007445	-1.232912	-0.011133
C	-0.799227	-0.146140	-0.278410
C	-1.183681	2.215212	-0.844483
C	-3.007653	0.647775	-0.515161
C	-2.587359	1.899879	-0.804644
C	-4.407264	0.213492	-0.470275
C	-5.435973	1.157500	-0.317285
C	-4.718280	-1.150858	-0.587655
C	-6.749730	0.751461	-0.299651
C	-6.030756	-1.565836	-0.572502
C	-7.068744	-0.618357	-0.439665
H	3.643630	-0.360454	1.787618
H	5.729801	1.600156	0.759025
H	3.728508	1.334221	-0.752719
H	5.839692	-1.425359	1.043659
H	5.777351	0.348196	-1.439386
H	7.117618	-1.665734	-1.802511
H	5.579013	-1.749338	-2.676729
H	2.487681	1.531210	2.095046
H	5.621133	1.430372	3.039174
H	7.828544	-0.217712	1.308707
H	-0.414398	-2.205813	0.192065
H	4.843301	-3.152368	-1.026931
H	0.898161	3.104463	-1.004345
H	3.068838	-1.942617	-0.009563
H	-3.295369	2.680181	-1.035150
H	-5.202787	2.204866	-0.193843
H	-3.926549	-1.875907	-0.701459
H	-7.555771	1.461869	-0.178121
H	-6.291725	-2.610343	-0.670313
H	-8.691464	-1.148339	0.591970
O	-8.749428	-1.233401	1.838683
O	-7.460657	-1.011975	2.200254
H	-7.397146	-0.058688	2.379101

ENERGIES
SCF ENERGY: -1715.79442156
SUM OF ELECTRONIC AND ZERO-POINT ENERGIES: -1715.384106
SUM OF ELECTRONIC AND THERMAL ENERGIES: -1715.354127
SUM OF ELECTRONIC AND THERMAL ENTHALPIES: -1715.353183
SUM OF ELECTRONIC AND THERMAL FREE ENERGIES: -1715.446091

==> ISOVITEXIN/WATER/BDE/C5/TS <==
54
XYZ-COORDINATES + ENERGIES

O	-3.371285	-0.659908	0.797995
O	-2.852763	1.130469	-2.368192
O	-5.446919	-0.011114	-2.695261
O	-6.835790	-0.465796	-0.272177
O	-5.076248	-2.820476	1.588684
O	-0.918215	2.377431	-0.147836
O	-1.526446	-2.341283	-0.077865
O	2.796833	-0.627122	-0.105677
O	1.680014	3.258601	0.258636
O	8.984532	-1.592992	-0.280859
C	-3.343732	0.126122	-1.494115
C	-4.851677	0.277925	-1.435249
C	-2.765989	0.296849	-0.078708
C	-5.441076	-0.690370	-0.429225
C	-4.787901	-0.484625	0.929956
C	-1.282525	0.063217	-0.054424
C	-5.259475	-1.463156	1.983108
C	-0.405705	1.170556	-0.063262
C	-0.745713	-1.233803	-0.072231
C	1.013145	0.982981	-0.031476
C	0.623531	-1.429468	-0.100513
C	1.474086	-0.324333	-0.080158
C	1.987250	2.071681	0.090526
C	3.726305	0.357716	-0.065047
C	3.367262	1.656606	0.034498
C	5.094689	-0.161298	-0.121552
C	6.163896	0.677574	-0.456609
C	5.350944	-1.506700	0.158116
C	7.455548	0.188215	-0.504559
C	6.643002	-2.002676	0.116226
C	7.694512	-1.153841	-0.216580
H	-3.093703	-0.868564	-1.871979
H	-5.093357	1.302043	-1.132862
H	-2.992204	1.302898	0.277291
H	-5.255625	-1.712482	-0.769775
H	-4.987421	0.533378	1.279701
H	-6.323953	-1.321448	2.150444
H	-4.725976	-1.261030	2.912846
H	-1.971511	0.886053	-2.669454
H	-5.169762	0.665459	-3.321884
H	-7.239530	-0.511402	-1.145917
H	1.035723	-2.427989	-0.122660
H	-4.131872	-3.003259	1.560088
H	-2.434737	-2.090054	0.171207
H	4.121839	2.423383	0.115518
H	5.991355	1.715840	-0.702128
H	4.539873	-2.169980	0.420295
H	8.283766	0.829379	-0.772161
H	6.843253	-3.042846	0.338750
H	9.025800	-2.535022	-0.076756
H	-1.050310	2.793559	0.903943
O	-1.130623	2.890966	2.139262
O	-0.780076	1.648268	2.552256
H	0.181993	1.680707	2.691430

ENERGIES
SCF ENERGY: -1715.78544385
SUM OF ELECTRONIC AND ZERO-POINT ENERGIES: -1715.375551
SUM OF ELECTRONIC AND THERMAL ENERGIES: -1715.345017
SUM OF ELECTRONIC AND THERMAL ENTHALPIES: -1715.344073
SUM OF ELECTRONIC AND THERMAL FREE ENERGIES: -1715.438899

==> ISOVITEXIN/WATER/BDE/C7/TS <==
54
XYZ-COORDINATES + ENERGIES

O	-3.357845	0.172193	1.278638
O	-2.860800	0.273112	-2.356701
O	-5.618863	-0.482799	-2.110464
O	-6.850683	0.382328	0.294333
O	-5.168472	-1.225451	2.941317
O	-0.840964	2.631448	-0.245985
O	-1.532511	-1.995858	0.507732
O	2.842571	-0.385009	0.189300
O	1.590333	3.436059	-0.371762
O	9.055134	-1.140182	0.435709
C	-3.425818	-0.138880	-1.118056
C	-4.892580	0.239689	-1.120523
C	-2.747380	0.581868	0.051943
C	-5.512184	-0.096714	0.222807
C	-4.729805	0.568848	1.349640
C	-1.267648	0.336150	0.115809
C	-5.222735	0.183288	2.727157
C	-0.381997	1.383290	-0.051786
C	-0.735174	-0.966014	0.321486
C	1.028168	1.167427	-0.032554
C	0.656949	-1.196246	0.341818
C	1.508608	-0.121973	0.163123
C	1.977669	2.259394	-0.200147
C	3.744402	0.610633	0.046992
C	3.358007	1.900070	-0.137546
C	5.124000	0.135903	0.133153
C	6.181969	0.925833	-0.333731
C	5.406093	-1.113584	0.695275
C	7.487118	0.486512	-0.230876
C	6.712036	-1.557668	0.806389
C	7.752160	-0.753820	0.346643
H	-3.336811	-1.217993	-0.999792
H	-4.985891	1.315593	-1.301900

H	-2.914215	1.652952	-0.077976
H	-5.490604	-1.181159	0.357757
H	-4.802364	1.656682	1.240234
H	-6.260784	0.483748	2.841967
H	-4.618754	0.703487	3.472423
H	-2.044723	-0.223771	-2.490791
H	-5.303323	-0.204680	-2.976599
H	-7.334033	0.012653	-0.452489
H	1.037052	-2.195040	0.500362
H	-4.251046	-1.501778	2.846085
H	-0.049564	3.223017	-0.332451
H	4.092767	2.685218	-0.220552
H	5.989431	1.883084	-0.796570
H	4.604088	-1.737160	1.061814
H	8.307565	1.088538	-0.595412
H	6.933673	-2.519198	1.251224
H	9.116201	-2.001714	0.864984
H	-1.571238	-2.605147	-0.480080
O	-1.456730	-2.964666	-1.637779
O	-0.731225	-1.958381	-2.188089
H	0.191869	-2.263723	-2.184308

ENERGIES
SCF ENERGY: -1715.78865310
SUM OF ELECTRONIC AND ZERO-POINT ENERGIES: -1715.380226
SUM OF ELECTRONIC AND THERMAL ENERGIES: -1715.349483
SUM OF ELECTRONIC AND THERMAL ENTHALPIES: -1715.348538
SUM OF ELECTRONIC AND THERMAL FREE ENERGIES: -1715.444073

==> ISOVITEXIN/WATER/PA/C4' <==
50
XYZ-COORDINATES + ENERGIES

O	3.424343	-0.712494	-0.855342
O	2.748307	1.843070	1.641045
O	5.429676	0.941223	2.346861
O	6.903567	-0.164201	0.180679
O	5.139496	-2.984338	-0.971422
O	0.860241	2.375380	-0.967864
O	1.611285	-2.108049	0.493751
O	-2.772332	-0.535333	0.097461
O	-1.580181	3.130686	-1.188820
O	-8.932889	-1.420738	0.586432
C	3.359472	0.669576	1.119816
C	4.868709	0.841640	1.041157
C	2.783560	0.433064	-0.278170
C	5.502528	-0.346474	0.342035
C	4.839134	-0.560252	-1.017251
C	1.303420	0.168103	-0.238389
C	5.333648	-1.796550	-1.736282
C	0.401662	1.173807	-0.561475
C	0.787559	-1.073950	0.177737
C	-0.991242	0.965458	-0.460011
C	-0.575449	-1.309283	0.293707
C	-1.439774	-0.279492	-0.023652
C	-1.957119	1.994892	-0.788413
C	-3.704543	0.408082	-0.197934
C	-3.326045	1.648907	-0.631442
C	-5.056925	-0.062168	0.001292
C	-6.168811	0.755384	-0.285351
C	-5.309783	-1.358546	0.492240
C	-7.453993	0.308641	-0.093685
C	-6.592648	-1.814001	0.688663
C	-7.731140	-1.001468	0.405021
H	3.129583	-0.190775	1.754141
H	5.090629	1.750081	0.470163
H	2.989328	1.306239	-0.899706
H	5.374674	-1.237518	0.956305
H	5.030613	0.314811	-1.649492
H	6.401675	-1.703890	-1.914429
H	4.820539	-1.875240	-2.695495
H	2.833543	1.830358	2.599279
H	5.219294	1.809523	2.704821
H	7.046523	0.630794	-0.346659
H	-0.945192	-2.272727	0.611584
H	4.195980	-3.169471	-0.931779
H	0.055061	2.931380	-1.147566
H	2.502089	-1.918378	0.147416
H	-4.067358	2.396703	-0.862058
H	-6.025180	1.757171	-0.666586
H	-4.480979	-2.012733	0.723224
H	-8.293462	0.953373	-0.321847
H	-6.762790	-2.813569	1.068765

ENERGIES
SCF ENERGY: -1564.41371439
SUM OF ELECTRONIC AND ZERO-POINT ENERGIES: -1564.027904
SUM OF ELECTRONIC AND THERMAL ENERGIES: -1564.001058
SUM OF ELECTRONIC AND THERMAL ENTHALPIES: -1564.000114
SUM OF ELECTRONIC AND THERMAL FREE ENERGIES: -1564.086387

==> ISOVITEXIN/WATER/PA/C5 <==
50
XYZ-COORDINATES + ENERGIES

O	-3.466111	0.723602	-0.823763
O	-2.841876	-1.872420	1.651418
O	-5.507841	-0.933245	2.350267
O	-6.956988	0.210066	0.187531
O	-5.133198	3.016865	-0.934773
O	-0.973645	-2.400460	-1.014873
O	-1.624616	2.036800	0.581908
O	2.725713	0.469130	0.098477
O	1.698234	-3.138058	-1.462153
O	8.917027	1.352823	0.654600
C	-3.426793	-0.682670	1.131147
C	-4.938331	-0.830149	1.046978
C	-2.835044	-0.442624	-0.260114
C	-5.553519	0.372132	0.356413
C	-4.878776	0.589186	-0.996394
C	-1.350726	-0.215951	-0.231023
C	-5.353193	1.837995	-1.707420
C	-0.487438	-1.287955	-0.624947
C	-0.822081	0.990239	0.214527
C	0.945180	-1.054641	-0.543974
C	0.549447	1.210425	0.323724
C	1.390254	0.184767	-0.049417
C	1.942722	-2.023720	-0.956605
C	3.667371	-0.436865	-0.250109
C	3.327131	-1.630900	-0.768890
C	5.033161	0.044174	-0.006813
C	6.097152	-0.858526	0.092205
C	5.290913	1.410167	0.130572
C	7.386818	-0.410203	0.311185
C	6.581287	1.867990	0.347370
C	7.627414	0.955670	0.436884
H	-3.187019	0.165756	1.777622
H	-5.172576	-1.730276	0.467737
H	-3.057852	-1.303537	-0.891687
H	-5.417136	1.255918	0.979511
H	-5.079426	-0.277926	-1.637293
H	-6.423171	1.769159	-1.884604
H	-4.839587	1.913398	-2.666790
H	-2.933716	-1.862055	2.608954
H	-5.309690	-1.807121	2.701383
H	-7.107687	-0.573733	-0.354214
H	0.939217	2.153375	0.675964
H	-4.183818	3.161719	-0.869815
H	-2.520116	1.875510	0.236409
H	4.090305	-2.328420	-1.077939
H	5.918240	-1.921747	0.016137
H	4.482829	2.123385	0.057541
H	8.210663	-1.105070	0.396899
H	6.783970	2.926695	0.447724
H	8.955283	2.312910	0.735563

ENERGIES
SCF ENERGY: -1564.40830071
SUM OF ELECTRONIC AND ZERO-POINT ENERGIES: -1564.022200
SUM OF ELECTRONIC AND THERMAL ENERGIES: -1563.995089
SUM OF ELECTRONIC AND THERMAL ENTHALPIES: -1563.994145
SUM OF ELECTRONIC AND THERMAL FREE ENERGIES: -1564.080059

==> ISOVITEXIN/WATER/PA/C7 <==
50
XYZ-COORDINATES + ENERGIES

O	-3.460280	0.599566	-0.909614
O	-2.889924	-1.494947	2.018015

O	-5.629209	-0.590754	2.402205
O	-6.982434	0.116574	0.009741
O	-5.130264	2.756969	-1.495398
O	-0.943225	-2.288753	-0.958304
O	-1.620223	2.049313	0.862668
O	2.756282	0.525303	0.176563
O	1.488036	-3.030334	-1.349568
O	8.982606	1.205727	0.478022
C	-3.484485	-0.426132	1.280954
C	-4.978392	-0.666679	1.135689
C	-2.827341	-0.400751	-0.098887
C	-5.596133	0.367709	0.215349
C	-4.851472	0.368620	-1.119958
C	-1.350748	-0.134473	-0.049178
C	-5.320609	1.459654	-2.058277
C	-0.472611	-1.108139	-0.477131
C	-0.842310	1.118034	0.463473
C	0.937341	-0.927653	-0.428104
C	0.572394	1.291528	0.513426
C	1.405530	0.295176	0.083080
C	1.862565	-1.920886	-0.873294
C	3.649084	-0.406222	-0.233574
C	3.258262	-1.589459	-0.757364
C	5.037744	0.027512	-0.044399
C	6.078186	-0.907746	-0.018382
C	5.341680	1.381824	0.116982
C	7.388381	-0.502128	0.154689
C	6.652769	1.796727	0.287374
C	7.674462	0.852497	0.306409
H	-3.315561	0.521763	1.789803
H	-5.133594	-1.661314	0.701349
H	-3.002852	-1.374079	-0.562726
H	-5.526179	1.352868	0.676000
H	-4.997361	-0.604903	-1.605453
H	-6.383432	1.349344	-2.255989
H	-4.773195	1.372642	-2.998407
H	-2.976697	-1.302956	2.956403
H	-5.370941	-1.360748	2.919462
H	-7.076597	-0.766425	-0.366455
H	0.978921	2.215423	0.900181
H	-4.198796	2.838201	-1.262450
H	-0.146826	-2.823264	-1.212148
H	3.989229	-2.301910	-1.106302
H	5.865992	-1.963178	-0.113478
H	4.552280	2.118967	0.099239
H	8.194720	-1.221666	0.185196
H	6.890439	2.846195	0.405280
H	9.053759	2.161648	0.582588

\# ENERGIES
SCF ENERGY: -1564.41386259
SUM OF ELECTRONIC AND ZERO-POINT ENERGIES: -1564.029278
SUM OF ELECTRONIC AND THERMAL ENERGIES: -1564.001880
SUM OF ELECTRONIC AND THERMAL ENTHALPIES: -1564.000936
SUM OF ELECTRONIC AND THERMAL FREE ENERGIES: -1564.088403

==> ISOVITEXIN/WATER/PA/C7/AIP <==
50
XYZ-COORDINATES + ENERGIES

O	-3.417546	0.670808	-0.863327
O	-2.983723	-1.619707	1.927224
O	-5.734251	-0.704748	2.268644
O	-6.981456	0.176237	-0.130625
O	-5.026080	2.906495	-1.338259
O	-0.932106	-2.296088	-0.961699
O	-1.588853	1.937079	1.009651
O	2.765177	0.494946	0.241480
O	1.491069	-3.007835	-1.370249
O	8.978583	1.233268	0.424737
C	-3.547496	-0.506118	1.240812
C	-5.039485	-0.709309	1.024448
C	-2.852318	-0.408784	-0.116067
C	-5.604849	0.397307	0.153863
C	-4.805876	0.490127	-1.146461
C	-1.371854	-0.188713	-0.011472
C	-5.213956	1.662900	-2.011291
C	-0.491999	-1.136667	-0.449233
C	-0.852637	1.036664	0.561873
C	0.941907	-0.933181	-0.381397
C	0.584598	1.233395	0.623090
C	1.431529	0.248575	0.150668
C	1.886436	-1.930449	-0.874201
C	3.660141	-0.407634	-0.207485
C	3.264549	-1.586041	-0.761567
C	5.041668	0.030962	-0.035996
C	6.094320	-0.890832	-0.098747
C	5.331071	1.380222	0.194791
C	7.401757	-0.475952	0.057889
C	6.639489	1.803551	0.345485
C	7.674130	0.873344	0.277386
H	-3.385273	0.410890	1.808081
H	-5.193810	-1.669254	0.518688
H	-3.035714	-1.341550	-0.654409
H	-5.547820	1.344666	0.689383
H	-4.947070	-0.437760	-1.714803
H	-6.269221	1.589348	-2.259892
H	-4.629198	1.639750	-2.932390
H	-3.220306	-1.554327	2.858143
H	-5.591853	-1.553228	2.700308
H	-7.063426	-0.672522	-0.581465
H	0.969305	2.151636	1.041105
H	-4.098740	2.962706	-1.083315
H	-0.137431	-2.826182	-1.231688
H	3.996562	-2.280359	-1.142825
H	5.895587	-1.942158	-0.248853
H	4.532374	2.105659	0.244088
H	8.218836	-1.182666	0.020995
H	6.867053	2.848064	0.514153
H	9.046877	2.183439	0.575772

\# ENERGIES
SCF ENERGY: -1564.21422566
SUM OF ELECTRONIC AND ZERO-POINT ENERGIES: -1563.829595
SUM OF ELECTRONIC AND THERMAL ENERGIES: -1563.802124
SUM OF ELECTRONIC AND THERMAL ENTHALPIES: -1563.801180
SUM OF ELECTRONIC AND THERMAL FREE ENERGIES: -1563.889525

==> ISOVITEXIN/WATER/PA/C7/BDE/C4'/TS <==
53
XYZ-COORDINATES + ENERGIES

O	4.104733	-0.487365	-0.945291
O	3.458854	1.049401	2.329857
O	6.127544	0.027670	2.620181
O	7.545331	-0.183616	0.184810
O	5.863422	-2.496134	-1.848270
O	1.668514	2.457434	-0.553118
O	2.118461	-2.166638	0.426334
O	-2.166536	-0.383491	-0.199655
O	-0.719111	3.360325	-0.890997
O	-8.356085	-0.935287	-0.639380
C	4.020757	0.122887	1.403153
C	5.514778	0.366297	1.377190
C	3.447526	0.366609	0.003016
C	6.161388	-0.493177	0.309565
C	5.504333	-0.226357	-1.039456
C	1.962943	0.155321	-0.064857
C	6.033999	-1.111680	-2.146361
C	1.138620	1.228080	-0.334188
C	1.390249	-1.152136	0.173831
C	-0.277550	1.095083	-0.391204
C	-0.030416	-1.278919	0.113270
C	-0.807933	-0.188783	-0.160331
C	-1.143334	2.192340	-0.672082
C	-3.004874	0.644745	-0.466979
C	-2.556641	1.896357	-0.703324
C	-4.411168	0.231156	-0.489826
C	-5.433555	1.185345	-0.355009
C	-4.738480	-1.125509	-0.647692
C	-6.752505	0.797872	-0.391793
C	-6.056376	-1.522253	-0.685235
C	-7.085169	-0.564189	-0.565594
H	3.821382	-0.900475	1.720493
H	5.700776	1.420891	1.148492
H	3.673489	1.401810	-0.261208
H	6.031820	-1.545835	0.574115
H	5.667686	0.821783	-1.315473

H	7.099186	-0.942253	-2.279314
H	5.516217	-0.857636	-3.072954
H	2.521533	0.846299	2.425371
H	5.819510	0.649056	3.287842
H	7.946299	-0.287896	1.054584
H	-0.483545	-2.245121	0.284682
H	4.926622	-2.644505	-1.680237
H	0.906502	3.065963	-0.730308
H	-3.242188	2.693976	-0.942817
H	-5.191383	2.226575	-0.200868
H	-3.953159	-1.859337	-0.748957
H	-7.552386	1.517450	-0.283301
H	-6.327458	-2.561069	-0.812396
H	-8.742042	-1.082543	0.403509
O	-8.834176	-1.183450	1.661051
O	-7.551030	-0.983625	2.053003
H	-7.477795	-0.033458	2.243987

ENERGIES
SCF ENERGY: -1715.33094884
SUM OF ELECTRONIC AND ZERO-POINT ENERGIES: -1714.934991
SUM OF ELECTRONIC AND THERMAL ENERGIES: -1714.904662
SUM OF ELECTRONIC AND THERMAL ENTHALPIES: -1714.903718
SUM OF ELECTRONIC AND THERMAL FREE ENERGIES: -1714.997993

==> ISOVITEXIN/WATER/PA/C7/BDE/C5/TS <==
53
XYZ-COORDINATES + ENERGIES

O	3.388748	-0.611432	-0.803114
O	2.760650	0.932854	2.475259
O	5.433630	-0.077605	2.738046
O	6.837750	-0.260802	0.285436
O	5.129970	-2.611668	-1.720635
O	0.935733	2.349763	-0.087054
O	1.472236	-2.340211	0.392724
O	-2.846821	-0.588457	0.161347
O	-1.670804	3.299390	-0.174831
O	-9.069825	-1.457895	0.166613
C	3.314403	0.009548	1.542555
C	4.810710	0.261661	1.501006
C	2.737260	0.247385	0.135378
C	5.459367	-0.590240	0.424936
C	4.784875	-0.335116	-0.919387
C	1.253413	0.019661	0.096450
C	5.300247	-1.229033	-2.029630
C	0.390903	1.140295	0.014943
C	0.710718	-1.326084	0.245904
C	-1.027797	0.996032	0.023211
C	-0.697993	-1.441392	0.234248
C	-1.506689	-0.323491	0.133152
C	-1.976937	2.089650	-0.074009
C	-3.760641	0.412246	0.061832
C	-3.375064	1.701200	-0.059233
C	-5.141412	-0.088360	0.094493
C	-6.211548	0.776691	0.366882
C	-5.411586	-1.441072	-0.148992
C	-7.515525	0.307390	0.385959
C	-6.715542	-1.919471	-0.133283
C	-7.766623	-1.042597	0.133401
H	3.120648	-1.017253	1.858774
H	4.986890	1.319878	1.275682
H	2.951832	1.283596	-0.136099
H	5.348370	-1.645733	0.691968
H	4.933287	0.713255	-1.207630
H	6.365570	-1.065174	-2.177110
H	4.771538	-0.979325	-2.952878
H	1.861582	0.648932	2.698905
H	5.152168	0.563583	3.407186
H	7.258813	-0.380974	1.150373
H	-1.152694	-2.418492	0.335567
H	4.189217	-2.759410	-1.538412
H	-4.110329	2.485452	-0.166447
H	-6.031090	1.821406	0.584277
H	-4.602465	-2.126545	-0.361883
H	-8.342376	0.971344	0.603762
H	-6.922516	-2.965437	-0.327722
H	-9.123510	-2.409537	-0.014544
H	1.263826	2.489003	-1.140726
O	1.518672	2.309472	-2.409501
O	1.331039	0.964664	-2.525446
H	0.386195	0.857453	-2.752706

ENERGIES
SCF ENERGY: -1714.85456512
SUM OF ELECTRONIC AND ZERO-POINT ENERGIES: -1714.457885
SUM OF ELECTRONIC AND THERMAL ENERGIES: -1714.427212
SUM OF ELECTRONIC AND THERMAL ENTHALPIES: -1714.426268
SUM OF ELECTRONIC AND THERMAL FREE ENERGIES: -1714.521801

==> ISOVITEXIN/WATER/PA/C7/PA/C4'/AIP <==
49
XYZ-COORDINATES + ENERGIES

O	-3.390674	0.563417	-0.935177
O	-2.893683	-1.444265	2.063935
O	-5.638627	-0.515097	2.362783
O	-6.934672	0.134654	-0.076431
O	-5.024989	2.717881	-1.614843
O	-0.885677	-2.335062	-0.840608
O	-1.586336	2.055075	0.824902
O	2.797534	0.544854	0.194847
O	1.557350	-3.081255	-1.187178
O	8.922771	1.333459	0.389731
C	-3.468855	-0.395799	1.284423
C	-4.961147	-0.631800	1.113474
C	-2.783414	-0.415952	-0.081448
C	-5.551145	0.380773	0.151241
C	-4.779306	0.338363	-1.167864
C	-1.306848	-0.153744	-0.013097
C	-5.218613	1.407358	-2.145082
C	-0.424308	-1.137430	-0.399251
C	-0.804715	1.117859	0.462730
C	0.987732	-0.947961	-0.349044
C	0.611213	1.300985	0.513578
C	1.450040	0.298606	0.119205
C	1.911942	-1.950780	-0.753129
C	3.699309	-0.395180	-0.178738
C	3.312683	-1.608667	-0.642268
C	5.074734	0.050572	-0.030357
C	6.157612	-0.831552	-0.299082
C	5.342606	1.379081	0.389327
C	7.442980	-0.412864	-0.160562
C	6.624390	1.817325	0.532141
C	7.744735	0.940023	0.262296
H	-3.305935	0.566378	1.768149
H	-5.114206	-1.637289	0.704378
H	-2.954601	-1.402203	-0.518946
H	-5.482663	1.377986	0.585390
H	-4.924015	-0.646955	-1.629245
H	-6.278619	1.302340	-2.360018
H	-4.654469	1.288488	-3.071731
H	-3.048320	-1.251554	2.993726
H	-5.425830	-1.288219	2.895320
H	-7.028620	-0.758464	-0.427842
H	1.009480	2.241913	0.866035
H	-4.096860	2.796571	-1.368051
H	-0.089933	-2.878446	-1.070569
H	4.035410	-2.348186	-0.945845
H	5.967157	-1.847620	-0.609580
H	4.519672	2.046511	0.592908
H	8.275343	-1.073703	-0.358478
H	6.843290	2.827894	0.847753

ENERGIES
SCF ENERGY: -1563.76287736
SUM OF ELECTRONIC AND ZERO-POINT ENERGIES: -1563.390966
SUM OF ELECTRONIC AND THERMAL ENERGIES: -1563.363825
SUM OF ELECTRONIC AND THERMAL ENTHALPIES: -1563.362881
SUM OF ELECTRONIC AND THERMAL FREE ENERGIES: -1563.450157

==> ISOVITEXIN/WATER/PA/C7/PA/C4'/PA/C5 <==
48
XYZ-COORDINATES + ENERGIES

O	-3.404220	0.504164	-0.943382
O	-2.942678	-1.209565	2.248592
O	-5.683169	-0.254180	2.409772
O	-6.954305	0.106218	-0.100720

O	-5.087825	2.544096	-1.881133
O	-0.973996	-2.399312	-0.723431
O	-1.540743	2.070998	0.774209
O	2.791369	0.465460	0.180020
O	1.679648	-3.188554	-1.223278
O	8.984931	1.425353	0.368249
C	-3.496459	-0.246609	1.347467
C	-4.987335	-0.501502	1.187800
C	-2.791256	-0.406242	-0.000225
C	-5.576153	0.395821	0.117615
C	-4.781662	0.231550	-1.177568
C	-1.310178	-0.185050	0.029706
C	-5.229983	1.177936	-2.270407
C	-0.473731	-1.267640	-0.363809
C	-0.786923	1.074733	0.439693
C	0.970702	-1.066837	-0.345417
C	0.629669	1.227174	0.474842
C	1.440952	0.191573	0.095240
C	1.941493	-2.043507	-0.768375
C	3.719501	-0.448280	-0.197939
C	3.334069	-1.654403	-0.669113
C	5.087087	0.034724	-0.038468
C	6.184226	-0.838918	-0.122029
C	5.353846	1.392514	0.198865
C	7.478315	-0.383047	0.013172
C	6.646133	1.858871	0.333639
C	7.771422	0.992361	0.244273
H	-3.336775	0.758650	1.733159
H	-5.132952	-1.546359	0.889011
H	-2.979562	-1.422598	-0.351051
H	-5.528919	1.433414	0.447669
H	-4.901516	-0.799610	-1.535029
H	-6.281593	1.018196	-2.493335
H	-4.642256	0.979775	-3.168454
H	-3.069079	-0.891558	3.147347
H	-5.444718	-0.945404	3.035604
H	-7.030281	-0.820116	-0.357925
H	1.060932	2.165661	0.795103
H	-4.166080	2.680472	-1.636822
H	4.070483	-2.369839	-1.000974
H	6.020979	-1.896767	-0.281560
H	4.533075	2.093050	0.269134
H	8.307018	-1.077865	-0.047376
H	6.826730	2.912134	0.510528

ENERGIES
SCF ENERGY: -1563.46374603
SUM OF ELECTRONIC AND ZERO-POINT ENERGIES: -1563.105014
SUM OF ELECTRONIC AND THERMAL ENERGIES: -1563.077952
SUM OF ELECTRONIC AND THERMAL ENTHALPIES: -1563.077008
SUM OF ELECTRONIC AND THERMAL FREE ENERGIES: -1563.163892

==> ISOVITEXIN/WATER/PA/C7/PA/C4'/RAF/C1' <==
52
XYZ-COORDINATES + ENERGIES

O	3.753190	-0.907842	-0.746063
O	3.382502	2.075260	1.367707
O	6.122430	1.325457	1.797501
O	7.273240	-0.091127	-0.358878
O	5.478447	-3.113117	-0.711577
O	1.215452	1.884904	-1.552199
O	2.021971	-1.672297	1.492085
O	-2.392517	-0.352192	0.652435
O	-1.236597	2.546781	-1.962472
O	-8.342378	-1.264972	-1.314147
C	3.892085	0.804567	0.969269
C	5.357454	0.993448	0.640066
C	3.156519	0.311885	-0.282078
C	5.932590	-0.290459	0.077456
C	5.117520	-0.742032	-1.128284
C	1.687238	0.109159	-0.050344
C	5.570173	-2.074597	-1.685223
C	0.780845	0.925506	-0.696184
C	1.216804	-0.902435	0.870512
C	-0.622250	0.804442	-0.492414
C	-0.190938	-1.020252	1.068565
C	-1.052042	-0.190318	0.405319
C	-1.573089	1.640583	-1.151150
C	-3.304208	0.430627	0.033747
C	-2.962497	1.395230	-0.838720
C	-4.716505	0.039208	0.401507
C	-5.749109	0.969391	-0.110503
C	-4.981727	-1.379914	0.055379
C	-6.922613	0.527950	-0.626911
C	-6.169232	-1.782117	-0.474273
C	-7.215121	-0.866537	-0.820909
H	3.787885	0.082064	1.778637
H	5.455609	1.787158	-0.108001
H	3.294574	1.070002	-1.055863
H	5.897226	-1.063493	0.849542
H	5.193204	0.017226	-1.915257
H	6.610732	-2.014465	-1.992860
H	4.956115	-2.316607	-2.554403
H	2.479332	1.951742	1.680810
H	5.856222	2.201803	2.093637
H	7.767147	0.280850	0.380040
H	-0.569284	-1.768190	1.751027
H	4.568543	-3.134065	-0.395489
H	0.404883	2.342439	-1.894839
H	-3.721193	1.987708	-1.325609
H	-5.565538	2.028366	0.017065
H	-4.202824	-2.096937	0.278463
H	-7.680049	1.241732	-0.930333
H	-6.338982	-2.833997	-0.674641
O	-4.634646	0.178970	1.923494
O	-5.872495	-0.210594	2.487761
H	-5.853784	-1.177197	2.423948

ENERGIES
SCF ENERGY: -1714.87476423
SUM OF ELECTRONIC AND ZERO-POINT ENERGIES: -1714.486263
SUM OF ELECTRONIC AND THERMAL ENERGIES: -1714.456175
SUM OF ELECTRONIC AND THERMAL ENTHALPIES: -1714.455231
SUM OF ELECTRONIC AND THERMAL FREE ENERGIES: -1714.548314

==> ISOVITEXIN/WATER/PA/C7/PA/C4'/RAF/C2 <==
52
XYZ-COORDINATES + ENERGIES

O	-3.707903	0.989881	-0.689968
O	-3.170018	-2.063522	1.283116
O	-5.899718	-1.407271	1.866115
O	-7.187240	0.076711	-0.165568
O	-5.486851	3.149565	-0.484186
O	-1.114764	-1.683498	-1.726179
O	-1.886349	1.702450	1.496913
O	2.528123	0.607411	0.387072
O	1.277744	-2.231559	-2.314210
O	8.596267	1.848165	0.019358
C	-3.726529	-0.791769	0.958356
C	-5.200859	-1.004265	0.690013
C	-3.061991	-0.232417	-0.304459
C	-5.830842	0.288160	0.212224
C	-5.085815	0.806639	-1.012416
C	-1.590691	-0.000733	-0.128280
C	-5.595945	2.147791	-1.493697
C	-0.687043	-0.758904	-0.840690
C	-1.103411	0.982719	0.811935
C	0.726881	-0.613451	-0.676067
C	0.321228	1.136710	0.958364
C	1.180026	0.364426	0.245436
C	1.647320	-1.404031	-1.414518
C	3.433978	-0.442285	0.096955
C	3.025092	-1.250299	-1.083700
C	4.817260	0.127374	0.043907
C	5.575504	0.170630	-1.127311
C	5.361602	0.687915	1.206944
C	6.835343	0.745436	-1.141886
C	6.617887	1.260423	1.204303
C	7.414129	1.313348	0.024653
H	-3.601315	-0.097823	1.789739
H	-5.315502	-1.766788	-0.087650
H	-3.214518	-0.963460	-1.101473
H	-5.773005	1.029707	1.013232
H	-5.182948	0.076947	-1.824588
H	-6.647609	2.071309	-1.756780
H	-5.028765	2.440063	-2.379098

H	-2.257557	-1.930533	1.564000
H	-5.582221	-2.280159	2.119390
H	-7.637545	-0.328161	0.583806
H	0.694001	1.880388	1.648704
H	-4.562222	3.193542	-0.217417
H	-0.287543	-2.076054	-2.139236
H	3.756451	-1.856535	-1.594061
H	5.181509	-0.239356	-2.048617
H	4.786207	0.669568	2.124793
H	7.409015	0.769081	-2.060265
H	7.024887	1.684380	2.114213
O	3.272449	-1.274083	1.299581
O	4.131333	-2.392134	1.195337
H	4.975360	-2.053550	1.531860

ENERGIES
SCF ENERGY: -1714.88577955
SUM OF ELECTRONIC AND ZERO-POINT ENERGIES: -1714.496715
SUM OF ELECTRONIC AND THERMAL ENERGIES: -1714.466999
SUM OF ELECTRONIC AND THERMAL ENTHALPIES: -1714.466055
SUM OF ELECTRONIC AND THERMAL FREE ENERGIES: -1714.558425

==> ISOVITEXIN/WATER/PA/C7/PA/C4'/RAF/C2/TS <==
52
XYZ-COORDINATES + ENERGIES

O	3.733266	0.891142	0.764263
O	3.185337	-1.918707	-1.540280
O	5.918778	-1.196911	-2.052772
O	7.208943	0.034481	0.138866
O	5.529208	3.053804	0.817925
O	1.166646	-1.865758	1.539325
O	1.901188	1.828592	-1.345173
O	-2.493885	0.656396	-0.254818
O	-1.263225	-2.472067	2.082482
O	-8.654671	1.712311	-0.162909
C	3.745180	-0.692754	-1.075484
C	5.220322	-0.935681	-0.836483
C	3.085065	-0.279705	0.244688
C	5.853183	0.291468	-0.211625
C	5.109733	0.666418	1.064526
C	1.611885	-0.028240	0.109649
C	5.625414	1.938158	1.702089
C	0.717675	-0.848810	0.765732
C	1.118068	1.049130	-0.717524
C	-0.692570	-0.670287	0.660485
C	-0.300715	1.227413	-0.812761
C	-1.147607	0.393870	-0.146973
C	-1.619788	-1.520692	1.325422
C	-3.416669	-0.231422	0.208113
C	-3.009673	-1.254796	1.087092
C	-4.777579	0.278934	0.142555
C	-5.787648	-0.182403	1.002435
C	-5.125311	1.226443	-0.838205
C	-7.078571	0.289960	0.904138
C	-6.412757	1.699459	-0.947207
C	-7.452565	1.257574	-0.073806
H	3.620180	0.090534	-1.822784
H	5.336763	-1.784444	-0.154450
H	3.243608	-1.095510	0.953243
H	5.796797	1.120667	-0.921718
H	5.202345	-0.153801	1.785778
H	6.675039	1.824008	1.959542
H	5.055108	2.127998	2.613037
H	2.267931	-1.755726	-1.786794
H	5.625285	-2.048505	-2.392027
H	7.655660	-0.291921	-0.649857
H	-0.688999	2.034861	-1.417411
H	4.607182	3.135199	0.550907
H	0.361856	-2.308935	1.918814
H	-3.743423	-1.927658	1.500439
H	-5.558044	-0.905405	1.773645
H	-4.368159	1.583869	-1.523563
H	-7.840675	-0.066986	1.585432
H	-6.663400	2.427794	-1.708328
O	-3.330770	-1.432780	-1.307610
O	-4.524851	-2.118106	-1.428407
H	-5.183188	-1.429437	-1.612173

ENERGIES
SCF ENERGY: -1714.86813353
SUM OF ELECTRONIC AND ZERO-POINT ENERGIES: -1714.481837
SUM OF ELECTRONIC AND THERMAL ENERGIES: -1714.451429
SUM OF ELECTRONIC AND THERMAL ENTHALPIES: -1714.450485
SUM OF ELECTRONIC AND THERMAL FREE ENERGIES: -1714.545521

==> ISOVITEXIN/WATER/PA/C7/PA/C4'/RAF/C2' <==
52
XYZ-COORDINATES + ENERGIES

O	3.795494	-0.208203	-1.119870
O	3.225754	0.667809	2.403698
O	5.889608	-0.402615	2.441148
O	7.259526	-0.171146	-0.018630
O	5.475035	-2.027721	-2.429417
O	1.362209	2.597274	-0.079941
O	1.847055	-2.131161	-0.061597
O	-2.453176	-0.282197	-0.186333
O	-1.021669	3.532193	-0.126870
O	-8.704670	-0.814697	-0.646386
C	3.763137	-0.065881	1.305272
C	5.258263	0.168503	1.296342
C	3.162353	0.444346	-0.008834
C	5.872879	-0.480212	0.072628
C	5.197029	0.048356	-1.187098
C	1.676421	0.243129	-0.078250
C	5.691350	-0.617782	-2.452858
C	0.842438	1.343353	-0.098553
C	1.110967	-1.088309	-0.085907
C	-0.572876	1.214333	-0.133148
C	-0.309595	-1.209181	-0.125503
C	-1.097306	-0.090237	-0.147351
C	-1.451958	2.340565	-0.142338
C	-3.313888	0.770142	-0.191598
C	-2.856550	2.054278	-0.164241
C	-4.696001	0.361497	-0.239028
C	-5.727467	1.277279	-0.394149
C	-5.025654	-1.089311	-0.054066
C	-7.045273	0.869669	-0.473341
C	-6.439351	-1.459661	-0.310480
C	-7.444054	-0.534332	-0.468379
H	3.560679	-1.130207	1.423523
H	5.452448	1.245794	1.266243
H	3.384724	1.511659	-0.072453
H	5.730118	-1.562196	0.134657
H	5.374660	1.127464	-1.261014
H	6.760749	-0.459369	-2.563401
H	5.176705	-0.171059	-3.305354
H	2.288565	0.453762	2.474892
H	5.594048	0.079984	3.220009
H	7.671593	-0.435445	0.811155
H	-0.757085	-2.193122	-0.137033
H	4.535479	-2.174445	-2.274345
H	0.585324	3.218687	-0.096790
H	-3.544489	2.884394	-0.154526
H	-5.508254	2.332254	-0.481700
H	-4.339695	-1.720533	-0.619817
H	-7.827896	1.607592	-0.588527
H	-6.679663	-2.515270	-0.340495
O	-4.657986	-1.319263	1.367546
O	-4.692639	-2.717801	1.610622
H	-5.623519	-2.882978	1.818505

ENERGIES
SCF ENERGY: -1714.87376750
SUM OF ELECTRONIC AND ZERO-POINT ENERGIES: -1714.485488
SUM OF ELECTRONIC AND THERMAL ENERGIES: -1714.455204
SUM OF ELECTRONIC AND THERMAL ENTHALPIES: -1714.454260
SUM OF ELECTRONIC AND THERMAL FREE ENERGIES: -1714.548704

==> ISOVITEXIN/WATER/PA/C7/PA/C4'/RAF/C3 <==
52
XYZ-COORDINATES + ENERGIES

O	3.595249	-1.019554	-0.745819
O	3.244583	1.956913	1.380276
O	5.994096	1.218025	1.767045
O	7.118157	-0.192094	-0.406231
O	5.326167	-3.218406	-0.738786

O	1.017477	1.756956	-1.480404
O	1.910548	-1.799824	1.520948
O	-2.531749	-0.595115	0.726286
O	-1.453828	2.445621	-1.780864
O	-8.588538	-2.167189	-0.030229
C	3.753876	0.689307	0.971743
C	5.213025	0.884962	0.620686
C	2.999378	0.195434	-0.267830
C	5.784163	-0.396152	0.047920
C	4.953404	-0.847996	-1.146895
C	1.537169	-0.019005	-0.008272
C	5.401966	-2.178438	-1.712309
C	0.607697	0.787392	-0.627458
C	1.091616	-1.036804	0.918829
C	-0.796497	0.661434	-0.385638
C	-0.316071	-1.181281	1.132985
C	-1.208466	-0.367095	0.497478
C	-1.735866	1.556701	-0.943799
C	-3.498290	0.121181	0.044794
C	-3.156451	1.469155	-0.428120
C	-4.787671	-0.450851	0.028058
C	-5.919372	0.261900	-0.452515
C	-5.015264	-1.773209	0.495518
C	-7.170214	-0.306267	-0.474614
C	-6.268433	-2.337491	0.475217
C	-7.410036	-1.634889	-0.012065
H	3.665440	-0.035047	1.781509
H	5.296919	1.680069	-0.127644
H	3.118209	0.956598	-1.041885
H	5.761723	-1.170542	0.819144
H	5.015293	-0.087047	-1.933429
H	6.437995	-2.115477	-2.034279
H	4.776445	-2.420858	-2.573165
H	2.343958	1.829040	1.699093
H	5.727056	2.091607	2.070507
H	7.620956	0.179048	0.327142
H	-0.666771	-1.964139	1.790967
H	4.421582	-3.239367	-0.407643
H	0.207838	2.219658	-1.805748
H	-5.807823	1.280786	-0.798657
H	-4.181080	-2.350449	0.869145
H	-8.015185	0.262623	-0.843214
H	-6.412078	-3.349160	0.835332
H	-3.840090	1.815511	-1.198680
O	-3.292113	2.373410	0.721238
O	-3.275054	3.722168	0.262365
H	-2.348314	3.981935	0.362627

ENERGIES
SCF ENERGY: -1714.88889362
SUM OF ELECTRONIC AND ZERO-POINT ENERGIES: -1714.498849
SUM OF ELECTRONIC AND THERMAL ENERGIES: -1714.468816
SUM OF ELECTRONIC AND THERMAL ENTHALPIES: -1714.467872
SUM OF ELECTRONIC AND THERMAL FREE ENERGIES: -1714.561533

==> ISOVITEXIN/WATER/PA/C7/PA/C4'/RAF/C3/TS <==
52
XYZ-COORDINATES + ENERGIES

O	-3.677914	-0.852560	0.855147
O	-3.125208	1.678706	-1.751435
O	-5.873873	0.945981	-2.150785
O	-7.151533	-0.026172	0.174095
O	-5.492922	-2.976428	1.140818
O	-1.046929	1.941751	1.241837
O	-1.921544	-2.059158	-1.153710
O	2.519465	-0.815815	-0.380456
O	1.429985	2.581881	1.593427
O	8.647162	-2.016522	-0.278798
C	-3.695752	0.519357	-1.148568
C	-5.163811	0.808247	-0.920912
C	-3.021311	0.243089	0.199550
C	-5.802902	-0.336105	-0.160887
C	-5.049197	-0.582097	1.140923
C	-1.555941	-0.049395	0.062210
C	-5.569343	-1.777060	1.910107
C	-0.631129	0.830084	0.585903
C	-1.103964	-1.224252	-0.649049
C	0.772006	0.619443	0.455756
C	0.305555	-1.434575	-0.767440
C	1.186309	-0.540258	-0.230104
C	1.733457	1.529507	0.966336
C	3.480725	-0.008023	0.147992
C	3.134108	1.234087	0.686771
C	4.803312	-0.513830	0.029646
C	5.930442	0.273733	0.379595
C	5.043276	-1.828308	-0.445751
C	7.202948	-0.218282	0.275036
C	6.313278	-2.327664	-0.551078
C	7.462079	-1.549812	-0.190518
H	-3.589702	-0.342744	-1.806917
H	-5.260877	1.727261	-0.333561
H	-3.154430	1.134662	0.816197
H	-5.764193	-1.237167	-0.778565
H	-5.127496	0.309870	1.773110
H	-6.614903	-1.627497	2.165989
H	-4.989766	-1.879990	2.829098
H	-2.209387	1.478986	-1.976760
H	-5.561526	1.742933	-2.591256
H	-7.602209	0.224052	-0.639840
H	0.665266	-2.313161	-1.284324
H	-4.578277	-3.082206	0.856843
H	-0.232772	2.426834	1.526665
H	5.793990	1.290553	0.722309
H	4.204015	-2.451173	-0.721165
H	8.052019	0.398424	0.540916
H	6.478071	-3.336202	-0.908613
H	3.857764	1.747535	1.299562
O	3.277696	2.541760	-0.737710
O	3.464135	3.783179	-0.107119
H	2.601391	3.968889	0.294725

ENERGIES
SCF ENERGY: -1714.87613013
SUM OF ELECTRONIC AND ZERO-POINT ENERGIES: -1714.488329
SUM OF ELECTRONIC AND THERMAL ENERGIES: -1714.458201
SUM OF ELECTRONIC AND THERMAL ENTHALPIES: -1714.457257
SUM OF ELECTRONIC AND THERMAL FREE ENERGIES: -1714.551058

==> ISOVITEXIN/WATER/PA/C7/PA/C4'/RAF/C3'/TS <==
52
XYZ-COORDINATES + ENERGIES

O	3.968464	-0.476742	-1.017595
O	3.439748	0.985742	2.312206
O	6.090744	-0.104965	2.511943
O	7.441237	-0.301010	0.040909
O	5.627087	-2.520246	-2.000299
O	1.616272	2.529353	-0.402493
O	1.975545	-2.155370	0.311508
O	-2.277670	-0.228691	-0.089214
O	-0.746769	3.509886	-0.597948
O	-8.512489	-0.526910	-0.463153
C	3.956757	0.070057	1.349272
C	5.455339	0.276584	1.292956
C	3.356954	0.365827	-0.029674
C	6.052246	-0.574914	0.190664
C	5.372952	-0.257799	-1.136516
C	1.866157	0.193000	-0.062378
C	5.847595	-1.137433	-2.272749
C	1.061485	1.300984	-0.240423
C	1.266733	-1.109389	0.126949
C	-0.356521	1.206822	-0.257516
C	-0.156732	-1.197042	0.098279
C	-0.914033	-0.072024	-0.086630
C	-1.205793	2.341855	-0.438478
C	-3.102840	0.832588	-0.267536
C	-2.620046	2.086287	-0.443161
C	-4.514778	0.453023	-0.264910
C	-5.518074	1.447704	-0.085644
C	-4.891175	-0.857448	-0.489540
C	-6.848585	1.135055	-0.152598
C	-6.255837	-1.232966	-0.458190
C	-7.282691	-0.201947	-0.392765
H	3.737738	-0.956380	1.644283
H	5.662678	1.331042	1.082788
H	3.601936	1.402305	-0.271671
H	5.898974	-1.629550	0.434033

H 5.564821 0.790206 -1.394132
H 6.916082 -1.007199 -2.422524
H 5.323016 -0.843374 -3.183533
H 2.494074 0.821573 2.402326
H 5.834611 0.522100 3.195945
H 7.854278 -0.416811 0.903696
H -0.631055 -2.159064 0.232390
H 4.690779 -2.631584 -1.802504
H 0.859237 3.163485 -0.511913
H -3.289374 2.913946 -0.616424
H -5.227633 2.469541 0.114383
H -4.147241 -1.618856 -0.672522
H -7.603882 1.899760 -0.027556
H -6.544843 -2.174246 -0.905932
O -6.572226 -1.900768 1.301777
O -7.800253 -2.603641 1.227690
H -8.406414 -1.942934 0.839761

ENERGIES
SCF ENERGY: -1714.87697870
SUM OF ELECTRONIC AND ZERO-POINT ENERGIES: -1714.488935
SUM OF ELECTRONIC AND THERMAL ENERGIES: -1714.459153
SUM OF ELECTRONIC AND THERMAL ENTHALPIES: -1714.458209
SUM OF ELECTRONIC AND THERMAL FREE ENERGIES: -1714.551268

==> ISOVITEXIN/WATER/PA/C7/PA/C4'/RAF/C4 <==
52
XYZ-COORDINATES + ENERGIES
O 3.690522 0.130276 -0.968584
O 2.743169 -0.777321 2.463913
O 5.494804 -1.603314 2.317266
O 7.027719 -0.140117 0.446479
O 5.721535 -0.727963 -2.742658
O 0.930365 2.094289 0.912552
O 1.791934 -2.165716 -1.099718
O -2.630930 -0.561699 -0.764581
O -1.381277 2.744013 1.339677
O -8.569212 -2.077251 0.144920
C 3.442413 -0.876099 1.223663
C 4.900964 -0.588802 1.507278
C 2.901257 0.161530 0.235187
C 5.681152 -0.538943 0.209219
C 5.052921 0.480681 -0.733459
C 1.448882 -0.032505 -0.081939
C 5.729943 0.538293 -2.085534
C 0.536732 0.957115 0.286987
C 0.981713 -1.228946 -0.727569
C -0.838829 0.816701 0.032659
C -0.418498 -1.367890 -0.935661
C -1.275893 -0.359741 -0.552396
C -1.789278 1.929766 0.337162
C -3.528423 0.204417 -0.056554
C -3.169847 1.373339 0.515905
C -4.849587 -0.385234 -0.001095
C -5.954850 0.340803 0.529446
C -5.062711 -1.700994 -0.491463
C -7.193224 -0.214228 0.583586
C -6.297554 -2.273491 -0.444728
C -7.431859 -1.557746 0.098498
H 3.339746 -1.879093 0.810042
H 4.983307 0.380061 2.010845
H 3.032549 1.143909 0.695176
H 5.657142 -1.526649 -0.258397
H 5.105374 1.473615 -0.271786
H 6.770555 0.828225 -1.966246
H 5.221744 1.285376 -2.697703
H 1.809457 -0.943069 2.287062
H 5.087495 -1.569718 3.188813
H 7.396000 -0.737710 1.106427
H -0.802705 -2.275171 -1.381646
H 4.803536 -1.005678 -2.829700
H -0.002124 2.542384 1.199010
H -3.868218 1.938506 1.112850
H -5.816129 1.351569 0.882695
H -4.228500 -2.252973 -0.896223
H -8.037423 0.332270 0.981189
H -6.466204 -3.278028 -0.807945
O -1.796252 2.674939 -0.973365
O -2.728895 3.753312 -0.896732
H -3.526012 3.376434 -1.292975

ENERGIES
SCF ENERGY: -1714.87409861
SUM OF ELECTRONIC AND ZERO-POINT ENERGIES: -1714.486906
SUM OF ELECTRONIC AND THERMAL ENERGIES: -1714.457159
SUM OF ELECTRONIC AND THERMAL ENTHALPIES: -1714.456215
SUM OF ELECTRONIC AND THERMAL FREE ENERGIES: -1714.548707

==> ISOVITEXIN/WATER/PA/C7/PA/C4'/RAF/C4a <==
52
XYZ-COORDINATES + ENERGIES
O -3.537597 0.971112 -0.862438
O -2.785690 -1.897017 1.311813
O -5.489882 -1.254372 2.024015
O -6.947419 0.020470 -0.036153
O -5.381201 3.088530 -0.735238
O -0.938335 -1.716492 -1.817927
O -1.633502 1.928252 1.152365
O 2.747002 0.634962 0.097640
O 1.478375 -2.354918 -2.352748
O 8.942947 1.216528 0.717109
C -3.397779 -0.667963 0.927877
C -4.879740 -0.935577 0.774715
C -2.829447 -0.193288 -0.414929
C -5.576171 0.295352 0.231854
C -4.925137 0.720983 -1.079251
C -1.358309 0.089203 -0.334727
C -5.508014 1.997361 -1.645618
C -0.474771 -0.720886 -1.022467
C -0.855145 1.141995 0.517123
C 0.933359 -0.564151 -0.915390
C 0.563116 1.262215 0.655948
C 1.402867 0.440384 -0.061177
C 1.866817 -1.422737 -1.585678
C 3.656704 -0.164516 -0.521794
C 3.251137 -1.177450 -1.338408
C 5.025319 0.197819 -0.207202
C 6.113140 -0.431172 -0.842573
C 5.314204 1.189990 0.748021
C 7.412036 -0.095434 -0.539777
C 6.611688 1.532422 1.058674
C 7.726263 0.903840 0.430316
H -3.240725 0.090371 1.695034
H -5.021602 -1.764194 0.072760
H -3.002305 -0.992955 -1.138220
H -5.488319 1.105230 0.960771
H -5.046697 -0.082253 -1.815247
H -6.568866 1.865872 -1.841345
H -4.999647 2.226914 -2.583712
H -1.878422 -1.714695 1.585876
H -5.120842 -2.088813 2.331602
H -7.334462 -0.338546 0.769779
H 0.969829 2.043295 1.283344
H -4.447285 3.177898 -0.516093
H -0.131898 -2.165446 -2.194015
H 3.973934 -1.819927 -1.814914
H 5.940010 -1.191299 -1.592355
H 4.503959 1.693908 1.255946
H 8.232594 -0.590174 -1.044327
H 6.809209 2.296827 1.799953
O 1.083233 -2.078303 2.087438
O 0.099457 -1.277321 2.385193
H 0.319619 -0.393800 2.003388

ENERGIES
SCF ENERGY: -1714.89645553
SUM OF ELECTRONIC AND ZERO-POINT ENERGIES: -1714.508587
SUM OF ELECTRONIC AND THERMAL ENERGIES: -1714.477626
SUM OF ELECTRONIC AND THERMAL ENTHALPIES: -1714.476681
SUM OF ELECTRONIC AND THERMAL FREE ENERGIES: -1714.573339

==> ISOVITEXIN/WATER/PA/C7/PA/C4'/RAF/C4' <==
52
XYZ-COORDINATES + ENERGIES
O 4.055450 -0.628897 -0.944916
O 3.565767 1.442365 2.050613

O	6.245908	0.483682	2.379944
O	7.546482	-0.166256	-0.043351
O	5.738892	-2.784970	-1.563534
O	1.595795	2.330845	-0.918266
O	2.172457	-2.066939	0.788059
O	-2.164737	-0.417260	0.172811
O	-0.813131	3.150903	-1.257798
O	-8.005219	-0.487600	1.517125
C	4.084663	0.368847	1.268303
C	5.573709	0.598983	1.127150
C	3.438972	0.371452	-0.121207
C	6.169890	-0.438181	0.197541
C	5.449491	-0.403129	-1.145158
C	1.956033	0.144009	-0.068199
C	5.922015	-1.476556	-2.101760
C	1.099883	1.152766	-0.461432
C	1.416978	-1.106569	0.421080
C	-0.313803	1.007622	-0.401917
C	-0.001940	-1.242639	0.481108
C	-0.812660	-0.215622	0.082291
C	-1.214486	2.037426	-0.809325
C	-3.049108	0.550803	-0.194882
C	-2.616156	1.748998	-0.680458
C	-4.422614	0.161486	-0.007493
C	-5.493034	1.062978	-0.316730
C	-4.739221	-1.136709	0.504473
C	-6.780098	0.706036	-0.125192
C	-6.020557	-1.520851	0.697583
C	-7.188007	-0.615662	0.455889
H	3.907046	-0.584769	1.765091
H	5.743716	1.595921	0.707002
H	3.638223	1.347963	-0.567992
H	6.052838	-1.427836	0.646736
H	5.604648	0.578465	-1.607742
H	6.984092	-1.357321	-2.298668
H	5.373514	-1.373847	-3.039675
H	2.637842	1.258387	2.235258
H	5.944225	1.199685	2.948620
H	7.987480	-0.117144	0.811816
H	-0.429078	-2.166137	0.846001
H	4.806318	-2.880164	-1.341029
H	0.809900	2.893879	-1.146759
H	-3.316956	2.512098	-0.977067
H	-5.271665	2.047543	-0.701858
H	-3.934644	-1.813922	0.748809
H	-7.586415	1.391654	-0.352637
H	-6.257210	-2.497985	1.098549
O	-7.938097	-1.352275	-0.644273
O	-9.262096	-0.817979	-0.648816
H	-9.353362	-0.624344	0.314776

ENERGIES
SCF ENERGY: -1714.87918382
SUM OF ELECTRONIC AND ZERO-POINT ENERGIES: -1714.491025
SUM OF ELECTRONIC AND THERMAL ENERGIES: -1714.461193
SUM OF ELECTRONIC AND THERMAL ENTHALPIES: -1714.460249
SUM OF ELECTRONIC AND THERMAL FREE ENERGIES: -1714.553195

==> ISOVITEXIN/WATER/PA/C7/PA/C4'/RAF/C5 <==
52
XYZ-COORDINATES + ENERGIES

O	3.443492	-0.508374	-0.903925
O	2.783570	0.678864	2.514498
O	5.485790	-0.232445	2.671116
O	6.883634	-0.152108	0.211072
O	5.259311	-2.365832	-1.987582
O	0.860769	2.319769	0.276417
O	1.516862	-2.381603	0.286999
O	-2.810944	-0.645750	0.014474
O	-1.593895	3.220572	0.071410
O	-8.990213	-1.622631	0.209302
C	3.363686	-0.121104	1.488873
C	4.846490	0.189009	1.468775
C	2.767404	0.236085	0.119905
C	5.517881	-0.538682	0.321755
C	4.831480	-0.184465	-0.991999
C	1.296682	-0.027565	0.040794
C	5.374527	-0.956536	-2.174744
C	0.430895	1.140406	-0.338385
C	0.764327	-1.354279	0.111599
C	-1.042302	0.932207	-0.144652
C	-0.638131	-1.485337	-0.004803
C	-1.484556	-0.362935	-0.064769
C	-1.966962	2.011444	-0.000900
C	-3.743945	0.345877	0.061924
C	-3.351057	1.647985	0.074569
C	-5.103190	-0.162576	0.107433
C	-6.205206	0.705167	0.220480
C	-5.365656	-1.542365	0.034882
C	-7.494876	0.226086	0.256446
C	-6.653432	-2.031060	0.068554
C	-7.783124	-1.169450	0.179722
H	3.209999	-1.179822	1.695678
H	4.981892	1.267656	1.336378
H	2.952594	1.299336	-0.051370
H	5.441873	-1.615624	0.491961
H	4.950295	0.888254	-1.181944
H	6.430618	-0.736813	-2.306911
H	4.832839	-0.647922	-3.070487
H	1.887258	0.365538	2.677026
H	5.145882	0.304396	3.394696
H	7.302570	-0.311912	1.063861
H	-1.084138	-2.468974	0.041395
H	4.332699	-2.561974	-1.811366
H	0.084859	2.929334	0.265072
H	-4.079957	2.440173	0.139057
H	-6.051571	1.774023	0.284996
H	-4.543953	-2.239274	-0.052917
H	-8.326619	0.913863	0.345719
H	-6.830720	-3.097718	0.008097
O	0.734447	1.221783	-1.773518
O	0.008039	2.295383	-2.357125
H	0.551088	3.068972	-2.146795

ENERGIES
SCF ENERGY: -1714.87573382
SUM OF ELECTRONIC AND ZERO-POINT ENERGIES: -1714.487475
SUM OF ELECTRONIC AND THERMAL ENERGIES: -1714.457373
SUM OF ELECTRONIC AND THERMAL ENTHALPIES: -1714.456429
SUM OF ELECTRONIC AND THERMAL FREE ENERGIES: -1714.550180

==> ISOVITEXIN/WATER/PA/C7/PA/C4'/RAF/C5'/TS <==
52
XYZ-COORDINATES + ENERGIES

O	-4.064283	0.752034	-0.749411
O	-3.404917	-1.828953	1.781951
O	-6.117212	-1.058882	2.346648
O	-7.506467	-0.039483	0.107513
O	-5.875471	2.901235	-0.909934
O	-1.518518	-2.092428	-1.327983
O	-2.168315	1.898348	1.169040
O	2.191763	0.578746	0.074063
O	0.899642	-2.747497	-1.886266
O	8.341651	1.551327	-0.060383
C	-3.990926	-0.652091	1.229875
C	-5.474065	-0.915959	1.081032
C	-3.389401	-0.364835	-0.149955
C	-6.137281	0.247742	0.372328
C	-5.452106	0.502592	-0.965017
C	-1.912937	-0.100701	-0.096645
C	-5.998151	1.713041	-1.690658
C	-1.041641	-0.992558	-0.690443
C	-1.396018	1.058908	0.595143
C	0.366566	-0.803158	-0.658433
C	0.017432	1.245673	0.617290
C	0.842222	0.339423	0.007389
C	1.285100	-1.708877	-1.274632
C	3.080493	-0.261295	-0.511556
C	2.677719	-1.370712	-1.176274
C	4.465008	0.183074	-0.343355
C	5.518912	-0.690416	-0.533893
C	4.734223	1.546756	-0.034813
C	6.853447	-0.266037	-0.329312
C	6.018050	2.016048	0.052491
C	7.134771	1.151773	-0.137746
H	-3.834696	0.197742	1.894052

H	-5.617849	-1.825422	0.488433
H	-3.574506	-1.243179	-0.772021
H	-6.052505	1.139080	0.999381
H	-5.575762	-0.379776	-1.603442
H	-7.056039	1.575205	-1.897909
H	-5.462706	1.824440	-2.635078
H	-2.477023	-1.642763	1.965110
H	-5.794211	-1.865916	2.760009
H	-7.916292	-0.293726	0.941569
H	0.430576	2.107138	1.122924
H	-4.947520	2.998180	-0.669606
H	-0.723814	-2.578577	-1.673455
H	3.399011	-2.010165	-1.660342
H	5.343475	-1.727336	-0.782935
H	3.907932	2.228789	0.104160
H	6.212941	3.061478	0.252555
H	7.657801	-0.868354	-0.729671
O	7.280559	-0.762696	1.457731
O	8.691011	-0.676737	1.555483
H	8.877128	0.232707	1.251739

ENERGIES
SCF ENERGY: -1714.87629569
SUM OF ELECTRONIC AND ZERO-POINT ENERGIES: -1714.488496
SUM OF ELECTRONIC AND THERMAL ENERGIES: -1714.458608
SUM OF ELECTRONIC AND THERMAL ENTHALPIES: -1714.457664
SUM OF ELECTRONIC AND THERMAL FREE ENERGIES: -1714.550663

==> ISOVITEXIN/WATER/PA/C7/PA/C4'/RAF/C6 <==
52
XYZ-COORDINATES + ENERGIES

O	-2.603514	0.934773	-0.127590
O	-4.327802	-2.313899	0.094922
O	-6.290660	-0.487346	0.954970
O	-6.033228	2.106933	-0.133669
O	-3.020390	3.502164	0.974724
O	-0.979888	-0.680994	-2.431499
O	-1.579046	-0.539483	2.289625
O	2.750059	-0.203350	0.533356
O	1.398553	-0.261778	-3.300510
O	8.891794	0.747293	1.283157
C	-3.969865	-0.986270	0.471543
C	-5.186305	-0.141256	0.122953
C	-2.767262	-0.470831	-0.331868
C	-4.920696	1.335417	0.306742
C	-3.718784	1.723087	-0.539290
C	-1.357586	-1.031903	-0.057366
C	-3.308697	3.170706	-0.382120
C	-0.448811	-0.673664	-1.179332
C	-0.783498	-0.628405	1.311525
C	0.921628	-0.464394	-0.999090
C	0.601742	-0.454716	1.432326
C	1.418783	-0.390813	0.311810
C	1.822850	-0.259815	-2.101005
C	3.629236	-0.037219	-0.486090
C	3.194837	-0.056920	-1.781186
C	4.993110	0.157182	-0.028905
C	6.056488	0.303382	-0.939263
C	5.300473	0.213244	1.342961
C	7.349212	0.497058	-0.508334
C	6.591858	0.406697	1.783665
C	7.680869	0.561019	0.877821
H	-3.780094	-0.928149	1.540277
H	-5.445388	-0.321810	-0.926095
H	-2.997897	-0.655997	-1.384018
H	-4.708512	1.540206	1.359052
H	-3.939912	1.529380	-1.594868
H	-4.120897	3.817893	-0.702625
H	-2.436947	3.357645	-1.011415
H	-3.721349	-2.915104	0.544618
H	-6.486718	-1.419181	0.808984
H	-6.810882	1.798226	0.343774
H	1.024300	-0.288700	2.413718
H	-2.309146	2.925902	1.273873
H	-0.206899	-0.515164	-3.039132
H	3.889628	0.089341	-2.592727
H	5.870111	0.262717	-2.003926
H	4.509565	0.105083	2.071958
H	8.150343	0.606263	-1.228830
H	6.802396	0.447906	2.845239
O	-1.352828	-2.521514	-0.148819
O	-1.936614	-3.142880	0.996495
H	-1.210683	-3.185341	1.635868

ENERGIES
SCF ENERGY: -1714.87556735
SUM OF ELECTRONIC AND ZERO-POINT ENERGIES: -1714.486812
SUM OF ELECTRONIC AND THERMAL ENERGIES: -1714.457033
SUM OF ELECTRONIC AND THERMAL ENTHALPIES: -1714.456088
SUM OF ELECTRONIC AND THERMAL FREE ENERGIES: -1714.549228

==> ISOVITEXIN/WATER/PA/C7/PA/C4'/RAF/C6' <==
52
XYZ-COORDINATES + ENERGIES

O	-3.883633	0.252818	-1.084316
O	-3.388051	-0.781821	2.407080
O	-6.133654	0.052081	2.378091
O	-7.372754	-0.162404	-0.154078
O	-5.675420	1.994702	-2.358096
O	-1.246203	-2.379187	-0.071153
O	-2.171972	2.275241	0.151679
O	2.286190	0.840190	0.144144
O	1.218535	-3.088550	-0.070949
O	8.459990	1.927464	0.634710
C	-3.939733	-0.030043	1.328053
C	-5.405908	-0.393553	1.234691
C	-3.240129	-0.404770	0.017119
C	-6.026178	0.266692	0.020358
C	-5.251380	-0.122763	-1.233154
C	-1.777495	-0.067387	0.028171
C	-5.750139	0.575114	-2.480062
C	-0.844270	-1.084130	-0.005731
C	-1.339756	1.308542	0.112346
C	0.553354	-0.824907	0.031756
C	0.063766	1.560549	0.146798
C	0.953587	0.521666	0.106120
C	1.533455	-1.863956	-0.003553
C	3.241411	-0.129288	0.112723
C	2.906727	-1.448123	0.043124
C	4.581862	0.388493	0.173385
C	5.732992	-0.565027	0.069691
C	4.831430	1.748294	0.276391
C	7.067041	0.021731	0.349648
C	6.122471	2.239708	0.378856
C	7.293030	1.375707	0.451472
H	-3.838326	1.038395	1.518038
H	-5.499653	-1.480265	1.137445
H	-3.360837	-1.482093	-0.115772
H	-5.988922	1.351669	0.147807
H	-5.326235	-1.206745	-1.377537
H	-6.792723	0.323548	-2.655187
H	-5.155314	0.237033	-3.330354
H	-2.477990	-0.493005	2.538811
H	-5.820860	-0.440442	3.143990
H	-7.845468	0.019974	0.665451
H	0.416874	2.580513	0.205580
H	-4.764191	2.219816	-2.140456
H	-0.416612	-2.926279	-0.087398
H	3.672712	-2.206565	0.013750
H	5.563902	-1.457196	0.675177
H	4.002790	2.441552	0.294572
H	7.902204	-0.662594	0.433553
H	6.286154	3.306942	0.446136
O	5.643477	-1.039890	-1.334341
O	6.541293	-2.128617	-1.489160
H	7.383272	-1.695780	-1.691707

ENERGIES
SCF ENERGY: -1714.87587072
SUM OF ELECTRONIC AND ZERO-POINT ENERGIES: -1714.487690
SUM OF ELECTRONIC AND THERMAL ENERGIES: -1714.457336
SUM OF ELECTRONIC AND THERMAL ENTHALPIES: -1714.456392
SUM OF ELECTRONIC AND THERMAL FREE ENERGIES: -1714.550717

==> ISOVITEXIN/WATER/PA/C7/PA/C4'/RAF/C7 <==
52

XYZ-COORDINATES + ENERGIES

O	3.207209	0.814677	-0.866206
O	3.250549	-1.351451	2.104749
O	5.911850	-1.580876	1.116808
O	6.807016	0.643904	-0.390641
O	4.728392	0.922181	-3.228275
O	0.874244	2.173851	1.316446
O	1.424206	-1.639100	-1.361603
O	-2.880312	-0.385495	-0.229474
O	-1.525698	2.922300	1.719354
O	-9.070336	-0.727533	-0.981489
C	3.604237	-0.889645	0.806245
C	5.058589	-0.467866	0.858228
C	2.752920	0.319093	0.409428
C	5.480977	0.129747	-0.467283
C	4.554790	1.285043	-0.825299
C	1.274920	0.065551	0.324336
C	4.844425	1.886246	-2.183452
C	0.416794	1.015796	0.764111
C	0.760621	-1.201199	-0.323362
C	-1.023436	0.882473	0.631986
C	-0.724905	-1.233619	-0.508314
C	-1.531074	-0.237976	-0.028172
C	-1.943380	1.888654	1.119941
C	-3.761734	0.536890	0.217315
C	-3.320811	1.651861	0.884338
C	-5.133011	0.209855	-0.086483
C	-6.196671	1.027116	0.350168
C	-5.456736	-0.939596	-0.836553
C	-7.501807	0.719114	0.056641
C	-6.760062	-1.255185	-1.137283
C	-7.850408	-0.439789	-0.705042
H	3.484835	-1.688363	0.073918
H	5.184653	0.283760	1.645025
H	2.930218	1.098237	1.154877
H	5.418325	-0.639452	-1.241315
H	4.649580	2.068363	-0.064391
H	5.862305	2.266112	-2.210297
H	4.152612	2.713023	-2.353606
H	2.410495	-1.826148	1.987854
H	5.670742	-1.943049	1.976068
H	7.382406	-0.073579	-0.103797
H	-1.146633	-2.086019	-1.021907
H	3.837105	0.558740	-3.190543
H	0.070313	2.692243	1.591257
H	-4.025202	2.389261	1.233989
H	-5.995763	1.915885	0.932538
H	-4.665514	-1.586770	-1.188105
H	-8.303696	1.358432	0.403809
H	-6.986680	-2.140759	-1.717634
O	0.934356	-2.185772	0.937841
O	0.715560	-3.531046	0.537528
H	-0.211807	-3.680430	0.763002

ENERGIES
SCF ENERGY: -1714.86530736
SUM OF ELECTRONIC AND ZERO-POINT ENERGIES: -1714.477544
SUM OF ELECTRONIC AND THERMAL ENERGIES: -1714.447490
SUM OF ELECTRONIC AND THERMAL ENTHALPIES: -1714.446546
SUM OF ELECTRONIC AND THERMAL FREE ENERGIES: -1714.540259

==> ISOVITEXIN/WATER/PA/C7/PA/C4'/RAF/C8/TS <==
52
XYZ-COORDINATES + ENERGIES

O	-3.434292	0.147897	1.222223
O	-2.951227	0.356347	-2.413824
O	-5.664623	-0.554003	-2.182621
O	-6.930920	0.201879	0.228426
O	-5.195831	-1.311545	2.878017
O	-0.915117	2.635681	-0.288195
O	-1.580414	-2.013133	0.404754
O	2.780934	-0.346259	0.158240
O	1.489977	3.474375	-0.354596
O	8.968521	-1.147523	0.480460
C	-3.484734	-0.132350	-1.185456
C	-4.965279	0.180320	-1.179675
C	-2.818212	0.575628	-0.001031
C	-5.567750	-0.202901	0.157187
C	-4.817630	0.495014	1.285578
C	-1.339992	0.326246	0.054772
C	-5.303407	0.094267	2.661494
C	-0.455206	1.379740	-0.110539
C	-0.836037	-0.997644	0.236109
C	0.959471	1.188163	-0.094722
C	0.604650	-1.229386	0.124549
C	1.444314	-0.096851	0.079804
C	1.891867	2.284595	-0.197747
C	3.687001	0.659787	0.067776
C	3.272915	1.950097	-0.114879
C	5.053875	0.196696	0.180914
C	6.137242	1.096810	0.170076
C	5.346853	-1.175402	0.302087
C	7.435750	0.656339	0.271032
C	6.643184	-1.625743	0.402827
C	7.754291	-0.730960	0.390188
H	-3.339247	-1.209785	-1.109533
H	-5.105486	1.254257	-1.341921
H	-2.997139	1.646818	-0.117223
H	-5.487846	-1.285621	0.284498
H	-4.931404	1.579348	1.172703
H	-6.352683	0.354538	2.772498
H	-4.722412	0.637714	3.408583
H	-2.041423	0.046552	-2.491400
H	-5.358856	-0.252054	-3.044177
H	-7.384810	-0.174300	-0.533675
H	0.991313	-2.126037	0.591624
H	-4.271945	-1.556046	2.759072
H	-0.107316	3.220317	-0.353129
H	3.993770	2.747617	-0.197679
H	5.959576	2.160303	0.085001
H	4.540064	-1.894708	0.313165
H	8.252936	1.366732	0.262354
H	6.844962	-2.685875	0.492493
O	0.615654	-1.938788	-1.569448
O	0.116142	-3.262426	-1.436210
H	-0.731490	-3.124589	-0.973128

ENERGIES
SCF ENERGY: -1714.87745653
SUM OF ELECTRONIC AND ZERO-POINT ENERGIES: -1714.489900
SUM OF ELECTRONIC AND THERMAL ENERGIES: -1714.459756
SUM OF ELECTRONIC AND THERMAL ENTHALPIES: -1714.458811
SUM OF ELECTRONIC AND THERMAL FREE ENERGIES: -1714.553779

==> ISOVITEXIN/WATER/PA/C7/PA/C4'/RAF/C8a <==
52
XYZ-COORDINATES + ENERGIES

O	3.493294	-0.406521	-1.153094
O	3.195096	1.041733	2.210908
O	5.895700	0.064015	2.174079
O	7.035126	-0.069059	-0.404671
O	5.177435	-2.351241	-2.310889
O	1.003129	2.386490	-0.706831
O	1.679008	-2.152636	0.447561
O	-2.659686	-0.503134	0.005286
O	-1.336471	3.242479	-0.900286
O	-8.840712	-1.364559	-0.452289
C	3.664782	0.149789	1.202636
C	5.142133	0.420836	1.016208
C	2.928070	0.414282	-0.115300
C	5.677162	-0.402611	-0.137597
C	4.868920	-0.113478	-1.396610
C	1.455895	0.168508	-0.006969
C	5.289494	-0.955100	-2.581650
C	0.546619	1.208256	-0.269265
C	0.950450	-1.127706	0.303100
C	-0.844552	1.081070	-0.116955
C	-0.482771	-1.292879	0.435315
C	-1.393473	-0.128919	0.515704
C	-1.743270	2.099643	-0.481152
C	-3.561031	0.503410	-0.228315
C	-3.148073	1.785203	-0.406233
C	-4.928870	0.020758	-0.299300
C	-5.939784	0.765264	-0.933754
C	-5.287057	-1.211675	0.274535
C	-7.238736	0.311061	-0.985834

C	-6.585097	-1.673686	0.229611
C	-7.624584	-0.933446	-0.403883
H	3.520775	-0.885140	1.513285
H	5.286149	1.483470	0.794744
H	3.099765	1.460540	-0.378139
H	5.590819	-1.463268	0.112469
H	4.980603	0.945188	-1.658023
H	6.330997	-0.759413	-2.822738
H	4.670280	-0.685208	-3.438856
H	2.268546	0.835759	2.380507
H	5.662678	0.670357	2.884560
H	7.523516	-0.164425	0.420584
H	-0.880301	-2.286685	0.581235
H	4.263141	-2.527415	-2.064551
H	0.170212	2.961161	-0.850893
H	-3.866604	2.571494	-0.578027
H	-5.695601	1.705243	-1.411630
H	-4.532672	-1.801719	0.777014
H	-7.999174	0.894272	-1.490397
H	-6.841256	-2.621471	0.686923
O	-1.585578	0.249819	1.915506
O	-2.138523	-0.846187	2.641907
H	-1.371778	-1.193822	3.118699

ENERGIES
SCF ENERGY: -1714.87593856
SUM OF ELECTRONIC AND ZERO-POINT ENERGIES: -1714.487582
SUM OF ELECTRONIC AND THERMAL ENERGIES: -1714.457839
SUM OF ELECTRONIC AND THERMAL ENTHALPIES: -1714.456895
SUM OF ELECTRONIC AND THERMAL FREE ENERGIES: -1714.549835

==> ISOVITEXIN/WATER/PA/C7/PA/C5 <==
49
XYZ-COORDINATES + ENERGIES

O	-3.465052	0.578094	-0.913855
O	-2.942525	-1.429530	2.089157
O	-5.674036	-0.487745	2.409940
O	-6.998207	0.141856	-0.018569
O	-5.113061	2.718387	-1.602798
O	-1.009347	-2.355282	-0.880823
O	-1.585190	2.031785	0.844373
O	2.749560	0.469035	0.167678
O	1.643334	-3.118647	-1.408398
O	8.973408	1.281707	0.480404
C	-3.513424	-0.379159	1.303963
C	-5.008469	-0.611423	1.152850
C	-2.835172	-0.407466	-0.066245
C	-5.611792	0.393574	0.192040
C	-4.851736	0.344542	-1.133255
C	-1.354438	-0.177762	-0.032287
C	-5.309064	1.400833	-2.116346
C	-0.515215	-1.239067	-0.473001
C	-0.833595	1.058111	0.448693
C	0.930223	-1.037431	-0.441719
C	0.584297	1.212682	0.489764
C	1.397878	0.200198	0.062782
C	1.895953	-1.998007	-0.899228
C	3.667337	-0.437891	-0.238038
C	3.294949	-1.616746	-0.764412
C	5.048658	0.025086	-0.042806
C	6.105813	-0.889829	0.002084
C	5.328369	1.385738	0.101687
C	7.408694	-0.458724	0.174526
C	6.632023	1.826900	0.273077
C	7.670160	0.902292	0.308541
H	-3.341534	0.582581	1.785387
H	-5.168347	-1.619277	0.752051
H	-3.022012	-1.390100	-0.503444
H	-5.542235	1.393856	0.619122
H	-4.997560	-0.645668	-1.584717
H	-6.371351	1.290735	-2.317383
H	-4.755866	1.274784	-3.048735
H	-3.081869	-1.223259	3.018147
H	-5.453174	-1.257039	2.944574
H	-7.091776	-0.751964	-0.368256
H	1.011823	2.133563	0.861646
H	-4.185090	2.795019	-1.354571
H	4.038238	-2.315763	-1.116210
H	5.911316	-1.949920	-0.080032
H	4.525711	2.108183	0.070461
H	8.227365	-1.163662	0.218060
H	6.850285	2.882008	0.378480
H	9.026298	2.239948	0.572536

ENERGIES
SCF ENERGY: -1563.93329796
SUM OF ELECTRONIC AND ZERO-POINT ENERGIES: -1563.561583
SUM OF ELECTRONIC AND THERMAL ENERGIES: -1563.534188
SUM OF ELECTRONIC AND THERMAL ENTHALPIES: -1563.533244
SUM OF ELECTRONIC AND THERMAL FREE ENERGIES: -1563.621006

==> ISOVITEXIN/WATER/PA/C7/RAF/C1' <==
53
XYZ-COORDINATES + ENERGIES

O	-3.758741	0.969418	-0.674510
O	-3.420948	-2.201698	1.153333
O	-6.158982	-1.483692	1.631257
O	-7.288497	0.148554	-0.379053
O	-5.481718	3.175621	-0.438027
O	-1.212562	-1.673936	-1.773495
O	-2.070234	1.446538	1.700248
O	2.360100	0.245975	0.768710
O	1.253509	-2.270222	-2.235667
O	8.154551	1.357839	-1.417693
C	-3.922494	-0.896534	0.871325
C	-5.385788	-1.044400	0.515942
C	-3.173642	-0.293427	-0.321647
C	-5.947856	0.292938	0.078517
C	-5.123146	0.851649	-1.075273
C	-1.707597	-0.119924	-0.052278
C	-5.564811	2.233712	-1.506184
C	-0.790795	-0.840049	-0.789829
C	-1.253465	0.762491	1.000503
C	0.609753	-0.746180	-0.551240
C	0.151031	0.849562	1.237763
C	1.022679	0.117196	0.481744
C	1.570866	-1.483662	-1.303800
C	3.277765	-0.446188	0.061607
C	2.957846	-1.282798	-0.937726
C	4.686884	-0.109420	0.507443
C	5.717667	-0.980162	-0.126608
C	4.941605	1.351495	0.322096
C	6.827738	-0.474482	-0.713654
C	6.065027	1.816728	-0.282003
C	7.023852	0.923201	-0.802955
H	-3.819164	-0.252595	1.744089
H	-5.483885	-1.760531	-0.306825
H	-3.306256	-0.974959	-1.164475
H	-5.909599	0.987337	0.921810
H	-5.200897	0.171583	-1.931480
H	-6.602004	2.207379	-1.829251
H	-4.940842	2.554710	-2.342077
H	-2.524287	-2.110643	1.495369
H	-5.879431	-2.376917	1.856926
H	-7.793136	-0.269200	0.327519
H	0.517599	1.498771	2.020291
H	-4.571688	3.178348	-0.122147
H	-0.400617	-2.083729	-2.164935
H	3.724861	-1.808190	-1.485263
H	5.568367	-2.048247	-0.049318
H	4.198868	2.032435	0.713128
H	7.581524	-1.129423	-1.130296
H	6.232921	2.882335	-0.382374
H	8.173223	2.322695	-1.439066
O	4.611091	-0.424389	1.955854
O	5.859171	-0.128456	2.554141
H	5.761810	0.795961	2.825122

ENERGIES
SCF ENERGY: -1715.33769716
SUM OF ELECTRONIC AND ZERO-POINT ENERGIES: -1714.936026
SUM OF ELECTRONIC AND THERMAL ENERGIES: -1714.905742
SUM OF ELECTRONIC AND THERMAL ENTHALPIES: -1714.904798
SUM OF ELECTRONIC AND THERMAL FREE ENERGIES: -1714.998062

==> ISOVITEXIN/WATER/PA/C7/RAF/C2/TS <==

53
XYZ-COORDINATES + ENERGIES

O	3.767850	0.902737	0.763406
O	3.222227	-1.923202	-1.521933
O	5.950584	-1.194571	-2.049767
O	7.244386	0.055825	0.128881
O	5.549732	3.073704	0.788909
O	1.212983	-1.875806	1.537047
O	1.928372	1.834277	-1.330033
O	-2.461755	0.633056	-0.248728
O	-1.217639	-2.499368	2.072493
O	-8.641884	1.653171	-0.101195
C	3.778948	-0.691982	-1.067639
C	5.255875	-0.927807	-0.832642
C	3.122787	-0.272981	0.252690
C	5.886486	0.305692	-0.218286
C	5.146924	0.686676	1.058421
C	1.648186	-0.027286	0.119947
C	5.658644	1.966747	1.682422
C	0.758986	-0.856851	0.769898
C	1.149462	1.051255	-0.703903
C	-0.653111	-0.685860	0.663642
C	-0.270615	1.224618	-0.797380
C	-1.111635	0.381902	-0.137699
C	-1.574813	-1.545783	1.321397
C	-3.369909	-0.263440	0.211624
C	-2.968466	-1.282799	1.082935
C	-4.748057	0.251407	0.145812
C	-5.725016	-0.168067	1.050384
C	-5.095899	1.152612	-0.864721
C	-7.022123	0.307804	0.956213
C	-6.390173	1.631367	-0.966405
C	-7.352628	1.207946	-0.051477
H	3.647900	0.086004	-1.819589
H	5.377885	-1.771547	-0.145390
H	3.286084	-1.084298	0.965292
H	5.824489	1.129917	-0.933683
H	5.247138	-0.127324	1.785608
H	6.710510	1.861442	1.934416
H	5.092558	2.160932	2.595097
H	2.304776	-1.765148	-1.771257
H	5.664019	-2.052528	-2.378696
H	7.688929	-0.275998	-0.658830
H	-0.662900	2.032469	-1.398765
H	4.626666	3.142229	0.521692
H	0.412498	-2.327288	1.913595
H	-3.701491	-1.959021	1.492951
H	-5.479543	-0.854058	1.848128
H	-4.352139	1.474758	-1.579606
H	-7.781108	-0.005201	1.659385
H	-6.662515	2.328319	-1.748429
H	-8.750048	2.283628	-0.823113
O	-3.332962	-1.418980	-1.333776
O	-4.479937	-2.167897	-1.434849
H	-5.172489	-1.531899	-1.673841

ENERGIES
SCF ENERGY: -1715.33474323
SUM OF ELECTRONIC AND ZERO-POINT ENERGIES: -1714.934717
SUM OF ELECTRONIC AND THERMAL ENERGIES: -1714.904249
SUM OF ELECTRONIC AND THERMAL ENTHALPIES: -1714.903305
SUM OF ELECTRONIC AND THERMAL FREE ENERGIES: -1714.997653

==> ISOVITEXIN/WATER/PA/C7/RAF/C2' <==
53
XYZ-COORDINATES + ENERGIES

O	3.811551	-0.121881	-1.131814
O	3.276833	0.483667	2.453826
O	5.928647	-0.612287	2.385982
O	7.284292	-0.192341	-0.059837
O	5.495069	-1.837370	-2.591202
O	1.392367	2.601690	0.095501
O	1.873790	-2.116843	-0.176865
O	-2.425172	-0.262695	-0.210830
O	-0.995992	3.540062	0.092863
O	-8.669923	-0.785443	-0.690957
C	3.798753	-0.168173	1.297705
C	5.295549	0.054791	1.295318
C	3.190045	0.444683	0.032225
C	5.896075	-0.497500	0.018637
C	5.213886	0.134690	-1.189011
C	1.703351	0.252491	-0.044077
C	5.698901	-0.427821	-2.507563
C	0.871099	1.352439	-0.000385
C	1.137601	-1.076038	-0.136165
C	-0.545053	1.227108	-0.049699
C	-0.283290	-1.193767	-0.186823
C	-1.069045	-0.075025	-0.146708
C	-1.421493	2.352510	0.002070
C	-3.279920	0.790599	-0.163866
C	-2.830158	2.067247	-0.047889
C	-4.667001	0.382524	-0.250734
C	-5.678696	1.285922	-0.516532
C	-4.983733	-1.064626	-0.015595
C	-7.000581	0.879819	-0.627389
C	-6.406965	-1.437999	-0.245094
C	-7.342024	-0.501657	-0.502193
H	3.589197	-1.236633	1.336062
H	5.498667	1.129450	1.351192
H	3.415698	1.513069	0.049698
H	5.745265	-1.579859	-0.005798
H	5.394502	1.215799	-1.176490
H	6.765769	-0.252007	-2.615987
H	5.171609	0.079039	-3.317597
H	2.340284	0.266541	2.521821
H	5.625028	-0.206330	3.204602
H	7.704343	-0.541639	0.733599
H	-0.731489	-2.174484	-0.262497
H	4.557718	-2.005405	-2.445351
H	0.619091	3.225402	0.111174
H	-3.522227	2.891644	0.020045
H	-5.447797	2.328839	-0.679039
H	-4.318778	-1.705465	-0.596271
H	-7.783099	1.592842	-0.839893
H	-6.674917	-2.484571	-0.176582
H	-8.814529	-1.735029	-0.602411
O	-4.617107	-1.280961	1.386699
O	-4.542588	-2.680818	1.610876
H	-5.412281	-2.892745	1.978384

ENERGIES
SCF ENERGY: -1715.34466762
SUM OF ELECTRONIC AND ZERO-POINT ENERGIES: -1714.943601
SUM OF ELECTRONIC AND THERMAL ENERGIES: -1714.912774
SUM OF ELECTRONIC AND THERMAL ENTHALPIES: -1714.911830
SUM OF ELECTRONIC AND THERMAL FREE ENERGIES: -1715.007452

==> ISOVITEXIN/WATER/PA/C7/RAF/C3/TS <==
53
XYZ-COORDINATES + ENERGIES

O	3.721188	-0.851152	-0.849362
O	3.153857	1.665481	1.767898
O	5.898124	0.922803	2.184507
O	7.191233	-0.035375	-0.138274
O	5.537904	-2.975355	-1.138374
O	1.105067	1.952225	-1.241820
O	1.944474	-2.061279	1.139694
O	-2.487329	-0.807962	0.331341
O	-1.380292	2.596103	-1.618441
O	-8.669778	-1.881540	0.329768
C	3.726887	0.508789	1.162531
C	5.197516	0.795448	0.948266
C	3.063381	0.242726	-0.193041
C	5.839787	-0.345144	0.185012
C	5.095064	-0.580844	-1.123918
C	1.595798	-0.045964	-0.070078
C	5.618961	-1.770529	-1.898520
C	0.678639	0.839000	-0.597466
C	1.135983	-1.223794	0.630737
C	-0.726572	0.630661	-0.482314
C	-0.277228	-1.432850	0.734388
C	-1.146243	-0.533370	0.193958
C	-1.680428	1.543041	-1.000021
C	-3.427667	0.005664	-0.189213
C	-3.087304	1.237319	-0.734258
C	-4.779534	-0.493266	-0.053082

C	-5.881125	0.346057	-0.287822
C	-5.012806	-1.824223	0.324072
C	-7.170153	-0.129831	-0.158833
C	-6.301957	-2.305788	0.452207
C	-7.381116	-1.457572	0.210769
H	3.613682	-0.357299	1.814378
H	5.301346	1.718229	0.368063
H	3.204530	1.137474	-0.803136
H	5.795058	-1.250287	0.796258
H	5.178657	0.315678	-1.748920
H	6.665986	-1.619358	-2.147358
H	5.044624	-1.866481	-2.821554
H	2.237400	1.464832	1.989597
H	5.587844	1.720026	2.625948
H	7.636620	0.208509	0.680490
H	-0.644325	-2.312851	1.243313
H	4.621843	-3.082807	-0.859770
H	0.301086	2.444266	-1.535381
H	-5.734355	1.383074	-0.553592
H	-4.180197	-2.486793	0.508100
H	-8.021021	0.514211	-0.331305
H	-6.483185	-3.334106	0.737456
H	-8.690179	-2.808325	0.596666
H	-3.816181	1.750891	-1.341322
O	-3.238896	2.524792	0.711614
O	-3.463424	3.753206	0.108978
H	-2.595568	4.012361	-0.238417

ENERGIES
SCF ENERGY: -1715.33911963
SUM OF ELECTRONIC AND ZERO-POINT ENERGIES: -1714.938111
SUM OF ELECTRONIC AND THERMAL ENERGIES: -1714.907778
SUM OF ELECTRONIC AND THERMAL ENTHALPIES: -1714.906834
SUM OF ELECTRONIC AND THERMAL FREE ENERGIES: -1715.000882

==> ISOVITEXIN/WATER/PA/C7/RAF/C3' <==
53
XYZ-COORDINATES + ENERGIES

O	3.961291	-0.518382	-1.005342
O	3.536386	1.064397	2.283765
O	6.183349	-0.043596	2.446357
O	7.464353	-0.323950	-0.054386
O	5.587458	-2.600858	-1.970833
O	1.652495	2.512634	-0.525581
O	1.996652	-2.111096	0.502104
O	-2.253380	-0.190409	0.050165
O	-0.715698	3.489571	-0.769208
O	-8.563204	-0.359984	-0.394516
C	4.020208	0.112801	1.338307
C	5.517917	0.305257	1.233657
C	3.384782	0.366343	-0.032529
C	6.078984	-0.585551	0.143701
C	5.362809	-0.310234	-1.173195
C	1.892555	0.206820	-0.021439
C	5.802607	-1.227977	-2.293144
C	1.092217	1.304263	-0.265181
C	1.289550	-1.076144	0.265839
C	-0.327576	1.218908	-0.252711
C	-0.135159	-1.155358	0.274157
C	-0.888398	-0.042141	0.020627
C	-1.170993	2.340722	-0.512091
C	-3.068484	0.859878	-0.200877
C	-2.591615	2.090860	-0.477440
C	-4.494425	0.492000	-0.159935
C	-5.473092	1.508458	0.026582
C	-4.864321	-0.807531	-0.342462
C	-6.834334	1.197819	-0.044826
C	-6.286236	-1.235136	-0.288707
C	-7.241604	-0.091280	-0.249791
H	3.802568	-0.900946	1.673393
H	5.727322	1.350846	0.984619
H	3.633047	1.390696	-0.318458
H	5.928406	-1.630571	0.426962
H	5.549958	0.727905	-1.470985
H	6.866816	-1.105697	-2.476466
H	5.253795	-0.961647	-3.198136
H	2.596687	0.898490	2.420194
H	5.932019	0.593544	3.122817
H	7.901334	-0.412383	0.799870
H	-0.612223	-2.102546	0.482904
H	4.653498	-2.707198	-1.760302
H	0.902534	3.144208	-0.673344
H	-3.264063	2.904160	-0.701088
H	-5.170440	2.525638	0.218724
H	-4.132663	-1.583324	-0.509099
H	-7.579492	1.976550	0.044631
H	-6.532676	-1.922242	-1.102511
H	-8.705562	-1.311303	-0.483722
O	-6.388263	-2.031542	0.941699
O	-7.586067	-2.802839	0.876366
H	-8.146193	-2.382416	1.544011

ENERGIES
SCF ENERGY: -1715.34278984
SUM OF ELECTRONIC AND ZERO-POINT ENERGIES: -1714.940451
SUM OF ELECTRONIC AND THERMAL ENERGIES: -1714.910118
SUM OF ELECTRONIC AND THERMAL ENTHALPIES: -1714.909174
SUM OF ELECTRONIC AND THERMAL FREE ENERGIES: -1715.003583

==> ISOVITEXIN/WATER/PA/C7/RAF/C4/TS <==
53
XYZ-COORDINATES + ENERGIES

O	-3.651188	0.080179	1.005569
O	-3.059718	-0.459345	-2.579807
O	-5.766691	-1.342318	-2.244886
O	-7.114475	-0.127042	-0.075901
O	-5.415795	-1.033077	2.880397
O	-1.006902	2.137852	-0.990945
O	-1.810317	-2.163917	0.805035
O	2.599378	-0.696424	0.356637
O	1.386842	2.838063	-1.281903
O	8.784281	-1.735984	0.103027
C	-3.623643	-0.699084	-1.294563
C	-5.106568	-0.409579	-1.392931
C	-2.998919	0.242234	-0.257566
C	-5.747148	-0.518240	-0.023522
C	-5.039583	0.411098	0.955244
C	-1.527103	0.007378	-0.089786
C	-5.556807	0.292306	2.372332
C	-0.620617	0.957663	-0.477713
C	-1.036087	-1.247830	0.454954
C	0.792478	0.735970	-0.343823
C	0.393382	-1.447333	0.592588
C	1.257493	-0.469152	0.199953
C	1.751005	1.792984	-0.659482
C	3.511393	0.184490	-0.161160
C	3.144690	1.369284	-0.675323
C	4.882420	-0.330853	-0.077456
C	5.971065	0.547652	-0.076694
C	5.121372	-1.704902	-0.001158
C	7.266687	0.067172	-0.019129
C	6.416757	-2.194265	0.055880
C	7.487880	-1.306339	0.045537
H	-3.473725	-1.737801	-0.998802
H	-5.247524	0.606784	-1.775114
H	-3.158215	1.266111	-0.603772
H	-5.661340	-1.549656	0.328013
H	-5.160886	1.446557	0.617964
H	-6.615500	0.535560	2.401248
H	-5.013082	0.998568	3.002045
H	-2.157716	-0.797695	-2.585617
H	-5.438276	-1.216410	-3.141231
H	-7.548902	-0.674162	-0.739235
H	0.746461	-2.381933	1.003708
H	-4.482925	-1.267784	2.827864
H	-0.154134	2.644818	-1.199624
H	3.875696	2.029324	-1.115832
H	5.806505	1.615998	-0.102179
H	4.293207	-2.398587	0.001416
H	8.111289	0.742058	-0.010506
H	6.604272	-3.259099	0.107658
H	8.811761	-2.698658	0.156475
O	1.591398	2.285718	1.316848
O	2.472641	3.394217	1.405096
H	3.346524	2.988711	1.475089

ENERGIES
SCF ENERGY: -1715.32585364
SUM OF ELECTRONIC AND ZERO-POINT ENERGIES: -1714.926727
SUM OF ELECTRONIC AND THERMAL ENERGIES: -1714.896090
SUM OF ELECTRONIC AND THERMAL ENTHALPIES: -1714.895146
SUM OF ELECTRONIC AND THERMAL FREE ENERGIES: -1714.990523

==> ISOVITEXIN/WATER/PA/C7/RAF/C4'/TS <==
53
XYZ-COORDINATES + ENERGIES

O	4.006374	-0.793799	-0.863452
O	3.658716	1.744308	1.771250
O	6.368532	0.857799	2.098326
O	7.536109	-0.163903	-0.265260
O	5.710427	-3.009989	-1.221011
O	1.553346	2.162984	-1.189679
O	2.210230	-1.904513	1.171976
O	-2.154069	-0.348641	0.497585
O	-0.874039	2.946941	-1.525066
O	-8.337468	-0.788315	1.170910
C	4.147789	0.560287	1.145388
C	5.624289	0.768098	0.884529
C	3.429415	0.334676	-0.189326
C	6.181209	-0.403264	0.101100
C	5.382740	-0.595536	-1.183230
C	1.950979	0.134017	-0.025728
C	5.819051	-1.806279	-1.979222
C	1.078455	1.076953	-0.529457
C	1.436711	-1.015334	0.686805
C	-0.331439	0.956118	-0.377474
C	0.021806	-1.131906	0.832276
C	-0.805093	-0.174655	0.314294
C	-1.247963	1.919522	-0.893276
C	-3.042571	0.554154	0.016112
C	-2.647343	1.656003	-0.662737
C	-4.426633	0.185094	0.293284
C	-5.457710	1.140031	0.180958
C	-4.739642	-1.131851	0.701950
C	-6.759797	0.787648	0.403465
C	-6.033739	-1.500405	0.938110
C	-7.093389	-0.576853	0.673678
H	4.008689	-0.301123	1.798569
H	5.758186	1.684928	0.300942
H	3.604750	1.220507	-0.803686
H	6.108556	-1.306365	0.712640
H	5.492407	0.298073	-1.808498
H	6.861895	-1.703808	-2.267341
H	5.206564	-1.870217	-2.880263
H	2.739307	1.593788	2.018644
H	6.104435	1.663764	2.554079
H	8.020272	0.054109	0.538685
H	-0.388042	-1.982983	1.357629
H	4.796322	-3.089423	-0.927628
H	0.761435	2.693640	-1.463926
H	-3.371024	2.347895	-1.063844
H	-5.223840	2.168263	-0.051238
H	-3.946656	-1.851003	0.840073
H	-7.562492	1.507732	0.334294
H	-6.288004	-2.502539	1.256862
H	-8.460151	-1.725528	1.370653
O	-7.363638	-1.147083	-1.150979
O	-8.552667	-0.578404	-1.572215
H	-9.240191	-1.137897	-1.184728

ENERGIES
SCF ENERGY: -1715.32975704
SUM OF ELECTRONIC AND ZERO-POINT ENERGIES: -1714.929928
SUM OF ELECTRONIC AND THERMAL ENERGIES: -1714.899447
SUM OF ELECTRONIC AND THERMAL ENTHALPIES: -1714.898503
SUM OF ELECTRONIC AND THERMAL FREE ENERGIES: -1714.992872

==> ISOVITEXIN/WATER/PA/C7/RAF/C5 <==
53
XYZ-COORDINATES + ENERGIES

O	3.479568	-0.578822	-0.858778
O	2.813508	0.885774	2.449423
O	5.504992	-0.044075	2.695749
O	6.916779	-0.173297	0.246834
O	5.278878	-2.528689	-1.790593
O	0.911099	2.342814	0.080221
O	1.548922	-2.340211	0.455230
O	-2.772744	-0.626669	0.073364
O	-1.549695	3.230945	-0.179836
O	-8.977374	-1.507775	0.332075
C	3.390950	-0.002073	1.497684
C	4.877285	0.289670	1.460580
C	2.806367	0.247837	0.099568
C	5.546083	-0.533625	0.378158
C	4.871352	-0.274712	-0.963231
C	1.334716	-0.014588	0.032302
C	5.412848	-1.138905	-2.081011
C	0.470362	1.122262	-0.436803
C	0.792017	-1.340960	0.205435
C	-1.001554	0.932873	-0.215913
C	-0.607696	-1.475257	0.104141
C	-1.444757	-0.358391	-0.035325
C	-1.914358	2.020419	-0.154863
C	-3.691805	0.374507	0.039472
C	-3.310634	1.664585	-0.058280
C	-5.067821	-0.127034	0.121988
C	-6.116747	0.717638	0.501348
C	-5.350942	-1.460777	-0.183361
C	-7.415219	0.246072	0.565565
C	-6.649762	-1.939548	-0.123935
C	-7.680795	-1.084470	0.251122
H	3.225046	-1.038437	1.790197
H	5.025403	1.353036	1.244687
H	2.997281	1.293438	-0.153727
H	5.455516	-1.593042	0.631134
H	5.000571	0.779335	-1.233451
H	6.472382	-0.941689	-2.220813
H	4.881217	-0.891209	-3.001369
H	1.925663	0.576739	2.658756
H	5.158984	0.543461	3.375684
H	7.334217	-0.293562	1.106731
H	-1.057844	-2.449639	0.231501
H	4.347222	-2.701581	-1.616819
H	0.140185	2.954757	0.030356
H	-4.045493	2.454167	-0.088946
H	-5.922414	1.747020	0.767640
H	-4.555723	-2.128930	-0.479870
H	-8.227392	0.892964	0.866780
H	-6.869253	-2.971362	-0.366540
H	-9.034541	-2.442583	0.101056
O	0.767444	1.082979	-1.873204
O	0.037002	2.103612	-2.539360
H	0.582593	2.891300	-2.399860

ENERGIES
SCF ENERGY: -1715.34330370
SUM OF ELECTRONIC AND ZERO-POINT ENERGIES: -1714.940666
SUM OF ELECTRONIC AND THERMAL ENERGIES: -1714.910612
SUM OF ELECTRONIC AND THERMAL ENTHALPIES: -1714.909668
SUM OF ELECTRONIC AND THERMAL FREE ENERGIES: -1715.002909

==> ISOVITEXIN/WATER/PA/C7/RAF/C5' <==
53
XYZ-COORDINATES + ENERGIES

O	-4.114879	0.763304	-0.727706
O	-3.420598	-1.890500	1.717714
O	-6.138665	-1.163849	2.315231
O	-7.545636	-0.094642	0.110510
O	-5.951949	2.895289	-0.821523
O	-1.538237	-2.011465	-1.425093
O	-2.230408	1.863555	1.233328
O	2.147279	0.617955	0.124958
O	0.892593	-2.618204	-2.007554
O	8.357421	1.516657	0.163372
C	-4.020285	-0.702496	1.205558
C	-5.501018	-0.977044	1.052462
C	-3.425847	-0.363890	-0.165930
C	-6.179174	0.200047	0.381022
C	-5.500049	0.504015	-0.948955
C	-1.952574	-0.083561	-0.105423
C	-6.062143	1.729158	-1.636704
C	-1.072082	-0.938856	-0.736439

C	-1.448889	1.052610	0.634159
C	0.335814	-0.739999	-0.689788
C	-0.036369	1.247180	0.675866
C	0.798163	0.374645	0.033386
C	1.260922	-1.609198	-1.343896
C	3.036159	-0.194047	-0.492324
C	2.655673	-1.265087	-1.217473
C	4.428115	0.241993	-0.283891
C	5.434143	-0.680516	-0.270801
C	4.699844	1.625736	-0.118916
C	6.853863	-0.295970	-0.067432
C	6.022595	2.071219	0.026661
C	7.052440	1.177380	0.024719
H	-3.871112	0.126739	1.896704
H	-5.637457	-1.870032	0.433598
H	-3.601321	-1.224142	-0.815368
H	-6.103098	1.072326	1.035406
H	-5.614020	-0.359737	-1.614074
H	-7.118825	1.584726	-1.845611
H	-5.530568	1.875057	-2.578621
H	-2.497569	-1.697760	1.917470
H	-5.802862	-1.976842	2.706030
H	-7.949435	-0.382536	0.936562
H	0.366960	2.087896	1.222527
H	-5.023929	2.999566	-0.584680
H	-0.742730	-2.475457	-1.793544
H	3.392131	-1.868246	-1.725701
H	5.231726	-1.737019	-0.371296
H	3.890182	2.337925	-0.125907
H	6.236778	3.127249	0.131848
H	8.449692	2.469434	0.294643
H	7.507051	-0.733098	-0.827350
O	7.263529	-0.914963	1.199592
O	8.668565	-1.130934	1.190334
H	9.030502	-0.253560	1.386914

ENERGIES
SCF ENERGY: -1715.34290561
SUM OF ELECTRONIC AND ZERO-POINT ENERGIES: -1714.941393
SUM OF ELECTRONIC AND THERMAL ENERGIES: -1714.910908
SUM OF ELECTRONIC AND THERMAL ENTHALPIES: -1714.909964
SUM OF ELECTRONIC AND THERMAL FREE ENERGIES: -1715.004808

==> ISOVITEXIN/WATER/PA/C7/RAF/C6 <==
53
XYZ-COORDINATES + ENERGIES

O	-2.650597	0.918162	-0.198666
O	-4.342277	-2.312178	0.331151
O	-6.331524	-0.433699	0.993169
O	-6.092193	2.048884	-0.330364
O	-3.099941	3.572951	0.662013
O	-1.028072	-0.849726	-2.356709
O	-1.599824	-0.338476	2.335546
O	2.719802	-0.163255	0.546410
O	1.355860	-0.501408	-3.270059
O	8.897510	0.814378	1.101636
C	-4.000714	-0.950133	0.575013
C	-5.224158	-0.156304	0.139981
C	-2.803628	-0.500852	-0.274973
C	-4.974700	1.334084	0.185801
C	-3.771148	1.654960	-0.686025
C	-1.388817	-1.026366	0.038259
C	-3.375888	3.114962	-0.660042
C	-0.487677	-0.749594	-1.113949
C	-0.811054	-0.508887	1.368376
C	0.885728	-0.531000	-0.958346
C	0.577144	-0.329153	1.469411
C	1.387207	-0.355570	0.345234
C	1.776746	-0.415759	-2.075910
C	3.585071	-0.083094	-0.491494
C	3.158229	-0.190080	-1.774110
C	4.966564	0.142099	-0.058537
C	6.037465	-0.152428	-0.910107
C	5.237570	0.661611	1.210663
C	7.341818	0.075908	-0.511707
C	6.541858	0.895014	1.616197
C	7.592978	0.603224	0.752451
H	-3.815861	-0.783344	1.633168
H	-5.473125	-0.438049	-0.888961
H	-3.036540	-0.782419	-1.304979
H	-4.771534	1.638335	1.215658
H	-3.983870	1.363710	-1.720569
H	-4.192169	3.721521	-1.043538
H	-2.501889	3.252011	-1.299021
H	-3.729797	-2.860689	0.836383
H	-6.535301	-1.371937	0.914269
H	-6.871563	1.765002	0.159669
H	1.005177	-0.089767	2.432927
H	-2.385247	3.033882	1.016949
H	-0.265703	-0.732210	-2.984675
H	3.854817	-0.084558	-2.591511
H	5.855554	-0.579979	-1.885987
H	4.425899	0.897661	1.883218
H	8.171699	-0.157107	-1.164331
H	6.750057	1.305450	2.596017
H	8.944370	1.180536	1.992392
O	-1.369029	-2.514176	0.067627
O	-1.930284	-3.042771	1.268342
H	-1.183974	-3.063382	1.885040

ENERGIES
SCF ENERGY: -1715.34353077
SUM OF ELECTRONIC AND ZERO-POINT ENERGIES: -1714.941554
SUM OF ELECTRONIC AND THERMAL ENERGIES: -1714.911565
SUM OF ELECTRONIC AND THERMAL ENTHALPIES: -1714.910621
SUM OF ELECTRONIC AND THERMAL FREE ENERGIES: -1715.003364

==> ISOVITEXIN/WATER/PA/C7/RAF/C6' <==
53
XYZ-COORDINATES + ENERGIES

O	-3.914617	0.214130	-1.087276
O	-3.421598	-0.617809	2.458473
O	-6.169981	0.176415	2.375090
O	-7.402493	-0.194102	-0.149294
O	-5.721899	1.864253	-2.465980
O	-1.286478	-2.368752	0.023417
O	-2.192149	2.292099	0.072474
O	2.261019	0.842554	0.106774
O	1.179965	-3.086875	0.039431
O	8.482409	1.842583	0.502813
C	-3.975650	0.060355	1.332944
C	-5.437819	-0.323368	1.257592
C	-3.271202	-0.385539	0.047631
C	-6.062677	0.262412	0.007550
C	-5.278134	-0.183035	-1.221279
C	-1.807588	-0.053630	0.040286
C	-5.780036	0.439089	-2.506281
C	-0.879097	-1.074287	0.039908
C	-1.363872	1.322806	0.068915
C	0.520624	-0.819361	0.061641
C	0.041235	1.571176	0.089668
C	0.926044	0.528422	0.084990
C	1.494255	-1.862593	0.059818
C	3.207462	-0.132327	0.104542
C	2.872803	-1.448587	0.082548
C	4.556478	0.382620	0.141801
C	5.692245	-0.593211	0.062426
C	4.807102	1.737163	0.228487
C	7.039839	0.002281	0.277151
C	6.104209	2.232500	0.317861
C	7.213812	1.338498	0.360586
H	-3.885634	1.139099	1.457561
H	-5.519337	-1.414916	1.220301
H	-3.393752	-1.468050	-0.028473
H	-6.040684	1.352990	0.077135
H	-5.340235	-1.273982	-1.307867
H	-6.819116	0.166077	-2.669725
H	-5.178814	0.060108	-3.334539
H	-2.529388	-0.283167	2.602542
H	-5.815400	-0.234041	3.170884
H	-7.886752	0.036595	0.650944
H	0.398090	2.591278	0.108504
H	-4.811578	2.112183	-2.271017
H	-0.462032	-2.921531	0.023615
H	3.635224	-2.211386	0.074479
H	5.536603	-1.432664	0.742458

H 3.982597 2.433930 0.248200
H 7.888931 -0.665509 0.323709
H 6.281194 3.297511 0.379580
H 8.448828 2.802196 0.585715
O 5.601801 -1.152411 -1.289745
O 6.402690 -2.323331 -1.334878
H 7.241708 -2.005080 -1.697096

ENERGIES
SCF ENERGY: -1715.34580224
SUM OF ELECTRONIC AND ZERO-POINT ENERGIES: -1714.944018
SUM OF ELECTRONIC AND THERMAL ENERGIES: -1714.913616
SUM OF ELECTRONIC AND THERMAL ENTHALPIES: -1714.912671
SUM OF ELECTRONIC AND THERMAL FREE ENERGIES: -1715.006559

==> ISOVITEXIN/WATER/PA/C7/RAF/C7 <==
53
XYZ-COORDINATES + ENERGIES
O 3.220810 0.811490 -0.865421
O 3.329486 -1.341539 2.113513
O 5.969654 -1.570262 1.073076
O 6.829747 0.646893 -0.464755
O 4.691429 0.913889 -3.258103
O 0.930225 2.189631 1.295287
O 1.440002 -1.651247 -1.325228
O -2.844134 -0.404974 -0.158604
O -1.494377 2.943085 1.709409
O -9.050300 -0.644995 -0.989493
C 3.654998 -0.886851 0.805091
C 5.109026 -0.460614 0.825727
C 2.792408 0.317197 0.418971
C 5.503011 0.131324 -0.511025
C 4.568209 1.284274 -0.853397
C 1.313317 0.058413 0.356568
C 4.828054 1.881606 -2.219184
C 0.460458 1.018872 0.779673
C 0.796248 -1.219645 -0.269668
C -0.983712 0.884879 0.663239
C -0.691651 -1.256843 -0.435045
C -1.495085 -0.247961 0.030115
C -1.899057 1.901033 1.136214
C -3.711158 0.536308 0.261747
C -3.288871 1.660198 0.894428
C -5.101216 0.208720 -0.058052
C -6.157050 0.841728 0.607964
C -5.391671 -0.736294 -1.046637
C -7.468800 0.546193 0.289553
C -6.703403 -1.032905 -1.374880
C -7.741114 -0.389432 -0.706390
H 3.523929 -1.690425 0.080194
H 5.248653 0.295448 1.605957
H 2.978835 1.098649 1.159770
H 5.424535 -0.641065 -1.280406
H 4.677132 2.070061 -0.096985
H 5.844530 2.262959 -2.268740
H 4.131375 2.706709 -2.377239
H 2.492025 -1.824120 2.017963
H 5.744827 -1.929758 1.937901
H 7.412064 -0.069076 -0.188283
H -1.119817 -2.119017 -0.926227
H 3.802420 0.547740 -3.198848
H 0.145253 2.722788 1.571230
H -3.996792 2.414889 1.198286
H -5.959422 1.556723 1.393666
H -4.590228 -1.233183 -1.573239
H -8.288535 1.025720 0.805901
H -6.930014 -1.756515 -2.147272
H -9.115445 -1.303216 -1.691695
O 0.997151 -2.189969 0.978438
O 0.775152 -3.537276 0.586924
H -0.141729 -3.694299 0.847661

ENERGIES
SCF ENERGY: -1715.33063254
SUM OF ELECTRONIC AND ZERO-POINT ENERGIES: -1714.929339
SUM OF ELECTRONIC AND THERMAL ENERGIES: -1714.898996
SUM OF ELECTRONIC AND THERMAL ENTHALPIES: -1714.898052
SUM OF ELECTRONIC AND THERMAL FREE ENERGIES: -1714.992413

==> ISOVITEXIN/WATER/PA/C7/RAF/C8/TS <==
53
XYZ-COORDINATES + ENERGIES
O 3.456867 0.030761 -1.236988
O 3.009757 0.542790 2.373209
O 5.727494 -0.364377 2.192109
O 6.962722 0.200690 -0.291630
O 5.212526 -1.562504 -2.773669
O 0.963631 2.642624 0.067799
O 1.609166 -2.046612 -0.203253
O -2.748290 -0.359010 -0.073954
O -1.455447 3.491788 0.068957
O -8.966984 -1.066988 -0.461990
C 3.534443 -0.043986 1.184792
C 5.012858 0.276410 1.137359
C 2.854521 0.561523 -0.047907
C 5.605848 -0.213182 -0.168738
C 4.837603 0.377224 -1.345766
C 1.375225 0.313181 -0.066558
C 5.311093 -0.142189 -2.685713
C 0.495740 1.378956 0.009465
C 0.867419 -1.022233 -0.121365
C -0.921577 1.192087 0.021840
C -0.573088 -1.238520 0.023682
C -1.409151 -0.106178 -0.033203
C -1.845217 2.295562 0.025877
C -3.638230 0.658824 -0.086378
C -3.237271 1.952425 -0.039641
C -5.025133 0.192514 -0.170269
C -6.085577 1.038866 0.172700
C -5.308341 -1.105800 -0.603865
C -7.394407 0.606474 0.072434
C -6.617553 -1.545298 -0.709890
C -7.659557 -0.686009 -0.374240
H 3.393826 -1.124655 1.200310
H 5.145662 1.361244 1.206911
H 3.036612 1.638038 -0.024594
H 5.538391 -1.303659 -0.201409
H 4.946200 1.467797 -1.331059
H 6.356920 0.114551 -2.831924
H 4.717258 0.327792 -3.471537
H 2.101423 0.238475 2.483051
H 5.424477 0.005763 3.027793
H 7.428818 -0.088667 0.500377
H -0.965542 -2.175890 -0.348507
H 4.293655 -1.802427 -2.613740
H 0.169262 3.240345 0.086061
H -3.959642 2.752616 -0.079159
H -5.893517 2.038014 0.536832
H -4.504087 -1.774595 -0.872979
H -8.216820 1.254235 0.342075
H -6.838421 -2.547633 -1.053805
H -9.024823 -1.975383 -0.780195
O -0.562302 -1.777589 1.782479
O -0.084047 -3.113592 1.774781
H 0.771719 -3.033715 1.315009

ENERGIES
SCF ENERGY: -1715.34414211
SUM OF ELECTRONIC AND ZERO-POINT ENERGIES: -1714.943203
SUM OF ELECTRONIC AND THERMAL ENERGIES: -1714.912758
SUM OF ELECTRONIC AND THERMAL ENTHALPIES: -1714.911814
SUM OF ELECTRONIC AND THERMAL FREE ENERGIES: -1715.006463

==> ISOVITEXIN/WATER/PA/C7/RAF/C8a <==
53
XYZ-COORDINATES + ENERGIES
O 3.527451 -0.197564 -1.200858
O 3.237716 0.576633 2.379206
O 5.943062 -0.343600 2.146176
O 7.070768 0.034923 -0.419178
O 5.226216 -1.891914 -2.697921
O 1.052830 2.466612 -0.260530
O 1.705490 -2.203551 0.053717
O -2.627194 -0.493315 -0.089736
O -1.292923 3.349494 -0.330681
O -8.845872 -1.142840 -0.661582

C 3.707996 -0.096116 1.213927
C 5.182140 0.219070 1.078759
C 2.967197 0.415422 -0.026542
C 5.719984 -0.364833 -0.211822
C 4.900555 0.141851 -1.392919
C 1.493724 0.161227 0.034803
C 5.319352 -0.468357 -2.712613
C 0.592078 1.231292 -0.043860
C 0.981303 -1.169860 0.100805
C -0.805503 1.084323 0.074251
C -0.453076 -1.352053 0.198694
C -1.360203 -0.217714 0.482826
C -1.692718 2.153444 -0.111858
C -3.513220 0.546321 -0.149212
C -3.107443 1.831931 -0.101253
C -4.899512 0.092091 -0.298699
C -5.865813 0.913286 -0.890439
C -5.276996 -1.172036 0.162017
C -7.177765 0.491552 -1.004689
C -6.589819 -1.602456 0.051337
C -7.538419 -0.767303 -0.529824
H 3.572602 -1.172969 1.315263
H 5.315191 1.305914 1.063133
H 3.142534 1.491860 -0.087777
H 5.651659 -1.454672 -0.163380
H 5.006007 1.230880 -1.459486
H 6.356357 -0.219118 -2.921548
H 4.689361 -0.056460 -3.502864
H 2.314667 0.331587 2.511765
H 5.692642 0.102220 2.961906
H 7.566673 -0.196501 0.373961
H -0.854014 -2.354372 0.161124
H 4.316492 -2.122897 -2.482294
H 0.228455 3.061546 -0.312275
H -3.825600 2.636750 -0.136347
H -5.588441 1.882176 -1.281468
H -4.544175 -1.816914 0.625046
H -7.926749 1.119116 -1.467425
H -6.885971 -2.577535 0.416663
H -8.969022 -2.028620 -0.300919
O -1.557729 -0.090887 1.924652
O -2.116258 -1.295919 2.444549
H -1.350188 -1.733057 2.842289

ENERGIES
SCF ENERGY: -1715.34384048
SUM OF ELECTRONIC AND ZERO-POINT ENERGIES: -1714.943056
SUM OF ELECTRONIC AND THERMAL ENERGIES: -1714.912605
SUM OF ELECTRONIC AND THERMAL ENTHALPIES: -1714.911661
SUM OF ELECTRONIC AND THERMAL FREE ENERGIES: -1715.006334

==> ISOVITEXIN/WATER/RAF/C1' <==
54
XYZ-COORDINATES + ENERGIES
O -3.789079 0.871375 -0.798794
O -3.286869 -2.177685 1.183626
O -5.982554 -1.466190 1.798947
O -7.280266 0.057769 -0.200031
O -5.565058 3.083911 -0.553992
O -1.167999 -2.114132 -1.357426
O -2.094363 1.962107 0.935336
O 2.327513 0.502613 0.594684
O 1.318420 -2.826339 -1.504423
O 8.260710 0.744069 -1.558802
C -3.820355 -0.905487 0.845598
C -5.305824 -1.081263 0.607074
C -3.162651 -0.365589 -0.432071
C -5.912728 0.229800 0.148777
C -5.185312 0.733034 -1.091015
C -1.694623 -0.110618 -0.225976
C -5.672337 2.084929 -1.564985
C -0.755811 -1.029744 -0.673605
C -1.234662 1.018125 0.482062
C 0.623783 -0.852793 -0.412258
C 0.110918 1.222796 0.755942
C 1.012526 0.280049 0.307544
C 1.618898 -1.800723 -0.857938
C 3.267120 -0.374420 0.187213
C 2.983593 -1.488982 -0.508132
C 4.660576 0.093783 0.555854
C 5.695598 -0.956253 0.335696
C 4.954130 1.382102 -0.142386
C 6.847988 -0.703457 -0.327376
C 6.118907 1.589604 -0.808606
C 7.084807 0.566161 -0.904106
H -3.666889 -0.196605 1.663810
H -5.456359 -1.838568 -0.169246
H -3.306978 -1.089327 -1.235487
H -5.814731 0.966222 0.950768
H -5.308925 0.008151 -1.902181
H -6.723240 2.013173 -1.832537
H -5.101442 2.374553 -2.448177
H -2.413568 -2.054407 1.571264
H -5.687005 -2.350087 2.040954
H -7.726779 -0.335388 0.557941
H 0.439114 2.097972 1.296342
H -4.631109 3.252788 -0.394304
H -0.361484 -2.636199 -1.587337
H -2.963417 1.835009 0.512508
H 3.770505 -2.156731 -0.822920
H 5.511411 -1.923397 0.782796
H 4.205713 2.159135 -0.074101
H 7.605686 -1.468477 -0.433850
H 6.316653 2.542128 -1.285560
H 8.310956 1.637229 -1.921285
O 4.511685 0.330248 2.012247
O 5.741663 0.808083 2.523021
H 5.644246 1.769870 2.471645

ENERGIES
SCF ENERGY: -1715.80136521
SUM OF ELECTRONIC AND ZERO-POINT ENERGIES: -1715.385394
SUM OF ELECTRONIC AND THERMAL ENERGIES: -1715.355340
SUM OF ELECTRONIC AND THERMAL ENTHALPIES: -1715.354396
SUM OF ELECTRONIC AND THERMAL FREE ENERGIES: -1715.446890

==> ISOVITEXIN/WATER/RAF/C2 <==
54
XYZ-COORDINATES + ENERGIES
O -3.746145 0.957487 -0.723675
O -3.121775 -2.169664 1.093608
O -5.780840 -1.498355 1.892223
O -7.195250 0.099617 0.030945
O -5.500313 3.151600 -0.289301
O -1.135794 -1.958196 -1.572864
O -1.961581 1.989631 0.954017
O 2.457331 0.669850 0.217510
O 1.359583 -2.549902 -2.026380
O 8.631528 1.704885 -0.059512
C -3.678348 -0.887130 0.841775
C -5.174833 -1.059870 0.680627
C -3.093863 -0.289107 -0.445012
C -5.811443 0.265637 0.311882
C -5.155439 0.824589 -0.943609
C -1.619262 -0.026651 -0.311229
C -5.668632 2.194154 -1.332212
C -0.691593 -0.907886 -0.859333
C -1.129160 1.075098 0.406707
C 0.695879 -0.720897 -0.674404
C 0.236476 1.291708 0.600063
C 1.123924 0.391227 0.064650
C 1.672251 -1.611272 -1.260030
C 3.377799 -0.391222 0.157082
C 3.041796 -1.377135 -0.909539
C 4.768111 0.185033 0.061602
C 5.848371 -0.611768 -0.322137
C 5.003013 1.510542 0.427557
C 7.134279 -0.094612 -0.352903
C 6.284482 2.036757 0.393861
C 7.349494 1.232440 -0.000254
H -3.481123 -0.214038 1.680634
H -5.367293 -1.785134 -0.116749
H -3.278016 -0.979801 -1.269250
H -5.672236 0.968421 1.137629
H -5.322857 0.134395 -1.776778
H -6.733582 2.132612 -1.540118

H	-5.150208	2.520313	-2.234760
H	-2.230269	-2.057217	1.441224
H	-5.471569	-2.391541	2.076426
H	-7.599100	-0.326471	0.794905
H	0.579891	2.154703	1.150789
H	-4.558958	3.323247	-0.184920
H	-0.348777	-2.435709	-1.922969
H	-2.858642	1.865820	0.592293
H	3.800681	-1.995242	-1.360226
H	5.706988	-1.646735	-0.597780
H	4.184842	2.143298	0.736537
H	7.971721	-0.708881	-0.653089
H	6.463962	3.068296	0.669062
H	8.654201	2.635991	0.191104
O	3.165064	-1.024174	1.461305
O	3.937317	-2.206393	1.525100
H	4.757535	-1.915242	1.951371

ENERGIES
SCF ENERGY: -1715.81396214
SUM OF ELECTRONIC AND ZERO-POINT ENERGIES: -1715.397095
SUM OF ELECTRONIC AND THERMAL ENERGIES: -1715.367132
SUM OF ELECTRONIC AND THERMAL ENTHALPIES: -1715.366188
SUM OF ELECTRONIC AND THERMAL FREE ENERGIES: -1715.458392

==> ISOVITEXIN/WATER/RAF/C2' <==
54
XYZ-COORDINATES + ENERGIES

O	3.811304	-0.451299	-1.050265
O	3.171053	1.385223	2.063880
O	5.739221	0.222067	2.530562
O	7.226611	-0.299242	0.179980
O	5.489495	-2.657958	-1.723649
O	1.353732	2.656863	-0.416510
O	1.894855	-2.077848	-0.185505
O	-2.412564	-0.280889	-0.273834
O	-1.064388	3.548071	-0.483180
O	-8.661542	-0.973956	-0.299672
C	3.695418	0.363867	1.227305
C	5.204583	0.497278	1.239645
C	3.179901	0.524620	-0.209523
C	5.822134	-0.497830	0.277385
C	5.235072	-0.305572	-1.114462
C	1.691985	0.321344	-0.289214
C	5.736255	-1.312938	-2.126229
C	0.837763	1.413623	-0.358120
C	1.120212	-0.965901	-0.235082
C	-0.566199	1.244640	-0.356957
C	-0.253237	-1.166353	-0.225567
C	-1.070305	-0.055090	-0.281903
C	-1.477895	2.365297	-0.419849
C	-3.291985	0.750620	-0.327037
C	-2.873048	2.043245	-0.392198
C	-4.668869	0.303725	-0.295531
C	-5.710260	1.132129	-0.659928
C	-4.935269	-1.092837	0.181143
C	-7.027828	0.691726	-0.634474
C	-6.360548	-1.516375	0.102775
C	-7.331382	-0.649162	-0.252386
H	3.417267	-0.622227	1.608737
H	5.476136	1.512222	0.931365
H	3.440840	1.522221	-0.565884
H	5.604185	-1.510550	0.626760
H	5.473608	0.701429	-1.472147
H	6.812073	-1.205784	-2.236797
H	5.260581	-1.110534	-3.086806
H	2.253705	1.177449	2.271920
H	5.432884	0.905909	3.135312
H	7.588480	-0.331452	1.072382
H	-0.666812	-2.163046	-0.187875
H	4.539029	-2.808167	-1.740267
H	0.585979	3.281943	-0.458267
H	2.810734	-1.841100	-0.419879
H	-3.585532	2.853020	-0.402196
H	-5.511263	2.136827	-1.003997
H	-4.291786	-1.806636	-0.335993
H	-7.835593	1.347402	-0.923358
H	-6.599715	-2.537704	0.370125
H	-8.786231	-1.883739	-0.004717
O	-4.479712	-1.083931	1.572645
O	-4.319608	-2.429917	1.993094
H	-5.145216	-2.615296	2.462453

ENERGIES
SCF ENERGY: -1715.80912046
SUM OF ELECTRONIC AND ZERO-POINT ENERGIES: -1715.393392
SUM OF ELECTRONIC AND THERMAL ENERGIES: -1715.363034
SUM OF ELECTRONIC AND THERMAL ENTHALPIES: -1715.362090
SUM OF ELECTRONIC AND THERMAL FREE ENERGIES: -1715.455835

==> ISOVITEXIN/WATER/RAF/C3 <==
54
XYZ-COORDINATES + ENERGIES

O	3.638221	-1.072167	-0.695497
O	3.170082	2.155566	0.990025
O	5.857375	1.482686	1.663816
O	7.138497	-0.238737	-0.183020
O	5.385750	-3.274194	-0.240824
O	1.028719	1.858250	-1.514814
O	1.945336	-1.987763	1.127144
O	-2.481051	-0.636296	0.598906
O	-1.491739	2.552077	-1.718654
O	-8.594203	-2.063386	-0.026602
C	3.689683	0.851152	0.773117
C	5.175852	0.989573	0.515332
C	3.021275	0.199008	-0.444567
C	5.770836	-0.364617	0.185018
C	5.035607	-0.978409	-0.998971
C	1.552763	-0.030081	-0.212597
C	5.505853	-2.375609	-1.341020
C	0.609784	0.841341	-0.741364
C	1.090191	-1.087540	0.592397
C	-0.771550	0.693254	-0.458718
C	-0.260375	-1.275510	0.873607
C	-1.169672	-0.388345	0.343583
C	-1.743935	1.639302	-0.926124
C	-3.464656	0.151009	0.058649
C	-3.129149	1.535226	-0.319066
C	-4.766784	-0.413964	0.052042
C	-5.903611	0.357918	-0.280966
C	-4.962613	-1.774722	0.378501
C	-7.161892	-0.205480	-0.301318
C	-6.224054	-2.335166	0.359314
C	-7.326766	-1.554165	0.014940
H	3.532492	0.225933	1.656435
H	5.330693	1.667313	-0.330613
H	3.167624	0.842977	-1.312962
H	5.669336	-1.018076	1.055435
H	5.166715	-0.337316	-1.876679
H	6.557706	-2.342821	-1.612462
H	4.931794	-2.740421	-2.193738
H	2.306840	2.079761	1.410653
H	5.546510	2.378108	1.834677
H	7.592809	0.219011	0.532862
H	-0.584756	-2.117331	1.466921
H	4.449871	-3.421378	-0.070961
H	0.240003	2.357628	-1.817546
H	2.814425	-1.902880	0.692080
H	-5.804393	1.408183	-0.513490
H	-4.114419	-2.390425	0.637821
H	-8.029349	0.387782	-0.555798
H	-6.365017	-3.380251	0.604860
H	-8.583205	-3.001796	0.196180
H	-3.854782	1.959398	-1.007383
O	-3.143101	2.319590	0.908843
O	-3.104447	3.698985	0.567793
H	-2.167114	3.924677	0.656043

ENERGIES
SCF ENERGY: -1715.81838165
SUM OF ELECTRONIC AND ZERO-POINT ENERGIES: -1715.401162
SUM OF ELECTRONIC AND THERMAL ENERGIES: -1715.370954
SUM OF ELECTRONIC AND THERMAL ENTHALPIES: -1715.370010
SUM OF ELECTRONIC AND THERMAL FREE ENERGIES: -1715.463040

==> ISOVITEXIN/WATER/RAF/C3/TS <==

54
XYZ-COORDINATES + ENERGIES

O	-3.719035	-0.953610	0.758020
O	-3.073803	2.036600	-1.271033
O	-5.728203	1.308059	-2.044055
O	-7.159510	-0.136962	-0.074586
O	-5.504586	-3.154901	0.511351
O	-1.070925	1.990931	1.368048
O	-1.965778	-2.125461	-0.855719
O	2.466034	-0.807511	-0.249655
O	1.447366	2.630270	1.656850
O	8.632285	-1.934521	-0.422752
C	-3.633552	0.773634	-0.939076
C	-5.130568	0.962101	-0.798647
C	-3.053732	0.261278	0.386469
C	-5.774549	-0.328420	-0.332121
C	-5.126623	-0.787040	0.967351
C	-1.582503	-0.030029	0.267419
C	-5.656436	-2.111762	1.470764
C	-0.644524	0.877168	0.744327
C	-1.112997	-1.190360	-0.376185
C	0.741703	0.655879	0.570919
C	0.243602	-1.443771	-0.552624
C	1.143332	-0.516555	-0.077349
C	1.732139	1.592563	1.033475
C	3.433696	0.023522	0.195756
C	3.118638	1.289949	0.683692
C	4.772164	-0.492060	0.032994
C	5.888813	0.346752	0.190503
C	4.980444	-1.841707	-0.292639
C	7.167592	-0.147110	0.036849
C	6.259365	-2.340508	-0.443998
C	7.353933	-1.492838	-0.278972
H	-3.435250	0.047675	-1.732193
H	-5.324723	1.747349	-0.060892
H	-3.226210	1.012349	1.158607
H	-5.632444	-1.094952	-1.098622
H	-5.284514	-0.026881	1.739334
H	-6.719659	-2.018409	1.675490
H	-5.139205	-2.368088	2.396302
H	-2.181442	1.902531	-1.608020
H	-5.423595	2.187547	-2.290784
H	-7.556411	0.230649	-0.871841
H	0.577118	-2.350764	-1.034192
H	-4.565488	-3.342778	0.416400
H	-0.276775	2.500577	1.646490
H	-2.855875	-1.967756	-0.490367
H	5.760927	1.395866	0.414959
H	4.136538	-2.503892	-0.416641
H	8.029592	0.495389	0.148950
H	6.421979	-3.382339	-0.688151
H	8.636340	-2.872415	-0.649374
H	3.875906	1.832693	1.227704
O	3.171590	2.439389	-0.836481
O	3.376387	3.726236	-0.369902
H	2.488997	4.063390	-0.177282

ENERGIES
SCF ENERGY: -1715.80113523
SUM OF ELECTRONIC AND ZERO-POINT ENERGIES: -1715.385473
SUM OF ELECTRONIC AND THERMAL ENERGIES: -1715.355421
SUM OF ELECTRONIC AND THERMAL ENTHALPIES: -1715.354477
SUM OF ELECTRONIC AND THERMAL FREE ENERGIES: -1715.447455

==> ISOVITEXIN/WATER/RAF/C3' <==
54
XYZ-COORDINATES + ENERGIES

O	3.947893	-0.724595	-0.903898
O	3.491229	1.696520	1.817801
O	6.036698	0.558280	2.412510
O	7.412171	-0.481913	0.164101
O	5.504337	-3.093210	-1.175170
O	1.630667	2.550662	-0.779514
O	2.010885	-2.077637	0.306062
O	-2.234534	-0.159180	-0.018061
O	-0.765076	3.509570	-0.956303
O	-8.546242	-0.436073	-0.156345
C	3.949256	0.515255	1.175407
C	5.461062	0.589907	1.110821
C	3.383969	0.419622	-0.248066
C	6.006694	-0.598977	0.344989
C	5.372547	-0.658104	-1.038445
C	1.887489	0.265696	-0.241633
C	5.793757	-1.867922	-1.843908
C	1.070640	1.361598	-0.484119
C	1.273650	-0.965701	0.064217
C	-0.337869	1.252869	-0.415805
C	-0.105210	-1.107000	0.138616
C	-0.883976	0.007075	-0.101202
C	-1.211876	2.377268	-0.664333
C	-3.071695	0.875806	-0.253403
C	-2.620741	2.110106	-0.567960
C	-4.488093	0.490001	-0.149749
C	-5.472005	1.493449	0.065702
C	-4.842741	-0.818843	-0.300548
C	-6.829838	1.157733	0.071838
C	-6.254379	-1.267990	-0.192737
C	-7.224752	-0.140049	-0.095569
H	3.655686	-0.369610	1.746706
H	5.751431	1.512099	0.596779
H	3.664545	1.319244	-0.797962
H	5.768455	-1.514584	0.892678
H	5.635450	0.246883	-1.596060
H	6.868496	-1.837057	-2.002147
H	5.292905	-1.836219	-2.812329
H	2.577847	1.565418	2.094230
H	5.771686	1.359261	2.877175
H	7.813307	-0.384868	1.034723
H	-0.552477	-2.062697	0.367511
H	4.549064	-3.208657	-1.154193
H	0.890517	3.191670	-0.917984
H	2.924940	-1.927655	0.002475
H	-3.313396	2.910391	-0.775819
H	-5.177825	2.518507	0.227074
H	-4.106026	-1.584798	-0.488778
H	-7.582839	1.924151	0.194825
H	-6.524316	-1.943441	-1.009427
H	-8.673473	-1.392959	-0.210331
O	-6.295627	-2.087891	1.023342
O	-7.489005	-2.869901	0.985267
H	-7.996473	-2.512923	1.727823

ENERGIES
SCF ENERGY: -1715.80695054
SUM OF ELECTRONIC AND ZERO-POINT ENERGIES: -1715.390691
SUM OF ELECTRONIC AND THERMAL ENERGIES: -1715.360454
SUM OF ELECTRONIC AND THERMAL ENTHALPIES: -1715.359509
SUM OF ELECTRONIC AND THERMAL FREE ENERGIES: -1715.452803

==> ISOVITEXIN/WATER/RAF/C4 <==
54
XYZ-COORDINATES + ENERGIES

O	3.594259	-0.774127	-0.911158
O	3.098055	1.575267	1.868609
O	5.741077	0.604953	2.328858
O	7.077654	-0.307566	0.010117
O	5.283507	-3.013499	-1.322128
O	1.071497	2.296818	-0.603163
O	1.855616	-2.296608	0.404573
O	-2.549831	-0.749460	0.340396
O	-2.048783	2.614586	0.876932
O	-8.680227	-1.998588	0.305104
C	3.604363	0.434047	1.189982
C	5.102692	0.614918	1.056083
C	2.976316	0.312283	-0.205769
C	5.692050	-0.522059	0.245675
C	5.001686	-0.597898	-1.109260
C	1.499123	0.033903	-0.126044
C	5.472380	-1.754670	-1.963929
C	0.574527	1.061905	-0.320515
C	1.017175	-1.241134	0.203164
C	-0.803981	0.838897	-0.197306
C	-0.339965	-1.487284	0.346703
C	-1.216146	-0.438443	0.150963
C	-1.838864	1.926244	-0.269147
C	-3.521756	0.131078	0.043917

C	-3.226578	1.413924	-0.340539
C	-4.856296	-0.426971	0.116160
C	-5.984849	0.408110	0.171750
C	-5.037444	-1.818588	0.130469
C	-7.252688	-0.129237	0.235437
C	-6.304637	-2.361081	0.189496
C	-7.413905	-1.515890	0.242907
H	3.401041	-0.473619	1.764574
H	5.304813	1.561969	0.545061
H	3.157838	1.237906	-0.753077
H	5.538501	-1.461126	0.784196
H	5.171798	0.336421	-1.654362
H	6.536925	-1.649713	-2.155875
H	4.937517	-1.729689	-2.914169
H	2.211758	1.379528	2.190946
H	5.456742	1.386825	2.813935
H	7.503644	-0.186823	0.865929
H	-0.702086	-2.473005	0.598843
H	4.338407	-3.190554	-1.282035
H	0.351211	2.879370	-0.892015
H	2.731193	-2.078233	0.038984
H	-4.000129	2.107582	-0.623442
H	-5.871713	1.482477	0.189690
H	-4.180806	-2.474331	0.083003
H	-8.125734	0.505659	0.288753
H	-6.449750	-3.433491	0.193597
H	-8.673075	-2.963960	0.313238
O	-1.449582	2.742076	-1.393824
O	-2.318470	3.851167	-1.515754
H	-2.895502	3.584321	-2.245455

ENERGIES
SCF ENERGY: -1715.77763148
SUM OF ELECTRONIC AND ZERO-POINT ENERGIES: -1715.360894
SUM OF ELECTRONIC AND THERMAL ENERGIES: -1715.330903
SUM OF ELECTRONIC AND THERMAL ENTHALPIES: -1715.329958
SUM OF ELECTRONIC AND THERMAL FREE ENERGIES: -1715.422133

==> ISOVITEXIN/WATER/RAF/C4a <==
54
XYZ-COORDINATES + ENERGIES

O	3.646045	-0.495228	-0.914041
O	2.679188	1.181729	2.207569
O	5.237072	0.099575	2.845612
O	6.935470	-0.301562	0.618564
O	5.441742	-2.627801	-1.515722
O	0.996781	2.461889	-0.323163
O	1.772379	-2.248004	-0.260716
O	-2.649053	-0.698838	-0.228888
O	-1.413111	3.130349	0.320315
O	-8.779907	-1.640699	0.629323
C	3.308836	0.216265	1.377583
C	4.805345	0.401228	1.523000
C	2.909573	0.419822	-0.090298
C	5.534479	-0.539776	0.585074
C	5.065595	-0.308567	-0.845030
C	1.442372	0.151778	-0.299688
C	5.686085	-1.262024	-1.843056
C	0.556431	1.191107	-0.381334
C	0.944132	-1.179734	-0.291444
C	-0.907495	0.975665	-0.614692
C	-0.430208	-1.442860	-0.246747
C	-1.309649	-0.408071	-0.306730
C	-1.821252	1.982943	0.075212
C	-3.532712	0.257842	0.104466
C	-3.146869	1.545971	0.331025
C	-4.896492	-0.248923	0.226341
C	-5.988278	0.628417	0.205777
C	-5.130956	-1.620843	0.371847
C	-7.277799	0.152164	0.340172
C	-6.419728	-2.104208	0.510221
C	-7.492294	-1.216025	0.496820
H	3.038844	-0.794749	1.694691
H	5.065172	1.434670	1.271128
H	3.154497	1.440975	-0.385172
H	5.316670	-1.570630	0.876629
H	5.303216	0.718015	-1.142214
H	6.763879	-1.122359	-1.846307
H	5.297055	-1.034034	-2.836167
H	1.750950	0.945698	2.311420
H	4.833716	0.736144	3.445260
H	7.222752	-0.380433	1.534751
H	-0.782897	-2.459076	-0.138359
H	4.509848	-2.814141	-1.667044
H	0.215581	3.038969	-0.155782
H	2.681519	-1.948717	-0.454110
H	-3.856761	2.269728	0.699347
H	-5.834373	1.688668	0.064565
H	-4.302106	-2.312974	0.390777
H	-8.125077	0.823098	0.317937
H	-6.604005	-3.163779	0.631931
H	-8.803976	-2.599784	0.731336
O	-1.195535	1.079283	-2.094327
O	-0.968727	2.404844	-2.552897
H	-1.857946	2.787385	-2.568764

ENERGIES
SCF ENERGY: -1715.78912666
SUM OF ELECTRONIC AND ZERO-POINT ENERGIES: -1715.373376
SUM OF ELECTRONIC AND THERMAL ENERGIES: -1715.343180
SUM OF ELECTRONIC AND THERMAL ENTHALPIES: -1715.342236
SUM OF ELECTRONIC AND THERMAL FREE ENERGIES: -1715.435130

==> ISOVITEXIN/WATER/RAF/C4' <==
54
XYZ-COORDINATES + ENERGIES

O	4.064067	-0.776211	-0.859980
O	3.542946	1.895186	1.604519
O	6.134275	0.914666	2.279240
O	7.527438	-0.289233	0.124550
O	5.728850	-3.090622	-0.923717
O	1.592937	2.379424	-1.051381
O	2.216146	-2.072267	0.538185
O	-2.119105	-0.388260	0.147657
O	-0.849329	3.204273	-1.266066
O	-7.956874	-0.528109	1.602497
C	4.038740	0.670470	1.082433
C	5.545568	0.796080	0.988359
C	3.458418	0.403975	-0.313730
C	6.129796	-0.441232	0.336786
C	5.482943	-0.662201	-1.023823
C	1.971104	0.186765	-0.259293
C	5.949754	-1.924941	-1.714341
C	1.097941	1.209432	-0.603484
C	1.422085	-1.026901	0.201010
C	-0.301857	1.047505	-0.478880
C	0.053841	-1.217154	0.338785
C	-0.782954	-0.173206	0.000452
C	-1.233750	2.094816	-0.828635
C	-3.022438	0.577388	-0.167647
C	-2.623810	1.789789	-0.645220
C	-4.385112	0.167392	0.059841
C	-5.476560	1.054027	-0.211466
C	-4.665426	-1.134001	0.580063
C	-6.753743	0.680110	-0.007462
C	-5.932273	-1.547054	0.786422
C	-7.113011	-0.682484	0.486305
H	3.786896	-0.158682	1.749134
H	5.794118	1.673211	0.381942
H	3.689449	1.253454	-0.958132
H	5.933976	-1.305914	0.976264
H	5.698567	0.194552	-1.670718
H	7.019532	-1.860081	-1.894240
H	5.434725	-2.012401	-2.671824
H	2.644674	1.757605	1.923416
H	5.824076	1.736089	2.674758
H	7.935555	-0.102953	0.977175
H	-0.340661	-2.158694	0.690342
H	4.780304	-3.243283	-0.867527
H	0.819335	2.965032	-1.242852
H	3.113162	-1.917173	0.188855
H	-3.343109	2.551637	-0.896983
H	-5.282751	2.050878	-0.577813
H	-3.846762	-1.794859	0.820052
H	-7.572685	1.358490	-0.196748
H	-6.149214	-2.528604	1.187989

H	-8.094395	-1.393621	2.010523
O	-7.815417	-1.431888	-0.531899
O	-8.987534	-0.728369	-0.914563
H	-9.627514	-0.984868	-0.234809

ENERGIES
SCF ENERGY: -1715.81489025
SUM OF ELECTRONIC AND ZERO-POINT ENERGIES: -1715.398694
SUM OF ELECTRONIC AND THERMAL ENERGIES: -1715.368439
SUM OF ELECTRONIC AND THERMAL ENTHALPIES: -1715.367495
SUM OF ELECTRONIC AND THERMAL FREE ENERGIES: -1715.460632

==> ISOVITEXIN/WATER/RAF/C4'/TS <==
54
XYZ-COORDINATES + ENERGIES

O	3.993925	-0.863894	-0.828629
O	3.567491	2.004645	1.423091
O	6.164301	1.030966	2.122279
O	7.484222	-0.364167	0.047975
O	5.616448	-3.201363	-0.754092
O	1.569801	2.317921	-1.187573
O	2.155410	-2.024701	0.693413
O	-2.156352	-0.277666	0.309014
O	-0.860510	3.190870	-1.357008
O	-8.308819	-0.662698	1.240567
C	4.034404	0.733419	0.994112
C	5.540846	0.823602	0.858923
C	3.418447	0.364900	-0.363519
C	6.088616	-0.472428	0.295588
C	5.410179	-0.785057	-1.030982
C	1.929353	0.177724	-0.255752
C	5.844118	-2.103005	-1.633647
C	1.066136	1.192910	-0.644332
C	1.371884	-0.989624	0.304654
C	-0.331708	1.071789	-0.464352
C	0.004919	-1.141027	0.493121
C	-0.820318	-0.103477	0.109697
C	-1.252556	2.118400	-0.843159
C	-3.043900	0.685697	-0.037403
C	-2.642558	1.856304	-0.592107
C	-4.420774	0.306171	0.261145
C	-5.500168	1.113782	-0.153389
C	-4.679866	-0.874470	0.997007
C	-6.789026	0.740500	0.107656
C	-5.959620	-1.251499	1.287268
C	-7.057573	-0.514024	0.741537
H	3.783742	-0.034452	1.730861
H	5.790984	1.644941	0.179789
H	3.645649	1.157070	-1.078437
H	5.889411	-1.280657	1.004383
H	5.623989	0.016749	-1.745160
H	6.911665	-2.070667	-1.834460
H	5.311555	-2.252284	-2.573789
H	2.666837	1.910828	1.751255
H	5.952207	1.920702	2.422487
H	7.903689	-0.077756	0.866903
H	-0.397911	-2.047469	0.919446
H	4.665943	-3.325039	-0.668960
H	0.803274	2.909056	-1.389578
H	3.047626	-1.909341	0.317709
H	-3.354989	2.621381	-0.854756
H	-5.325883	2.038807	-0.681481
H	-3.856532	-1.468071	1.363264
H	-7.628000	1.344578	-0.206683
H	-6.169538	-2.138319	1.870281
H	-8.387555	-1.510802	1.696896
O	-7.201143	-1.574914	-0.857017
O	-8.409062	-1.250269	-1.444626
H	-9.066845	-1.750626	-0.941519

ENERGIES
SCF ENERGY: -1715.79327227
SUM OF ELECTRONIC AND ZERO-POINT ENERGIES: -1715.379482
SUM OF ELECTRONIC AND THERMAL ENERGIES: -1715.349054
SUM OF ELECTRONIC AND THERMAL ENTHALPIES: -1715.348110
SUM OF ELECTRONIC AND THERMAL FREE ENERGIES: -1715.442337

==> ISOVITEXIN/WATER/RAF/C5 <==
54
XYZ-COORDINATES + ENERGIES

O	3.486550	-0.710220	-0.779735
O	2.833491	1.479551	2.102271
O	5.391125	0.412925	2.690186
O	6.896156	-0.371598	0.423110
O	5.190181	-2.942627	-1.213623
O	0.891866	2.417336	-0.116205
O	1.602030	-2.266858	0.337474
O	-2.744265	-0.613194	0.090147
O	-1.620743	3.253326	-0.349925
O	-8.910493	-1.621819	0.551575
C	3.350415	0.370467	1.384089
C	4.859751	0.519698	1.373925
C	2.829486	0.346333	-0.064883
C	5.493832	-0.573618	0.538701
C	4.907342	-0.544167	-0.865031
C	1.354083	0.078847	-0.126235
C	5.423984	-1.647858	-1.761515
C	0.453041	1.187963	-0.595098
C	0.828894	-1.175806	0.074714
C	-1.011310	0.967502	-0.308071
C	-0.554520	-1.405882	0.050773
C	-1.437596	-0.308390	-0.080241
C	-1.964246	2.052596	-0.270840
C	-3.686445	0.359937	0.036276
C	-3.339416	1.657147	-0.133672
C	-5.045089	-0.166057	0.180126
C	-6.103310	0.673059	0.547146
C	-5.302645	-1.519664	-0.055555
C	-7.387330	0.175512	0.667382
C	-6.586956	-2.023494	0.059224
C	-7.628591	-1.174232	0.420669
H	3.084788	-0.566014	1.881110
H	5.117650	1.493356	0.944578
H	3.061227	1.301342	-0.540344
H	5.285093	-1.542768	0.999475
H	5.126832	0.422048	-1.330839
H	6.498828	-1.540527	-1.880934
H	4.948576	-1.556915	-2.739068
H	1.904834	1.322170	2.303603
H	5.060196	1.154332	3.208480
H	7.260715	-0.328809	1.313932
H	-0.948207	-2.399585	0.201496
H	4.240345	-3.097286	-1.207088
H	0.139063	3.039207	-0.205662
H	2.516564	-2.076724	0.065236
H	-4.097550	2.423431	-0.181084
H	-5.927493	1.717972	0.760310
H	-4.499588	-2.182673	-0.342294
H	-8.207210	0.816805	0.959215
H	-6.789024	-3.069607	-0.131306
H	-8.950726	-2.567955	0.366993
O	0.679616	1.111656	-2.036039
O	-0.036135	2.156072	-2.681052
H	-0.820199	1.702452	-3.022571

ENERGIES
SCF ENERGY: -1715.80841460
SUM OF ELECTRONIC AND ZERO-POINT ENERGIES: -1715.392159
SUM OF ELECTRONIC AND THERMAL ENERGIES: -1715.362121
SUM OF ELECTRONIC AND THERMAL ENTHALPIES: -1715.361176
SUM OF ELECTRONIC AND THERMAL FREE ENERGIES: -1715.453247

==> ISOVITEXIN/WATER/RAF/C5' <==
54
XYZ-COORDINATES + ENERGIES

O	-4.099143	0.820417	-0.675086
O	-3.388868	-2.161094	1.345523
O	-5.994873	-1.394852	2.240550
O	-7.503504	0.046285	0.325553
O	-5.834036	3.045482	-0.368996
O	-1.512287	-2.158363	-1.386287
O	-2.260190	1.990415	0.844941
O	2.124088	0.617136	0.008277
O	0.950403	-2.812937	-1.828285
O	8.317292	1.578858	0.176675
C	-3.949061	-0.893213	1.032287

C	-5.453316	-1.065703	0.965051
C	-3.427665	-0.397244	-0.323498
C	-6.106407	0.227020	0.517559
C	-5.516339	0.667188	-0.815303
C	-1.949859	-0.117904	-0.281947
C	-6.054452	1.993637	-1.305540
C	-1.048415	-1.036614	-0.801604
C	-1.437207	1.047774	0.322271
C	0.346725	-0.817993	-0.721500
C	-0.074434	1.292170	0.419589
C	0.790804	0.352697	-0.103736
C	1.306998	-1.753811	-1.263717
C	3.038068	-0.241295	-0.496968
C	2.688156	-1.384763	-1.125495
C	4.417452	0.227346	-0.287106
C	5.432023	-0.680680	-0.179979
C	4.669699	1.621944	-0.215979
C	6.841472	-0.265321	0.033358
C	5.983157	2.092176	-0.065367
C	7.021788	1.213302	0.026671
H	-3.704437	-0.165049	1.810140
H	-5.690511	-1.854996	0.244419
H	-3.644054	-1.153937	-1.079104
H	-5.921832	0.998918	1.269544
H	-5.719865	-0.098042	-1.571370
H	-7.128557	1.914171	-1.450682
H	-5.585059	2.231433	-2.260994
H	-2.481593	-2.036583	1.644046
H	-5.693742	-2.278262	2.477517
H	-7.866766	-0.319227	1.139587
H	0.294568	2.195537	0.881648
H	-4.888685	3.222626	-0.333317
H	-0.723909	-2.671583	-1.691644
H	-3.167706	1.838396	0.523900
H	3.444756	-2.027733	-1.548052
H	5.243597	-1.743963	-0.205642
H	3.854399	2.322818	-0.298136
H	6.183822	3.155476	-0.030872
H	8.395061	2.539786	0.242715
H	7.518721	-0.746065	-0.677619
O	7.221920	-0.789830	1.349875
O	8.627746	-0.994498	1.391530
H	8.978300	-0.104128	1.545477

ENERGIES
SCF ENERGY: -1715.80722131
SUM OF ELECTRONIC AND ZERO-POINT ENERGIES: -1715.391320
SUM OF ELECTRONIC AND THERMAL ENERGIES: -1715.361131
SUM OF ELECTRONIC AND THERMAL ENTHALPIES: -1715.360187
SUM OF ELECTRONIC AND THERMAL FREE ENERGIES: -1715.453154

==> ISOVITEXIN/WATER/RAF/C6 <==
54
XYZ-COORDINATES + ENERGIES

O	-2.854559	0.801402	-0.658638
O	-3.971940	-2.217561	1.126393
O	-6.269071	-0.552726	1.178206
O	-6.436527	1.262467	-0.965799
O	-3.726712	3.475311	-0.830254
O	-0.886426	-2.161064	-1.448348
O	-1.589638	1.503715	1.574597
O	2.779613	0.306778	0.367575
O	1.545849	-2.568657	-2.220561
O	8.960196	1.317550	0.525695
C	-3.884958	-0.826006	0.841971
C	-5.221489	-0.454797	0.219030
C	-2.761130	-0.534749	-0.163826
C	-5.214931	0.965737	-0.302755
C	-4.088753	1.112855	-1.313156
C	-1.318354	-0.654452	0.386422
C	-3.941558	2.515048	-1.861826
C	-0.379125	-1.242957	-0.615662
C	-0.754772	0.625329	0.955454
C	0.987971	-0.978983	-0.572748
C	0.579286	0.865589	0.987757
C	1.455329	0.029428	0.269638
C	1.933878	-1.695564	-1.409367
C	3.684740	-0.368366	-0.375341
C	3.308262	-1.336085	-1.250971
C	5.056772	0.077418	-0.128057
C	6.144489	-0.725516	-0.490536
C	5.299743	1.316367	0.473050
C	7.440155	-0.300009	-0.267374
C	6.595270	1.751023	0.695640
C	7.664815	0.940883	0.324593
H	-3.748063	-0.251917	1.760198
H	-5.418119	-1.134823	-0.615874
H	-2.883760	-1.234808	-0.990799
H	-5.051881	1.659107	0.526994
H	-4.246706	0.417196	-2.143383
H	-4.854996	2.798017	-2.378252
H	-3.114089	2.529405	-2.572487
H	-3.134418	-2.502404	1.509851
H	-6.396577	-1.482441	1.393777
H	-7.155185	1.063861	-0.355210
H	0.964304	1.738313	1.496243
H	-2.864888	3.304691	-0.436002
H	-0.117549	-2.529895	-1.958222
H	-2.417905	1.550469	1.070286
H	4.043408	-1.835372	-1.862285
H	5.984850	-1.698913	-0.931793
H	4.473949	1.951578	0.758031
H	8.283759	-0.920174	-0.536394
H	6.784622	2.713324	1.153649
H	8.990898	2.181942	0.952418
O	-1.280221	-1.711248	1.452707
O	-1.629450	-1.180440	2.725788
H	-0.764414	-1.014545	3.128733

ENERGIES
SCF ENERGY: -1715.79463458
SUM OF ELECTRONIC AND ZERO-POINT ENERGIES: -1715.379218
SUM OF ELECTRONIC AND THERMAL ENERGIES: -1715.349027
SUM OF ELECTRONIC AND THERMAL ENTHALPIES: -1715.348083
SUM OF ELECTRONIC AND THERMAL FREE ENERGIES: -1715.440601

==> ISOVITEXIN/WATER/RAF/C6' <==
54
XYZ-COORDINATES + ENERGIES

O	-3.933970	0.521095	-0.982237
O	-3.294599	-1.552651	1.979678
O	-5.973129	-0.647188	2.404831
O	-7.380395	-0.007474	0.038338
O	-5.739977	2.644775	-1.544901
O	-1.244221	-2.418151	-0.487943
O	-2.221393	2.206716	0.148110
O	2.231606	0.803420	0.147464
O	1.247435	-3.089492	-0.493537
O	8.401991	1.891917	0.891684
C	-3.861673	-0.498325	1.214236
C	-5.353387	-0.749783	1.126852
C	-3.263329	-0.476463	-0.199023
C	-6.002328	0.285173	0.229650
C	-5.335924	0.271620	-1.138962
C	-1.797115	-0.142886	-0.171518
C	-5.868614	1.329922	-2.080070
C	-0.845640	-1.147167	-0.287144
C	-1.347008	1.176177	0.036922
C	0.535493	-0.860300	-0.185172
C	0.000739	1.491225	0.144006
C	0.917394	0.464803	0.035511
C	1.546190	-1.887776	-0.296346
C	3.202985	-0.139943	0.046827
C	2.904919	-1.449905	-0.167866
C	4.534627	0.396008	0.202917
C	5.697689	-0.539044	0.057471
C	4.742557	1.732129	0.471427
C	7.020951	0.061052	0.382073
C	6.022362	2.242996	0.674107
C	7.154261	1.380197	0.642252
H	-3.687041	0.464063	1.702463
H	-5.524736	-1.746621	0.707843
H	-3.417853	-1.453189	-0.660167
H	-5.882144	1.273305	0.681874
H	-5.473576	-0.712393	-1.598940
H	-6.927689	1.157060	-2.251992

H -5.339039 1.253003 -3.030543
H -2.417145 -1.286593 2.274768
H -5.664553 -1.378390 2.950126
H -7.785427 -0.086765 0.909113
H 0.319697 2.511267 0.297568
H -4.803823 2.865544 -1.511233
H -0.423161 -2.969462 -0.538286
H -3.097916 1.916925 -0.164807
H 3.689742 -2.184304 -0.255324
H 5.541482 -1.447121 0.643923
H 3.899341 2.402401 0.545776
H 7.886880 -0.586363 0.368462
H 6.163739 3.295791 0.877010
H 8.335978 2.832090 1.093152
O 5.667128 -0.959326 -1.344743
O 6.448944 -2.137429 -1.467151
H 7.301012 -1.808219 -1.786430

ENERGIES
SCF ENERGY: -1715.80980547
SUM OF ELECTRONIC AND ZERO-POINT ENERGIES: -1715.394477
SUM OF ELECTRONIC AND THERMAL ENERGIES: -1715.363934
SUM OF ELECTRONIC AND THERMAL ENTHALPIES: -1715.362990
SUM OF ELECTRONIC AND THERMAL FREE ENERGIES: -1715.456357

==> ISOVITEXIN/WATER/RAF/C7 <==
54
XYZ-COORDINATES + ENERGIES
O 3.239079 -0.040745 -1.156744
O 3.207728 0.667399 2.442157
O 5.892250 -0.148164 1.995882
O 6.832392 0.208603 -0.662099
O 4.782984 -1.818358 -2.723494
O 0.849544 2.653719 -0.154875
O 1.447024 -1.995056 -0.416866
O -2.838683 -0.403324 0.028816
O -1.580528 3.451068 -0.218617
O -9.041225 -1.203358 -0.309991
C 3.602605 0.063528 1.219626
C 5.060786 0.400202 0.978119
C 2.775568 0.604082 0.048181
C 5.509310 -0.202582 -0.339357
C 4.602668 0.266866 -1.469961
C 1.296754 0.364979 0.144221
C 4.901640 -0.399185 -2.795448
C 0.419300 1.384609 0.011014
C 0.798227 -1.033727 0.369602
C -1.021472 1.167802 0.007033
C -0.671228 -1.222630 0.202485
C -1.500994 -0.136894 0.080451
C -1.964571 2.260531 -0.132238
C -3.735710 0.596293 -0.098562
C -3.344709 1.894738 -0.180956
C -5.114877 0.114104 -0.145661
C -6.185345 0.989888 0.073524
C -5.383351 -1.232234 -0.413908
C -7.488615 0.536684 0.017086
C -6.687153 -1.692187 -0.477055
C -7.739250 -0.805451 -0.262209
H 3.501103 -1.021445 1.279270
H 5.183048 1.487524 0.941273
H 2.957730 1.676223 -0.036428
H 5.459800 -1.291568 -0.260294
H 4.706515 1.350706 -1.586367
H 5.924067 -0.178943 -3.090678
H 4.221109 0.000298 -3.548804
H 2.401366 0.224919 2.733728
H 5.657705 0.270598 2.830786
H 7.410049 -0.066651 0.057946
H -1.068155 -2.225934 0.239908
H 3.866887 -2.029776 -2.515729
H 0.044569 3.231323 -0.209772
H 2.277548 -1.624724 -0.759849
H -4.072910 2.678834 -0.315421
H -6.005763 2.028994 0.308795
H -4.571259 -1.922928 -0.586679
H -8.318834 1.205978 0.193694
H -6.897972 -2.731523 -0.693325
H -9.094885 -2.146374 -0.504918
O 1.111518 -1.260703 1.778454
O 0.805277 -2.601810 2.131822
H -0.018094 -2.506168 2.631388

ENERGIES
SCF ENERGY: -1715.80298304
SUM OF ELECTRONIC AND ZERO-POINT ENERGIES: -1715.387045
SUM OF ELECTRONIC AND THERMAL ENERGIES: -1715.356861
SUM OF ELECTRONIC AND THERMAL ENTHALPIES: -1715.355917
SUM OF ELECTRONIC AND THERMAL FREE ENERGIES: -1715.448726

==> ISOVITEXIN/WATER/RAF/C8 <==
54
XYZ-COORDINATES + ENERGIES
O 3.444195 -0.446431 -1.097244
O 3.069466 1.731542 1.833413
O 5.705285 0.689649 2.158360
O 6.961749 -0.099196 -0.254339
O 5.100219 -2.690640 -1.672553
O 0.979890 2.689079 -0.547373
O 1.664505 -2.003382 0.116070
O -2.708438 -0.310942 0.015443
O -1.490746 3.479807 -0.723598
O -8.907329 -1.227414 -0.078418
C 3.538610 0.630921 1.066878
C 5.035984 0.793478 0.905825
C 2.874486 0.611629 -0.315112
C 5.579845 -0.302249 0.010780
C 4.849722 -0.286955 -1.325466
C 1.391315 0.367866 -0.203118
C 5.274552 -1.401908 -2.256023
C 0.493742 1.448696 -0.331679
C 0.895820 -0.890479 0.042116
C -0.910948 1.236647 -0.262107
C -0.532579 -1.176444 0.333865
C -1.387647 -0.022368 -0.004075
C -1.869129 2.308344 -0.502373
C -3.623741 0.651030 -0.225829
C -3.247594 1.930312 -0.483054
C -4.997754 0.149627 -0.182072
C -6.073111 1.031060 -0.023233
C -5.253604 -1.219761 -0.300473
C -7.371731 0.559350 0.009650
C -6.552128 -1.698437 -0.273611
C -7.610448 -0.807237 -0.117789
H 3.331480 -0.310415 1.583268
H 5.244427 1.767003 0.450201
H 3.064112 1.563743 -0.812970
H 5.423879 -1.268710 0.497469
H 5.023180 0.673247 -1.822270
H 6.331784 -1.294735 -2.483910
H 4.703725 -1.323593 -3.182148
H 2.186117 1.529682 2.160470
H 5.439696 1.437599 2.703893
H 7.417413 -0.046091 0.592937
H -0.871409 -2.089929 -0.157318
H 4.156349 -2.863475 -1.600254
H 0.200552 3.286048 -0.654507
H 2.553710 -1.800466 -0.229755
H -3.988788 2.683722 -0.698750
H -5.899910 2.091194 0.095905
H -4.436922 -1.915466 -0.426456
H -8.205584 1.234414 0.142060
H -6.754425 -2.757063 -0.373633
H -8.948398 -2.187346 -0.165853
O -0.722100 -1.408603 1.778021
O -0.523145 -2.785250 2.062570
H 0.418644 -2.833861 2.280215

ENERGIES
SCF ENERGY: -1715.80884884
SUM OF ELECTRONIC AND ZERO-POINT ENERGIES: -1715.391933
SUM OF ELECTRONIC AND THERMAL ENERGIES: -1715.361747
SUM OF ELECTRONIC AND THERMAL ENTHALPIES: -1715.360803
SUM OF ELECTRONIC AND THERMAL FREE ENERGIES: -1715.453725

==> ISOVITEXIN/WATER/RAF/C8a <==

54
XYZ-COORDINATES + ENERGIES

O	3.477567	-0.846842	-0.931969
O	3.178257	1.980812	1.391112
O	5.839357	1.042096	1.869113
O	7.016930	-0.286646	-0.336589
O	5.162153	-3.157577	-1.012417
O	1.041938	2.265625	-1.222293
O	1.748953	-2.058280	0.670742
O	-2.571320	-0.456353	0.101675
O	-1.371376	3.146893	-1.352051
O	-8.751463	-1.455044	-0.219695
C	3.633858	0.725978	0.908710
C	5.124179	0.843369	0.655277
C	2.930996	0.369447	-0.412825
C	5.650407	-0.431692	0.026260
C	4.873469	-0.742927	-1.245581
C	1.461188	0.176789	-0.196295
C	5.279555	-2.046477	-1.897074
C	0.546033	1.218905	-0.563700
C	0.939185	-1.000580	0.381264
C	-0.795655	1.130078	-0.288020
C	-0.390324	-1.130788	0.687315
C	-1.312053	0.024427	0.551825
C	-1.743704	2.089870	-0.783147
C	-3.486331	0.478377	-0.283100
C	-3.133404	1.732546	-0.649867
C	-4.854119	-0.041237	-0.272900
C	-5.839398	0.515134	-1.096977
C	-5.195226	-1.098884	0.575160
C	-7.134519	0.034119	-1.068629
C	-6.492379	-1.582365	0.613582
C	-7.460312	-1.014656	-0.210487
H	3.450955	-0.060390	1.644792
H	5.304333	1.680974	-0.026300
H	3.103276	1.174221	-1.128404
H	5.529496	-1.253849	0.736454
H	5.014211	0.069990	-1.965248
H	6.322998	-1.985106	-2.194761
H	4.666772	-2.200904	-2.786068
H	2.358563	1.851979	1.879782
H	5.619723	1.916112	2.208284
H	7.498930	-0.001247	0.447409
H	-0.767076	-2.043716	1.122393
H	4.227712	-3.311622	-0.841786
H	0.273389	2.870738	-1.408706
H	2.598578	-1.944649	0.207710
H	-3.886690	2.450404	-0.934676
H	-5.588834	1.314114	-1.780678
H	-4.446830	-1.535856	1.220604
H	-7.898341	0.450622	-1.710359
H	-6.760477	-2.394716	1.276749
H	-8.859444	-2.182994	0.403796
O	-1.531517	0.641963	1.844244
O	-2.036349	-0.327987	2.750781
H	-2.994963	-0.269960	2.623050

ENERGIES
SCF ENERGY: -1715.80616780
SUM OF ELECTRONIC AND ZERO-POINT ENERGIES: -1715.389937
SUM OF ELECTRONIC AND THERMAL ENERGIES: -1715.360155
SUM OF ELECTRONIC AND THERMAL ENTHALPIES: -1715.359211
SUM OF ELECTRONIC AND THERMAL FREE ENERGIES: -1715.450451

==> VITEXIN/LIPID <==
51
XYZ-COORDINATES + ENERGIES

O	0.103332	-1.665342	0.988878
O	-0.962511	-2.023510	-2.464803
O	1.744902	-2.788594	-2.604642
O	2.885251	-3.787678	-0.151552
O	-0.404789	0.916900	-0.060850
O	1.055010	-2.381196	3.476998
O	-3.516116	-2.585931	0.090616
O	-5.063737	1.884016	0.205956
O	-3.290552	3.747911	0.146920
O	5.503036	2.830359	-0.062620
C	-0.045036	-1.809515	-1.408005
C	1.015374	-2.892732	-1.391493
C	-0.817739	-1.864744	-0.088808
C	1.919118	-2.742322	-0.181229
C	1.071696	-2.707951	1.086641
C	-1.928265	-0.857408	0.005245
C	1.884224	-2.408637	2.327298
C	-1.700430	0.514722	0.010678
C	-3.265768	-1.257053	0.079971
C	-2.720284	1.465050	0.077795
C	-4.321455	-0.346553	0.144236
C	-4.056968	1.009723	0.142507
C	-2.400573	2.880302	0.091381
C	-0.068348	2.225413	-0.022352
C	-0.998243	3.203423	0.057656
C	1.385339	2.408150	-0.054647
C	1.953816	3.623138	-0.439709
C	2.224877	1.350255	0.311757
C	3.328506	3.785054	-0.447581
C	3.598568	1.505769	0.309583
C	4.152251	2.725841	-0.070954
H	0.445607	-0.838687	-1.509741
H	0.512246	-3.868393	-1.339880
H	-1.255607	-2.861422	-0.001587
H	2.486250	-1.814184	-0.270814
H	0.552592	-3.668975	1.210742
H	2.397760	-1.450650	2.195312
H	2.629490	-3.185277	2.481530
H	-0.446769	-2.136158	-3.269730
H	2.490482	-3.395756	-2.556799
H	2.429386	-4.621408	0.010567
H	-5.346658	-0.689322	0.197685
H	0.329782	-1.779688	3.281028
H	-0.703790	4.239341	0.121505
H	-4.465733	-2.740972	0.133346
H	-4.652827	2.785764	0.196352
H	1.327096	4.444977	-0.756282
H	1.787881	0.408013	0.612622
H	3.764630	4.727312	-0.755687
H	4.254992	0.696806	0.600025
H	5.768633	3.713640	-0.339209

ENERGIES
SCF ENERGY: -1564.85502924
SUM OF ELECTRONIC AND ZERO-POINT ENERGIES: -1564.455744
SUM OF ELECTRONIC AND THERMAL ENERGIES: -1564.428389
SUM OF ELECTRONIC AND THERMAL ENTHALPIES: -1564.427445
SUM OF ELECTRONIC AND THERMAL FREE ENERGIES: -1564.512907

==> VITEXIN/LIPID/AIP <==
51
XYZ-COORDINATES + ENERGIES

O	0.121009	-1.651143	0.995546
O	-1.046071	-2.054647	-2.414883
O	1.654451	-2.848947	-2.613602
O	2.839811	-3.839015	-0.170045
O	-0.372498	0.904482	-0.040146
O	1.112887	-2.339547	3.475728
O	-3.529570	-2.539414	0.148571
O	-5.037045	1.929788	0.209051
O	-3.212838	3.795024	0.087234
O	5.461634	2.824246	-0.096077
C	-0.095236	-1.844928	-1.391105
C	0.952080	-2.941294	-1.384180
C	-0.830151	-1.864069	-0.049762
C	1.884303	-2.787516	-0.194651
C	1.068349	-2.717659	1.092634
C	-1.925008	-0.837001	0.044552
C	1.914272	-2.416609	2.310186
C	-1.683172	0.521766	0.034738
C	-3.277175	-1.221353	0.125022
C	-2.692932	1.492775	0.088970
C	-4.320098	-0.299993	0.181657
C	-4.039219	1.052366	0.160617
C	-2.367759	2.894303	0.055867
C	-0.012487	2.195155	-0.055502
C	-0.945224	3.205330	-0.019391
C	1.402063	2.395242	-0.065289
C	1.965708	3.675764	-0.332825

C	2.259397	1.286092	0.186743
C	3.316351	3.840707	-0.352148
C	3.609709	1.446168	0.181048
C	4.152647	2.727679	-0.094418
H	0.409574	-0.883821	-1.522322
H	0.440377	-3.910683	-1.313408
H	-1.280177	-2.851290	0.070251
H	2.459305	-1.866646	-0.313609
H	0.527902	-3.661915	1.243751
H	2.455097	-1.477949	2.147127
H	2.639180	-3.212076	2.465139
H	-0.560476	-2.237433	-3.226221
H	2.358789	-3.505428	-2.608560
H	2.384383	-4.667661	0.018583
H	-5.349675	-0.628139	0.239296
H	0.388075	-1.738051	3.279304
H	-0.652197	4.243085	-0.019946
H	-4.479362	-2.701670	0.194164
H	-4.651301	2.833118	0.178386
H	1.329764	4.519629	-0.553513
H	1.819189	0.324099	0.407642
H	3.759613	4.803451	-0.570280
H	4.287399	0.628918	0.383886
H	5.761891	3.726292	-0.287279

ENERGIES
SCF ENERGY: -1564.60841257
SUM OF ELECTRONIC AND ZERO-POINT ENERGIES: -1564.208639
SUM OF ELECTRONIC AND THERMAL ENERGIES: -1564.181591
SUM OF ELECTRONIC AND THERMAL ENTHALPIES: -1564.180646
SUM OF ELECTRONIC AND THERMAL FREE ENERGIES: -1564.265247

==> VITEXIN/LIPID/BDE/C4' <==
50
XYZ-COORDINATES + ENERGIES

O	0.283314	-1.614882	0.987332
O	-0.798588	-2.103853	-2.442989
O	1.957046	-2.666020	-2.605786
O	3.177682	-3.581990	-0.151786
O	-0.443553	0.899004	-0.068084
O	1.309018	-2.238579	3.479575
O	-3.264483	-2.833444	0.143888
O	-5.168703	1.493332	0.212107
O	-3.539369	3.500713	0.118201
O	5.202352	3.337332	-0.080697
C	0.113028	-1.809854	-1.401169
C	1.249131	-2.813084	-1.384513
C	-0.635634	-1.903713	-0.070981
C	2.153319	-2.594233	-0.184146
C	1.319295	-2.591085	1.093150
C	-1.821626	-0.986326	0.022873
C	2.123201	-2.218327	2.319179
C	-1.703906	0.397746	0.012679
C	-3.123203	-1.490910	0.112693
C	-2.797370	1.265777	0.076678
C	-4.248306	-0.667464	0.172353
C	-4.093737	0.704832	0.153728
C	-2.593419	2.699071	0.068740
C	-0.211544	2.230904	-0.052065
C	-1.215665	3.135255	0.012967
C	1.214091	2.534834	-0.077708
C	1.672927	3.841739	-0.379340
C	2.143471	1.503739	0.208032
C	3.005942	4.118936	-0.385491
C	3.482130	1.762304	0.213224
C	3.987428	3.090976	-0.083356
H	0.529836	-0.807048	-1.521794
H	0.815636	-3.820772	-1.321579
H	-0.992635	-2.929685	0.038731
H	2.666129	-1.636722	-0.291001
H	0.865277	-3.581059	1.240788
H	2.564220	-1.227069	2.171046
H	2.924880	-2.937031	2.471295
H	-0.285362	-2.198131	-3.251999
H	2.736199	-3.230432	-2.569904
H	2.772253	-4.438628	0.024253
H	-5.242247	-1.091147	0.234591
H	0.541639	-1.689547	3.290845
H	-1.013250	4.193604	0.049590
H	-4.197972	-3.066407	0.193759
H	-4.842574	2.425210	0.189582
H	0.964704	4.619092	-0.627269
H	1.771742	0.515450	0.440174
H	3.377659	5.106235	-0.622090
H	4.208824	0.994460	0.441736

ENERGIES
SCF ENERGY: -1564.20400730
SUM OF ELECTRONIC AND ZERO-POINT ENERGIES: -1563.817976
SUM OF ELECTRONIC AND THERMAL ENERGIES: -1563.790831
SUM OF ELECTRONIC AND THERMAL ENTHALPIES: -1563.789887
SUM OF ELECTRONIC AND THERMAL FREE ENERGIES: -1563.875754

==> VITEXIN/LIPID/BDE/C4'/TS <==
54
XYZ-COORDINATES + ENERGIES

O	0.670546	1.714094	1.042389
O	1.429278	1.846973	-2.506724
O	-1.132863	3.013322	-2.408130
O	-1.851404	4.226686	0.109493
O	0.655941	-0.927843	0.041250
O	0.110439	2.623837	3.585707
O	4.285075	2.024284	-0.231529
O	5.107649	-2.630346	-0.124059
O	3.054742	-4.188536	0.044431
O	-5.414627	-1.966217	0.709580
C	0.600025	1.794842	-1.360489
C	-0.277257	3.028358	-1.275887
C	1.499369	1.747712	-0.123937
C	-1.065765	3.043010	0.021498
C	-0.107952	2.899697	1.199902
C	2.440397	0.576188	-0.105720
C	-0.821898	2.757494	2.526003
C	2.000065	-0.740080	-0.032011
C	3.825597	0.755288	-0.174824
C	2.857339	-1.843215	-0.034924
C	4.723600	-0.312659	-0.184074
C	4.249612	-1.608158	-0.116046
C	2.321031	-3.186791	0.049741
C	0.123116	-2.164309	0.146109
C	0.883619	-3.280074	0.155178
C	-1.336024	-2.115406	0.273048
C	-2.119028	-3.252202	0.006135
C	-1.950371	-0.918522	0.666236
C	-3.486094	-3.195370	0.130666
C	-3.319512	-0.856608	0.809615
C	-4.113693	-1.998149	0.548569
H	-0.041439	0.910794	-1.385581
H	0.370052	3.915771	-1.307769
H	2.096295	2.662077	-0.109507
H	-1.775223	2.213485	0.019275
H	0.564894	3.768015	1.237928
H	-1.488332	1.889811	2.483661
H	-1.417982	3.644714	2.725664
H	0.860712	2.035251	-3.260376
H	-1.777669	3.720785	-2.304772
H	-1.258779	4.982574	0.191116
H	5.789432	-0.135642	-0.242582
H	0.711079	1.913737	3.338678
H	0.436275	-4.254987	0.267709
H	5.246801	2.025123	-0.286002
H	4.567379	-3.456199	-0.065825
H	-1.650823	-4.168599	-0.323801
H	-1.338365	-0.051278	0.870701
H	-4.107516	-4.054993	-0.078620
H	-3.811148	0.053593	1.125457
H	-5.882269	-1.267108	-0.056997
O	-6.132680	-0.642475	-1.093163
O	-4.893144	-0.361163	-1.579678
H	-4.762029	-0.994137	-2.303609

ENERGIES
SCF ENERGY: -1715.76788561
SUM OF ELECTRONIC AND ZERO-POINT ENERGIES: -1715.357360
SUM OF ELECTRONIC AND THERMAL ENERGIES: -1715.326906
SUM OF ELECTRONIC AND THERMAL ENTHALPIES: -1715.325961

SUM OF ELECTRONIC AND THERMAL FREE ENERGIES: -1715.420487

==> VITEXIN/LIPID/BDE/C5 <==
50
XYZ-COORDINATES + ENERGIES

O	0.029512	-1.591681	0.993516
O	-1.101509	-1.920382	-2.438437
O	1.593031	-2.762603	-2.621533
O	2.733545	-3.796133	-0.182235
O	-0.427482	0.941548	-0.038580
O	1.004716	-2.346206	3.474515
O	-3.574400	-2.513154	0.085696
O	-5.066792	1.941009	0.243055
O	-3.102343	3.968821	0.104783
O	5.594536	2.499354	-0.073581
C	-0.152350	-1.737222	-1.405974
C	0.876387	-2.850767	-1.401347
C	-0.900391	-1.780305	-0.070602
C	1.797766	-2.724613	-0.199829
C	0.970447	-2.665218	1.081507
C	-2.002151	-0.761832	0.026362
C	1.810282	-2.389287	2.309219
C	-1.741819	0.641758	0.035477
C	-3.342911	-1.179253	0.088115
C	-2.714966	1.610252	0.097125
C	-4.360677	-0.261522	0.154934
C	-4.112325	1.165370	0.167968
C	-2.319039	3.033621	0.085310
C	-0.003827	2.231186	-0.011054
C	-0.879623	3.248996	0.062071
C	1.458492	2.323352	-0.050270
C	2.096997	3.497146	-0.452837
C	2.234030	1.222247	0.326749
C	3.479101	3.575725	-0.466386
C	3.615160	1.294306	0.318286
C	4.239423	2.474268	-0.078247
H	0.363965	-0.781297	-1.513841
H	0.347119	-3.812082	-1.344288
H	-1.354172	-2.768718	0.022499
H	2.389299	-1.812684	-0.297349
H	0.422842	-3.608284	1.215220
H	2.348763	-1.446113	2.171956
H	2.536219	-3.187158	2.446032
H	-0.614536	-2.029484	-3.261829
H	2.325560	-3.386346	-2.583896
H	2.257236	-4.618105	-0.018288
H	-5.396513	-0.571501	0.209449
H	0.309276	-1.701483	3.312734
H	-0.527185	4.267820	0.113540
H	-4.522559	-2.682276	0.115580
H	1.518356	4.351539	-0.775772
H	1.741366	0.312978	0.641977
H	3.970888	4.485437	-0.788033
H	4.223234	0.451052	0.616764
H	5.910278	3.362880	-0.359502

ENERGIES
SCF ENERGY: -1564.18265713
SUM OF ELECTRONIC AND ZERO-POINT ENERGIES: -1563.796629
SUM OF ELECTRONIC AND THERMAL ENERGIES: -1563.769149
SUM OF ELECTRONIC AND THERMAL ENTHALPIES: -1563.768205
SUM OF ELECTRONIC AND THERMAL FREE ENERGIES: -1563.854115

==> VITEXIN/LIPID/BDE/C7 <==
50
XYZ-COORDINATES + ENERGIES

O	0.156986	-1.654126	0.995950
O	-0.993817	-2.066299	-2.418894
O	1.722410	-2.815158	-2.618406
O	2.925533	-3.778526	-0.174520
O	-0.427188	0.896813	-0.037517
O	1.190625	-2.328994	3.476805
O	-3.493476	-2.628552	0.159127
O	-5.132658	1.757090	0.190629
O	-3.392050	3.644123	0.124342
O	5.416656	2.983810	-0.112679
C	-0.046702	-1.843189	-1.393445
C	1.022870	-2.917141	-1.388553
C	-0.781264	-1.897125	-0.051425
C	1.952584	-2.740848	-0.199712
C	1.138873	-2.688815	1.090349
C	-1.904831	-0.911427	0.037286
C	1.981843	-2.355491	2.301252
C	-1.712916	0.462373	0.036094
C	-3.282362	-1.406708	0.114873
C	-2.768770	1.369532	0.087071
C	-4.365843	-0.457637	0.151694
C	-4.125248	0.880277	0.144974
C	-2.478975	2.805564	0.082566
C	-0.126140	2.209829	-0.017771
C	-1.091417	3.160432	0.047295
C	1.317173	2.438264	-0.056280
C	1.843303	3.683837	-0.406558
C	2.193929	1.393988	0.263710
C	3.211086	3.886755	-0.430526
C	3.561007	1.591313	0.246369
C	4.071895	2.839829	-0.103170
H	0.434002	-0.868671	-1.510847
H	0.530637	-3.896410	-1.315427
H	-1.214355	-2.893200	0.064304
H	2.511156	-1.810709	-0.319545
H	0.629879	-3.649518	1.248358
H	2.473004	-1.390192	2.139998
H	2.746204	-3.116090	2.441022
H	-0.506193	-2.191214	-3.239678
H	2.466277	-3.425771	-2.591047
H	2.480113	-4.613260	0.010513
H	-5.375862	-0.837968	0.198404
H	0.452844	-1.735710	3.305759
H	-0.831202	4.206271	0.091072
H	-4.737036	2.662015	0.176409
H	1.189457	4.499420	-0.680924
H	1.792455	0.429350	0.541929
H	3.614163	4.851655	-0.711911
H	4.245977	0.794065	0.500574
H	5.653742	3.882835	-0.364201

ENERGIES
SCF ENERGY: -1564.19685949
SUM OF ELECTRONIC AND ZERO-POINT ENERGIES: -1563.811567
SUM OF ELECTRONIC AND THERMAL ENERGIES: -1563.784287
SUM OF ELECTRONIC AND THERMAL ENTHALPIES: -1563.783343
SUM OF ELECTRONIC AND THERMAL FREE ENERGIES: -1563.869716

==> VITEXIN/LIPID/BDE/C7/TS <==
54
XYZ-COORDINATES + ENERGIES

O	-0.260129	-1.539152	0.675758
O	-1.039154	-1.336919	-2.855887
O	1.299816	-2.920193	-2.837419
O	1.767825	-4.406262	-0.405461
O	0.179255	1.118125	-0.173682
O	-0.016572	-2.707808	3.144186
O	-3.923372	-1.153101	-0.517043
O	-3.942376	3.526583	-0.161619
O	-1.669871	4.707471	0.048896
O	6.348036	0.998093	0.477885
C	-0.212685	-1.492791	-1.718864
C	0.443696	-2.861419	-1.707414
C	-1.089633	-1.372460	-0.469968
C	1.207126	-3.098430	-0.412997
C	0.272893	-2.860129	0.767116
C	-1.826984	-0.069438	-0.369180
C	0.940312	-2.930561	2.121102
C	-1.175797	1.155202	-0.232053
C	-3.250623	-0.034040	-0.414057
C	-1.852508	2.367591	-0.159795
C	-3.957998	1.184203	-0.338878
C	-3.278000	2.371846	-0.224839
C	-1.096180	3.608026	-0.008649
C	0.908555	2.239207	0.003060
C	0.327474	3.460392	0.092387
C	2.337968	1.942926	0.103457
C	3.302213	2.931129	-0.106622
C	2.752412	0.644261	0.424208
C	4.648632	2.635297	0.012567

C	4.094871	0.343552	0.550109
C	5.045854	1.340884	0.345014
H	0.568286	-0.729416	-1.696068
H	-0.344887	-3.621597	-1.795195
H	-1.829031	-2.175231	-0.504409
H	2.051370	-2.408989	-0.360608
H	-0.555392	-3.581947	0.735679
H	1.740678	-2.184433	2.166194
H	1.371057	-3.916671	2.279864
H	-0.510542	-1.563104	-3.628129
H	1.794145	-3.745222	-2.794364
H	1.049871	-5.047983	-0.360271
H	-5.037243	1.167825	-0.384150
H	-0.569514	-1.972771	2.855871
H	0.923118	4.342551	0.267116
H	-3.266131	4.244145	-0.075277
H	3.010674	3.934400	-0.384257
H	2.010603	-0.124959	0.589365
H	5.393124	3.402760	-0.160198
H	4.423667	-0.653853	0.808278
H	6.911462	1.762375	0.316643
H	-3.892026	-1.699115	0.500296
O	-3.751576	-1.994482	1.676010
O	-2.874739	-1.054076	2.123626
H	-3.430014	-0.366482	2.527379

ENERGIES

SCF ENERGY: -1715.76559990

SUM OF ELECTRONIC AND ZERO-POINT ENERGIES: -1715.355110

SUM OF ELECTRONIC AND THERMAL ENERGIES: -1715.324849

SUM OF ELECTRONIC AND THERMAL ENTHALPIES: -1715.323905

SUM OF ELECTRONIC AND THERMAL FREE ENERGIES: -1715.417295

==> VITEXIN/LIPID/RAF/C1' <==

54

XYZ-COORDINATES + ENERGIES

O	1.555280	1.075341	1.072971
O	2.705655	0.209061	-2.263170
O	2.041074	2.934105	-2.563015
O	2.109324	4.502139	-0.146422
O	-0.327745	-0.674013	-0.199484
O	1.466312	2.335395	3.514056
O	4.180838	-1.639745	0.638130
O	1.044500	-5.178205	0.556953
O	-1.422313	-4.560048	0.119073
O	-5.957156	1.691958	1.322037
C	1.962387	0.934199	-1.298161
C	2.373948	2.393266	-1.293632
C	2.273290	0.339520	0.076787
C	1.684608	3.142297	-0.167596
C	1.964013	2.438410	1.155779
C	1.932478	-1.118074	0.202973
C	1.191716	3.035751	2.311362
C	0.634992	-1.596803	0.061813
C	2.911626	-2.076277	0.478472
C	0.291929	-2.946463	0.175187
C	2.623090	-3.437020	0.593978
C	1.322549	-3.877055	0.444237
C	-1.086442	-3.368597	0.022236
C	-1.611359	-1.040105	-0.348425
C	-2.035067	-2.310983	-0.246812
C	-2.492357	0.175745	-0.558382
C	-3.868655	-0.181961	-1.013914
C	-2.472321	1.004879	0.688528
C	-4.976871	0.335275	-0.425582
C	-3.606674	1.477308	1.256414
C	-4.873078	1.172140	0.707235
H	0.892419	0.870342	-1.501642
H	3.461003	2.446194	-1.139904
H	3.344722	0.454462	0.255169
H	0.609596	3.165975	-0.351869
H	3.041122	2.477631	1.374062
H	0.122688	3.005758	2.078032
H	1.486553	4.071494	2.462093
H	2.585287	0.656581	-3.106620
H	2.170534	3.887032	-2.521566
H	3.043416	4.525848	0.090119
H	3.408702	-4.151683	0.801110
H	1.342776	1.400461	3.321939
H	-3.080957	-2.553883	-0.354708
H	4.765861	-2.385171	0.810502
H	0.069761	-5.272733	0.419683
H	-3.955632	-0.838906	-1.867947
H	-1.497542	1.219307	1.104227
H	-5.958817	0.095334	-0.817523
H	-3.565674	2.087030	2.149389
H	-6.762539	1.413516	0.872105
O	-1.834248	1.031695	-1.547288
O	-1.577892	0.256613	-2.714770
H	-2.255120	0.578346	-3.324194

ENERGIES

SCF ENERGY: -1715.77195642

SUM OF ELECTRONIC AND ZERO-POINT ENERGIES: -1715.355847

SUM OF ELECTRONIC AND THERMAL ENERGIES: -1715.325440

SUM OF ELECTRONIC AND THERMAL ENTHALPIES: -1715.324496

SUM OF ELECTRONIC AND THERMAL FREE ENERGIES: -1715.418217

==> VITEXIN/LIPID/RAF/C2 <==

54

XYZ-COORDINATES + ENERGIES

O	-0.095846	-1.953869	0.979996
O	-1.369590	-1.952732	-2.421748
O	1.225597	-3.015922	-2.759466
O	2.341014	-4.313339	-0.442542
O	-0.260166	0.727462	0.137761
O	0.843959	-2.972719	3.361826
O	-3.834094	-2.302245	0.276210
O	-4.733209	2.309224	0.746145
O	-2.691906	3.921094	0.744714
O	5.768123	2.091548	0.744627
C	-0.375101	-1.909847	-1.413279
C	0.543181	-3.111823	-1.518790
C	-1.080938	-1.949555	-0.057037
C	1.511767	-3.158930	-0.350779
C	0.728586	-3.116411	0.956735
C	-2.028831	-0.802810	0.160516
C	1.619727	-3.019904	2.175636
C	-1.604171	0.513145	0.208557
C	-3.408923	-1.022027	0.312084
C	-2.483761	1.590504	0.394507
C	-4.318744	0.013458	0.495719
C	-3.860015	1.318799	0.546332
C	-1.969851	2.942708	0.474445
C	0.220206	1.960806	-0.323208
C	-0.569748	3.110788	0.202432
C	1.693271	2.027653	-0.005366
C	2.407088	3.211209	-0.177383
C	2.368845	0.883816	0.422700
C	3.772123	3.253543	0.066211
C	3.730222	0.919166	0.671934
C	4.434487	2.105540	0.489500
H	0.221259	-0.999288	-1.495794
H	-0.075508	-4.020211	-1.501846
H	-1.651313	-2.879566	-0.007064
H	2.184757	-2.301324	-0.404519
H	0.093548	-4.010370	1.036300
H	2.249032	-2.128364	2.087720
H	2.261123	-3.895565	2.240280
H	-0.913466	-2.022067	-3.266353
H	1.898860	-3.703604	-2.781705
H	1.790883	-5.095069	-0.318561
H	-5.375834	-0.186933	0.610632
H	0.201729	-2.265922	3.242807
H	-0.132812	4.091026	0.305432
H	-4.792181	-2.334359	0.372845
H	-4.218338	3.145788	0.803287
H	1.913100	4.109920	-0.516865
H	1.819418	-0.034755	0.572541
H	4.320619	4.177270	-0.071991
H	4.259737	0.037852	1.007772
H	6.143500	2.962233	0.578600
O	-0.010824	1.834032	-1.759835
O	0.357770	3.051666	-2.385438
H	-0.501285	3.400740	-2.660302

ENERGIES
SCF ENERGY: -1715.78300166
SUM OF ELECTRONIC AND ZERO-POINT ENERGIES: -1715.365931
SUM OF ELECTRONIC AND THERMAL ENERGIES: -1715.335738
SUM OF ELECTRONIC AND THERMAL ENTHALPIES: -1715.334794
SUM OF ELECTRONIC AND THERMAL FREE ENERGIES: -1715.427242

==> VITEXIN/LIPID/RAF/C2' <==
54
XYZ-COORDINATES + ENERGIES

O	1.687049	-1.236511	-0.935687
O	2.473658	-0.438183	2.517229
O	1.466104	-3.063899	2.726809
O	1.750315	-4.682975	0.351082
O	-0.140992	0.739165	-0.089702
O	1.823079	-2.527410	-3.372300
O	4.524043	1.187490	0.012057
O	1.844208	5.065644	-0.366376
O	-0.706754	4.738183	-0.410350
O	-5.216283	-2.878581	-0.478028
C	1.797340	-1.094749	1.461360
C	2.042476	-2.589910	1.519283
C	2.347580	-0.560267	0.138393
C	1.455110	-3.290824	0.307026
C	1.974597	-2.632875	-0.967050
C	2.190209	0.925043	-0.023131
C	1.308904	-3.166899	-2.216095
C	0.949471	1.547222	-0.116354
C	3.305349	1.767862	-0.063849
C	0.791732	2.930345	-0.233689
C	3.199713	3.153900	-0.180225
C	1.950824	3.739460	-0.260181
C	-0.534922	3.510125	-0.312194
C	-1.398022	1.241218	-0.181030
C	-1.629679	2.575796	-0.275912
C	-2.405706	0.206578	-0.193619
C	-3.850219	0.606267	-0.146593
C	-2.045117	-1.126768	-0.238521
C	-4.806344	-0.526699	-0.313733
C	-2.996451	-2.135228	-0.302460
C	-4.386433	-1.808657	-0.357511
H	0.721959	-0.909298	1.517310
H	3.128539	-2.758023	1.530984
H	3.413720	-0.793729	0.102273
H	0.366692	-3.217478	0.338378
H	3.062118	-2.775686	-1.040296
H	0.227630	-3.013381	-2.135516
H	1.504830	-4.231619	-2.318252
H	2.219561	-0.878038	3.334849
H	1.538668	-4.023772	2.735515
H	2.701343	-4.797562	0.243371
H	4.084980	3.776002	-0.204832
H	1.753206	-1.580026	-3.218421
H	-2.635402	2.960722	-0.325404
H	5.211091	1.861945	-0.015209
H	0.875144	5.267641	-0.406855
H	-0.996057	-1.384206	-0.253724
H	-5.859034	-0.283622	-0.385172
H	-2.707165	-3.175378	-0.337532
H	-6.132104	-2.581735	-0.503358
H	-4.062041	1.382755	-0.886508
O	-4.008653	1.233369	1.158300
O	-5.269776	1.889392	1.163784
H	-5.788137	1.323371	1.750367

ENERGIES
SCF ENERGY: -1715.77946667
SUM OF ELECTRONIC AND ZERO-POINT ENERGIES: -1715.362538
SUM OF ELECTRONIC AND THERMAL ENERGIES: -1715.332251
SUM OF ELECTRONIC AND THERMAL ENTHALPIES: -1715.331307
SUM OF ELECTRONIC AND THERMAL FREE ENERGIES: -1715.423643

==> VITEXIN/LIPID/RAF/C3 <==
54
XYZ-COORDINATES + ENERGIES

O	1.728588	-1.073624	0.909341
O	1.036761	-2.348473	-2.419029
O	3.345253	-0.786090	-2.853618
O	5.041761	-0.415271	-0.548429
O	-0.656071	0.133272	-0.078133
O	3.097796	-0.683974	3.275715
O	0.046901	-4.457260	0.439852
O	-4.350131	-2.793478	0.754622
O	-4.676772	-0.245025	0.362234
O	1.636884	5.939014	0.363736
C	1.546385	-1.435858	-1.463635
C	3.054984	-1.329520	-1.575498
C	1.197998	-1.967860	-0.072851
C	3.622034	-0.470741	-0.458704
C	3.153982	-1.015041	0.886803
C	-0.276550	-2.150192	0.158934
C	3.555912	-0.135902	2.050668
C	-1.172740	-1.091837	0.149912
C	-0.814838	-3.422570	0.404307
C	-2.555556	-1.260317	0.356059
C	-2.175654	-3.642128	0.610787
C	-3.047762	-2.573231	0.575948
C	-3.444954	-0.144530	0.276088
C	-1.396572	1.273692	0.027528
C	-2.870481	1.224040	-0.013381
C	-0.641863	2.472916	0.101469
C	-1.266470	3.735402	0.035717
C	0.762968	2.429256	0.250343
C	-0.522726	4.895828	0.127428
C	1.502353	3.588832	0.335330
C	0.862838	4.828291	0.275324
H	1.111895	-0.443866	-1.603038
H	3.478321	-2.340443	-1.494008
H	1.680228	-2.941165	0.037659
H	3.275618	0.556777	-0.581540
H	3.557921	-2.026781	1.034772
H	3.148142	0.868464	1.898160
H	4.639591	-0.068187	2.109377
H	1.365819	-2.072249	-3.280415
H	4.289891	-0.604756	-2.893181
H	5.394874	-1.293630	-0.366788
H	-2.558627	-4.638361	0.786546
H	2.154276	-0.842125	3.172368
H	-0.428119	-5.279487	0.604498
H	-4.815689	-1.934715	0.657611
H	-2.335068	3.809579	-0.109301
H	1.250522	1.467285	0.316296
H	-1.013000	5.860216	0.072050
H	2.577237	3.558939	0.453693
H	1.087904	6.728129	0.309989
H	-3.304149	1.946599	0.680485
O	-3.327269	1.585326	-1.345544
O	-4.653523	2.087839	-1.242514
H	-5.158733	1.300533	-0.982558

ENERGIES
SCF ENERGY: -1715.79043586
SUM OF ELECTRONIC AND ZERO-POINT ENERGIES: -1715.372195
SUM OF ELECTRONIC AND THERMAL ENERGIES: -1715.342088
SUM OF ELECTRONIC AND THERMAL ENTHALPIES: -1715.341144
SUM OF ELECTRONIC AND THERMAL FREE ENERGIES: -1715.433397

==> VITEXIN/LIPID/RAF/C3/TS <==
54
XYZ-COORDINATES + ENERGIES

O	-1.599423	-1.318986	-0.906294
O	-0.776016	-2.237368	2.508688
O	-3.237111	-0.897340	2.834236
O	-4.961947	-0.888940	0.523393
O	0.622067	0.229241	-0.062513
O	-3.005420	-1.280072	-3.283580
O	0.463516	-4.447815	-0.157442
O	4.625918	-2.289972	-0.727637
O	4.628592	0.308014	-0.659329
O	-2.313911	5.713838	-0.280578
C	-1.379814	-1.466009	1.486852
C	-2.891218	-1.510356	1.602346
C	-0.974684	-2.068363	0.140630
C	-3.543916	-0.808584	0.424789
C	-3.022905	-1.412230	-0.875001
C	0.510420	-2.102110	-0.089404

C	-3.517856	-0.682783	-2.104246
C	1.280859	-0.952111	-0.183396
C	1.195329	-3.316461	-0.229156
C	2.664644	-0.967102	-0.396678
C	2.571478	-3.385592	-0.441514
C	3.308885	-2.220192	-0.524964
C	3.400985	0.268137	-0.472685
C	1.248888	1.417539	-0.199806
C	2.637158	1.498612	-0.258107
C	0.343944	2.542994	-0.208061
C	0.810361	3.844450	0.018977
C	-1.026373	2.341876	-0.441634
C	-0.062357	4.915878	-0.003464
C	-1.900223	3.409263	-0.466520
C	-1.418564	4.700246	-0.248157
H	-1.054958	-0.424850	1.542244
H	-3.206420	-2.563155	1.605780
H	-1.345448	-3.095042	0.111904
H	-3.301732	0.254982	0.459429
H	-3.314509	-2.470521	-0.933533
H	-3.219964	0.368964	-2.040008
H	-4.602636	-0.735446	-2.157401
H	-1.129188	-1.925045	3.347892
H	-4.195471	-0.812123	2.866367
H	-5.224950	-1.810208	0.417003
H	3.070214	-4.340912	-0.540313
H	-2.051125	-1.336931	-3.173543
H	1.034037	-5.217922	-0.256384
H	4.966240	-1.366889	-0.757086
H	1.853817	4.019283	0.241448
H	-1.389838	1.339267	-0.620464
H	0.301173	5.919073	0.181916
H	-2.955531	3.266412	-0.655228
H	-1.871924	6.553242	-0.113928
H	3.076635	2.411375	-0.630397
O	3.291809	1.750593	1.487136
O	4.518582	2.363084	1.335882
H	5.098083	1.649634	1.023805

ENERGIES
SCF ENERGY: -1715.77229163
SUM OF ELECTRONIC AND ZERO-POINT ENERGIES: -1715.356268
SUM OF ELECTRONIC AND THERMAL ENERGIES: -1715.326030
SUM OF ELECTRONIC AND THERMAL ENTHALPIES: -1715.325086
SUM OF ELECTRONIC AND THERMAL FREE ENERGIES: -1715.417925

==> VITEXIN/LIPID/RAF/C3' <==
54
XYZ-COORDINATES + ENERGIES

O	-1.289320	-1.614066	0.924390
O	-2.020830	-1.264509	-2.616246
O	-0.054130	-3.283532	-2.617771
O	0.159547	-4.811197	-0.180740
O	-0.310565	0.892898	0.065192
O	-1.099278	-2.798934	3.407062
O	-4.761665	-0.524203	-0.307462
O	-3.840138	4.095931	0.130409
O	-1.368744	4.788515	0.369929
O	5.709443	-0.529306	0.740112
C	-1.234301	-1.578155	-1.481407
C	-0.864178	-3.048338	-1.476205
C	-2.064597	-1.279370	-0.231841
C	-0.142773	-3.419727	-0.193086
C	-0.990487	-3.006507	1.005622
C	-2.518924	0.149212	-0.130564
C	-0.276884	-3.203600	2.325255
C	-1.631051	1.209166	0.016861
C	-3.874255	0.487555	-0.179026
C	-2.029764	2.543845	0.110011
C	-4.324200	1.806327	-0.097664
C	-3.412222	2.834207	0.046816
C	-1.042689	3.592158	0.286151
C	0.631265	1.841176	0.254340
C	0.326730	3.149421	0.377848
C	1.978409	1.251096	0.327815
C	3.074433	1.993130	0.000329
C	2.102368	-0.096425	0.760079
C	4.450056	1.436265	0.076094
C	3.368630	-0.679955	0.888234
C	4.494908	0.029020	0.581389
H	-0.316836	-0.985420	-1.468184
H	-1.789303	-3.637645	-1.546193
H	-2.951505	-1.915830	-0.260982
H	0.818542	-2.904108	-0.159528
H	-1.930592	-3.576339	1.008142
H	0.658821	-2.634787	2.313760
H	-0.043082	-4.255628	2.469541
H	-1.545327	-1.586611	-3.388702
H	0.279979	-4.184828	-2.564781
H	-0.667313	-5.303574	-0.125724
H	-5.381356	2.034023	-0.142067
H	-1.402701	-1.908028	3.206093
H	1.101779	3.876281	0.565056
H	-5.658216	-0.173474	-0.338126
H	-3.033914	4.660751	0.235107
H	2.985547	3.008973	-0.356603
H	1.212465	-0.656780	1.003101
H	3.474287	-1.698416	1.237703
H	6.371073	0.032507	0.313860
H	5.102860	2.079556	0.679518
O	4.973594	1.514774	-1.281682
O	6.400317	1.373674	-1.167378
H	6.606106	0.946020	-2.009807

ENERGIES
SCF ENERGY: -1715.77849806
SUM OF ELECTRONIC AND ZERO-POINT ENERGIES: -1715.361111
SUM OF ELECTRONIC AND THERMAL ENERGIES: -1715.330950
SUM OF ELECTRONIC AND THERMAL ENTHALPIES: -1715.330005
SUM OF ELECTRONIC AND THERMAL FREE ENERGIES: -1715.422468

==> VITEXIN/LIPID/RAF/C4 <==
54
XYZ-COORDINATES + ENERGIES

O	1.291436	-1.397870	0.989554
O	0.627382	-2.499735	-2.407251
O	3.322932	-1.692523	-2.566450
O	4.790312	-1.775530	-0.083903
O	-0.518313	0.414290	-0.261528
O	2.438164	-1.338660	3.492814
O	-1.226063	-4.181735	0.300638
O	-4.975051	-1.334145	0.245743
O	-3.911424	0.965644	-1.410304
O	3.488365	5.167663	0.023499
C	1.271353	-1.752423	-1.391094
C	2.747300	-2.093838	-1.332528
C	0.634046	-2.126950	-0.052288
C	3.415843	-1.409137	-0.154028
C	2.665251	-1.755621	1.127505
C	-0.847420	-1.877440	0.015666
C	3.176907	-0.996304	2.332070
C	-1.399547	-0.605452	-0.086349
C	-1.744140	-2.937207	0.192875
C	-2.773154	-0.366252	-0.007091
C	-3.118130	-2.740078	0.272797
C	-3.638823	-1.462843	0.168506
C	-3.298365	1.031657	-0.197649
C	-0.934021	1.716121	-0.184460
C	-2.219739	2.059619	-0.121428
C	0.212284	2.637175	-0.161547
C	0.094862	3.952217	-0.610175
C	1.444887	2.194485	0.326933
C	1.177280	4.815899	-0.553611
C	2.530447	3.050892	0.387296
C	2.395947	4.365033	-0.051796
H	1.162995	-0.680639	-1.567280
H	2.844947	-3.181695	-1.211393
H	0.801529	-3.194396	0.105896
H	3.400697	-0.328897	-0.308765
H	2.737535	-2.835793	1.317406
H	3.110930	0.077930	2.131883
H	4.216910	-1.252295	2.519839
H	1.122021	-2.357677	-3.220574
H	4.277553	-1.795527	-2.497009
H	4.845938	-2.714283	0.127646
H	-3.793451	-3.572707	0.418375

H 1.508008 -1.231576 3.270059
H -2.506042 3.096091 -0.040398
H -1.935088 -4.824637 0.408467
H -5.205420 -0.397706 0.330228
H -0.839219 4.303447 -1.027062
H 1.539474 1.173231 0.670227
H 1.082575 5.833377 -0.913421
H 3.486008 2.718475 0.770027
H 3.275133 6.045210 -0.309861
O -4.273453 1.222739 0.854209
O -4.956736 2.442149 0.593750
H -4.671850 2.984445 1.341908

ENERGIES
SCF ENERGY: -1715.74368543
SUM OF ELECTRONIC AND ZERO-POINT ENERGIES: -1715.326935
SUM OF ELECTRONIC AND THERMAL ENERGIES: -1715.296446
SUM OF ELECTRONIC AND THERMAL ENTHALPIES: -1715.295502
SUM OF ELECTRONIC AND THERMAL FREE ENERGIES: -1715.388622

==> VITEXIN/LIPID/RAF/C4a <==
54
XYZ-COORDINATES + ENERGIES
O 0.854589 -1.575671 1.005727
O -0.274595 -2.537732 -2.304320
O 2.534317 -2.570296 -2.599363
O 4.013390 -3.057944 -0.162395
O -0.375673 0.690174 -0.154210
O 2.087025 -1.796483 3.469437
O -2.421196 -3.515753 0.415766
O -5.120966 0.382807 0.583259
O -3.739633 2.480904 1.196071
O 4.777323 4.162752 -0.347629
C 0.613461 -1.987511 -1.349769
C 1.922168 -2.753711 -1.332570
C -0.036601 -2.112619 0.027661
C 2.821585 -2.280072 -0.203764
C 2.060086 -2.331516 1.117198
C -1.371931 -1.421115 0.121246
C 2.832680 -1.720913 2.265786
C -1.503736 -0.058918 0.031196
C -2.543929 -2.173622 0.323251
C -2.797246 0.649125 -0.042801
C -3.803513 -1.562497 0.491514
C -3.934820 -0.213430 0.393372
C -2.732458 1.999813 0.674470
C -0.357861 1.993854 0.175362
C -1.457555 2.639162 0.645516
C 0.970712 2.583955 0.021133
C 1.134353 3.961957 -0.133903
C 2.102835 1.761492 0.038174
C 2.399297 4.508670 -0.259423
C 3.369489 2.300274 -0.081800
C 3.518233 3.677884 -0.230515
H 0.816884 -0.936251 -1.567312
H 1.691946 -3.817279 -1.178248
H -0.189455 -3.174416 0.230897
H 3.142123 -1.256727 -0.405333
H 1.808662 -3.374011 1.357720
H 3.073604 -0.681079 2.022558
H 3.759870 -2.267066 2.422295
H 0.184660 -2.546478 -3.150119
H 3.406832 -2.975973 -2.570869
H 3.781585 -3.959711 0.087131
H -4.671050 -2.161056 0.740955
H 1.228478 -1.400992 3.289543
H -1.381157 3.652096 1.008421
H -3.285556 -3.916523 0.558477
H -4.925893 1.323494 0.808462
H 0.271682 4.611798 -0.179838
H 1.975172 0.696309 0.167650
H 2.522349 5.576468 -0.391676
H 4.250843 1.673820 -0.060755
H 4.755462 5.120525 -0.445301
O -2.932597 0.941639 -1.506479
O -3.989915 1.866268 -1.721154
H -4.745088 1.286163 -1.890349

ENERGIES
SCF ENERGY: -1715.75924624
SUM OF ELECTRONIC AND ZERO-POINT ENERGIES: -1715.342810
SUM OF ELECTRONIC AND THERMAL ENERGIES: -1715.312366
SUM OF ELECTRONIC AND THERMAL ENTHALPIES: -1715.311422
SUM OF ELECTRONIC AND THERMAL FREE ENERGIES: -1715.403951

==> VITEXIN/LIPID/RAF/C4' <==
54
XYZ-COORDINATES + ENERGIES
O 0.983847 1.616889 1.010864
O 2.268536 1.551634 -2.384593
O 0.142105 3.387554 -2.593705
O -0.567083 4.747111 -0.145348
O 0.417950 -0.925625 -0.081897
O 0.305401 2.626885 3.490883
O 4.697837 0.952510 0.257862
O 4.244153 -3.754116 0.240883
O 1.856762 -4.709768 0.059657
O -5.310747 -0.144124 -1.313391
C 1.297898 1.727232 -1.369268
C 0.792041 3.156396 -1.353061
C 1.956783 1.429352 -0.021272
C -0.143468 3.388321 -0.179736
C 0.545292 2.970239 1.115368
C 2.538487 0.047199 0.073881
C -0.378436 3.018956 2.312611
C 1.759546 -1.103665 0.026458
C 3.918081 -0.149402 0.193748
C 2.291160 -2.394749 0.079613
C 4.497086 -1.417409 0.249168
C 3.693268 -2.539410 0.190140
C 1.415220 -3.547870 0.020337
C -0.438678 -1.976815 -0.119797
C 0.006922 -3.260760 -0.074800
C -1.817076 -1.556025 -0.183147
C -2.863533 -2.483421 -0.478715
C -2.147580 -0.186172 0.052414
C -4.151611 -2.090680 -0.520235
C -3.425650 0.242181 0.028291
C -4.568480 -0.678282 -0.250219
H 0.450343 1.055182 -1.523793
H 1.657759 3.826169 -1.253981
H 2.767077 2.147627 0.120024
H -1.049990 2.797382 -0.321116
H 1.415451 3.616929 1.297009
H -1.238661 2.367250 2.127262
H -0.735975 4.034377 2.466000
H 1.889631 1.892086 -3.201441
H -0.275324 4.254187 -2.554994
H 0.196035 5.300616 0.055797
H 5.569526 -1.532528 0.336561
H 0.712449 1.774998 3.304837
H -0.681706 -4.090261 -0.080646
H 5.624022 0.696801 0.325487
H 3.500597 -4.404719 0.185432
H -2.622678 -3.514333 -0.693597
H -1.347793 0.507904 0.266347
H -4.941085 -2.794850 -0.747724
H -3.684325 1.276946 0.207511
H -5.884304 -0.832821 -1.667966
O -5.352596 -0.622259 0.959739
O -6.535334 -1.384383 0.748330
H -7.191313 -0.688724 0.608038

ENERGIES
SCF ENERGY: -1715.78449955
SUM OF ELECTRONIC AND ZERO-POINT ENERGIES: -1715.367868
SUM OF ELECTRONIC AND THERMAL ENERGIES: -1715.337663
SUM OF ELECTRONIC AND THERMAL ENTHALPIES: -1715.336719
SUM OF ELECTRONIC AND THERMAL FREE ENERGIES: -1715.429118

==> VITEXIN/LIPID/RAF/C5 <==
54
XYZ-COORDINATES + ENERGIES
O 0.734300 -1.663690 1.004909
O -0.522204 -2.103392 -2.370134
O 2.250801 -2.532715 -2.708172

O	3.645861	-3.458509	-0.352827
O	-0.147603	0.828399	0.061503
O	1.930302	-2.295020	3.414974
O	-2.725404	-3.063830	0.521649
O	-4.725520	1.112534	1.140680
O	-3.305175	3.301499	0.531089
O	5.505866	3.398954	-0.255107
C	0.441185	-1.797333	-1.380885
C	1.619169	-2.748520	-1.456748
C	-0.222010	-1.968959	-0.012308
C	2.576993	-2.520277	-0.299664
C	1.819022	-2.593239	1.022677
C	-1.445524	-1.120547	0.167898
C	2.674507	-2.214030	2.211415
C	-1.390746	0.308610	0.151932
C	-2.709050	-1.710255	0.389484
C	-2.485656	1.111284	0.250649
C	-3.851984	-0.968997	0.485703
C	-3.856447	0.500845	0.243965
C	-2.337312	2.550852	0.341726
C	0.050142	2.168466	0.089676
C	-0.979743	3.029989	0.236551
C	1.470222	2.510641	-0.020474
C	1.874019	3.772797	-0.457612
C	2.442868	1.564185	0.319453
C	3.218752	4.090322	-0.542486
C	3.787646	1.875339	0.240189
C	4.177149	3.141409	-0.190882
H	0.806326	-0.774079	-1.493131
H	1.234871	-3.776587	-1.402500
H	-0.523250	-3.013396	0.083189
H	3.035813	-1.535928	-0.405979
H	1.419267	-3.606538	1.165767
H	3.065421	-1.202146	2.062338
H	3.512254	-2.901626	2.299838
H	-0.068366	-2.120720	-3.219023
H	3.057262	-3.058027	-2.730857
H	3.289713	-4.339109	-0.188720
H	-4.809769	-1.437410	0.665000
H	1.135103	-1.768307	3.288537
H	-0.799345	4.091305	0.312511
H	-3.635170	-3.373506	0.583028
H	-4.526696	2.068608	1.081219
H	1.139919	4.508175	-0.756301
H	2.134542	0.585330	0.660747
H	3.525801	5.068445	-0.891910
H	4.547015	1.153878	0.509420
H	5.654102	4.299500	-0.562189
O	-4.306780	0.767954	-1.111898
O	-5.619913	0.250473	-1.256131
H	-6.150299	0.895387	-0.766820

ENERGIES
SCF ENERGY: -1715.77803085
SUM OF ELECTRONIC AND ZERO-POINT ENERGIES: -1715.361304
SUM OF ELECTRONIC AND THERMAL ENERGIES: -1715.331046
SUM OF ELECTRONIC AND THERMAL ENTHALPIES: -1715.330102
SUM OF ELECTRONIC AND THERMAL FREE ENERGIES: -1715.422964

==> VITEXIN/LIPID/RAF/C5' <==
54
XYZ-COORDINATES + ENERGIES

O	-0.275663	-1.663645	1.000581
O	-1.380433	-2.019326	-2.441174
O	1.302258	-2.856384	-2.602617
O	2.443271	-3.862352	-0.153052
O	-0.729822	0.910094	-0.054579
O	0.698102	-2.363801	3.481860
O	-3.931324	-2.502896	0.132701
O	-5.361359	2.007011	0.189518
O	-3.529481	3.825845	0.099870
O	5.247739	2.667649	-0.014967
C	-0.448459	-1.822110	-1.393252
C	0.586267	-2.929366	-1.379391
C	-1.210600	-1.852149	-0.067011
C	1.507774	-2.788730	-0.181686
C	0.675633	-2.723682	1.095480
C	-2.298165	-0.819124	0.025467
C	1.510949	-2.425841	2.321124
C	-2.035719	0.545980	0.016484
C	-3.645602	-1.182123	0.105264
C	-3.028394	1.525834	0.070579
C	-4.676532	-0.242238	0.158503
C	-4.376932	1.106404	0.139031
C	-2.668555	2.930717	0.060940
C	-0.357095	2.206328	-0.029195
C	-1.253390	3.213281	0.023700
C	1.109068	2.342910	-0.032047
C	1.713095	3.555689	-0.451875
C	1.864892	1.285701	0.386917
C	3.112707	3.679967	-0.445141
C	3.348250	1.338188	0.437817
C	3.894282	2.647030	-0.022787
H	0.064906	-0.864255	-1.504630
H	0.060981	-3.892654	-1.311492
H	-1.669500	-2.838248	0.032002
H	2.100836	-1.878538	-0.285391
H	0.139755	-3.673025	1.237224
H	2.044039	-1.481687	2.168476
H	2.241800	-3.215870	2.475544
H	-0.874100	-2.150461	-3.249255
H	2.070519	-3.432523	-2.531918
H	1.962276	-4.681989	0.007931
H	-5.710353	-0.557653	0.214825
H	-0.023146	-1.758979	3.281725
H	-0.928060	4.240920	0.067969
H	-4.884717	-2.632785	0.176070
H	-4.931238	2.897430	0.166019
H	1.108846	4.371093	-0.817022
H	1.397073	0.364648	0.703471
H	3.574299	4.596617	-0.792074
H	3.720491	1.097160	1.439458
H	5.578273	3.499479	-0.375941
O	3.837073	0.274708	-0.427674
O	5.094291	-0.175796	0.056044
H	5.698962	0.506488	-0.268581

ENERGIES
SCF ENERGY: -1715.77898889
SUM OF ELECTRONIC AND ZERO-POINT ENERGIES: -1715.361490
SUM OF ELECTRONIC AND THERMAL ENERGIES: -1715.331440
SUM OF ELECTRONIC AND THERMAL ENTHALPIES: -1715.330496
SUM OF ELECTRONIC AND THERMAL FREE ENERGIES: -1715.422118

==> VITEXIN/LIPID/RAF/C6 <==
54
XYZ-COORDINATES + ENERGIES

O	0.451257	-1.678164	1.032523
O	-0.716556	-1.933542	-2.396255
O	1.954791	-2.802890	-2.619889
O	3.118224	-3.886687	-0.215490
O	0.065950	0.946796	0.022461
O	1.445081	-2.468402	3.484258
O	-3.248344	-2.412036	0.222638
O	-4.532128	2.169343	0.423235
O	-2.667234	3.924863	0.275557
O	6.061153	2.550619	-0.232699
C	0.236997	-1.774970	-1.363069
C	1.253591	-2.899404	-1.390005
C	-0.500419	-1.823326	-0.023885
C	2.193669	-2.804217	-0.201814
C	1.382380	-2.758346	1.089235
C	-1.561857	-0.766685	0.118475
C	2.239139	-2.506584	2.310398
C	-1.242602	0.617545	0.132289
C	-2.874687	-1.115098	0.216862
C	-2.217624	1.617868	0.223865
C	-3.999880	-0.144590	0.364925
C	-3.550293	1.264412	0.343253
C	-1.816357	3.019356	0.205108
C	0.472019	2.231948	0.025906
C	-0.406070	3.262562	0.120049
C	1.930008	2.339833	-0.061731
C	2.545392	3.525336	-0.467794
C	2.728889	1.238837	0.268649
C	3.925145	3.615174	-0.531182

C	4.107352	1.322583	0.211259
C	4.707768	2.514175	-0.188771
H	0.764884	-0.823849	-1.463312
H	0.714143	-3.855524	-1.340147
H	-0.981283	-2.800378	0.057959
H	2.794076	-1.897234	-0.290991
H	0.830600	-3.700693	1.213824
H	2.781518	-1.564730	2.176967
H	2.960837	-3.310766	2.431775
H	-0.228979	-2.052725	-3.217670
H	2.678523	-3.437439	-2.600836
H	2.633741	-4.705211	-0.058740
H	0.738083	-1.836505	3.320742
H	-0.053469	4.281380	0.152524
H	-4.183029	-2.471425	-0.011737
H	-4.084073	3.058228	0.375838
H	1.951506	4.381011	-0.757154
H	2.257809	0.318369	0.584648
H	4.397796	4.533647	-0.856438
H	4.731473	0.479146	0.473498
H	6.359931	3.420314	-0.518378
H	-4.582610	-0.366305	1.267790
O	-4.906896	-0.410721	-0.749261
O	-6.223557	-0.051706	-0.359522
H	-6.210749	0.912452	-0.452749

ENERGIES
SCF ENERGY: -1715.78043210
SUM OF ELECTRONIC AND ZERO-POINT ENERGIES: -1715.362537
SUM OF ELECTRONIC AND THERMAL ENERGIES: -1715.332455
SUM OF ELECTRONIC AND THERMAL ENTHALPIES: -1715.331511
SUM OF ELECTRONIC AND THERMAL FREE ENERGIES: -1715.423306

==> VITEXIN/LIPID/RAF/C6' <==
54
XYZ-COORDINATES + ENERGIES

O	-0.244168	-1.665915	1.116680
O	-1.611538	-2.098916	-2.220083
O	0.903836	-3.334359	-2.460664
O	2.051847	-4.317361	-0.002246
O	-0.414384	0.902347	0.064712
O	0.787656	-2.317999	3.587951
O	-4.031576	-2.071771	0.330903
O	-4.879251	2.576457	0.159865
O	-2.846713	4.144018	0.029665
O	5.736475	1.893799	0.480071
C	-0.595068	-1.964436	-1.242167
C	0.269428	-3.209169	-1.198284
C	-1.264204	-1.794240	0.122242
C	1.278727	-3.123369	-0.067904
C	0.553423	-2.841409	1.244528
C	-2.194752	-0.615509	0.178447
C	1.503513	-2.574452	2.391068
C	-1.757732	0.703733	0.116846
C	-3.579131	-0.800788	0.249120
C	-2.624645	1.800299	0.113965
C	-4.484220	0.260411	0.241217
C	-4.016344	1.557970	0.169682
C	-2.097913	3.149801	0.052551
C	0.115726	2.148927	0.059616
C	-0.664828	3.259504	0.025477
C	1.564887	2.120910	0.108412
C	2.302625	3.229506	0.454894
C	2.246102	0.832064	-0.248163
C	3.691962	3.172054	0.551562
C	3.715188	0.825968	-0.021032
C	4.383998	1.946972	0.330142
H	0.035597	-1.097602	-1.454009
H	-0.384393	-4.075309	-1.021113
H	-1.847469	-2.694848	0.326364
H	1.988192	-2.321645	-0.279398
H	-0.096922	-3.691380	1.495371
H	2.144393	-1.725013	2.131535
H	2.131736	-3.444417	2.566372
H	-1.178693	-2.344609	-3.044019
H	1.556674	-4.039020	-2.397841
H	1.471336	-5.043352	0.252995
H	-5.549583	0.077072	0.288346
H	0.144384	-1.632190	3.383050
H	-0.224517	4.243095	-0.018137
H	-4.994329	-2.077034	0.355718
H	-4.336593	3.403829	0.105316
H	1.808468	4.160268	0.696023
H	1.769661	0.002918	0.282977
H	4.248424	4.058332	0.827150
H	4.249741	-0.097682	-0.196229
H	6.071216	2.747123	0.771534
O	1.940225	0.472443	-1.624238
O	2.504578	1.452974	-2.483068
H	1.736324	1.997257	-2.701718

ENERGIES
SCF ENERGY: -1715.77746204
SUM OF ELECTRONIC AND ZERO-POINT ENERGIES: -1715.361554
SUM OF ELECTRONIC AND THERMAL ENERGIES: -1715.330935
SUM OF ELECTRONIC AND THERMAL ENTHALPIES: -1715.329991
SUM OF ELECTRONIC AND THERMAL FREE ENERGIES: -1715.423102

==> VITEXIN/LIPID/RAF/C7 <==
54
XYZ-COORDINATES + ENERGIES

O	-0.135038	-1.600227	0.831169
O	-0.817050	-1.441511	-2.731541
O	1.509874	-3.006532	-2.641287
O	1.958552	-4.451111	-0.178039
O	0.194301	1.077668	-0.134478
O	0.229036	-2.692470	3.347304
O	-3.691239	-1.202202	-1.121772
O	-4.059205	3.305023	-0.096389
O	-1.825402	4.578411	0.082542
O	6.383455	1.320577	0.297707
C	-0.037936	-1.586203	-1.562431
C	0.634240	-2.946072	-1.525948
C	-0.950905	-1.479676	-0.337225
C	1.383145	-3.149896	-0.219888
C	0.444350	-2.899569	0.955267
C	-1.739478	-0.205814	-0.250951
C	1.150053	-2.913189	2.292595
C	-1.168273	1.040015	-0.142328
C	-3.232863	-0.324661	-0.140502
C	-1.908343	2.226865	-0.063172
C	-3.979366	0.967251	-0.157881
C	-3.355642	2.158167	-0.102427
C	-1.207530	3.501694	0.034288
C	0.866517	2.237646	0.002889
C	0.225315	3.426857	0.092965
C	2.312863	2.023773	0.061051
C	3.209242	3.063650	-0.193855
C	2.814033	0.756843	0.383580
C	4.573899	2.848786	-0.120138
C	4.175833	0.536422	0.463874
C	5.058603	1.584297	0.211051
H	0.732657	-0.814379	-1.510331
H	-0.141894	-3.718264	-1.616793
H	-1.664415	-2.305943	-0.368689
H	2.220498	-2.451470	-0.176462
H	-0.358217	-3.649912	0.956821
H	1.932239	-2.146806	2.291788
H	1.611816	-3.883435	2.459993
H	-0.247650	-1.654928	-3.477947
H	2.015083	-3.823724	-2.580616
H	1.246611	-5.099579	-0.134868
H	-5.058874	0.927326	-0.213829
H	-0.276365	-1.906523	3.116331
H	0.778343	4.342271	0.231844
H	-4.641660	-1.309352	-0.996175
H	-3.410567	4.044123	-0.034145
H	2.848015	4.043980	-0.471164
H	2.124230	-0.050767	0.586647
H	5.264535	3.656300	-0.328985
H	4.572221	-0.436125	0.721476
H	6.893471	2.114357	0.104546
O	-3.372263	-0.963730	1.162932
O	-4.752779	-1.243305	1.358071
H	-5.024579	-0.524979	1.945936

ENERGIES
SCF ENERGY: -1715.77140438
SUM OF ELECTRONIC AND ZERO-POINT ENERGIES: -1715.355264
SUM OF ELECTRONIC AND THERMAL ENERGIES: -1715.325000
SUM OF ELECTRONIC AND THERMAL ENTHALPIES: -1715.324055
SUM OF ELECTRONIC AND THERMAL FREE ENERGIES: -1715.416726

==> VITEXIN/LIPID/RAF/C8 <==
54
XYZ-COORDINATES + ENERGIES

O	0.038630	-1.683527	0.605300
O	-2.635812	-1.060797	-1.751506
O	-0.745748	-2.429315	-3.314929
O	1.005542	-4.215626	-1.868210
O	0.212978	1.056030	0.353792
O	1.644054	-3.120742	2.278739
O	-3.887800	-1.066986	1.380044
O	-3.916439	3.331713	-0.434479
O	-1.617352	4.400185	-0.969509
O	6.424745	0.904646	0.285614
C	-1.309372	-1.300066	-1.321109
C	-0.789056	-2.601254	-1.907097
C	-1.288620	-1.409273	0.201376
C	0.573541	-2.965940	-1.341722
C	0.488966	-2.966511	0.178707
C	-1.769222	-0.118731	0.910363
C	1.815854	-3.177375	0.871507
C	-1.135688	1.121247	0.375605
C	-3.261438	0.029049	0.875194
C	-1.813631	2.233240	-0.062530
C	-3.926721	1.142932	0.475933
C	-3.229988	2.258266	-0.021027
C	-1.047423	3.373652	-0.564610
C	0.956267	2.082098	-0.096406
C	0.379121	3.225062	-0.546663
C	2.391173	1.793111	-0.018629
C	3.332353	2.628857	-0.623733
C	2.837020	0.660192	0.673067
C	4.684675	2.349518	-0.534312
C	4.186687	0.377034	0.769296
C	5.113571	1.222942	0.165524
H	-0.646463	-0.488467	-1.638590
H	-1.499825	-3.398431	-1.649281
H	-1.946015	-2.225062	0.509794
H	1.311867	-2.229815	-1.663254
H	-0.226785	-3.729582	0.516595
H	2.518985	-2.411098	0.530591
H	2.219885	-4.157118	0.626332
H	-2.635820	-1.103785	-2.713537
H	-0.327547	-3.207765	-3.697000
H	0.431964	-4.908755	-1.522443
H	-5.008289	1.180663	0.520082
H	1.052991	-2.379994	2.456915
H	0.981174	4.052980	-0.885917
H	-4.844273	-0.948811	1.340186
H	-3.245434	3.991582	-0.734319
H	3.018063	3.500709	-1.179310
H	2.116744	0.003435	1.139671
H	5.408172	2.999993	-1.010407
H	4.537319	-0.491051	1.310625
H	6.967179	1.560499	-0.164711
O	-1.344638	-0.155828	2.317228
O	-1.533136	-1.449079	2.874215
H	-2.472490	-1.443823	3.105172

ENERGIES
SCF ENERGY: -1715.77740835
SUM OF ELECTRONIC AND ZERO-POINT ENERGIES: -1715.360051
SUM OF ELECTRONIC AND THERMAL ENERGIES: -1715.330085
SUM OF ELECTRONIC AND THERMAL ENTHALPIES: -1715.329141
SUM OF ELECTRONIC AND THERMAL FREE ENERGIES: -1715.420573

==> VITEXIN/LIPID/RAF/C8a <==
54
XYZ-COORDINATES + ENERGIES

O	0.429344	-1.619036	1.047075
O	-1.102973	-2.355205	-2.168968
O	1.525595	-3.219542	-2.565837
O	2.984873	-3.962436	-0.185252
O	-0.265223	0.950762	0.135500
O	1.703763	-2.049085	3.453841
O	-3.156865	-2.569329	1.136717
O	-4.833870	1.852008	0.864579
O	-3.217356	3.722949	0.152605
O	5.588516	3.011713	-0.087497
C	-0.048500	-2.041885	-1.261463
C	0.979377	-3.154418	-1.258308
C	-0.638062	-1.926383	0.144464
C	2.047381	-2.892235	-0.210782
C	1.385026	-2.673990	1.145901
C	-1.732332	-0.908057	0.284903
C	2.365751	-2.247675	2.216342
C	-1.551042	0.493267	-0.218936
C	-2.960713	-1.274523	0.771436
C	-2.607635	1.457069	0.177478
C	-4.023345	-0.357796	0.908029
C	-3.822846	1.020280	0.627478
C	-2.320549	2.865620	0.027311
C	0.026596	2.263331	-0.062893
C	-0.934517	3.208539	-0.181260
C	1.475318	2.492710	-0.073054
C	2.015184	3.775334	-0.206330
C	2.349977	1.406873	0.058173
C	3.384400	3.969200	-0.216705
C	3.720358	1.592926	0.053554
C	4.240504	2.876248	-0.086320
H	0.431320	-1.104413	-1.545778
H	0.470752	-4.097743	-1.017598
H	-1.054525	-2.899680	0.411768
H	2.614473	-2.002522	-0.490505
H	0.873274	-3.594399	1.460505
H	2.869662	-1.331173	1.893286
H	3.112871	-3.023346	2.366972
H	-0.693591	-2.621783	-3.000844
H	2.254633	-3.848406	-2.555409
H	2.542595	-4.750362	0.150537
H	-4.988740	-0.679455	1.275624
H	0.981929	-1.434987	3.286484
H	-0.683538	4.246927	-0.322834
H	-4.049365	-2.687076	1.477633
H	-4.499290	2.756413	0.622771
H	1.372819	4.638566	-0.302946
H	1.942930	0.412487	0.173110
H	3.791476	4.967313	-0.323139
H	4.399506	0.757471	0.156277
H	5.827471	3.938923	-0.189648
O	-1.434005	0.479444	-1.680007
O	-2.628303	-0.035017	-2.229348
H	-2.366619	-0.967167	-2.347641

ENERGIES
SCF ENERGY: -1715.77877340
SUM OF ELECTRONIC AND ZERO-POINT ENERGIES: -1715.361068
SUM OF ELECTRONIC AND THERMAL ENERGIES: -1715.331534
SUM OF ELECTRONIC AND THERMAL ENTHALPIES: -1715.330590
SUM OF ELECTRONIC AND THERMAL FREE ENERGIES: -1715.420659

==> VITEXIN/WATER <==
51
XYZ-COORDINATES + ENERGIES

O	0.104276	-1.642218	1.006959
O	-1.057277	-2.098794	-2.405143
O	1.697535	-2.765758	-2.619502
O	2.895685	-3.732336	-0.164979
O	-0.410233	0.905029	-0.038559
O	1.182990	-2.370057	3.495910
O	-3.544647	-2.583344	0.110666
O	-5.076631	1.899475	0.212359
O	-3.271826	3.764159	0.123995
O	5.519570	2.790092	-0.096339
C	-0.097643	-1.828685	-1.390405
C	0.992425	-2.880802	-1.387771
C	-0.832557	-1.868177	-0.049467
C	1.918281	-2.696908	-0.197172
C	1.098033	-2.667232	1.090588
C	-1.943733	-0.858937	0.040461

C 1.950142 -2.338870 2.294588
C -1.708252 0.513331 0.033757
C -3.282914 -1.252530 0.108459
C -2.722675 1.471454 0.091083
C -4.333321 -0.333900 0.166741
C -4.058533 1.016744 0.157743
C -2.384649 2.879197 0.080161
C -0.060481 2.209220 -0.030429
C -0.987844 3.194377 0.031615
C 1.392867 2.382544 -0.072628
C 1.964764 3.612071 -0.411035
C 2.230921 1.305183 0.238278
C 3.339692 3.767553 -0.426959
C 3.605506 1.454058 0.228292
C 4.159289 2.687921 -0.102032
H 0.353594 -0.846258 -1.543628
H 0.524701 -3.870061 -1.318689
H -1.257746 -2.866682 0.067202
H 2.467543 -1.760094 -0.303708
H 0.603655 -3.636549 1.231329
H 2.403023 -1.353467 2.155567
H 2.736827 -3.080683 2.400856
H -0.585303 -2.175061 -3.241714
H 2.334315 -3.486804 -2.668939
H 2.439794 -4.578970 -0.084497
H -5.357812 -0.677445 0.216614
H 0.469808 -1.729962 3.402805
H -0.689287 4.230539 0.063395
H -4.494342 -2.739586 0.182342
H -4.673987 2.800878 0.192597
H 1.341228 4.453278 -0.678896
H 1.803429 0.349792 0.505266
H 3.785305 4.717175 -0.693686
H 4.256495 0.626994 0.477037
H 5.787312 3.687334 -0.327409

ENERGIES
SCF ENERGY: -1564.87840113
SUM OF ELECTRONIC AND ZERO-POINT ENERGIES: -1564.480453
SUM OF ELECTRONIC AND THERMAL ENERGIES: -1564.452809
SUM OF ELECTRONIC AND THERMAL ENTHALPIES: -1564.451865
SUM OF ELECTRONIC AND THERMAL FREE ENERGIES: -1564.538056

==> VITEXIN/WATER/AIP <==
51
XYZ-COORDINATES + ENERGIES
O 0.158719 -1.586898 1.008224
O -1.124625 -2.212460 -2.330246
O 1.617313 -2.911839 -2.604767
O 2.892321 -3.763910 -0.142200
O -0.399894 0.891097 -0.064973
O 1.327297 -2.174394 3.492782
O -3.522486 -2.584702 0.212366
O -5.075225 1.881537 0.232955
O -3.253105 3.764872 0.063273
O 5.422140 2.822061 -0.118561
C -0.130118 -1.900830 -1.363437
C 0.954820 -2.958623 -1.345482
C -0.816004 -1.872627 0.003114
C 1.924242 -2.720977 -0.199131
C 1.153116 -2.609488 1.114419
C -1.929237 -0.863056 0.078103
C 2.049864 -2.214560 2.264654
C -1.698870 0.503865 0.033103
C -3.269732 -1.258875 0.174017
C -2.717347 1.465756 0.083669
C -4.322098 -0.342311 0.226694
C -4.053770 1.007764 0.182338
C -2.391813 2.867811 0.029936
C -0.052673 2.191381 -0.098502
C -0.979425 3.187623 -0.061308
C 1.374483 2.387601 -0.127669
C 1.934113 3.672567 -0.387582
C 2.227258 1.272945 0.113482
C 3.281407 3.840636 -0.399989
C 3.575736 1.431289 0.115914
C 4.121806 2.722049 -0.137847
H 0.319071 -0.928041 -1.576374
H 0.484322 -3.939511 -1.209193
H -1.232924 -2.864015 0.189113
H 2.479766 -1.799145 -0.379361
H 0.662027 -3.566368 1.331091
H 2.498326 -1.240168 2.052597
H 2.839211 -2.951572 2.384012
H -0.683973 -2.323083 -3.180095
H 2.236054 -3.649015 -2.645424
H 2.435173 -4.598435 0.019290
H -5.344223 -0.688214 0.300504
H 0.609463 -1.541431 3.387419
H -0.691669 4.226602 -0.077098
H -4.470106 -2.748439 0.300176
H -4.698964 2.787653 0.188693
H 1.293074 4.515074 -0.596693
H 1.790193 0.306983 0.316910
H 3.736181 4.800519 -0.603921
H 4.253110 0.612226 0.310603
H 5.731701 3.725829 -0.294045

ENERGIES
SCF ENERGY: -1564.64846812
SUM OF ELECTRONIC AND ZERO-POINT ENERGIES: -1564.250039
SUM OF ELECTRONIC AND THERMAL ENERGIES: -1564.222422
SUM OF ELECTRONIC AND THERMAL ENTHALPIES: -1564.221478
SUM OF ELECTRONIC AND THERMAL FREE ENERGIES: -1564.307963

==> VITEXIN/WATER/BDE/C4' <==
50
XYZ-COORDINATES + ENERGIES
O 0.263881 -1.576684 1.012984
O -0.938788 -2.249175 -2.347457
O 1.849228 -2.750195 -2.599769
O 3.160279 -3.531656 -0.136625
O -0.435472 0.886840 -0.048700
O 1.435130 -2.116741 3.502350
O -3.336002 -2.789297 0.174174
O -5.160608 1.578809 0.207783
O -3.470996 3.563260 0.086737
O 5.258673 3.214421 -0.173898
C 0.022699 -1.877006 -1.368025
C 1.178444 -2.857030 -1.348486
C -0.678435 -1.907542 -0.009248
C 2.116224 -2.564428 -0.188695
C 1.324096 -2.531033 1.116329
C -1.851330 -0.968338 0.064716
C 2.176882 -2.094670 2.285023
C -1.705441 0.414602 0.033154
C -3.162700 -1.446638 0.144822
C -2.782296 1.306703 0.080894
C -4.271479 -0.598348 0.190500
C -4.086892 0.766375 0.160193
C -2.540686 2.730473 0.049496
C -0.173408 2.211718 -0.064783
C -1.159764 3.138100 -0.019637
C 1.255713 2.488866 -0.104717
C 1.732994 3.798675 -0.368083
C 2.170819 1.431391 0.126842
C 3.069864 4.049353 -0.394651
C 3.512996 1.662790 0.109085
C 4.031160 2.990387 -0.154075
H 0.405062 -0.873969 -1.569869
H 0.776918 -3.870044 -1.227460
H -1.036248 -2.924995 0.158377
H 2.602748 -1.601170 -0.348763
H 0.897081 -3.522951 1.310016
H 2.563899 -1.090578 2.091483
H 3.010324 -2.780716 2.408081
H -0.484077 -2.318880 -3.194128
H 2.531655 -3.429285 -2.632772
H 2.766586 -4.399995 0.011498
H -5.270473 -1.008970 0.250227
H 0.683948 -1.523899 3.395668
H -0.936986 4.193145 -0.017996
H -4.272625 -3.005924 0.261480
H -4.826548 2.504697 0.177574
H 1.035964 4.599520 -0.564725
H 1.791866 0.442246 0.334803

H 3.455467 5.037971 -0.600852
H 4.226072 0.870743 0.292789

ENERGIES
SCF ENERGY: -1564.22544577
SUM OF ELECTRONIC AND ZERO-POINT ENERGIES: -1563.841190
SUM OF ELECTRONIC AND THERMAL ENERGIES: -1563.813483
SUM OF ELECTRONIC AND THERMAL ENTHALPIES: -1563.812538
SUM OF ELECTRONIC AND THERMAL FREE ENERGIES: -1563.900069

==> VITEXIN/WATER/BDE/C4'/TS <==
54
XYZ-COORDINATES + ENERGIES
O 0.823771 1.707683 1.027353
O 1.604956 1.788236 -2.521168
O -0.929166 3.094228 -2.431451
O -1.526756 4.383697 0.093713
O 0.590867 -0.916667 0.100362
O 0.285762 2.763113 3.597866
O 4.439579 1.730413 -0.364598
O 4.907153 -2.985571 -0.132797
O 2.706496 -4.380769 0.152811
O -5.564912 -1.427565 0.818186
C 0.747493 1.782760 -1.385189
C -0.055446 3.068118 -1.306621
C 1.641436 1.676834 -0.146344
C -0.831338 3.143266 0.000552
C 0.120476 2.944534 1.180447
C 2.488676 0.432367 -0.130280
C -0.618898 2.855071 2.498094
C 1.944554 -0.845617 -0.010256
C 3.882850 0.497531 -0.248994
C 2.710538 -2.019326 -0.001523
C 4.694155 -0.642937 -0.255543
C 4.115345 -1.890506 -0.130247
C 2.058493 -3.304267 0.137556
C -0.046012 -2.100085 0.237814
C 0.623986 -3.277763 0.266152
C -1.497588 -1.929486 0.370918
C -2.367830 -3.014561 0.157383
C -2.017296 -0.666068 0.710070
C -3.730496 -2.845531 0.290655
C -3.378634 -0.488324 0.847155
C -4.256613 -1.580010 0.646371
H 0.062333 0.931889 -1.430513
H 0.637665 3.917897 -1.355322
H 2.302828 2.546609 -0.135128
H -1.598116 2.365336 0.013492
H 0.845724 3.768871 1.212026
H -1.285773 1.987610 2.480262
H -1.208968 3.755471 2.652776
H 1.049950 1.855482 -3.312932
H -1.407050 3.937476 -2.421015
H -0.875063 5.103442 0.089810
H 5.768677 -0.549523 -0.354739
H 0.790137 1.941974 3.500426
H 0.100397 -4.211907 0.406329
H 5.406697 1.664075 -0.409196
H 4.317970 -3.775059 -0.034094
H -1.978094 -3.980991 -0.132764
H -1.346413 0.165504 0.875034
H -4.417220 -3.665241 0.122873
H -3.795782 0.475357 1.111678
H -6.015105 -1.064326 -0.161843
O -6.178195 -0.747819 -1.368675
O -4.899509 -0.792011 -1.829626
H -4.802264 -1.681678 -2.221997

ENERGIES
SCF ENERGY: -1715.79153952
SUM OF ELECTRONIC AND ZERO-POINT ENERGIES: -1715.382646
SUM OF ELECTRONIC AND THERMAL ENERGIES: -1715.352838
SUM OF ELECTRONIC AND THERMAL ENTHALPIES: -1715.351894
SUM OF ELECTRONIC AND THERMAL FREE ENERGIES: -1715.443478

==> VITEXIN/WATER/BDE/C5 <==
50
XYZ-COORDINATES + ENERGIES
O 0.037236 -1.551428 1.008070
O -1.187735 -2.010489 -2.376543
O 1.538544 -2.824994 -2.604591
O 2.682660 -3.838137 -0.136580
O -0.424123 0.942588 -0.028554
O 1.108514 -2.275188 3.506833
O -3.606087 -2.485861 0.064056
O -5.047125 1.985021 0.269497
O -3.078557 3.986595 0.086791
O 5.613154 2.474906 -0.148156
C -0.199700 -1.780173 -1.382541
C 0.835338 -2.887645 -1.369413
C -0.912481 -1.778538 -0.027309
C 1.778089 -2.739601 -0.185215
C 0.977894 -2.627583 1.111424
C -2.008222 -0.752173 0.047826
C 1.860637 -2.313258 2.296786
C -1.734265 0.650723 0.047723
C -3.354046 -1.154801 0.089295
C -2.699588 1.628532 0.104172
C -4.362728 -0.225554 0.159061
C -4.095964 1.192734 0.182031
C -2.287784 3.034572 0.073324
C 0.012069 2.224725 -0.025568
C -0.862307 3.251271 0.034315
C 1.471703 2.309844 -0.076779
C 2.111259 3.504929 -0.417513
C 2.246126 1.184125 0.224857
C 3.492532 3.577508 -0.445947
C 3.627368 1.250147 0.200559
C 4.249410 2.449732 -0.134240
H 0.296845 -0.822113 -1.547046
H 0.315211 -3.849012 -1.284695
H -1.362559 -2.762289 0.114051
H 2.388724 -1.845187 -0.317625
H 0.431829 -3.562523 1.286497
H 2.365009 -1.358136 2.129013
H 2.607120 -3.094515 2.410740
H -0.741399 -2.079183 -3.227769
H 2.136541 -3.579079 -2.648011
H 2.170242 -4.651713 -0.052410
H -5.398098 -0.535271 0.208359
H 0.445375 -1.583160 3.416889
H -0.507794 4.270124 0.059291
H -4.555506 -2.648230 0.131469
H 1.536068 4.382362 -0.677483
H 1.762874 0.256654 0.494748
H 3.992095 4.499347 -0.714971
H 4.230233 0.384813 0.440595
H 5.923325 3.355901 -0.389315

ENERGIES
SCF ENERGY: -1564.21408848
SUM OF ELECTRONIC AND ZERO-POINT ENERGIES: -1563.829024
SUM OF ELECTRONIC AND THERMAL ENERGIES: -1563.801108
SUM OF ELECTRONIC AND THERMAL ENTHALPIES: -1563.800164
SUM OF ELECTRONIC AND THERMAL FREE ENERGIES: -1563.887675

==> VITEXIN/WATER/BDE/C5/TS <==
54
XYZ-COORDINATES + ENERGIES
O 0.657232 -1.579297 0.968946
O 0.103914 -2.341448 -2.538365
O 2.947587 -2.446272 -2.340522
O 3.939989 -3.098557 0.298381
O -0.219547 0.740054 -0.238731
O 1.501959 -2.013768 3.622868
O -2.508378 -3.339820 -0.401523
O -4.973456 0.664715 -0.749951
O -3.554808 3.024620 -0.407795
O 5.225697 3.730365 0.131370
C 0.842058 -1.862510 -1.422104
C 2.105966 -2.672919 -1.215628
C -0.046137 -2.028602 -0.186973
C 2.796283 -2.278872 0.080350
C 1.812361 -2.378992 1.244406
C -1.352258 -1.288785 -0.301872
C 2.403930 -1.851747 2.531054

C	-1.418079	0.118906	-0.323952
C	-2.554829	-1.986500	-0.411590
C	-2.599770	0.839503	-0.431407
C	-3.765562	-1.313933	-0.529429
C	-3.809707	0.085121	-0.559937
C	-2.548260	2.304716	-0.389756
C	-0.124039	2.088085	-0.242244
C	-1.224706	2.868073	-0.305629
C	1.265539	2.539192	-0.153625
C	1.627491	3.832995	-0.537658
C	2.252478	1.665843	0.316486
C	2.944479	4.249075	-0.448518
C	3.569718	2.075842	0.412067
C	3.914323	3.368711	0.027341
H	1.105387	-0.811910	-1.558108
H	1.836072	-3.734243	-1.160166
H	-0.264557	-3.091889	-0.075668
H	3.164963	-1.255361	-0.001827
H	1.502389	-3.423345	1.372508
H	2.663482	-0.797772	2.402438
H	3.301965	-2.412488	2.775301
H	0.679230	-2.304214	-3.310493
H	3.726727	-3.005460	-2.247144
H	3.647575	-4.015129	0.371485
H	-4.692781	-1.863858	-0.620421
H	0.710429	-1.501587	3.428209
H	-1.134131	3.942576	-0.265329
H	-3.397491	-3.706610	-0.482674
H	0.886659	4.517078	-0.927232
H	1.983517	0.665470	0.622711
H	3.230469	5.246871	-0.755245
H	4.335600	1.407537	0.781555
H	5.341921	4.641039	-0.163851
H	-5.345509	1.065977	0.256481
O	-5.536594	1.188435	1.468738
O	-4.465290	0.540638	1.993985
H	-4.776044	-0.359214	2.189992

ENERGIES
SCF ENERGY: -1715.78157973
SUM OF ELECTRONIC AND ZERO-POINT ENERGIES: -1715.372872
SUM OF ELECTRONIC AND THERMAL ENERGIES: -1715.341681
SUM OF ELECTRONIC AND THERMAL ENTHALPIES: -1715.340736
SUM OF ELECTRONIC AND THERMAL FREE ENERGIES: -1715.436865

==> VITEXIN/WATER/BDE/C7 <==
50
XYZ-COORDINATES + ENERGIES

O	0.140280	-1.601154	1.024148
O	-1.139553	-2.213777	-2.315840
O	1.613966	-2.905650	-2.591279
O	2.886595	-3.759210	-0.129750
O	-0.417323	0.888011	-0.010479
O	1.301232	-2.208544	3.511770
O	-3.558232	-2.577831	0.110031
O	-5.105524	1.843917	0.183987
O	-3.323873	3.700549	0.123927
O	5.471375	2.871696	-0.236153
C	-0.140735	-1.908709	-1.353782
C	0.947585	-2.963178	-1.335668
C	-0.823202	-1.893190	0.017643
C	1.911550	-2.723591	-0.183722
C	1.137316	-2.624077	1.129205
C	-1.932353	-0.888618	0.075656
C	2.027725	-2.233687	2.285795
C	-1.709433	0.486426	0.062260
C	-3.322588	-1.354625	0.109927
C	-2.743410	1.411797	0.101112
C	-4.384522	-0.387024	0.143337
C	-4.110647	0.941197	0.146515
C	-2.421157	2.836567	0.086116
C	-0.085200	2.191665	-0.020532
C	-1.035238	3.163736	0.038075
C	1.358733	2.392975	-0.085001
C	1.898789	3.647868	-0.384942
C	2.224438	1.317251	0.152926
C	3.268267	3.827495	-0.438957
C	3.594046	1.490910	0.104191
C	4.116376	2.747953	-0.193075
H	0.305189	-0.932309	-1.556838
H	0.480519	-3.946325	-1.205266
H	-1.251105	-2.881165	0.197574
H	2.458903	-1.795631	-0.357372
H	0.645769	-3.581966	1.338657
H	2.472967	-1.255893	2.084118
H	2.819381	-2.968974	2.400413
H	-0.709519	-2.293941	-3.174554
H	2.241114	-3.635821	-2.631334
H	2.435901	-4.597776	0.029027
H	-5.404297	-0.742770	0.168864
H	0.589953	-1.566638	3.417179
H	-0.754355	4.205053	0.055477
H	-4.693006	2.742964	0.171160
H	1.254426	4.489668	-0.594081
H	1.822150	0.343296	0.389970
H	3.689747	4.795335	-0.677807
H	4.266898	0.665834	0.293768
H	5.719783	3.778169	-0.454346

ENERGIES
SCF ENERGY: -1564.21740831
SUM OF ELECTRONIC AND ZERO-POINT ENERGIES: -1563.833473
SUM OF ELECTRONIC AND THERMAL ENERGIES: -1563.805667
SUM OF ELECTRONIC AND THERMAL ENTHALPIES: -1563.804723
SUM OF ELECTRONIC AND THERMAL FREE ENERGIES: -1563.892935

==> VITEXIN/WATER/BDE/C7/TS <==
54
XYZ-COORDINATES + ENERGIES

O	-0.116424	-1.536485	1.226468
O	-1.170786	-1.596182	-2.256603
O	1.256950	-3.041057	-2.334030
O	1.940667	-4.383599	0.142277
O	0.160004	1.073357	0.070718
O	0.556412	-2.584652	3.755199
O	-3.851014	-1.337034	0.529415
O	-4.002562	3.356499	0.684901
O	-1.755136	4.618845	0.450139
O	6.344050	1.238592	-0.581970
C	-0.242649	-1.625621	-1.172261
C	0.478041	-2.959145	-1.146529
C	-1.030982	-1.467073	0.131061
C	1.346828	-3.090669	0.094306
C	0.511739	-2.817678	1.341807
C	-1.803005	-0.181984	0.247834
C	1.359038	-2.764788	2.591273
C	-1.187396	1.065840	0.230759
C	-3.212087	-0.194138	0.452772
C	-1.888402	2.264127	0.368054
C	-3.943534	1.007266	0.589820
C	-3.297733	2.216451	0.550987
C	-1.158762	3.526693	0.330926
C	0.862174	2.223141	0.039634
C	0.256473	3.431029	0.167333
C	2.294089	1.987829	-0.129600
C	3.144075	3.013556	-0.553557
C	2.830180	0.720253	0.129249
C	4.498630	2.783046	-0.710134
C	4.183814	0.484528	-0.020100
C	5.017839	1.517572	-0.441718
H	0.483851	-0.821600	-1.282017
H	-0.271200	-3.759436	-1.135285
H	-1.738875	-2.295695	0.199138
H	2.169986	-2.376630	0.039271
H	-0.262005	-3.588591	1.441184
H	2.084612	-1.952527	2.497369
H	1.889885	-3.704963	2.713193
H	-0.686601	-1.664419	-3.087964
H	1.659223	-3.915863	-2.367095
H	1.236240	-5.041468	0.194972
H	-5.013520	0.956282	0.731080
H	0.079687	-1.754120	3.656019
H	0.839554	4.338531	0.169408
H	-3.360108	4.104091	0.633331
H	2.749824	3.993569	-0.782275
H	2.185883	-0.079736	0.464115

H 5.157396 3.572110 -1.048826
H 4.605731 -0.489683 0.185359
H 6.820242 2.020044 -0.887558
H -4.130656 -1.664934 -0.593432
O -4.255071 -1.700793 -1.758393
O -3.601204 -0.587225 -2.178568
H -2.656471 -0.878510 -2.287904

ENERGIES
SCF ENERGY: -1715.79535689
SUM OF ELECTRONIC AND ZERO-POINT ENERGIES: -1715.386264
SUM OF ELECTRONIC AND THERMAL ENERGIES: -1715.356164
SUM OF ELECTRONIC AND THERMAL ENTHALPIES: -1715.355220
SUM OF ELECTRONIC AND THERMAL FREE ENERGIES: -1715.447671

==> VITEXIN/WATER/PA/C4' <==
50
XYZ-COORDINATES + ENERGIES
O 0.211239 -1.621763 1.019007
O -0.911266 -2.136191 -2.396648
O 1.862066 -2.722135 -2.586208
O 3.058540 -3.658798 -0.112776
O -0.421739 0.904990 -0.016864
O 1.306951 -2.276247 3.522392
O -3.384640 -2.739791 0.080365
O -5.130888 1.667857 0.176378
O -3.438730 3.609585 0.120739
O 5.374042 3.187777 -0.206992
C 0.029939 -1.831896 -1.374275
C 1.151354 -2.850758 -1.358886
C -0.711697 -1.894064 -0.038551
C 2.064796 -2.639292 -0.162723
C 1.238164 -2.611140 1.121382
C -1.867992 -0.936264 0.040075
C 2.074676 -2.232956 2.321653
C -1.699032 0.447243 0.038373
C -3.187127 -1.395555 0.087656
C -2.761475 1.350135 0.085153
C -4.282214 -0.531704 0.134369
C -4.072822 0.831525 0.132783
C -2.492959 2.775635 0.079958
C -0.130325 2.229411 -0.015933
C -1.127416 3.162414 0.035399
C 1.291037 2.483466 -0.066632
C 1.799357 3.785635 -0.240479
C 2.216409 1.429144 0.059744
C 3.152313 4.022494 -0.286362
C 3.572216 1.656332 0.018644
C 4.108294 2.967440 -0.159337
H 0.448707 -0.834718 -1.525039
H 0.710340 -3.852279 -1.289125
H -1.092943 -2.909623 0.082972
H 2.601648 -1.696552 -0.276616
H 0.776111 -3.593985 1.277879
H 2.487790 -1.232390 2.168481
H 2.889733 -2.941574 2.440097
H -0.428414 -2.205863 -3.227557
H 2.512829 -3.430799 -2.632086
H 2.615889 -4.510747 -0.016289
H -5.288991 -0.926036 0.168517
H 0.576818 -1.656465 3.423563
H -0.889691 4.214003 0.053017
H -4.327308 -2.938457 0.136585
H -4.757095 2.586567 0.165113
H 1.122183 4.621787 -0.353715
H 1.854592 0.420867 0.202743
H 3.524042 5.029444 -0.428832
H 4.266170 0.831070 0.123094

ENERGIES
SCF ENERGY: -1564.41289122
SUM OF ELECTRONIC AND ZERO-POINT ENERGIES: -1564.028722
SUM OF ELECTRONIC AND THERMAL ENERGIES: -1564.001211
SUM OF ELECTRONIC AND THERMAL ENTHALPIES: -1564.000267
SUM OF ELECTRONIC AND THERMAL FREE ENERGIES: -1564.086600

==> VITEXIN/WATER/PA/C5 <==
50
XYZ-COORDINATES + ENERGIES
O -0.012928 1.664452 1.001337
O 0.908829 1.892671 -2.510363
O -1.834643 2.615672 -2.566986
O -2.833524 3.753193 -0.099783
O 0.443452 -0.928011 0.006556
O -0.901003 2.556939 3.510553
O 3.565551 2.551574 -0.124157
O 5.156270 -1.885474 0.131053
O 3.180990 -3.892467 0.323890
O -5.536171 -2.682313 -0.143201
C 0.023986 1.702621 -1.410692
C -1.041329 2.779904 -1.394731
C 0.858001 1.804094 -0.131981
C -1.886433 2.689253 -0.136084
C -0.979327 2.712347 1.091633
C 1.974417 0.804779 -0.043950
C -1.751037 2.472061 2.368488
C 1.761334 -0.568107 0.025458
C 3.318670 1.208611 -0.045849
C 2.777067 -1.540311 0.105665
C 4.364164 0.311441 0.014560
C 4.158654 -1.089093 0.088518
C 2.387235 -2.936154 0.205958
C 0.059187 -2.220100 0.064600
C 0.963937 -3.209524 0.173749
C -1.400423 -2.364358 0.007077
C -1.988859 -3.572656 -0.373251
C -2.223559 -1.279476 0.325955
C -3.366933 -3.699635 -0.426571
C -3.601643 -1.398646 0.277107
C -4.171783 -2.610443 -0.100628
H -0.456469 0.725157 -1.474538
H -0.549655 3.759810 -1.415392
H 1.291574 2.805738 -0.107822
H -2.464937 1.764231 -0.150215
H -0.460863 3.678293 1.141256
H -2.226168 1.488600 2.319539
H -2.517569 3.233032 2.486136
H 0.373928 1.932713 -3.310808
H -2.461605 3.345847 -2.608102
H -2.348853 4.587506 -0.099221
H 5.384138 0.675375 0.000133
H -0.199730 1.906682 3.398295
H 0.646301 -4.237762 0.255802
H 4.518110 2.702464 -0.130861
H -1.373373 -4.417432 -0.649333
H -1.782912 -0.340032 0.626626
H -3.824757 -4.632528 -0.729817
H -4.241744 -0.563613 0.527819
H -5.811960 -3.563608 -0.420874

ENERGIES
SCF ENERGY: -1564.40709662
SUM OF ELECTRONIC AND ZERO-POINT ENERGIES: -1564.022563
SUM OF ELECTRONIC AND THERMAL ENERGIES: -1563.994766
SUM OF ELECTRONIC AND THERMAL ENTHALPIES: -1563.993822
SUM OF ELECTRONIC AND THERMAL FREE ENERGIES: -1564.080594

==> VITEXIN/WATER/PA/C7 <==
50
XYZ-COORDINATES + ENERGIES
O 0.098513 -1.709729 0.995816
O -0.825402 -1.991118 -2.511764
O 1.940022 -2.621988 -2.568654
O 2.974803 -3.722625 -0.097544
O -0.449304 0.887346 -0.013621
O 1.010594 -2.564258 3.508857
O -3.515385 -2.690831 -0.106647
O -5.160071 1.714156 0.192456
O -3.420430 3.640506 0.226851
O 5.401855 3.032769 -0.113737
C 0.053011 -1.767066 -1.412893
C 1.153925 -2.807428 -1.394234
C -0.774271 -1.887919 -0.132521
C 1.998147 -2.685789 -0.137348
C 1.094245 -2.728549 1.091761
C -1.923559 -0.929513 -0.046486

C	1.858854	-2.459071	2.366944
C	-1.740193	0.437769	0.013960
C	-3.267336	-1.439118	-0.035570
C	-2.788423	1.371253	0.095760
C	-4.340380	-0.495194	0.051105
C	-4.111449	0.851265	0.113245
C	-2.505696	2.771913	0.155704
C	-0.152897	2.203878	0.042186
C	-1.116789	3.146555	0.136777
C	1.293978	2.441803	-0.003529
C	1.805379	3.703044	-0.320192
C	2.185881	1.397238	0.263931
C	3.171618	3.920578	-0.359382
C	3.553314	1.607291	0.228106
C	4.045658	2.870968	-0.084321
H	0.500576	-0.773962	-1.482540
H	0.694483	-3.802814	-1.410851
H	-1.184923	-2.899313	-0.097070
H	2.549656	-1.744185	-0.156196
H	0.603850	-3.708691	1.147310
H	2.303373	-1.461536	2.313353
H	2.648245	-3.195625	2.488803
H	-0.288148	-2.022957	-3.311004
H	2.581699	-3.338882	-2.615340
H	2.512690	-4.569759	-0.091934
H	-5.355745	-0.868375	0.062603
H	0.282597	-1.945993	3.385509
H	-0.861372	4.192230	0.208693
H	-4.771906	2.622147	0.220462
H	1.140037	4.521688	-0.555533
H	1.807693	0.416448	0.513340
H	3.568124	4.895559	-0.612276
H	4.244973	0.803247	0.439587
H	5.624436	3.943513	-0.341033

ENERGIES
SCF ENERGY: -1564.41782081
SUM OF ELECTRONIC AND ZERO-POINT ENERGIES: -1564.033347
SUM OF ELECTRONIC AND THERMAL ENERGIES: -1564.005855
SUM OF ELECTRONIC AND THERMAL ENTHALPIES: -1564.004911
SUM OF ELECTRONIC AND THERMAL FREE ENERGIES: -1564.091296

==> VITEXIN/WATER/PA/C7/AIP <==
50
XYZ-COORDINATES + ENERGIES

O	0.139793	-1.601665	1.024176
O	-1.137038	-2.212176	-2.317384
O	1.615866	-2.904505	-2.590845
O	2.886450	-3.760081	-0.128539
O	-0.417381	0.888122	-0.010205
O	1.299455	-2.211284	3.512608
O	-3.557850	-2.578214	0.107624
O	-5.105791	1.843239	0.183661
O	-3.324402	3.700152	0.124587
O	5.470936	2.872928	-0.237247
C	-0.139276	-1.907890	-1.353992
C	0.948819	-2.962552	-1.335599
C	-0.822937	-1.893261	0.016793
C	1.912061	-2.723851	-0.182896
C	1.136854	-2.624512	1.129510
C	-1.932195	-0.888799	0.074764
C	2.026382	-2.234396	2.286842
C	-1.709445	0.486274	0.062062
C	-3.322381	-1.354963	0.108395
C	-2.743595	1.411468	0.101110
C	-4.384485	-0.387609	0.142123
C	-4.110766	0.940656	0.145974
C	-2.421554	2.836289	0.086704
C	-0.085460	2.191832	-0.019742
C	-1.035700	3.163702	0.039089
C	1.358423	2.393447	-0.084146
C	1.898168	3.648931	-0.382235
C	2.224436	1.317511	0.151693
C	3.267579	3.828857	-0.436669
C	3.593992	1.491429	0.102416
C	4.115985	2.748993	-0.193244
H	0.306994	-0.931402	-1.555911
H	0.481437	-3.945661	-1.206023
H	-1.250868	-2.881369	0.195855
H	2.460124	-1.796191	-0.355853
H	0.645259	-3.582514	1.338373
H	2.470548	-1.255881	2.086295
H	2.818836	-2.968892	2.401004
H	-0.705093	-2.296759	-3.174749
H	2.241340	-3.636040	-2.631922
H	2.435075	-4.598498	0.029080
H	-5.404209	-0.743512	0.167155
H	0.588310	-1.569089	3.419008
H	-0.755099	4.205086	0.056887
H	-4.693356	2.742375	0.171341
H	1.253623	4.491009	-0.589636
H	1.822439	0.343118	0.387390
H	3.688755	4.797166	-0.674151
H	4.267083	0.666140	0.290201
H	5.719068	3.779453	-0.455557

ENERGIES
SCF ENERGY: -1564.21740791
SUM OF ELECTRONIC AND ZERO-POINT ENERGIES: -1563.833452
SUM OF ELECTRONIC AND THERMAL ENERGIES: -1563.805661
SUM OF ELECTRONIC AND THERMAL ENTHALPIES: -1563.804717
SUM OF ELECTRONIC AND THERMAL FREE ENERGIES: -1563.892878

==> VITEXIN/WATER/PA/C7/BDE/C4' <==
49
XYZ-COORDINATES + ENERGIES

O	0.331515	-1.585007	1.018586
O	-0.810509	-2.269123	-2.364525
O	1.988400	-2.692648	-2.582471
O	3.289273	-3.459584	-0.109087
O	-0.486808	0.857488	-0.051195
O	1.486061	-2.101803	3.516756
O	-3.268311	-2.937822	0.155498
O	-5.248284	1.331988	0.203897
O	-3.660453	3.387934	0.086290
O	5.079532	3.495239	-0.184505
C	0.122895	-1.875889	-1.363942
C	1.305264	-2.823631	-1.338743
C	-0.598359	-1.936981	-0.016747
C	2.222542	-2.516381	-0.167103
C	1.414603	-2.509397	1.128431
C	-1.815301	-1.062904	0.057511
C	2.242401	-2.055592	2.308624
C	-1.737553	0.312544	0.028874
C	-3.117530	-1.672588	0.134410
C	-2.857942	1.167993	0.077565
C	-4.261375	-0.810124	0.183948
C	-4.138725	0.549296	0.155784
C	-2.690894	2.582485	0.048987
C	-0.293154	2.195382	-0.064136
C	-1.330257	3.065312	-0.020498
C	1.115262	2.552123	-0.105351
C	1.523676	3.904270	-0.260782
C	2.093391	1.532116	0.018870
C	2.844147	4.226846	-0.288781
C	3.420646	1.835492	-0.004752
C	3.865113	3.205514	-0.160864
H	0.478412	-0.860678	-1.554222
H	0.929328	-3.847745	-1.229300
H	-0.919685	-2.968346	0.140848
H	2.689250	-1.541684	-0.316232
H	1.014750	-3.514555	1.312595
H	2.602738	-1.039895	2.123327
H	3.093516	-2.717756	2.441059
H	-0.335420	-2.329774	-3.200426
H	2.687254	-3.354917	-2.611808
H	2.913052	-4.337583	0.026420
H	-5.243123	-1.260472	0.242878
H	0.708456	-1.547632	3.391781
H	-1.167723	4.131035	-0.023497
H	-4.936190	2.267524	0.172376
H	0.786099	4.685683	-0.367030
H	1.773358	0.509463	0.144842
H	3.172327	5.249682	-0.410855
H	4.177162	1.069037	0.095470

ENERGIES
SCF ENERGY: -1563.76686120
SUM OF ELECTRONIC AND ZERO-POINT ENERGIES: -1563.395169
SUM OF ELECTRONIC AND THERMAL ENERGIES: -1563.367909
SUM OF ELECTRONIC AND THERMAL ENTHALPIES: -1563.366964
SUM OF ELECTRONIC AND THERMAL FREE ENERGIES: -1563.453796

==> VITEXIN/WATER/PA/C7/BDE/C4'/TS <==
53
XYZ-COORDINATES + ENERGIES

O	0.917306	1.728768	1.013815
O	1.555776	1.722716	-2.569470
O	-0.890539	3.153031	-2.400865
O	-1.331680	4.506181	0.130048
O	0.578979	-0.912805	0.110923
O	0.530006	2.795169	3.579413
O	4.536803	1.583927	-0.498742
O	4.839449	-3.111051	-0.149107
O	2.608592	-4.437508	0.204827
O	-5.592219	-1.261579	0.838502
C	0.755486	1.772707	-1.391330
C	0.019592	3.097090	-1.304801
C	1.696425	1.639204	-0.190818
C	-0.704051	3.229780	0.027287
C	0.281361	2.998254	1.173069
C	2.510280	0.378206	-0.181539
C	-0.410691	2.961767	2.519109
C	1.940389	-0.871904	-0.027257
C	3.939046	0.461009	-0.341612
C	2.670410	-2.079874	-0.005438
C	4.690837	-0.760286	-0.325229
C	4.083615	-1.977127	-0.162132
C	1.997080	-3.328431	0.173076
C	-0.083837	-2.078363	0.281090
C	0.560969	-3.268251	0.323423
C	-1.530987	-1.874295	0.419625
C	-2.424703	-2.955241	0.295980
C	-2.029058	-0.580812	0.668191
C	-3.783004	-2.751538	0.423650
C	-3.386350	-0.367658	0.796750
C	-4.285126	-1.453129	0.680328
H	0.028614	0.955807	-1.395187
H	0.750972	3.911090	-1.390980
H	2.394122	2.480860	-0.217015
H	-1.508967	2.493233	0.080366
H	1.044854	3.788341	1.165066
H	-1.141769	2.147035	2.527859
H	-0.923860	3.903447	2.700210
H	0.974133	1.888594	-3.326915
H	-1.284360	4.038113	-2.421753
H	-0.646144	5.191870	0.076012
H	5.766079	-0.706837	-0.444086
H	1.005339	1.965509	3.422405
H	0.022368	-4.189922	0.486180
H	4.216373	-3.869955	-0.017871
H	-2.057398	-3.949648	0.080991
H	-1.343513	0.249127	0.766112
H	-4.484963	-3.569634	0.323514
H	-3.783602	0.621228	0.989168
H	-6.041664	-1.018679	-0.167952
O	-6.214029	-0.812686	-1.411443
O	-4.937853	-0.908748	-1.871931
H	-4.837447	-1.839569	-2.152420

ENERGIES
SCF ENERGY: -1715.33226400
SUM OF ELECTRONIC AND ZERO-POINT ENERGIES: -1714.936109
SUM OF ELECTRONIC AND THERMAL ENERGIES: -1714.906716
SUM OF ELECTRONIC AND THERMAL ENTHALPIES: -1714.905772
SUM OF ELECTRONIC AND THERMAL FREE ENERGIES: -1714.996462

==> VITEXIN/WATER/PA/C7/BDE/C4'/TS/V2 <==
53
XYZ-COORDINATES + ENERGIES

O	0.917525	1.728800	1.013698
O	1.555567	1.722502	-2.569658
O	-0.890644	3.152908	-2.400834
O	-1.331316	4.506355	0.130033
O	0.578908	-0.912845	0.110822
O	0.530913	2.795456	3.579229
O	4.536877	1.583632	-0.498967
O	4.839265	-3.111337	-0.149000
O	2.608326	-4.437652	0.204963
O	-5.592307	-1.261296	0.838718
C	0.755419	1.772565	-1.391416
C	0.019613	3.096998	-1.304874
C	1.696499	1.639078	-0.191019
C	-0.703830	3.229877	0.027307
C	0.281766	2.998379	1.172938
C	2.510285	0.378041	-0.181705
C	-0.410003	2.962137	2.519130
C	1.940323	-0.872032	-0.027366
C	3.939057	0.460761	-0.341762
C	2.670275	-2.080038	-0.005460
C	4.690780	-0.760575	-0.325273
C	4.083489	-1.977375	-0.162124
C	1.996871	-3.328546	0.173133
C	-0.083966	-2.078354	0.281092
C	0.560766	-3.268274	0.323496
C	-1.531106	-1.874197	0.419690
C	-2.424883	-2.955054	0.295851
C	-2.029090	-0.580727	0.668470
C	-3.783178	-2.751292	0.423576
C	-3.386377	-0.367502	0.797024
C	-4.285215	-1.452903	0.680491
H	0.028508	0.955703	-1.395170
H	0.751040	3.910938	-1.391228
H	2.394242	2.480689	-0.217383
H	-1.508809	2.493415	0.080593
H	1.045357	3.788370	1.164679
H	-1.141224	2.147535	2.528117
H	-0.922961	3.903924	2.700266
H	0.973848	1.888452	-3.327030
H	-1.284642	4.037917	-2.421560
H	-0.645705	5.191968	0.075964
H	5.766029	-0.707194	-0.444104
H	1.006122	1.965743	3.422109
H	0.022112	-4.189898	0.486349
H	4.216149	-3.870203	-0.017743
H	-2.057632	-3.949436	0.080654
H	-1.343497	0.249160	0.766508
H	-4.485188	-3.569324	0.323268
H	-3.783573	0.621389	0.989528
H	-6.041852	-1.018388	-0.167672
O	-6.214331	-0.812495	-1.411168
O	-4.938205	-0.908664	-1.871719
H	-4.837935	-1.839477	-2.152285

ENERGIES
SCF ENERGY: -1715.33226380
SUM OF ELECTRONIC AND ZERO-POINT ENERGIES: -1714.936107
SUM OF ELECTRONIC AND THERMAL ENERGIES: -1714.906715
SUM OF ELECTRONIC AND THERMAL ENTHALPIES: -1714.905771
SUM OF ELECTRONIC AND THERMAL FREE ENERGIES: -1714.996458

==> VITEXIN/WATER/PA/C7/BDE/C5 <==
49
XYZ-COORDINATES + ENERGIES

O	0.143723	-1.594462	1.001084
O	-0.948895	-2.025122	-2.440722
O	1.820778	-2.621449	-2.614458
O	2.994782	-3.611442	-0.154321
O	-0.540590	0.893712	-0.035853
O	1.211028	-2.304741	3.496169
O	-3.504090	-2.736628	0.057317
O	-5.243157	1.573838	0.266474
O	-3.413744	3.731501	0.107006
O	5.373951	2.864708	-0.070547
C	-0.019064	-1.749264	-1.398622
C	1.097286	-2.773665	-1.396275
C	-0.776031	-1.840624	-0.072001
C	2.001102	-2.590846	-0.188433
C	1.163822	-2.591155	1.088640
C	-1.960632	-0.926021	0.029147
C	1.993035	-2.248929	2.305017
C	-1.824489	0.476658	0.035056

C -3.254831 -1.491548 0.084333
C -2.862996 1.412472 0.100031
C -4.353844 -0.558310 0.172926
C -4.206952 0.879493 0.181852
C -2.558390 2.824012 0.080032
C -0.203782 2.209496 -0.026178
C -1.149381 3.163174 0.034764
C 1.249523 2.400095 -0.065398
C 1.806838 3.633168 -0.413237
C 2.100402 1.338821 0.260886
C 3.180187 3.807849 -0.421948
C 3.474084 1.506168 0.256716
C 4.013026 2.743362 -0.082221
H 0.408383 -0.752953 -1.524689
H 0.652363 -3.774791 -1.353418
H -1.151923 -2.862074 0.021501
H 2.537846 -1.645099 -0.275808
H 0.696059 -3.575528 1.216566
H 2.422574 -1.251577 2.176978
H 2.796598 -2.971398 2.418540
H -0.452128 -2.096076 -3.263270
H 2.464073 -3.335965 -2.672432
H 2.550122 -4.464440 -0.079284
H -5.359645 -0.950221 0.236825
H 0.476682 -1.691020 3.389729
H -0.878243 4.207381 0.065967
H 1.172271 4.462542 -0.692517
H 1.683161 0.380954 0.534772
H 3.613779 4.761136 -0.695577
H 4.134403 0.689833 0.516831
H 5.629867 3.763524 -0.308428

ENERGIES
SCF ENERGY: -1563.75657609
SUM OF ELECTRONIC AND ZERO-POINT ENERGIES: -1563.385154
SUM OF ELECTRONIC AND THERMAL ENERGIES: -1563.357530
SUM OF ELECTRONIC AND THERMAL ENTHALPIES: -1563.356585
SUM OF ELECTRONIC AND THERMAL FREE ENERGIES: -1563.444213

==> VITEXIN/WATER/PA/C7/PA/C4' <==
49
XYZ-COORDINATES + ENERGIES
O 0.266957 -1.626201 1.016030
O -0.793584 -2.172485 -2.419647
O 2.005073 -2.627179 -2.577677
O 3.224819 -3.505939 -0.101438
O -0.482763 0.871023 -0.023598
O 1.345618 -2.250054 3.522643
O -3.329485 -2.890871 0.044243
O -5.230050 1.422464 0.186617
O -3.619165 3.442515 0.140854
O 5.213453 3.407202 -0.193346
C 0.117792 -1.827102 -1.380810
C 1.285347 -2.792778 -1.358516
C -0.641397 -1.923966 -0.056456
C 2.175037 -2.543003 -0.152595
C 1.332989 -2.569884 1.120228
C -1.845545 -1.033692 0.022019
C 2.135366 -2.171328 2.337449
C -1.743518 0.345526 0.029576
C -3.155450 -1.621813 0.063618
C -2.845134 1.213929 0.084389
C -4.281977 -0.741945 0.121906
C -4.132905 0.618509 0.131066
C -2.642888 2.633701 0.092054
C -0.255453 2.206982 -0.010506
C -1.289847 3.090103 0.050930
C 1.159393 2.526363 -0.060979
C 1.610965 3.842206 -0.268429
C 2.128274 1.519612 0.099781
C 2.954669 4.137639 -0.311679
C 3.475222 1.805432 0.060917
C 3.954310 3.131554 -0.148664
H 0.492436 -0.811837 -1.527180
H 0.892382 -3.814652 -1.297169
H -0.981961 -2.955331 0.054837
H 2.660332 -1.571162 -0.253270
H 0.914429 -3.575114 1.257480
H 2.514034 -1.154977 2.198790
H 2.973513 -2.850152 2.468484
H -0.297089 -2.207739 -3.244353
H 2.715067 -3.278050 -2.597020
H 2.831621 -4.380224 0.007390
H -5.273435 -1.173729 0.155561
H 0.576747 -1.684446 3.394444
H -1.102352 4.151759 0.082042
H -4.886506 2.351735 0.182719
H 0.898046 4.643507 -0.412159
H 1.809304 0.500857 0.268862
H 3.282053 5.155901 -0.481173
H 4.202901 1.013573 0.192473

ENERGIES
SCF ENERGY: -1563.95042507
SUM OF ELECTRONIC AND ZERO-POINT ENERGIES: -1563.579588
SUM OF ELECTRONIC AND THERMAL ENERGIES: -1563.552123
SUM OF ELECTRONIC AND THERMAL ENTHALPIES: -1563.551179
SUM OF ELECTRONIC AND THERMAL FREE ENERGIES: -1563.638634

==> VITEXIN/WATER/PA/C7/PA/C4'/AIP <==
49
XYZ-COORDINATES + ENERGIES
O 0.331639 -1.584888 1.018537
O -0.810658 -2.269351 -2.364420
O 1.988334 -2.692539 -2.582569
O 3.289322 -3.459474 -0.109236
O -0.486878 0.857459 -0.051154
O 1.486614 -2.101590 3.516694
O -3.268203 -2.937938 0.156070
O -5.248364 1.331809 0.203910
O -3.660631 3.387797 0.085947
O 5.079448 3.495198 -0.184513
C 0.122807 -1.875928 -1.363963
C 1.305266 -2.823559 -1.338819
C -0.598300 -1.936987 -0.016673
C 2.222604 -2.516244 -0.167241
C 1.414782 -2.509194 1.128365
C -1.815283 -1.062970 0.057643
C 2.242711 -2.055254 2.308420
C -1.737589 0.312483 0.028925
C -3.117471 -1.672703 0.134739
C -2.858014 1.167900 0.077621
C -4.261355 -0.810274 0.184163
C -4.138750 0.549143 0.155867
C -2.691037 2.582390 0.048798
C -0.293258 2.195368 -0.064207
C -1.330412 3.065256 -0.020728
C 1.115150 2.552125 -0.105339
C 1.523586 3.904290 -0.260608
C 2.093282 1.532089 0.018774
C 2.844072 4.226840 -0.288623
C 3.420551 1.835451 -0.004866
C 3.865029 3.205475 -0.160898
H 0.478197 -0.860701 -1.554366
H 0.929406 -3.847701 -1.229326
H -0.919553 -2.968362 0.141007
H 2.689309 -1.541555 -0.316442
H 1.015020 -3.514368 1.312648
H 2.602860 -1.039504 2.123055
H 3.093956 -2.717283 2.440695
H -0.335768 -2.329502 -3.200464
H 2.687645 -3.354337 -2.611663
H 2.913064 -4.337453 0.026292
H -5.243090 -1.260641 0.243147
H 0.709414 -1.546745 3.392256
H -1.167867 4.130975 -0.023899
H -4.936309 2.267329 0.172144
H 0.786008 4.685728 -0.366667
H 1.773215 0.509434 0.144659
H 3.172251 5.249685 -0.410560
H 4.177033 1.068958 0.095260

ENERGIES
SCF ENERGY: -1563.76686124
SUM OF ELECTRONIC AND ZERO-POINT ENERGIES: -1563.395174
SUM OF ELECTRONIC AND THERMAL ENERGIES: -1563.367912

SUM OF ELECTRONIC AND THERMAL ENTHALPIES: -1563.366968
SUM OF ELECTRONIC AND THERMAL FREE ENERGIES: -1563.453805

==> VITEXIN/WATER/PA/C7/PA/C4'/BDE/C5 <==
48
XYZ-COORDINATES + ENERGIES
O 0.160084 -1.525396 1.019551
O -1.043910 -2.125076 -2.353685
O 1.707021 -2.827400 -2.562687
O 2.897434 -3.729903 -0.068591
O -0.461068 0.918452 -0.036465
O 1.284098 -2.112817 3.531421
O -3.459331 -2.689554 0.022202
O -5.153077 1.667533 0.307930
O -3.329883 3.774092 0.053499
O 5.430351 2.961320 -0.238679
C -0.073046 -1.827697 -1.359214
C 1.010079 -2.887582 -1.322899
C -0.786738 -1.827436 -0.004303
C 1.950258 -2.668674 -0.147545
C 1.152249 -2.547905 1.149903
C -1.939714 -0.872582 0.049451
C 2.026639 -2.146064 2.314872
C -1.756493 0.531114 0.045709
C -3.299066 -1.426468 0.070811
C -2.793024 1.437824 0.100865
C -4.346524 -0.511584 0.155587
C -4.171897 0.893652 0.194500
C -2.473543 2.867959 0.052439
C -0.111265 2.225776 -0.042601
C -1.076509 3.183614 0.008878
C 1.321012 2.423828 -0.101422
C 1.879886 3.697767 -0.314383
C 2.201601 1.338345 0.060745
C 3.242484 3.878899 -0.360199
C 3.566560 1.509941 0.020146
C 4.154805 2.792029 -0.193860
H 0.378611 -0.850839 -1.543196
H 0.532146 -3.867990 -1.211930
H -1.189340 -2.827665 0.164517
H 2.527399 -1.757479 -0.311322
H 0.656896 -3.503325 1.362557
H 2.469644 -1.167870 2.110243
H 2.819929 -2.876805 2.446504
H -0.584907 -2.209356 -3.196769
H 2.329238 -3.562133 -2.593392
H 2.419325 -4.556489 0.071491
H -5.361531 -0.883201 0.207122
H 0.571597 -1.474876 3.420511
H -0.802749 4.227272 0.017134
H 1.235659 4.554944 -0.459378
H 1.797279 0.351056 0.233652
H 3.654454 4.865288 -0.533077
H 4.226061 0.660906 0.154481

ENERGIES
SCF ENERGY: -1563.28983445
SUM OF ELECTRONIC AND ZERO-POINT ENERGIES: -1562.931747
SUM OF ELECTRONIC AND THERMAL ENERGIES: -1562.904279
SUM OF ELECTRONIC AND THERMAL ENTHALPIES: -1562.903335
SUM OF ELECTRONIC AND THERMAL FREE ENERGIES: -1562.991523

==> VITEXIN/WATER/PA/C7/PA/C4'/PA/C5 <==
48
XYZ-COORDINATES + ENERGIES
O 0.175623 -1.662094 0.997113
O -0.680385 -1.899462 -2.534841
O 2.103193 -2.438085 -2.554386
O 3.132154 -3.559190 -0.093252
O -0.517624 0.892562 0.005988
O 1.090161 -2.510933 3.507086
O -3.352987 -2.850666 -0.137584
O -5.312026 1.410170 0.156845
O -3.522702 3.602080 0.267166
O 5.261589 3.273810 -0.155564
C 0.167438 -1.669237 -1.411596
C 1.302647 -2.672708 -1.397781
C -0.678781 -1.851915 -0.149724
C 2.124267 -2.551252 -0.126652
C 1.207117 -2.642872 1.089709
C -1.887269 -0.972704 -0.057625
C 1.948354 -2.363800 2.376946
C -1.802752 0.405830 0.018045
C -3.196656 -1.567833 -0.060748
C -2.904258 1.291164 0.098777
C -4.309478 -0.703221 0.012730
C -4.234868 0.697362 0.092894
C -2.641417 2.706473 0.181998
C -0.247248 2.216516 0.068668
C -1.253043 3.112405 0.169191
C 1.182444 2.498510 0.016847
C 1.665356 3.787770 -0.259759
C 2.124397 1.480662 0.235020
C 3.018152 4.048979 -0.313314
C 3.480617 1.732345 0.185595
C 3.991799 3.030865 -0.095054
H 0.580431 -0.660383 -1.451202
H 0.876912 -3.682371 -1.442900
H -1.029272 -2.885959 -0.141978
H 2.649563 -1.594960 -0.121898
H 0.754784 -3.642395 1.126202
H 2.359122 -1.351138 2.340479
H 2.760767 -3.074681 2.500444
H -0.126815 -1.889568 -3.323231
H 2.808058 -3.094843 -2.566921
H 2.696460 -4.420044 -0.095088
H -5.296699 -1.150906 0.006499
H 0.341595 -1.918724 3.378337
H -1.039376 4.167010 0.251506
H 0.969880 4.594064 -0.454756
H 1.780326 0.480441 0.459262
H 3.369308 5.048149 -0.540374
H 4.188066 0.930725 0.362058

ENERGIES
SCF ENERGY: -1563.46739349
SUM OF ELECTRONIC AND ZERO-POINT ENERGIES: -1563.108976
SUM OF ELECTRONIC AND THERMAL ENERGIES: -1563.081767
SUM OF ELECTRONIC AND THERMAL ENTHALPIES: -1563.080823
SUM OF ELECTRONIC AND THERMAL FREE ENERGIES: -1563.167353

==> VITEXIN/WATER/PA/C7/PA/C4'/RAF/C1' <==
52
XYZ-COORDINATES + ENERGIES
O -1.163946 -1.387516 1.071742
O -2.696410 -0.934082 -2.198658
O -1.184276 -3.320244 -2.563827
O -0.676802 -4.817967 -0.151008
O 0.131151 0.771989 -0.166834
O -0.674816 -2.635585 3.523842
O -4.521345 0.570332 0.573190
O -2.388954 4.771209 0.598716
O 0.177612 4.813315 0.205243
O 6.216504 -0.520026 1.088382
C -1.702023 -1.389684 -1.285794
C -1.635627 -2.903378 -1.278033
C -2.098773 -0.884888 0.102879
C -0.707508 -3.393459 -0.179401
C -1.159862 -2.812147 1.156215
C -2.173836 0.608559 0.211505
C -0.220343 -3.174004 2.283799
C -1.053450 1.406233 0.089367
C -3.439061 1.241489 0.462831
C -1.064532 2.807865 0.213989
C -3.459924 2.668118 0.586388
C -2.323526 3.418730 0.470046
C 0.147635 3.556753 0.097160
C 1.285258 1.458248 -0.276220
C 1.348899 2.795943 -0.152781
C 2.452074 0.525409 -0.495528
C 3.708034 1.220485 -0.874234
C 2.613051 -0.389627 0.664906
C 4.911367 0.839256 -0.378950
C 3.840163 -0.726654 1.146546
C 5.056563 -0.169328 0.636470
H -0.723166 -0.997146 -1.564533

H	-2.640234	-3.301208	-1.091187
H	-3.087285	-1.288759	0.331722
H	0.312988	-3.067057	-0.389067
H	-2.173802	-3.164210	1.385248
H	0.781359	-2.803167	2.051843
H	-0.182564	-4.253448	2.401095
H	-2.485383	-1.295357	-3.066359
H	-1.173369	-4.283439	-2.576433
H	-1.564468	-5.137660	0.050932
H	-4.406889	3.154645	0.778696
H	-0.740905	-1.680908	3.416190
H	2.293662	3.310740	-0.234079
H	-1.468186	5.108186	0.481859
H	3.640819	1.997739	-1.623497
H	1.710375	-0.808886	1.088805
H	5.815880	1.321068	-0.732368
H	3.918126	-1.427163	1.970431
O	2.031938	-0.409206	-1.607414
O	1.687440	0.349830	-2.767888
H	0.720798	0.376117	-2.730133

ENERGIES
SCF ENERGY: -1714.87808481
SUM OF ELECTRONIC AND ZERO-POINT ENERGIES: -1714.488955
SUM OF ELECTRONIC AND THERMAL ENERGIES: -1714.459084
SUM OF ELECTRONIC AND THERMAL ENTHALPIES: -1714.458139
SUM OF ELECTRONIC AND THERMAL FREE ENERGIES: -1714.550057

==> VITEXIN/WATER/PA/C7/PA/C4'/RAF/C2 <==
52
XYZ-COORDINATES + ENERGIES

O	-0.494337	1.820909	1.068389
O	0.772108	2.455348	-2.283683
O	-2.053259	2.539857	-2.673485
O	-3.563648	3.283828	-0.326933
O	0.458282	-0.627213	0.141333
O	-1.796981	2.429745	3.466371
O	3.054320	3.299481	0.376659
O	5.218783	-0.862601	0.695684
O	3.783710	-2.975294	0.569985
O	-4.894546	-3.625477	0.724953
C	-0.180048	2.000493	-1.325742
C	-1.453574	2.816844	-1.410448
C	0.457408	2.171404	0.054191
C	-2.390900	2.476370	-0.265254
C	-1.653701	2.653680	1.058431
C	1.705254	1.354850	0.227680
C	-2.494450	2.226172	2.239021
C	1.683585	-0.014289	0.219917
C	2.978726	2.034660	0.383529
C	2.848461	-0.811293	0.366193
C	4.155776	1.235324	0.541735
C	4.091605	-0.128508	0.538560
C	2.772155	-2.231616	0.350282
C	0.376319	-1.934384	-0.394886
C	1.500813	-2.802057	0.042741
C	-1.000036	-2.451201	-0.115199
C	-1.252739	-3.598179	0.637379
C	-2.093946	-1.707257	-0.577116
C	-2.550296	-3.997684	0.916913
C	-3.389667	-2.094538	-0.303354
C	-3.675942	-3.261926	0.461706
H	-0.417581	0.950534	-1.500718
H	-1.196444	3.881047	-1.347013
H	0.719626	3.224743	0.171741
H	-2.724921	1.441529	-0.358631
H	-1.349666	3.701942	1.172075
H	-2.763684	1.172884	2.123947
H	-3.400500	2.823848	2.284782
H	0.373803	2.370564	-3.156546
H	-2.846215	3.080980	-2.752452
H	-3.299962	4.208175	-0.244846
H	5.107467	1.733885	0.664747
H	-0.977180	1.926882	3.417328
H	1.411992	-3.875010	-0.018670
H	4.932534	-1.815982	0.691082
H	-0.434984	-4.192865	1.023612
H	-1.918664	-0.811162	-1.160418
H	-2.726317	-4.891637	1.502561
H	-4.222875	-1.507343	-0.670542
O	0.541659	-1.637744	-1.827416
O	0.443484	-2.848692	-2.553641
H	1.368161	-3.061351	-2.745435

ENERGIES
SCF ENERGY: -1714.88508625
SUM OF ELECTRONIC AND ZERO-POINT ENERGIES: -1714.496361
SUM OF ELECTRONIC AND THERMAL ENERGIES: -1714.466364
SUM OF ELECTRONIC AND THERMAL ENTHALPIES: -1714.465420
SUM OF ELECTRONIC AND THERMAL FREE ENERGIES: -1714.558755

==> VITEXIN/WATER/PA/C7/PA/C4'/RAF/C2' <==
52
XYZ-COORDINATES + ENERGIES

O	1.756195	-1.093833	-0.956382
O	2.558648	-0.280142	2.496499
O	1.739886	-2.986835	2.697041
O	2.113202	-4.541816	0.273555
O	-0.191033	0.722510	-0.061148
O	1.943837	-2.413432	-3.420541
O	4.441613	1.586944	0.043662
O	1.456506	5.207992	-0.373640
O	-1.074113	4.673031	-0.424302
O	-5.043849	-3.231610	-0.470475
C	1.899243	-0.978499	1.444754
C	2.257446	-2.450557	1.482061
C	2.378858	-0.378694	0.122784
C	1.697677	-3.178237	0.271271
C	2.139487	-2.467847	-1.005418
C	2.119543	1.091756	-0.012295
C	1.477377	-3.050317	-2.232760
C	0.843568	1.613594	-0.096768
C	3.234591	1.998997	-0.040687
C	0.564885	2.987502	-0.219963
C	2.957620	3.397690	-0.165448
C	1.677865	3.871307	-0.251573
C	-0.786894	3.445614	-0.312967
C	-1.484756	1.121160	-0.164022
C	-1.808993	2.438795	-0.280805
C	-2.413284	0.022559	-0.156442
C	-3.882569	0.310114	-0.178607
C	-1.974477	-1.290894	-0.095850
C	-4.752650	-0.874125	-0.386148
C	-2.862552	-2.352882	-0.148387
C	-4.295931	-2.171919	-0.334267
H	0.816855	-0.867588	1.537601
H	3.349787	-2.544188	1.472030
H	3.458604	-0.529994	0.061584
H	0.608037	-3.192606	0.326254
H	3.231008	-2.535629	-1.098384
H	0.393579	-2.938100	-2.140829
H	1.721332	-4.105399	-2.320808
H	2.333320	-0.719859	3.323650
H	2.047001	-3.895875	2.780497
H	3.075028	-4.565875	0.200389
H	3.789731	4.088522	-0.191203
H	1.757299	-1.472531	-3.335128
H	-2.839523	2.747598	-0.356886
H	0.475070	5.323313	-0.422506
H	-0.914993	-1.492800	-0.028653
H	-5.806213	-0.690808	-0.557212
H	-2.489507	-3.367035	-0.090250
H	-4.119474	1.098434	-0.895616
O	-4.120712	0.926782	1.149970
O	-5.432835	1.469131	1.155412
H	-5.982023	0.719925	1.427489

ENERGIES
SCF ENERGY: -1714.87724077
SUM OF ELECTRONIC AND ZERO-POINT ENERGIES: -1714.488444
SUM OF ELECTRONIC AND THERMAL ENERGIES: -1714.458356
SUM OF ELECTRONIC AND THERMAL ENTHALPIES: -1714.457412
SUM OF ELECTRONIC AND THERMAL FREE ENERGIES: -1714.550282

==> VITEXIN/WATER/PA/C7/PA/C4'/RAF/C3 <==
52

XYZ-COORDINATES + ENERGIES

O	1.756992	-1.046151	0.922147
O	1.075226	-2.359274	-2.400886
O	3.335484	-0.686508	-2.859748
O	5.046769	-0.325404	-0.554399
O	-0.637902	0.132014	-0.050913
O	3.214217	-0.635512	3.286311
O	0.054720	-4.491144	0.408112
O	-4.316023	-2.827065	0.834526
O	-4.665482	-0.279677	0.460691
O	1.435025	6.058503	0.361749
C	1.566268	-1.412154	-1.456184
C	3.068799	-1.262182	-1.583371
C	1.225328	-1.945289	-0.064048
C	3.626117	-0.400171	-0.462976
C	3.181465	-0.960529	0.884853
C	-0.241798	-2.151178	0.170385
C	3.585308	-0.064233	2.032984
C	-1.138376	-1.106120	0.175000
C	-0.728444	-3.490402	0.404289
C	-2.530580	-1.276512	0.397840
C	-2.131509	-3.669197	0.634748
C	-2.992459	-2.612428	0.626902
C	-3.417991	-0.186907	0.323854
C	-1.406722	1.267165	0.055040
C	-2.868239	1.172244	-0.031346
C	-0.688080	2.479220	0.123746
C	-1.346529	3.736796	0.086994
C	0.727927	2.496197	0.232367
C	-0.643568	4.914988	0.168864
C	1.428668	3.676298	0.309621
C	0.778737	4.946018	0.285784
H	1.096199	-0.440400	-1.615014
H	3.527765	-2.256076	-1.521462
H	1.717374	-2.913509	0.049256
H	3.259443	0.621618	-0.572897
H	3.608233	-1.962281	1.022198
H	3.115472	0.914828	1.905664
H	4.664952	0.056769	2.046768
H	1.340758	-2.064499	-3.278541
H	4.290817	-0.639920	-2.973024
H	5.407050	-1.213258	-0.440427
H	-2.504957	-4.669442	0.806756
H	2.261039	-0.771231	3.270521
H	-4.770161	-1.957770	0.750742
H	-2.422836	3.777632	-0.020653
H	1.263264	1.557474	0.262794
H	-1.169246	5.861635	0.136639
H	2.508928	3.659578	0.396300
H	-3.357197	1.934225	0.574931
O	-3.282823	1.429672	-1.422597
O	-4.646235	1.847540	-1.431487
H	-5.122570	1.058929	-1.120651

ENERGIES
SCF ENERGY: -1714.89033163
SUM OF ELECTRONIC AND ZERO-POINT ENERGIES: -1714.500701
SUM OF ELECTRONIC AND THERMAL ENERGIES: -1714.470523
SUM OF ELECTRONIC AND THERMAL ENTHALPIES: -1714.469578
SUM OF ELECTRONIC AND THERMAL FREE ENERGIES: -1714.563316

==> VITEXIN/WATER/PA/C7/PA/C4'/RAF/C3/TS <==
52
XYZ-COORDINATES + ENERGIES

O	-1.732176	-1.161120	-0.929314
O	-0.977068	-2.172584	2.481070
O	-3.308594	-0.578609	2.825401
O	-5.048401	-0.489046	0.511435
O	0.622634	0.181101	-0.099602
O	-3.208085	-0.988898	-3.306969
O	0.107897	-4.498204	-0.126769
O	4.406646	-2.690432	-0.755486
O	4.638640	-0.099694	-0.667570
O	-1.668402	5.979536	-0.232252
C	-1.515168	-1.330693	1.465991
C	-3.022441	-1.237935	1.594830
C	-1.161601	-1.961056	0.118440
C	-3.626145	-0.493671	0.415968
C	-3.159227	-1.133179	-0.888818
C	0.310366	-2.133315	-0.110818
C	-3.603945	-0.343629	-2.098189
C	1.172828	-1.063237	-0.210425
C	0.849333	-3.465844	-0.225627
C	2.561074	-1.193358	-0.423513
C	2.255238	-3.604672	-0.454597
C	3.075425	-2.515928	-0.546660
C	3.391401	-0.043089	-0.484135
C	1.357739	1.317615	-0.218630
C	2.752526	1.248300	-0.262865
C	0.588214	2.511509	-0.219615
C	1.203507	3.788535	-0.171903
C	-0.828828	2.464596	-0.261506
C	0.458254	4.935660	-0.174826
C	-1.581038	3.608367	-0.266652
C	-0.975352	4.907208	-0.225877
H	-1.087551	-0.329151	1.538441
H	-3.435300	-2.253643	1.613775
H	-1.617974	-2.952598	0.085830
H	-3.314737	0.551591	0.445073
H	-3.542517	-2.159712	-0.948072
H	-3.177992	0.662046	-2.045664
H	-4.687911	-0.271617	-2.116360
H	-1.221005	-1.797803	3.334159
H	-4.264068	-0.571156	2.946660
H	-5.352800	-1.404579	0.509894
H	2.668355	-4.600065	-0.546527
H	-2.250666	-1.088500	-3.279455
H	4.812532	-1.792244	-0.777273
H	2.280903	3.867505	-0.116063
H	-1.321937	1.503561	-0.301562
H	0.942058	5.903072	-0.131116
H	-2.661999	3.553639	-0.305723
H	3.300920	2.116102	-0.593328
O	3.403964	1.427816	1.553151
O	4.727906	1.885532	1.437691
H	5.199039	1.127273	1.057577

ENERGIES
SCF ENERGY: -1714.87759064
SUM OF ELECTRONIC AND ZERO-POINT ENERGIES: -1714.489639
SUM OF ELECTRONIC AND THERMAL ENERGIES: -1714.459400
SUM OF ELECTRONIC AND THERMAL ENTHALPIES: -1714.458455
SUM OF ELECTRONIC AND THERMAL FREE ENERGIES: -1714.552030

==> VITEXIN/WATER/PA/C7/PA/C4'/RAF/C3' <==
52
XYZ-COORDINATES + ENERGIES

O	-1.311172	-1.613716	0.940899
O	-2.062173	-1.262723	-2.601647
O	-0.040402	-3.255833	-2.613388
O	0.141715	-4.804543	-0.170952
O	-0.316316	0.879810	0.072870
O	-1.105174	-2.875089	3.428662
O	-4.823392	-0.446996	-0.317854
O	-3.822665	4.136297	0.137204
O	-1.324781	4.799219	0.360302
O	5.726830	-0.533790	0.543890
C	-1.252271	-1.569042	-1.470378
C	-0.867874	-3.034599	-1.474193
C	-2.080954	-1.263998	-0.221480
C	-0.152121	-3.409750	-0.188007
C	-1.007410	-3.006780	1.010121
C	-2.534808	0.161678	-0.122501
C	-0.280145	-3.220228	2.318005
C	-1.641931	1.206640	0.021353
C	-3.937125	0.466129	-0.184463
C	-2.017727	2.557711	0.112751
C	-4.327739	1.840558	-0.093113
C	-3.408502	2.842932	0.050257
C	-1.022969	3.574021	0.274229
C	0.645034	1.815233	0.241894
C	0.340926	3.129721	0.350631
C	1.987297	1.223861	0.299777
C	3.098994	1.999442	0.069628
C	2.113974	-0.150100	0.629489
C	4.465713	1.436403	0.148546

C 3.367956 -0.747144 0.735010
C 4.544096 -0.028637 0.514600
H -0.344494 -0.963083 -1.479355
H -1.780355 -3.636948 -1.556987
H -2.970955 -1.896120 -0.251196
H 0.808497 -2.893867 -0.141650
H -1.942232 -3.581775 1.000959
H 0.633775 -2.619819 2.321284
H -0.018631 -4.268877 2.429422
H -1.575547 -1.527282 -3.389875
H 0.162568 -4.196201 -2.659093
H -0.691426 -5.290472 -0.197259
H -5.381595 2.081131 -0.137812
H -1.374891 -1.957984 3.312231
H 1.117386 3.860237 0.515193
H -3.004758 4.682394 0.237477
H 3.015078 3.045738 -0.187298
H 1.225489 -0.735021 0.811612
H 3.451252 -1.798586 0.981937
H 5.120921 2.023750 0.800800
O 5.059803 1.576429 -1.195693
O 6.480220 1.419412 -1.081738
H 6.535500 0.580649 -0.563763

ENERGIES
SCF ENERGY: -1714.88847572
SUM OF ELECTRONIC AND ZERO-POINT ENERGIES: -1714.499145
SUM OF ELECTRONIC AND THERMAL ENERGIES: -1714.469350
SUM OF ELECTRONIC AND THERMAL ENTHALPIES: -1714.468406
SUM OF ELECTRONIC AND THERMAL FREE ENERGIES: -1714.561256

==> VITEXIN/WATER/PA/C7/PA/C4'/RAF/C3'/TS <==
52
XYZ-COORDINATES + ENERGIES
O 1.147461 1.697979 0.943529
O 1.821407 1.424523 -2.620419
O -0.414332 3.182298 -2.563484
O -0.689667 4.701830 -0.118179
O 0.393953 -0.884685 0.131815
O 0.912381 2.964035 3.434754
O 4.711714 0.899335 -0.487031
O 4.221676 -3.756825 0.061493
O 1.816941 -4.675284 0.391433
O -5.723048 -0.132303 0.843055
C 1.015318 1.639909 -1.465765
C 0.470481 3.053939 -1.453428
C 1.910871 1.428399 -0.243749
C -0.239498 3.349547 -0.143798
C 0.698040 3.050854 1.022589
C 2.514674 0.058516 -0.160394
C 0.005054 3.192615 2.358127
C 1.743212 -1.072256 0.026439
C 3.936058 -0.099114 -0.292573
C 2.261116 -2.376092 0.107996
C 4.472340 -1.423932 -0.206066
C 3.670268 -2.515110 -0.014416
C 1.384446 -3.489815 0.310519
C -0.458645 -1.915519 0.328177
C -0.015529 -3.191298 0.429212
C -1.848086 -1.464926 0.417330
C -2.899303 -2.344008 0.243433
C -2.117579 -0.102049 0.730628
C -4.236070 -1.879116 0.272211
C -3.400158 0.352558 0.886019
C -4.515915 -0.517185 0.713408
H 0.180705 0.936277 -1.449408
H 1.307333 3.754067 -1.563711
H 2.728422 2.150084 -0.303110
H -1.133273 2.728581 -0.064888
H 1.563663 3.723787 0.975569
H -0.829222 2.487340 2.405701
H -0.375348 4.203864 2.472738
H 1.280024 1.609519 -3.395258
H -0.728785 4.092281 -2.591150
H 0.081585 5.277755 -0.182873
H 5.542204 -1.554527 -0.299815
H 1.285923 2.084619 3.314483
H -0.704682 -4.000031 0.616163
H 3.472043 -4.385317 0.201099
H -2.721472 -3.386264 0.018702
H -1.289277 0.576014 0.878732
H -3.593006 1.382185 1.159326
H -5.035024 -2.598407 0.392242
O -4.696486 -1.562222 -1.546264
O -6.108090 -1.451450 -1.578327
H -6.289665 -0.755162 -0.918928

ENERGIES
SCF ENERGY: -1714.87809074
SUM OF ELECTRONIC AND ZERO-POINT ENERGIES: -1714.490155
SUM OF ELECTRONIC AND THERMAL ENERGIES: -1714.460155
SUM OF ELECTRONIC AND THERMAL ENTHALPIES: -1714.459211
SUM OF ELECTRONIC AND THERMAL FREE ENERGIES: -1714.552180

==> VITEXIN/WATER/PA/C7/PA/C4'/RAF/C4 <==
52
XYZ-COORDINATES + ENERGIES
O 1.334035 -1.475341 1.071778
O 1.009709 -1.999404 -2.515177
O 3.588875 -0.828597 -2.303093
O 4.911691 -1.329306 0.233381
O -0.593094 0.382377 0.187954
O 2.406396 -1.845571 3.643404
O -0.895518 -4.299908 -0.567663
O -4.823992 -1.628796 -0.613848
O -4.594933 0.813332 -0.594196
O 2.786461 5.520000 -0.449078
C 1.493387 -1.371287 -1.330276
C 2.994847 -1.546499 -1.223572
C 0.811635 -2.049371 -0.140101
C 3.515975 -1.060686 0.117998
C 2.728133 -1.724528 1.244143
C -0.683596 -1.956414 -0.172781
C 3.099227 -1.166033 2.598178
C -1.357878 -0.745141 -0.047535
C -1.452956 -3.137976 -0.432027
C -2.732550 -0.617185 -0.154227
C -2.863098 -2.997958 -0.573584
C -3.481517 -1.770788 -0.450475
C -3.419891 0.693831 0.065100
C -1.125655 1.620208 -0.074636
C -2.456691 1.814949 -0.182120
C -0.112405 2.648909 -0.179868
C -0.452481 3.990598 -0.516105
C 1.248149 2.325828 0.069426
C 0.505614 4.948361 -0.613239
C 2.221513 3.273965 -0.018298
C 1.899670 4.640797 -0.367614
H 1.251500 -0.306762 -1.343764
H 3.226065 -2.614221 -1.318767
H 1.081433 -3.107147 -0.162182
H 3.409715 0.023187 0.183140
H 2.907954 -2.807071 1.223141
H 2.867762 -0.097678 2.619400
H 4.161689 -1.304877 2.778452
H 1.498581 -1.631549 -3.259243
H 4.534713 -1.010267 -2.299635
H 5.041464 -2.284388 0.186920
H -3.452239 -3.877223 -0.800726
H 1.463904 -1.772729 3.459547
H -2.860888 2.785959 -0.421712
H -4.942428 -0.575618 -0.641471
H -1.482375 4.254993 -0.703617
H 1.504042 1.313009 0.339682
H 0.256841 5.967681 -0.875078
H 3.259033 3.034953 0.173113
O -3.657390 0.656440 1.554974
O -4.280939 1.874744 1.962587
H -3.533774 2.408503 2.264291

ENERGIES
SCF ENERGY: -1714.87473244
SUM OF ELECTRONIC AND ZERO-POINT ENERGIES: -1714.487318
SUM OF ELECTRONIC AND THERMAL ENERGIES: -1714.457467
SUM OF ELECTRONIC AND THERMAL ENTHALPIES: -1714.456523
SUM OF ELECTRONIC AND THERMAL FREE ENERGIES: -1714.548846

==> VITEXIN/WATER/PA/C7/PA/C4'/RAF/C4a <==
52
XYZ-COORDINATES + ENERGIES
O 0.915273 -1.662769 1.077640
O -0.472335 -2.218410 -2.238680
O 2.322149 -2.227529 -2.749275
O 3.956720 -3.010772 -0.492680
O -0.327051 0.738901 0.273402
O 2.386384 -2.241742 3.397554
O -2.540978 -3.423921 0.429400
O -5.045065 0.527046 1.024134
O -3.792375 2.770457 0.853561
O 4.845058 4.170262 -0.386269
C 0.500332 -1.780973 -1.294310
C 1.791906 -2.554789 -1.467688
C -0.065409 -2.049206 0.101007
C 2.776973 -2.221797 -0.360824
C 2.113107 -2.435070 0.997141
C -1.367687 -1.358626 0.372880
C 2.994410 -1.975087 2.135050
C -1.475597 0.018061 0.447128
C -2.559746 -2.146233 0.517531
C -2.680430 0.697634 0.682666
C -3.792357 -1.459054 0.753904
C -3.849750 -0.093593 0.827845
C -2.709554 2.130505 0.683022
C -0.314098 2.093851 0.295162
C -1.463067 2.799080 0.489465
C 1.018249 2.637152 0.104713
C 1.234157 4.017167 -0.063625
C 2.143958 1.793712 0.093172
C 2.501372 4.526759 -0.231216
C 3.417027 2.294335 -0.071229
C 3.657700 3.689301 -0.238552
H 0.699327 -0.714798 -1.421340
H 1.565811 -3.627047 -1.423157
H -0.237981 -3.124107 0.188257
H 3.094778 -1.182514 -0.456246
H 1.866916 -3.497753 1.117849
H 3.191214 -0.905183 2.024934
H 3.936778 -2.515515 2.115704
H -0.097222 -2.104898 -3.118644
H 3.113663 -2.758584 -2.888221
H 3.710245 -3.940343 -0.416292
H -4.697541 -2.042015 0.860411
H 1.531033 -1.799558 3.404507
H -1.448099 3.876996 0.513791
H -4.852196 1.500395 1.006085
H 0.395894 4.701175 -0.072298
H 2.009459 0.729790 0.227984
H 2.645409 5.591789 -0.364532
H 4.268685 1.624672 -0.070025
O -2.998826 0.372851 -2.423349
O -4.147586 0.968906 -2.272615
H -4.736096 0.342485 -1.810808

ENERGIES
SCF ENERGY: -1714.89373335
SUM OF ELECTRONIC AND ZERO-POINT ENERGIES: -1714.506738
SUM OF ELECTRONIC AND THERMAL ENERGIES: -1714.474921
SUM OF ELECTRONIC AND THERMAL ENTHALPIES: -1714.473977
SUM OF ELECTRONIC AND THERMAL FREE ENERGIES: -1714.574090

==> VITEXIN/WATER/PA/C7/PA/C4'/RAF/C4' <==
52
XYZ-COORDINATES + ENERGIES
O 0.936064 1.626432 1.026549
O 2.271637 1.636974 -2.356519
O 0.056554 3.397835 -2.581838
O -0.684353 4.724994 -0.116540
O 0.447455 -0.917442 -0.067431
O 0.180775 2.658819 3.518272
O 4.728911 1.030488 0.219808
O 4.356878 -3.665469 0.249054
O 1.979796 -4.677372 0.081801
O -5.384420 -0.497352 -1.372367
C 1.266584 1.765082 -1.355281
C 0.714012 3.175898 -1.337252
C 1.921486 1.465408 -0.006003
C -0.235380 3.373380 -0.168200
C 0.456335 2.967151 1.130449
C 2.546739 0.105157 0.078414
C -0.493392 2.989281 2.305753
C 1.802042 -1.056614 0.034580
C 3.976914 -0.001576 0.182702
C 2.359115 -2.348307 0.090097
C 4.549922 -1.312563 0.240221
C 3.774648 -2.437394 0.194246
C 1.516090 -3.501112 0.039420
C -0.385346 -1.989473 -0.105575
C 0.103713 -3.260206 -0.058353
C -1.773504 -1.614825 -0.182800
C -2.797423 -2.601831 -0.348310
C -2.146059 -0.236464 -0.091764
C -4.097936 -2.246466 -0.416044
C -3.441309 0.144830 -0.145798
C -4.565918 -0.821221 -0.356622
H 0.452812 1.061446 -1.542898
H 1.550224 3.877184 -1.230508
H 2.712937 2.201556 0.150471
H -1.127458 2.763000 -0.317090
H 1.302958 3.640048 1.316974
H -1.309116 2.285341 2.119425
H -0.904393 3.987146 2.431608
H 1.883009 1.905216 -3.196203
H -0.253011 4.309925 -2.596956
H 0.082958 5.294011 0.018460
H 5.625012 -1.402928 0.319366
H 0.581481 1.791583 3.397035
H -0.558501 -4.110466 -0.080369
H 3.621660 -4.325286 0.198437
H -2.527205 -3.644426 -0.435093
H -1.370581 0.505906 0.023087
H -4.871093 -2.991729 -0.553164
H -3.720427 1.189207 -0.084135
O -5.342019 -0.685344 0.949066
O -6.640716 -1.222537 0.696441
H -6.731763 -0.965758 -0.252114

ENERGIES
SCF ENERGY: -1714.88090434
SUM OF ELECTRONIC AND ZERO-POINT ENERGIES: -1714.492702
SUM OF ELECTRONIC AND THERMAL ENERGIES: -1714.462742
SUM OF ELECTRONIC AND THERMAL ENTHALPIES: -1714.461798
SUM OF ELECTRONIC AND THERMAL FREE ENERGIES: -1714.554791

==> VITEXIN/WATER/PA/C7/PA/C4'/RAF/C5 <==
52
XYZ-COORDINATES + ENERGIES
O 0.812373 -1.613674 1.031923
O -0.440157 -2.140870 -2.343659
O 2.361179 -2.404375 -2.697458
O 3.799337 -3.269754 -0.335200
O -0.200284 0.827203 0.068908
O 2.103149 -2.203081 3.438920
O -2.702573 -3.127939 0.579278
O -4.788135 0.999438 1.155007
O -3.450105 3.208976 0.415574
O 5.371957 3.601461 -0.333205
C 0.513528 -1.764564 -1.355057
C 1.739743 -2.651164 -1.438809
C -0.145747 -1.951123 0.013477
C 2.692169 -2.373156 -0.288873
C 1.944595 -2.483890 1.037173
C -1.407489 -1.168844 0.202496
C 2.804123 -2.058605 2.205460
C -1.419723 0.248114 0.173548
C -2.620480 -1.857545 0.456334
C -2.541076 1.042172 0.264716
C -3.813377 -1.070449 0.600341
C -3.878587 0.378586 0.289870
C -2.433093 2.462741 0.294393
C -0.032259 2.178652 0.079357
C -1.105020 3.000523 0.204359
C 1.365444 2.556635 -0.035557

C	1.750802	3.881153	-0.304596
C	2.380498	1.598293	0.127953
C	3.079363	4.232228	-0.402687
C	3.712063	1.940114	0.033801
C	4.126051	3.276250	-0.237927
H	0.813433	-0.724104	-1.493479
H	1.418976	-3.698235	-1.382946
H	-0.399751	-3.008342	0.115648
H	3.108003	-1.370128	-0.395512
H	1.604479	-3.517946	1.176590
H	3.113802	-1.019612	2.062700
H	3.686909	-2.689281	2.264384
H	0.001753	-2.116347	-3.199444
H	3.124990	-2.986445	-2.772918
H	3.465163	-4.168136	-0.224156
H	-4.735570	-1.570680	0.859130
H	1.298305	-1.678012	3.374759
H	-0.973910	4.070472	0.251104
H	-4.629920	1.959324	1.044496
H	0.997250	4.643715	-0.454025
H	2.111403	0.574229	0.345833
H	3.355606	5.256525	-0.620188
H	4.477375	1.185139	0.169607
O	-4.334954	0.560571	-1.093837
O	-5.618939	-0.032521	-1.257543
H	-5.410264	-0.882342	-1.669866

ENERGIES
SCF ENERGY: -1714.87816953
SUM OF ELECTRONIC AND ZERO-POINT ENERGIES: -1714.488413
SUM OF ELECTRONIC AND THERMAL ENERGIES: -1714.458597
SUM OF ELECTRONIC AND THERMAL ENTHALPIES: -1714.457653
SUM OF ELECTRONIC AND THERMAL FREE ENERGIES: -1714.549637

==> VITEXIN/WATER/PA/C7/PA/C4'/RAF/C5' <==
52
XYZ-COORDINATES + ENERGIES

O	-0.374171	-1.720759	1.039074
O	-1.228233	-1.999751	-2.486705
O	1.497131	-2.793523	-2.467511
O	2.407663	-3.912686	0.044620
O	-0.717256	0.887961	-0.006436
O	0.436336	-2.582147	3.585302
O	-4.019125	-2.473510	-0.141803
O	-5.361600	2.039123	0.068927
O	-3.496265	3.839189	0.141623
O	5.250495	2.748249	0.135504
C	-0.362475	-1.810255	-1.370905
C	0.673254	-2.914768	-1.310723
C	-1.226918	-1.858576	-0.110150
C	1.492555	-2.822515	-0.035202
C	0.558873	-2.793020	1.172327
C	-2.311157	-0.824147	-0.065891
C	1.311272	-2.545516	2.459316
C	-2.035285	0.528653	-0.011417
C	-3.686045	-1.240171	-0.082055
C	-3.018271	1.531866	0.036591
C	-4.693365	-0.223775	-0.032551
C	-4.373683	1.104629	0.023725
C	-2.639175	2.910118	0.098783
C	-0.326960	2.180179	0.060321
C	-1.229585	3.187750	0.120375
C	1.133967	2.315596	0.071825
C	1.725938	3.582149	-0.172244
C	1.912819	1.215578	0.343830
C	3.107783	3.739697	-0.133692
C	3.389353	1.291928	0.353667
C	3.966914	2.669009	0.131895
H	0.146347	-0.847393	-1.445571
H	0.156019	-3.881623	-1.324727
H	-1.703734	-2.840466	-0.072904
H	2.098674	-1.915662	-0.054968
H	0.012144	-3.742736	1.231938
H	1.813488	-1.576097	2.398512
H	2.054647	-3.323555	2.609408
H	-0.679126	-2.049997	-3.276716
H	2.114215	-3.533113	-2.474352
H	1.899958	-4.733123	0.053206
H	-5.732034	-0.526197	-0.042521
H	-0.251470	-1.924691	3.437379
H	-0.906319	4.213650	0.199385
H	-4.910316	2.918028	0.103289
H	1.103778	4.431293	-0.412677
H	1.465609	0.260169	0.573150
H	3.551124	4.706881	-0.334695
H	3.824210	0.834111	1.247687
O	3.863920	0.444011	-0.763409
O	5.254982	0.163459	-0.561573
H	5.612920	1.067150	-0.402658

ENERGIES
SCF ENERGY: -1714.88964304
SUM OF ELECTRONIC AND ZERO-POINT ENERGIES: -1714.500198
SUM OF ELECTRONIC AND THERMAL ENERGIES: -1714.470402
SUM OF ELECTRONIC AND THERMAL ENTHALPIES: -1714.469458
SUM OF ELECTRONIC AND THERMAL FREE ENERGIES: -1714.561702

==> VITEXIN/WATER/PA/C7/PA/C4'/RAF/C5'/TS <==
52
XYZ-COORDINATES + ENERGIES

O	-0.414882	-1.713599	1.071462
O	-1.257749	-2.018807	-2.454896
O	1.455129	-2.847467	-2.419022
O	2.348505	-3.944385	0.110588
O	-0.702135	0.882868	0.020340
O	0.376318	-2.558102	3.627883
O	-4.068796	-2.409845	-0.148455
O	-5.321491	2.131988	-0.024237
O	-3.419051	3.894851	0.062116
O	5.265695	2.588998	0.468068
C	-0.393635	-1.828523	-1.338138
C	0.627881	-2.945151	-1.262623
C	-1.263897	-1.850031	-0.080293
C	1.444360	-2.846547	0.014135
C	0.506061	-2.794533	1.217635
C	-2.329320	-0.795126	-0.057156
C	1.256287	-2.543845	2.505348
C	-2.026941	0.551986	-0.011443
C	-3.711915	-1.183037	-0.096898
C	-2.988777	1.576606	0.001605
C	-4.698391	-0.145144	-0.081103
C	-4.352204	1.177130	-0.035402
C	-2.581497	2.947600	0.050600
C	-0.285367	2.167327	0.080831
C	-1.166821	3.196198	0.098685
C	1.171752	2.265232	0.139696
C	1.821209	3.512989	-0.078032
C	1.927413	1.151182	0.454052
C	3.180191	3.632348	0.032317
C	3.340699	1.215356	0.468346
C	3.999244	2.510084	0.357836
H	0.127937	-0.873087	-1.421546
H	0.098990	-3.905758	-1.266026
H	-1.757805	-2.823109	-0.034940
H	2.059685	-1.945756	-0.014292
H	-0.051129	-3.737741	1.283776
H	1.771299	-1.581690	2.437177
H	1.988839	-3.329929	2.666162
H	-0.706942	-2.079354	-3.243000
H	2.061459	-3.595852	-2.418348
H	1.833232	-4.759964	0.128173
H	-5.742785	-0.425931	-0.109362
H	-0.301961	-1.892967	3.470694
H	-0.823802	4.216198	0.172020
H	-4.853821	3.001944	0.009048
H	1.235435	4.383331	-0.339138
H	1.447411	0.208511	0.671019
H	3.666325	4.586836	-0.121214
H	3.890764	0.433193	0.974772
O	3.900905	0.587797	-1.246121
O	5.281679	0.299934	-1.113244
H	5.645887	1.134664	-0.760815

ENERGIES
SCF ENERGY: -1714.87930433
SUM OF ELECTRONIC AND ZERO-POINT ENERGIES: -1714.491451

SUM OF ELECTRONIC AND THERMAL ENERGIES: -1714.461402
SUM OF ELECTRONIC AND THERMAL ENTHALPIES: -1714.460458
SUM OF ELECTRONIC AND THERMAL FREE ENERGIES: -1714.553536

==> VITEXIN/WATER/PA/C7/PA/C4'/RAF/C6 <==
52
XYZ-COORDINATES + ENERGIES

O	0.567017	-1.608352	1.091986
O	-0.637279	-2.072639	-2.308853
O	2.093417	-2.801765	-2.536766
O	3.281419	-3.795431	-0.084832
O	-0.016536	0.944513	0.003813
O	1.666667	-2.317413	3.577032
O	-3.150608	-2.567404	0.357406
O	-4.664598	1.897875	0.700101
O	-2.866248	3.765986	0.611540
O	5.861277	2.976126	-0.580464
C	0.328599	-1.813783	-1.294467
C	1.396578	-2.888964	-1.296528
C	-0.398053	-1.835203	0.051526
C	2.341195	-2.724350	-0.118576
C	1.539977	-2.649330	1.179200
C	-1.524154	-0.851990	0.158964
C	2.416736	-2.316122	2.363915
C	-1.303073	0.534394	0.155097
C	-2.837862	-1.341890	0.280779
C	-2.322746	1.488973	0.321754
C	-4.004497	-0.368521	0.259605
C	-3.634600	1.038325	0.465067
C	-1.973287	2.885938	0.407096
C	0.342232	2.247114	0.055830
C	-0.598057	3.216399	0.260169
C	1.770007	2.441515	-0.113576
C	2.315554	3.717241	-0.343692
C	2.658117	1.352856	-0.047492
C	3.672014	3.897359	-0.497546
C	4.017089	1.523504	-0.198029
C	4.590355	2.807210	-0.432188
H	0.796374	-0.840785	-1.456226
H	0.905748	-3.866271	-1.216493
H	-0.819245	-2.834301	0.183731
H	2.924902	-1.811247	-0.244338
H	1.030124	-3.606793	1.346175
H	2.878704	-1.338352	2.202249
H	3.195751	-3.065940	2.471193
H	-0.163530	-2.174721	-3.141589
H	2.715660	-3.535625	-2.582629
H	2.795058	-4.623703	0.008251
H	0.950316	-1.682162	3.474354
H	-0.305738	4.252145	0.334229
H	-4.252609	2.798553	0.734755
H	1.664938	4.578802	-0.418327
H	2.268222	0.361822	0.138314
H	4.070715	4.887025	-0.683216
H	4.681838	0.670069	-0.137442
H	-4.773745	-0.699388	0.958797
O	-4.596477	-0.560739	-1.086513
O	-5.962431	-0.164220	-1.055890
H	-5.921201	0.794525	-1.181710

ENERGIES
SCF ENERGY: -1714.88400687
SUM OF ELECTRONIC AND ZERO-POINT ENERGIES: -1714.494433
SUM OF ELECTRONIC AND THERMAL ENERGIES: -1714.464292
SUM OF ELECTRONIC AND THERMAL ENTHALPIES: -1714.463348
SUM OF ELECTRONIC AND THERMAL FREE ENERGIES: -1714.556931

==> VITEXIN/WATER/PA/C7/PA/C4'/RAF/C6' <==
52
XYZ-COORDINATES + ENERGIES

O	-0.169280	-1.698272	1.121529
O	-1.414583	-2.204554	-2.258361
O	1.265995	-3.121702	-2.463786
O	2.388569	-4.103212	0.024092
O	-0.480328	0.854830	0.059618
O	0.867486	-2.408111	3.628981
O	-3.957089	-2.333215	0.184138
O	-5.060007	2.246694	0.185136
O	-3.118093	3.945838	0.106852
O	5.589548	2.384479	0.544446
C	-0.424274	-1.973310	-1.259628
C	0.565782	-3.119301	-1.222023
C	-1.145685	-1.875632	0.084199
C	1.518893	-2.975900	-0.048807
C	0.722437	-2.806303	1.242169
C	-2.164218	-0.775436	0.131974
C	1.619049	-2.503761	2.420707
C	-1.815165	0.561498	0.103353
C	-3.559873	-1.117860	0.172036
C	-2.747900	1.615179	0.123615
C	-4.512541	-0.050154	0.193314
C	-4.123372	1.260178	0.167219
C	-2.304509	2.975163	0.104415
C	-0.025255	2.133405	0.085564
C	-0.887821	3.187706	0.086581
C	1.415634	2.208017	0.127411
C	2.080350	3.392400	0.401290
C	2.207539	0.972803	-0.182469
C	3.461645	3.434115	0.491447
C	3.661281	1.067719	0.083543
C	4.302881	2.250345	0.363283
H	0.109132	-1.043814	-1.463931
H	0.010674	-4.058155	-1.107688
H	-1.665829	-2.820570	0.255018
H	2.159344	-2.106002	-0.203451
H	0.142848	-3.718311	1.434045
H	2.156160	-1.570283	2.230531
H	2.337404	-3.307589	2.555851
H	-0.958814	-2.328950	-3.097799
H	1.852339	-3.885741	-2.476074
H	1.848601	-4.893898	0.143797
H	-5.565049	-0.298114	0.226074
H	0.209220	-1.716504	3.504449
H	-0.519819	4.201333	0.075507
H	-4.560810	3.101381	0.157395
H	1.522245	4.300619	0.583769
H	1.768803	0.104750	0.313534
H	3.955065	4.372611	0.706712
H	4.236122	0.154591	-0.016762
O	1.942039	0.592232	-1.591489
O	2.424199	1.612490	-2.453591
H	1.665447	2.207446	-2.536644

ENERGIES
SCF ENERGY: -1714.87722947
SUM OF ELECTRONIC AND ZERO-POINT ENERGIES: -1714.488690
SUM OF ELECTRONIC AND THERMAL ENERGIES: -1714.458558
SUM OF ELECTRONIC AND THERMAL ENTHALPIES: -1714.457614
SUM OF ELECTRONIC AND THERMAL FREE ENERGIES: -1714.550315

==> VITEXIN/WATER/PA/C7/PA/C4'/RAF/C7 <==
52
XYZ-COORDINATES + ENERGIES

O	-0.086647	-1.597969	0.857460
O	-0.841062	-1.537694	-2.701350
O	1.601546	-2.967018	-2.627167
O	2.105929	-4.376853	-0.139300
O	0.172899	1.066342	-0.119294
O	0.406529	-2.700196	3.397017
O	-3.692841	-1.276824	-1.165761
O	-4.145749	3.204561	-0.107306
O	-1.947797	4.526729	0.051058
O	6.367513	1.605737	0.245569
C	-0.014498	-1.611701	-1.546695
C	0.726715	-2.934091	-1.503623
C	-0.912970	-1.515801	-0.310223
C	1.494736	-3.090950	-0.200978
C	0.558319	-2.867641	0.983486
C	-1.738746	-0.266113	-0.236519
C	1.303754	-2.835204	2.297086
C	-1.195637	0.987888	-0.139426
C	-3.245346	-0.444523	-0.238937
C	-1.964434	2.165487	-0.077490
C	-4.002001	0.851255	-0.188565
C	-3.401470	2.053495	-0.119493
C	-1.289976	3.447130	0.005583

C 0.824411 2.242582 -0.011794
C 0.126383 3.420811 0.045927
C 2.256815 2.088177 0.042206
C 3.122600 3.201380 0.030394
C 2.838117 0.805625 0.120827
C 4.485371 3.044891 0.094661
C 4.200216 0.638764 0.188012
C 5.094036 1.753306 0.179445
H 0.708174 -0.794597 -1.544868
H -0.005016 -3.747299 -1.578450
H -1.596872 -2.367609 -0.323316
H 2.308330 -2.364736 -0.168413
H -0.202690 -3.657692 1.000516
H 2.017114 -2.006795 2.282151
H 1.842478 -3.768077 2.438881
H -0.273658 -1.653943 -3.471319
H 2.024713 -3.831897 -2.652254
H 1.410467 -5.045492 -0.166140
H -5.080688 0.786809 -0.245325
H -0.098691 -1.891605 3.262182
H 0.648647 4.359200 0.139520
H -3.499859 3.951226 -0.047970
H 2.720485 4.203268 -0.038332
H 2.198323 -0.065314 0.140848
H 5.134637 3.911225 0.080095
H 4.625166 -0.355323 0.254608
O -3.375138 -1.058823 1.173563
O -4.741177 -1.374053 1.421674
H -5.071477 -0.581460 1.865000

ENERGIES
SCF ENERGY: -1714.86364997
SUM OF ELECTRONIC AND ZERO-POINT ENERGIES: -1714.475891
SUM OF ELECTRONIC AND THERMAL ENERGIES: -1714.445636
SUM OF ELECTRONIC AND THERMAL ENTHALPIES: -1714.444692
SUM OF ELECTRONIC AND THERMAL FREE ENERGIES: -1714.537657

==> VITEXIN/WATER/PA/C7/PA/C4'/RAF/C8 <==
52
XYZ-COORDINATES + ENERGIES
O 0.094676 -1.729014 0.681465
O -2.366866 -1.096720 -1.906444
O -0.248926 -2.313218 -3.343244
O 1.402857 -4.112366 -1.780128
O 0.150811 1.067629 0.421586
O 1.624215 -3.256313 2.425364
O -3.899667 -1.285613 1.190511
O -4.088718 3.141121 -0.482039
O -1.834184 4.359006 -0.878179
O 6.382102 1.322886 0.230219
C -1.069411 -1.305249 -1.363842
C -0.442633 -2.556935 -1.953715
C -1.197325 -1.475531 0.148040
C 0.867414 -2.898803 -1.262267
C 0.630087 -2.977536 0.240994
C -1.801630 -0.230937 0.840389
C 1.901071 -3.200444 1.027280
C -1.215198 1.049677 0.393588
C -3.325916 -0.218194 0.760517
C -1.941347 2.151288 -0.038111
C -4.011250 0.914257 0.339161
C -3.350304 2.072700 -0.067012
C -1.219252 3.331066 -0.477837
C 0.848900 2.136963 -0.007679
C 0.201172 3.257733 -0.451195
C 2.282906 1.937095 0.057551
C 3.185448 2.998507 -0.137736
C 2.816672 0.660412 0.313963
C 4.545733 2.797875 -0.082459
C 4.175943 0.449228 0.367479
C 5.108694 1.510907 0.173459
H -0.423372 -0.450863 -1.585634
H -1.138628 -3.392833 -1.817189
H -1.846394 -2.329517 0.352717
H 1.605154 -2.122148 -1.470833
H -0.089246 -3.775917 0.462456
H 2.611038 -2.398508 0.810282
H 2.344540 -4.152997 0.750210
H -2.276571 -1.033242 -2.863733
H 0.094465 -3.119619 -3.742785
H 0.777855 -4.824152 -1.596685
H -5.093136 0.892651 0.335015
H 1.147193 -2.450949 2.655316
H 0.763243 4.104972 -0.810832
H -3.438586 3.844900 -0.722201
H 2.815595 3.998664 -0.321143
H 2.145159 -0.173900 0.462870
H 5.224388 3.629393 -0.226320
H 4.565229 -0.543198 0.560595
O -1.486178 -0.309181 2.291969
O -1.850823 -1.591090 2.822588
H -2.760970 -1.704212 2.455140

ENERGIES
SCF ENERGY: -1714.88380973
SUM OF ELECTRONIC AND ZERO-POINT ENERGIES: -1714.494756
SUM OF ELECTRONIC AND THERMAL ENERGIES: -1714.465202
SUM OF ELECTRONIC AND THERMAL ENTHALPIES: -1714.464258
SUM OF ELECTRONIC AND THERMAL FREE ENERGIES: -1714.555721

==> VITEXIN/WATER/PA/C7/PA/C4'/RAF/C8a <==
52
XYZ-COORDINATES + ENERGIES
O 0.454566 -1.581577 1.103036
O -0.914568 -2.407595 -2.162589
O 1.825052 -3.083839 -2.473423
O 3.200325 -3.757532 -0.013974
O -0.292918 0.916999 0.131188
O 1.715902 -1.998391 3.577849
O -3.139360 -2.764621 0.883031
O -4.891713 1.597340 0.865041
O -3.378808 3.552845 0.311561
O 5.425113 3.397834 -0.232567
C 0.103368 -2.030657 -1.235358
C 1.203163 -3.071227 -1.192847
C -0.546724 -1.950121 0.145732
C 2.203248 -2.743935 -0.094659
C 1.474530 -2.574436 1.236118
C -1.700296 -0.999431 0.233968
C 2.397447 -2.086877 2.328825
C -1.562001 0.418907 -0.231358
C -2.999032 -1.519784 0.653272
C -2.658181 1.316334 0.180017
C -4.054282 -0.601924 0.807660
C -3.867030 0.771260 0.606288
C -2.436294 2.709004 0.112889
C -0.056827 2.254980 -0.048190
C -1.084133 3.141073 -0.128450
C 1.361759 2.556852 -0.081829
C 1.837548 3.876757 0.030080
C 2.312682 1.530363 -0.232013
C 3.183339 4.159149 -0.018606
C 3.662736 1.803166 -0.280264
C 4.163592 3.133655 -0.180823
H 0.531222 -1.066389 -1.511206
H 0.757740 -4.050870 -0.984888
H -0.923898 -2.942571 0.397687
H 2.725301 -1.818659 -0.345014
H 1.015148 -3.527603 1.525950
H 2.802883 -1.112069 2.044516
H 3.215308 -2.789698 2.461804
H -0.498237 -2.547318 -3.021530
H 2.472741 -3.796824 -2.484810
H 2.772040 -4.590166 0.219455
H -5.020021 -0.958205 1.137966
H 0.981891 -1.385593 3.464798
H -0.890871 4.193219 -0.267157
H -4.538689 2.524671 0.684113
H 1.139340 4.691588 0.170370
H 1.978548 0.505541 -0.321784
H 3.528517 5.181365 0.075344
H 4.376214 0.997248 -0.403072
O -1.412650 0.415892 -1.709181
O -2.565345 -0.160409 -2.291882
H -2.261624 -1.081875 -2.409972

ENERGIES
SCF ENERGY: -1714.87624966
SUM OF ELECTRONIC AND ZERO-POINT ENERGIES: -1714.487357
SUM OF ELECTRONIC AND THERMAL ENERGIES: -1714.457894
SUM OF ELECTRONIC AND THERMAL ENTHALPIES: -1714.456950
SUM OF ELECTRONIC AND THERMAL FREE ENERGIES: -1714.547863

==> VITEXIN/WATER/PA/C7/PA/C5 <==
49
XYZ-COORDINATES + ENERGIES

O	0.040175	-1.681891	0.992803
O	-0.808782	-1.888674	-2.542959
O	1.942138	-2.568143	-2.548297
O	2.905126	-3.724821	-0.075727
O	-0.485798	0.907413	-0.010997
O	0.889299	-2.587534	3.508662
O	-3.541226	-2.652391	-0.168919
O	-5.237397	1.716135	0.150570
O	-3.314121	3.793972	0.329084
O	5.438640	2.865258	-0.114897
C	0.045646	-1.695163	-1.417871
C	1.127027	-2.755946	-1.393228
C	-0.815614	-1.823175	-0.159881
C	1.947098	-2.669934	-0.118243
C	1.017434	-2.716196	1.091302
C	-1.966807	-0.869339	-0.075621
C	1.761736	-2.479370	2.385133
C	-1.798152	0.499170	0.005607
C	-3.310918	-1.383801	-0.081721
C	-2.843525	1.451973	0.099391
C	-4.369381	-0.454077	0.000316
C	-4.209414	0.938058	0.087867
C	-2.498583	2.845100	0.207840
C	-0.144300	2.210064	0.063220
C	-1.080748	3.166599	0.181372
C	1.311592	2.404194	0.011367
C	1.861363	3.638748	-0.341211
C	2.171331	1.341973	0.308818
C	3.234694	3.813853	-0.386082
C	3.545392	1.508548	0.267398
C	4.075933	2.746486	-0.080730
H	0.511754	-0.709732	-1.462969
H	0.650350	-3.742581	-1.435265
H	-1.231861	-2.832975	-0.152087
H	2.516127	-1.738799	-0.110980
H	0.512610	-3.690400	1.121751
H	2.223269	-1.488522	2.355878
H	2.536583	-3.230509	2.511570
H	-0.250583	-1.925536	-3.327381
H	2.577956	-3.290758	-2.585461
H	2.427761	-4.563237	-0.089636
H	-5.382046	-0.840447	-0.011231
H	0.169558	-1.961655	3.375345
H	-0.798497	4.204367	0.274173
H	1.218708	4.468370	-0.600839
H	1.762579	0.381302	0.586402
H	3.660978	4.768445	-0.667145
H	4.212177	0.689810	0.501927
H	5.687302	3.761678	-0.368881

ENERGIES
SCF ENERGY: -1563.93685713
SUM OF ELECTRONIC AND ZERO-POINT ENERGIES: -1563.565531
SUM OF ELECTRONIC AND THERMAL ENERGIES: -1563.538001
SUM OF ELECTRONIC AND THERMAL ENTHALPIES: -1563.537057
SUM OF ELECTRONIC AND THERMAL FREE ENERGIES: -1563.624496

==> VITEXIN/WATER/PA/C7/RAF/C1' <==
53
XYZ-COORDINATES + ENERGIES

O	1.420171	1.158300	1.083480
O	2.910177	0.388567	-2.146505
O	1.921683	3.026564	-2.548962
O	1.703300	4.611028	-0.140797
O	-0.242258	-0.703220	-0.181241
O	1.130109	2.494780	3.524118
O	4.333883	-1.432689	0.666095
O	1.417498	-5.131132	0.585037
O	-1.100087	-4.659989	0.111347
O	-6.058830	1.334142	1.198988
C	2.006624	1.039245	-1.257255
C	2.250007	2.535006	-1.252082
C	2.261857	0.473454	0.141118
C	1.421346	3.214461	-0.175318
C	1.702877	2.554309	1.170611
C	2.036718	-1.004882	0.249084
C	0.822772	3.101850	2.270457
C	0.785782	-1.564094	0.091202
C	3.145479	-1.877155	0.523271
C	0.514623	-2.941618	0.195124
C	2.880258	-3.280824	0.630499
C	1.621963	-3.790990	0.474171
C	-0.815386	-3.435774	0.025968
C	-1.498090	-1.150375	-0.344755
C	-1.834083	-2.446876	-0.253930
C	-2.456475	0.001345	-0.566383
C	-3.795711	-0.446209	-1.049723
C	-2.514960	0.844831	0.668311
C	-4.947099	0.011021	-0.497697
C	-3.691101	1.258655	1.199919
C	-4.919995	0.869884	0.623388
H	0.976131	0.847981	-1.559917
H	3.310880	2.718552	-1.043915
H	3.304822	0.673165	0.396039
H	0.360426	3.118754	-0.413585
H	2.759586	2.694246	1.431787
H	-0.225319	2.930673	2.011413
H	0.992741	4.168954	2.384036
H	2.777531	0.762464	-3.024299
H	2.146298	3.962703	-2.579255
H	2.637186	4.724695	0.074235
H	3.706881	-3.946738	0.839287
H	1.027511	1.543301	3.415958
H	-2.858243	-2.763039	-0.381341
H	0.454716	-5.284199	0.436425
H	-3.819154	-1.115432	-1.898321
H	-1.568724	1.128751	1.107555
H	-5.906560	-0.288360	-0.901883
H	-3.707540	1.883714	2.083074
H	-6.830597	1.001066	0.724501
O	-1.835471	0.901289	-1.556385
O	-1.495151	0.153911	-2.718517
H	-2.243209	0.314754	-3.310930

ENERGIES
SCF ENERGY: -1715.34078984
SUM OF ELECTRONIC AND ZERO-POINT ENERGIES: -1714.938781
SUM OF ELECTRONIC AND THERMAL ENERGIES: -1714.908573
SUM OF ELECTRONIC AND THERMAL ENTHALPIES: -1714.907629
SUM OF ELECTRONIC AND THERMAL FREE ENERGIES: -1715.000165

==> VITEXIN/WATER/PA/C7/RAF/C2 <==
53
XYZ-COORDINATES + ENERGIES

O	0.351744	-1.839369	1.068563
O	-0.990865	-2.427275	-2.261782
O	1.806942	-2.771028	-2.668556
O	3.275204	-3.582318	-0.316251
O	-0.430858	0.649194	0.105170
O	1.628836	-2.486322	3.468062
O	-3.300498	-3.068108	0.454660
O	-5.153115	1.245805	0.714791
O	-3.572537	3.243739	0.539186
O	5.131104	3.189747	0.684602
C	0.002472	-2.040265	-1.316603
C	1.200677	-2.964258	-1.393628
C	-0.634018	-2.138459	0.070737
C	2.176681	-2.676402	-0.265843
C	1.443032	-2.759554	1.068757
C	-1.817844	-1.231222	0.241835
C	2.327360	-2.369570	2.230282
C	-1.698598	0.130699	0.210702
C	-3.136018	-1.813776	0.430243
C	-2.798770	1.014725	0.358272
C	-4.250163	-0.927675	0.586304
C	-4.086998	0.427186	0.557694

C	-2.620360	2.424865	0.319276
C	-0.262455	1.936131	-0.442670
C	-1.315136	2.894089	-0.012513
C	1.157609	2.345707	-0.151531
C	1.468727	3.389913	0.710029
C	2.188460	1.589022	-0.712660
C	2.794279	3.689840	1.002463
C	3.510989	1.878492	-0.427744
C	3.809763	2.934373	0.431182
H	0.328577	-1.017038	-1.509093
H	0.854082	-4.000351	-1.300806
H	-0.970817	-3.167587	0.210231
H	2.595571	-1.676063	-0.389860
H	1.059481	-3.777553	1.213001
H	2.676982	-1.343933	2.085244
H	3.184339	-3.035182	2.283747
H	-0.585282	-2.411884	-3.135491
H	2.541964	-3.388822	-2.746185
H	2.936140	-4.475870	-0.183370
H	-5.233554	-1.353293	0.731613
H	0.843267	-1.932418	3.407765
H	-1.142145	3.955660	-0.097296
H	-4.795562	2.175739	0.691101
H	0.688661	3.978830	1.172400
H	1.952216	0.768548	-1.377358
H	3.041447	4.504143	1.671527
H	4.316187	1.300367	-0.859948
H	5.209261	3.938976	1.286801
O	-0.427977	1.659377	-1.869460
O	-0.211553	2.859915	-2.587360
H	-1.110388	3.167719	-2.774536

ENERGIES
SCF ENERGY: -1715.35555958
SUM OF ELECTRONIC AND ZERO-POINT ENERGIES: -1714.953996
SUM OF ELECTRONIC AND THERMAL ENERGIES: -1714.923518
SUM OF ELECTRONIC AND THERMAL ENTHALPIES: -1714.922574
SUM OF ELECTRONIC AND THERMAL FREE ENERGIES: -1715.016556

==> VITEXIN/WATER/PA/C7/RAF/C2/TS <==
53
XYZ-COORDINATES + ENERGIES

O	-0.133378	-1.938226	-1.015505
O	1.124640	-2.100977	2.391772
O	-1.601852	-2.836200	2.722477
O	-2.811593	-4.038586	0.378623
O	0.403699	0.690588	-0.188201
O	-1.254634	-2.848088	-3.414432
O	3.593692	-2.771361	-0.174915
O	5.081024	1.666610	-0.642619
O	3.287613	3.541263	-0.603182
O	-5.494557	2.687649	-0.499206
C	0.137114	-1.939125	1.378686
C	-0.918401	-3.021291	1.485988
C	0.843478	-2.062599	0.028241
C	-1.878727	-2.963217	0.309830
C	-1.095366	-2.993309	-0.999999
C	1.945976	-1.064743	-0.168967
C	-1.991127	-2.767468	-2.196002
C	1.713858	0.287130	-0.239950
C	3.310157	-1.534576	-0.252759
C	2.731743	1.253487	-0.396277
C	4.347400	-0.562147	-0.421311
C	4.070735	0.773433	-0.488001
C	2.415585	2.639991	-0.440559
C	0.067559	1.993910	-0.045504
C	1.028433	2.984004	-0.282464
C	-1.383656	2.210119	-0.180866
C	-1.922553	3.490358	-0.313276
C	-2.248026	1.111264	-0.145487
C	-3.293028	3.671027	-0.416525
C	-3.616015	1.282332	-0.252500
C	-4.138206	2.565858	-0.389732
H	-0.338368	-0.960238	1.466542
H	-0.418937	-3.997391	1.481555
H	1.286194	-3.059452	-0.026452
H	-2.467709	-2.046704	0.365336
H	-0.577036	-3.956175	-1.091924
H	-2.468512	-1.787964	-2.103339
H	-2.757864	-3.536300	-2.235853
H	0.674805	-2.091079	3.243528
H	-2.222224	-3.564812	2.832338
H	-2.320740	-4.867339	0.323501
H	5.370847	-0.905244	-0.489217
H	-0.543558	-2.200533	-3.364310
H	0.758739	4.027279	-0.254248
H	4.667548	2.563147	-0.668624
H	-1.287264	4.363227	-0.341806
H	-1.846332	0.114945	-0.039744
H	-3.710629	4.663860	-0.523207
H	-4.285794	0.433385	-0.232803
H	-5.738215	3.614717	-0.606361
O	0.300138	2.028089	1.869272
O	-0.354818	3.087176	2.447299
H	0.343673	3.722568	2.658617

ENERGIES
SCF ENERGY: -1715.33532204
SUM OF ELECTRONIC AND ZERO-POINT ENERGIES: -1714.934795
SUM OF ELECTRONIC AND THERMAL ENERGIES: -1714.904434
SUM OF ELECTRONIC AND THERMAL ENTHALPIES: -1714.903490
SUM OF ELECTRONIC AND THERMAL FREE ENERGIES: -1714.996792

==> VITEXIN/WATER/PA/C7/RAF/C2' <==
53
XYZ-COORDINATES + ENERGIES

O	1.730117	-1.162539	-0.957376
O	2.560175	-0.373518	2.494137
O	1.617251	-3.039092	2.703042
O	1.923256	-4.619323	0.285612
O	-0.134706	0.733538	-0.071461
O	1.858745	-2.500123	-3.416711
O	4.526071	1.406383	0.046438
O	1.696051	5.149379	-0.368004
O	-0.859384	4.714950	-0.428205
O	-5.122014	-3.045281	-0.385577
C	1.872963	-1.045838	1.443483
C	2.162944	-2.532573	1.487576
C	2.382505	-0.473067	0.120720
C	1.572500	-3.237655	0.278086
C	2.048658	-2.553135	-1.000766
C	2.187118	1.007165	-0.017364
C	1.360607	-3.107929	-2.226609
C	0.935470	1.581759	-0.103970
C	3.338724	1.868083	-0.040638
C	0.712503	2.966675	-0.226089
C	3.119860	3.278017	-0.162951
C	1.861690	3.804749	-0.251103
C	-0.617629	3.479620	-0.318476
C	-1.407963	1.186733	-0.169681
C	-1.682358	2.511990	-0.284717
C	-2.382668	0.121395	-0.158272
C	-3.837219	0.482842	-0.153753
C	-1.988315	-1.203425	-0.144769
C	-4.763232	-0.675785	-0.298087
C	-2.914267	-2.237302	-0.192520
C	-4.308541	-1.945869	-0.287832
H	0.796252	-0.885722	1.531978
H	3.249740	-2.676949	1.481368
H	3.454671	-0.672044	0.062377
H	0.483308	-3.200648	0.331530
H	3.135770	-2.672415	-1.092543
H	0.283406	-2.942427	-2.136431
H	1.552889	-4.174019	-2.309835
H	2.317526	-0.802295	3.322125
H	1.882450	-3.960727	2.791730
H	2.883800	-4.690189	0.226142
H	3.980054	3.933703	-0.183149
H	1.731038	-1.549398	-3.331364
H	-2.699630	2.863648	-0.354936
H	0.721860	5.307632	-0.418717
H	-0.936027	-1.444424	-0.122037
H	-5.822063	-0.468087	-0.385981
H	-2.594748	-3.269020	-0.181198
H	-6.043530	-2.766786	-0.447159
H	-4.054227	1.236857	-0.913008

O	-4.049971	1.143921	1.135524
O	-5.287469	1.836294	1.073902
H	-5.907603	1.217879	1.484917

ENERGIES
SCF ENERGY: -1715.34825298
SUM OF ELECTRONIC AND ZERO-POINT ENERGIES: -1714.946557
SUM OF ELECTRONIC AND THERMAL ENERGIES: -1714.916028
SUM OF ELECTRONIC AND THERMAL ENTHALPIES: -1714.915083
SUM OF ELECTRONIC AND THERMAL FREE ENERGIES: -1715.008609

==> VITEXIN/WATER/PA/C7/RAF/C3 <==
53
XYZ-COORDINATES + ENERGIES

O	1.735275	-1.111693	0.935293
O	1.094764	-2.261703	-2.454383
O	3.387373	-0.605850	-2.796133
O	5.065395	-0.387113	-0.446139
O	-0.645581	0.105785	-0.021851
O	3.146669	-0.810884	3.342067
O	0.046858	-4.529499	0.245807
O	-4.331764	-2.897391	0.692066
O	-4.679220	-0.328599	0.432900
O	1.515638	5.993055	0.237925
C	1.583604	-1.370493	-1.456705
C	3.090406	-1.235337	-1.552496
C	1.218479	-1.965856	-0.096524
C	3.642045	-0.434013	-0.385222
C	3.161495	-1.040744	0.930021
C	-0.252164	-2.182674	0.108525
C	3.549520	-0.194680	2.120580
C	-1.151442	-1.146421	0.147813
C	-0.739736	-3.535613	0.277567
C	-2.545571	-1.325936	0.347663
C	-2.144515	-3.726334	0.481239
C	-3.007931	-2.671588	0.509306
C	-3.430982	-0.234521	0.325348
C	-1.406702	1.229393	0.091374
C	-2.877910	1.148159	0.071782
C	-0.679917	2.449607	0.132113
C	-1.342628	3.696315	0.127820
C	0.733056	2.447862	0.174911
C	-0.627497	4.878209	0.169514
C	1.444124	3.629507	0.211497
C	0.766005	4.847707	0.207421
H	1.128289	-0.385757	-1.572538
H	3.533982	-2.237801	-1.528931
H	1.707623	-2.938934	-0.018204
H	3.300420	0.599450	-0.459944
H	3.573784	-2.052110	1.036542
H	3.090905	0.793041	2.022007
H	4.629687	-0.084550	2.161190
H	1.346249	-1.907719	-3.314079
H	4.345117	-0.569292	-2.891831
H	5.401465	-1.288804	-0.375854
H	-2.517757	-4.733739	0.604409
H	2.193793	-0.942675	3.297788
H	-4.789566	-2.028493	0.644143
H	-2.421642	3.742280	0.078812
H	1.263357	1.507447	0.184955
H	-1.140407	5.831772	0.163573
H	2.525488	3.624740	0.244544
H	0.936086	6.763177	0.216948
H	-3.323755	1.850843	0.776860
O	-3.340457	1.539667	-1.258969
O	-4.682548	2.003926	-1.162138
H	-5.172774	1.204214	-0.903240

ENERGIES
SCF ENERGY: -1715.35904612
SUM OF ELECTRONIC AND ZERO-POINT ENERGIES: -1714.956339
SUM OF ELECTRONIC AND THERMAL ENERGIES: -1714.925754
SUM OF ELECTRONIC AND THERMAL ENTHALPIES: -1714.924810
SUM OF ELECTRONIC AND THERMAL FREE ENERGIES: -1715.019045

==> VITEXIN/WATER/PA/C7/RAF/C3/TS <==
53
XYZ-COORDINATES + ENERGIES

O	-1.541408	-1.361240	-0.925305
O	-0.737915	-2.298367	2.494783
O	-3.237000	-0.980403	2.803441
O	-4.933882	-1.051494	0.456955
O	0.617810	0.218926	-0.086571
O	-2.970751	-1.313184	-3.327656
O	0.651505	-4.486541	-0.083185
O	4.711402	-2.199277	-0.729329
O	4.627485	0.416478	-0.702116
O	-2.466190	5.633863	-0.186849
C	-1.348905	-1.519031	1.471607
C	-2.859552	-1.593699	1.574071
C	-0.908045	-2.102580	0.128706
C	-3.517882	-0.906888	0.389256
C	-2.964080	-1.482712	-0.910879
C	0.577466	-2.114252	-0.079920
C	-3.467941	-0.733779	-2.123238
C	1.312474	-0.957422	-0.188754
C	1.268354	-3.377821	-0.183582
C	2.705583	-0.921607	-0.405164
C	2.682166	-3.351807	-0.407012
C	3.370540	-2.176740	-0.515446
C	3.390114	0.318034	-0.503124
C	1.209498	1.418433	-0.226816
C	2.591861	1.529125	-0.303598
C	0.277978	2.525024	-0.216580
C	0.724839	3.846316	-0.070606
C	-1.100001	2.284146	-0.343056
C	-0.174976	4.895232	-0.061541
C	-2.002180	3.328644	-0.336017
C	-1.539074	4.635951	-0.194080
H	-1.035948	-0.476100	1.550831
H	-3.158959	-2.648531	1.578037
H	-1.256292	-3.136510	0.085711
H	-3.317152	0.164823	0.431631
H	-3.236414	-2.543209	-0.984383
H	-3.163216	0.313839	-2.049885
H	-4.552200	-0.787831	-2.167807
H	-1.060516	-1.974022	3.342599
H	-4.191422	-1.067337	2.900513
H	-5.142042	-1.992495	0.412366
H	3.208571	-4.292968	-0.490547
H	-2.009275	-1.306731	-3.273616
H	5.012394	-1.264068	-0.780487
H	1.776810	4.060446	0.055022
H	-1.457700	1.271766	-0.460260
H	0.166108	5.915389	0.058346
H	-3.063628	3.148957	-0.439478
H	-2.036302	6.490392	-0.076028
H	3.017207	2.456986	-0.653372
O	3.216871	1.813125	1.507353
O	4.447162	2.442536	1.389561
H	5.049816	1.736434	1.106781

ENERGIES
SCF ENERGY: -1715.34065563
SUM OF ELECTRONIC AND ZERO-POINT ENERGIES: -1714.939452
SUM OF ELECTRONIC AND THERMAL ENERGIES: -1714.909128
SUM OF ELECTRONIC AND THERMAL ENTHALPIES: -1714.908184
SUM OF ELECTRONIC AND THERMAL FREE ENERGIES: -1715.001674

==> VITEXIN/WATER/PA/C7/RAF/C3' <==
53
XYZ-COORDINATES + ENERGIES

O	-1.264816	-1.676931	0.924046
O	-1.945153	-1.348844	-2.635184
O	0.244551	-3.167922	-2.604220
O	0.492566	-4.714013	-0.173945
O	-0.385799	0.868466	0.111526
O	-1.087605	-2.992240	3.394504
O	-4.798625	-0.692195	-0.440520
O	-4.067765	3.928205	0.125592
O	-1.612154	4.722547	0.433747
O	5.716131	-0.237433	0.837519
C	-1.136583	-1.597148	-1.488849
C	-0.633959	-3.026718	-1.490517
C	-2.016623	-1.361674	-0.259459
C	0.070306	-3.352901	-0.185025

C	-0.861921	-3.045414	0.982821
C	-2.554879	0.034473	-0.157021
C	-0.181847	-3.233048	2.319357
C	-1.726585	1.123603	0.028642
C	-3.969648	0.263619	-0.259088
C	-2.176563	2.452075	0.132017
C	-4.436918	1.613732	-0.155054
C	-3.579666	2.661943	0.032208
C	-1.246239	3.518847	0.335511
C	0.511427	1.856159	0.308483
C	0.142918	3.147022	0.435218
C	1.887393	1.332717	0.373217
C	2.944944	2.134110	0.058439
C	2.085476	-0.014418	0.781650
C	4.342886	1.630903	0.077657
C	3.381312	-0.525463	0.922588
C	4.466801	0.250801	0.631884
H	-0.279349	-0.921620	-1.477051
H	-1.490130	-3.702013	-1.605580
H	-2.865270	-2.045690	-0.324700
H	0.976195	-2.750500	-0.097882
H	-1.749461	-3.687931	0.920568
H	0.675724	-2.557634	2.383737
H	0.163303	-4.258428	2.418750
H	-1.418810	-1.551992	-3.415870
H	0.520172	-4.089441	-2.653353
H	-0.290471	-5.273916	-0.236940
H	-5.500419	1.797802	-0.229205
H	-1.436506	-2.101621	3.283066
H	0.879213	3.913108	0.623376
H	-3.288351	4.519372	0.258389
H	2.806467	3.153565	-0.271100
H	1.234818	-0.632957	1.019730
H	3.536218	-1.533063	1.284939
H	6.376125	0.381394	0.496160
H	5.015274	2.319238	0.598188
O	4.764986	1.660512	-1.327550
O	6.187191	1.543772	-1.360760
H	6.315005	0.691613	-1.801329

ENERGIES
SCF ENERGY: -1715.34398089
SUM OF ELECTRONIC AND ZERO-POINT ENERGIES: -1714.941787
SUM OF ELECTRONIC AND THERMAL ENERGIES: -1714.911429
SUM OF ELECTRONIC AND THERMAL ENTHALPIES: -1714.910485
SUM OF ELECTRONIC AND THERMAL FREE ENERGIES: -1715.003714

==> VITEXIN/WATER/PA/C7/RAF/C4 <==
53
XYZ-COORDINATES + ENERGIES

O	1.078218	-1.579399	1.071684
O	0.610332	-2.381859	-2.440799
O	3.353508	-1.582546	-2.362161
O	4.633305	-1.944747	0.204586
O	-0.511288	0.422337	0.075939
O	2.151786	-1.806213	3.665312
O	-1.496714	-4.157678	-0.356916
O	-5.007361	-1.023003	-0.434046
O	-4.317007	1.480744	-0.821790
O	3.702426	4.998599	-0.395994
C	1.219364	-1.723398	-1.337654
C	2.683230	-2.096319	-1.216447
C	0.488088	-2.190337	-0.074910
C	3.276198	-1.535679	0.066765
C	2.437901	-1.980632	1.262711
C	-0.974123	-1.871554	-0.125910
C	2.897283	-1.330919	2.547151
C	-1.454223	-0.556486	-0.054817
C	-1.941168	-2.977739	-0.300244
C	-2.792740	-0.269001	-0.135858
C	-3.308720	-2.659791	-0.404259
C	-3.777261	-1.349208	-0.334188
C	-3.262211	1.145980	0.034284
C	-0.872590	1.732664	-0.097003
C	-2.150168	2.115719	-0.146975
C	0.312214	2.601973	-0.197279
C	0.222099	3.891478	-0.726053
C	1.555427	2.140950	0.245852
C	1.340579	4.705414	-0.800678
C	2.678747	2.949258	0.176968
C	2.568828	4.232860	-0.344945
H	1.129330	-0.640229	-1.441121
H	2.766801	-3.189189	-1.194276
H	0.596674	-3.273535	-0.001190
H	3.288419	-0.445673	0.013756
H	2.482013	-3.072790	1.355283
H	2.797939	-0.246197	2.454933
H	3.938801	-1.579504	2.731037
H	1.063986	-2.099909	-3.242536
H	4.277495	-1.850859	-2.310698
H	4.655476	-2.906784	0.275889
H	-4.018152	-3.463457	-0.547017
H	1.227694	-1.580069	3.518099
H	-2.407952	3.154237	-0.285463
H	-4.905090	0.693314	-0.804974
H	-0.721122	4.266162	-1.098606
H	1.646974	1.146774	0.658737
H	1.270518	5.702660	-1.215817
H	3.639775	2.595396	0.524907
H	3.499451	5.861418	-0.775520
O	-3.743643	1.146726	1.410837
O	-4.168395	2.458380	1.754284
H	-3.390894	2.826107	2.198018

ENERGIES
SCF ENERGY: -1715.34848655
SUM OF ELECTRONIC AND ZERO-POINT ENERGIES: -1714.945747
SUM OF ELECTRONIC AND THERMAL ENERGIES: -1714.915506
SUM OF ELECTRONIC AND THERMAL ENTHALPIES: -1714.914562
SUM OF ELECTRONIC AND THERMAL FREE ENERGIES: -1715.007294

==> VITEXIN/WATER/PA/C7/RAF/C4a <==
53
XYZ-COORDINATES + ENERGIES

O	0.851206	-1.541597	1.033115
O	-0.238165	-2.677168	-2.241073
O	2.593614	-2.629169	-2.528338
O	4.046605	-3.012287	-0.042962
O	-0.408937	0.647734	-0.170996
O	2.127765	-1.644073	3.521045
O	-2.467670	-3.553960	0.376855
O	-5.146765	0.311965	0.547667
O	-3.800552	2.369750	1.238265
O	4.699694	4.242944	-0.453179
C	0.640464	-2.056279	-1.309076
C	1.968176	-2.785958	-1.257874
C	-0.023037	-2.134430	0.065590
C	2.846540	-2.249289	-0.139227
C	2.075817	-2.262940	1.178719
C	-1.369980	-1.472401	0.115226
C	2.841114	-1.579170	2.287955
C	-1.520429	-0.114828	0.015166
C	-2.539940	-2.295732	0.302364
C	-2.817822	0.579984	-0.038096
C	-3.819292	-1.635353	0.466121
C	-3.951205	-0.300768	0.369767
C	-2.758816	1.895516	0.693247
C	-0.393651	1.955250	0.160333
C	-1.512781	2.557508	0.667714
C	0.916810	2.570428	-0.021354
C	1.077285	3.960052	0.034534
C	2.048739	1.772933	-0.239376
C	2.328621	4.534519	-0.106493
C	3.301988	2.340685	-0.383300
C	3.442515	3.723497	-0.311728
H	0.809634	-1.012812	-1.583508
H	1.773403	-3.848276	-1.068544
H	-0.159861	-3.189065	0.313629
H	3.153380	-1.229187	-0.374600
H	1.851390	-3.299953	1.458202
H	3.022931	-0.538110	2.007302
H	3.793106	-2.079129	2.443702
H	0.185350	-2.654572	-3.105950
H	3.388308	-3.172937	-2.542097
H	3.810088	-3.925649	0.159646
H	-4.678173	-2.248088	0.705777

H 1.269789 -1.230116 3.379420
H -1.459911 3.566224 1.047525
H -4.928421 1.245834 0.814602
H 0.222486 4.605669 0.177871
H 1.946455 0.699144 -0.285878
H 2.448692 5.609531 -0.064824
H 4.175739 1.724137 -0.545401
H 4.671451 5.203094 -0.367132
O -2.980091 0.868724 -1.538828
O -3.927743 1.906790 -1.753783
H -4.774209 1.437713 -1.773316

ENERGIES
SCF ENERGY: -1715.33218308
SUM OF ELECTRONIC AND ZERO-POINT ENERGIES: -1714.931371
SUM OF ELECTRONIC AND THERMAL ENERGIES: -1714.900701
SUM OF ELECTRONIC AND THERMAL ENTHALPIES: -1714.899756
SUM OF ELECTRONIC AND THERMAL FREE ENERGIES: -1714.993952

==> VITEXIN/WATER/PA/C7/RAF/C4' <==
53
XYZ-COORDINATES + ENERGIES
O 0.981507 1.614421 1.032867
O 2.324959 1.615462 -2.346668
O 0.138630 3.411296 -2.570912
O -0.586082 4.743243 -0.101180
O 0.447471 -0.913336 -0.056308
O 0.244130 2.649629 3.527035
O 4.766671 0.950069 0.206237
O 4.307695 -3.736395 0.230031
O 1.906606 -4.703225 0.078394
O -5.313873 -0.340830 -1.428326
C 1.320162 1.757058 -1.347311
C 0.790891 3.176900 -1.325741
C 1.966248 1.440165 0.002240
C -0.156672 3.385692 -0.157180
C 0.527105 2.963858 1.140818
C 2.566135 0.068018 0.080759
C -0.422275 3.000453 2.316065
C 1.800292 -1.078627 0.038702
C 3.995503 -0.066263 0.174296
C 2.332835 -2.382090 0.088510
C 4.543974 -1.388503 0.225865
C 3.748078 -2.498137 0.182800
C 1.469939 -3.518217 0.041159
C -0.402315 -1.969830 -0.094399
C 0.058971 -3.249078 -0.049667
C -1.783789 -1.569954 -0.173197
C -2.826313 -2.535237 -0.343913
C -2.131239 -0.185529 -0.095013
C -4.119204 -2.164674 -0.417145
C -3.413834 0.224045 -0.161773
C -4.546856 -0.735511 -0.314577
H 0.494636 1.068230 -1.539499
H 1.638017 3.864442 -1.216567
H 2.770632 2.160929 0.163928
H -1.057362 2.788948 -0.309274
H 1.386274 3.619932 1.329319
H -1.252932 2.315095 2.126095
H -0.811835 4.006265 2.446609
H 1.945725 1.902748 -3.184406
H -0.136364 4.334076 -2.595514
H 0.190168 5.301474 0.027143
H 5.617731 -1.498983 0.296560
H 0.626423 1.774692 3.401532
H -0.619008 -4.086844 -0.072506
H 3.562350 -4.383629 0.183549
H -2.578973 -3.582867 -0.431731
H -1.345173 0.545047 0.019796
H -4.902356 -2.896015 -0.563837
H -3.679210 1.271388 -0.109461
H -5.921651 -1.053234 -1.664689
O -5.323301 -0.529605 0.889526
O -6.480572 -1.352588 0.836441
H -7.143625 -0.764054 0.448069

ENERGIES
SCF ENERGY: -1715.35279392
SUM OF ELECTRONIC AND ZERO-POINT ENERGIES: -1714.950309
SUM OF ELECTRONIC AND THERMAL ENERGIES: -1714.919969
SUM OF ELECTRONIC AND THERMAL ENTHALPIES: -1714.919025
SUM OF ELECTRONIC AND THERMAL FREE ENERGIES: -1715.012151

==> VITEXIN/WATER/PA/C7/RAF/C5 <==
53
XYZ-COORDINATES + ENERGIES
O 0.742890 -1.641893 1.026900
O -0.528621 -2.175268 -2.340589
O 2.264059 -2.504687 -2.698908
O 3.695590 -3.363765 -0.331178
O -0.198439 0.809924 0.055149
O 2.020810 -2.250683 3.436952
O -2.820936 -3.060391 0.578098
O -4.778588 1.125001 1.162141
O -3.368428 3.296004 0.409619
O 5.471247 3.380406 -0.287917
C 0.435380 -1.808859 -1.358761
C 1.642097 -2.722519 -1.435386
C -0.225063 -1.964443 0.013213
C 2.602848 -2.449897 -0.290904
C 1.855131 -2.537451 1.036632
C -1.464248 -1.145487 0.197648
C 2.724152 -2.126006 2.202755
C -1.434486 0.266923 0.168197
C -2.698328 -1.796622 0.456563
C -2.532315 1.099493 0.265569
C -3.867409 -0.972039 0.609451
C -3.890398 0.477341 0.295020
C -2.384194 2.511887 0.292252
C 0.001638 2.152597 0.070040
C -1.031548 3.009173 0.199183
C 1.423775 2.493187 -0.041438
C 1.831398 3.789642 -0.365828
C 2.396488 1.513674 0.184940
C 3.176757 4.104503 -0.451741
C 3.743480 1.820554 0.100809
C 4.132267 3.118374 -0.216100
H 0.758135 -0.777001 -1.510434
H 1.299553 -3.761709 -1.365624
H -0.506758 -3.013496 0.126049
H 3.033871 -1.454123 -0.406305
H 1.491887 -3.562794 1.181781
H 3.056149 -1.094493 2.056171
H 3.593124 -2.775388 2.263757
H -0.084614 -2.184457 -3.195696
H 2.992140 -3.129311 -2.785728
H 3.348951 -4.255427 -0.205508
H -4.801175 -1.443901 0.879410
H 1.223602 -1.714576 3.368610
H -0.864208 4.074210 0.243929
H -4.599166 2.079856 1.049719
H 1.100443 4.560533 -0.565611
H 2.096395 0.508353 0.442114
H 3.492967 5.107554 -0.707605
H 4.497836 1.066596 0.281142
H 5.613831 4.310746 -0.497980
O -4.342937 0.669290 -1.086940
O -5.644685 0.115189 -1.245997
H -5.463201 -0.738324 -1.663535

ENERGIES
SCF ENERGY: -1715.34603730
SUM OF ELECTRONIC AND ZERO-POINT ENERGIES: -1714.943044
SUM OF ELECTRONIC AND THERMAL ENERGIES: -1714.912841
SUM OF ELECTRONIC AND THERMAL ENTHALPIES: -1714.911897
SUM OF ELECTRONIC AND THERMAL FREE ENERGIES: -1715.005016

==> VITEXIN/WATER/PA/C7/RAF/C5' <==
53
XYZ-COORDINATES + ENERGIES
O -0.386050 -1.718277 1.035036
O -1.264287 -1.999280 -2.484551
O 1.465395 -2.778744 -2.485410
O 2.400843 -3.893304 0.018773
O -0.735779 0.885212 -0.008588
O 0.443173 -2.582587 3.574560

O	-4.040804	-2.470443	-0.117258
O	-5.380332	2.041070	0.095620
O	-3.506419	3.843050	0.146027
O	5.256774	2.698335	0.175232
C	-0.391216	-1.806422	-1.375333
C	0.650287	-2.905711	-1.322970
C	-1.246252	-1.857555	-0.108159
C	1.477750	-2.809619	-0.053089
C	0.551763	-2.787600	1.160426
C	-2.331037	-0.823759	-0.056137
C	1.310871	-2.539851	2.443341
C	-2.054778	0.527690	-0.004862
C	-3.706979	-1.238727	-0.061704
C	-3.036152	1.533978	0.048037
C	-4.713214	-0.220696	-0.005970
C	-4.392888	1.107109	0.045554
C	-2.657309	2.910762	0.100788
C	-0.349752	2.176320	0.048631
C	-1.241461	3.186852	0.107667
C	1.117519	2.309992	0.052918
C	1.715401	3.562621	-0.250912
C	1.886281	1.225309	0.364630
C	3.109070	3.706866	-0.207297
C	3.369627	1.277525	0.359407
C	3.904547	2.643861	0.110772
H	0.113394	-0.841655	-1.454154
H	0.138298	-3.875302	-1.334297
H	-1.721435	-2.840105	-0.068196
H	2.077024	-1.898218	-0.075472
H	0.008978	-3.739424	1.221168
H	1.808640	-1.568114	2.381533
H	2.058501	-3.314994	2.587094
H	-0.720027	-2.056038	-3.277530
H	2.080433	-3.519878	-2.502137
H	1.898897	-4.717322	0.025494
H	-5.752049	-0.522566	-0.007884
H	-0.250512	-1.930367	3.430861
H	-0.914011	4.212498	0.175436
H	-4.933440	2.920740	0.123330
H	1.105965	4.407228	-0.530488
H	1.439189	0.275285	0.615438
H	3.565374	4.663812	-0.426975
H	3.793944	0.847109	1.269792
H	5.572024	3.583359	-0.051164
O	3.796885	0.385572	-0.727611
O	5.090163	-0.128474	-0.440556
H	5.681839	0.583234	-0.726479

ENERGIES
SCF ENERGY: -1715.34600986
SUM OF ELECTRONIC AND ZERO-POINT ENERGIES: -1714.943797
SUM OF ELECTRONIC AND THERMAL ENERGIES: -1714.913384
SUM OF ELECTRONIC AND THERMAL ENTHALPIES: -1714.912440
SUM OF ELECTRONIC AND THERMAL FREE ENERGIES: -1715.006063

==> VITEXIN/WATER/PA/C7/RAF/C6 <==
53
XYZ-COORDINATES + ENERGIES

O	0.493177	-1.653674	1.083356
O	-0.652562	-2.011964	-2.350841
O	2.076220	-2.756554	-2.550654
O	3.221705	-3.813998	-0.108855
O	-0.029931	0.926526	0.019162
O	1.533249	-2.449837	3.565704
O	-3.223443	-2.525916	0.293119
O	-4.661401	1.955786	0.714872
O	-2.818212	3.798320	0.658192
O	5.905007	2.770805	-0.511350
C	0.297805	-1.789524	-1.313738
C	1.357330	-2.872769	-1.325470
C	-0.453849	-1.838003	0.018103
C	2.281298	-2.743275	-0.126955
C	1.455121	-2.706545	1.156473
C	-1.566207	-0.840064	0.134620
C	2.309521	-2.421029	2.369643
C	-1.322229	0.539921	0.158199
C	-2.890263	-1.308786	0.242280
C	-2.324324	1.514859	0.336959
C	-4.040125	-0.312472	0.237509
C	-3.645514	1.084740	0.468405
C	-1.950843	2.900203	0.442905
C	0.346476	2.219926	0.086009
C	-0.560819	3.205610	0.299906
C	1.792611	2.384994	-0.077929
C	2.340398	3.621425	-0.430345
C	2.650213	1.296212	0.117977
C	3.709237	3.770908	-0.576665
C	4.019055	1.438000	-0.026107
C	4.548010	2.677439	-0.374097
H	0.778434	-0.818055	-1.442810
H	0.859931	-3.848850	-1.280094
H	-0.892466	-2.833746	0.114089
H	2.865416	-1.825781	-0.214253
H	0.933412	-3.663986	1.280522
H	2.787304	-1.444972	2.248777
H	3.076711	-3.183818	2.470314
H	-0.165139	-2.104093	-3.176825
H	2.695543	-3.492306	-2.604869
H	2.733919	-4.644669	-0.053134
H	0.823526	-1.806795	3.465203
H	-0.242659	4.233057	0.387150
H	-4.241793	2.849587	0.766532
H	1.699035	4.472505	-0.611205
H	2.243364	0.335452	0.398165
H	4.132840	4.726641	-0.857551
H	4.684564	0.599864	0.130258
H	6.153082	3.670784	-0.753708
H	-4.811897	-0.642235	0.934308
O	-4.633069	-0.472007	-1.108075
O	-5.995770	-0.067446	-1.068129
H	-5.950313	0.890447	-1.198981

ENERGIES
SCF ENERGY: -1715.35187910
SUM OF ELECTRONIC AND ZERO-POINT ENERGIES: -1714.948812
SUM OF ELECTRONIC AND THERMAL ENERGIES: -1714.918498
SUM OF ELECTRONIC AND THERMAL ENTHALPIES: -1714.917554
SUM OF ELECTRONIC AND THERMAL FREE ENERGIES: -1715.011191

==> VITEXIN/WATER/PA/C7/RAF/C6' <==
53
XYZ-COORDINATES + ENERGIES

O	-0.218313	-1.680951	1.128053
O	-1.532041	-2.207779	-2.222563
O	1.127364	-3.179050	-2.461222
O	2.273088	-4.150264	0.020516
O	-0.484167	0.861568	0.057259
O	0.849364	-2.373706	3.625880
O	-4.038474	-2.236650	0.232577
O	-5.028803	2.367087	0.185479
O	-3.041191	4.017893	0.076779
O	5.629807	2.127724	0.495772
C	-0.520255	-1.983583	-1.244281
C	0.448283	-3.148032	-1.208226
C	-1.215875	-1.851720	0.110413
C	1.424233	-3.007437	-0.053523
C	0.654459	-2.804156	1.248622
C	-2.206940	-0.726054	0.156533
C	1.577480	-2.500761	2.406393
C	-1.826001	0.600776	0.110674
C	-3.611129	-1.032955	0.206185
C	-2.732084	1.678910	0.121745
C	-4.536814	0.058970	0.219579
C	-4.116500	1.358530	0.175755
C	-2.256979	3.026110	0.083145
C	-0.001903	2.128156	0.065095
C	-0.831463	3.203189	0.057240
C	1.446371	2.163573	0.099485
C	2.142681	3.323273	0.373928
C	2.184128	0.892537	-0.204466
C	3.529743	3.325694	0.468732
C	3.648318	0.946751	0.042918
C	4.267035	2.111128	0.326207
H	0.027271	-1.067441	-1.470378
H	-0.122181	-4.074653	-1.072243
H	-1.754690	-2.781783	0.304020

H 2.078987 -2.153070 -0.232229
H 0.061633 -3.702144 1.464388
H 2.127333 -1.579828 2.192335
H 2.283925 -3.315178 2.540886
H -1.094196 -2.349162 -3.068844
H 1.698409 -3.954545 -2.473755
H 1.719905 -4.927029 0.167499
H -5.594883 -0.162861 0.259576
H 0.195576 -1.678173 3.499224
H -0.437576 4.206945 0.034307
H -4.512351 3.209658 0.145148
H 1.615535 4.248786 0.556340
H 1.735972 0.053122 0.330504
H 4.060623 4.241956 0.688654
H 4.211764 0.029211 -0.059394
H 5.919474 3.014117 0.738725
O 1.921619 0.491730 -1.587485
O 2.452629 1.476773 -2.461577
H 1.691677 2.049675 -2.633410

ENERGIES
SCF ENERGY: -1715.34812999
SUM OF ELECTRONIC AND ZERO-POINT ENERGIES: -1714.946464
SUM OF ELECTRONIC AND THERMAL ENERGIES: -1714.915901
SUM OF ELECTRONIC AND THERMAL ENTHALPIES: -1714.914957
SUM OF ELECTRONIC AND THERMAL FREE ENERGIES: -1715.008271

==> VITEXIN/WATER/PA/C7/RAF/C7 <==
53
XYZ-COORDINATES + ENERGIES
O -0.173436 -1.604423 0.848431
O -0.828157 -1.482574 -2.727641
O 1.583319 -2.955977 -2.609193
O 2.011762 -4.396645 -0.125526
O 0.164769 1.058859 -0.119494
O 0.228260 -2.752276 3.384120
O -3.725766 -1.166694 -1.252687
O -4.115202 3.279766 -0.061436
O -1.868453 4.555068 0.146576
O 6.372838 1.383585 0.256000
C -0.035379 -1.587859 -1.552700
C 0.681878 -2.923349 -1.507175
C -0.965647 -1.494406 -0.339791
C 1.414802 -3.104527 -0.187464
C 0.449151 -2.885790 0.974133
C -1.771326 -0.231647 -0.262379
C 1.155723 -2.883940 2.309399
C -1.203838 1.012817 -0.139513
C -3.280485 -0.379451 -0.285816
C -1.947850 2.201520 -0.051859
C -4.011106 0.929925 -0.202250
C -3.389259 2.117340 -0.100381
C -1.248517 3.465602 0.070833
C 0.830908 2.217569 0.016148
C 0.179773 3.403407 0.116984
C 2.280363 2.021510 0.059026
C 3.158360 3.094983 -0.117328
C 2.805585 0.743554 0.285938
C 4.526720 2.901401 -0.057183
C 4.172184 0.543186 0.350422
C 5.032569 1.624853 0.181702
H 0.702860 -0.785421 -1.518885
H -0.060014 -3.723966 -1.610032
H -1.663775 -2.333505 -0.383421
H 2.233432 -2.385522 -0.125139
H -0.323998 -3.663838 0.957953
H 1.881399 -2.066348 2.328604
H 1.676256 -3.826880 2.452423
H -0.244710 -1.617684 -3.482545
H 1.983638 -3.831322 -2.643367
H 1.310982 -5.057997 -0.180186
H -5.090571 0.888385 -0.264949
H -0.264266 -1.936522 3.245443
H 0.728463 4.322252 0.250412
H -3.469013 4.017162 0.022511
H 2.780076 4.087478 -0.317529
H 2.139241 -0.094779 0.428296
H 5.208313 3.730023 -0.199282
H 4.582679 -0.440229 0.534385
H 6.865638 2.206613 0.153529
O -3.440833 -1.039386 1.095923
O -4.815364 -1.335693 1.314604
H -5.134162 -0.556962 1.789892

ENERGIES
SCF ENERGY: -1715.32902851
SUM OF ELECTRONIC AND ZERO-POINT ENERGIES: -1714.927645
SUM OF ELECTRONIC AND THERMAL ENERGIES: -1714.897231
SUM OF ELECTRONIC AND THERMAL ENTHALPIES: -1714.896287
SUM OF ELECTRONIC AND THERMAL FREE ENERGIES: -1714.989220

==> VITEXIN/WATER/PA/C7/RAF/C8 <==
53
XYZ-COORDINATES + ENERGIES
O 0.051802 -1.721609 0.707292
O -2.374064 -1.093055 -1.913506
O -0.260320 -2.356226 -3.312374
O 1.350177 -4.160312 -1.706197
O 0.130139 1.064416 0.415047
O 1.558027 -3.246621 2.483424
O -3.948201 -1.248065 1.148555
O -4.088424 3.178122 -0.511273
O -1.807472 4.381620 -0.886803
O 6.356995 1.174005 0.183052
C -1.086197 -1.313694 -1.353866
C -0.469676 -2.580389 -1.921847
C -1.232908 -1.466640 0.157763
C 0.829886 -2.929589 -1.214042
C 0.582298 -2.980015 0.289064
C -1.837518 -0.211820 0.829866
C 1.846575 -3.196014 1.087504
C -1.236461 1.060023 0.380609
C -3.360866 -0.188300 0.728580
C -1.949568 2.173161 -0.055599
C -4.032269 0.952060 0.300932
C -3.359463 2.105462 -0.094747
C -1.218471 3.345329 -0.488413
C 0.828309 2.126428 -0.016767
C 0.211870 3.251132 -0.461194
C 2.276168 1.903583 0.039934
C 3.170298 2.976403 0.063813
C 2.778403 0.597907 0.065770
C 4.535982 2.752915 0.108576
C 4.141624 0.366818 0.107814
C 5.019806 1.446922 0.131815
H -0.425535 -0.470755 -1.577122
H -1.177416 -3.405618 -1.781342
H -1.889566 -2.314234 0.364493
H 1.580640 -2.168206 -1.431986
H -0.142765 -3.770818 0.518911
H 2.554646 -2.391455 0.873313
H 2.296121 -4.148034 0.818615
H -2.272709 -1.047579 -2.870776
H 0.068928 -3.173685 -3.701141
H 0.713297 -4.859268 -1.513906
H -5.114081 0.938854 0.285116
H 1.078101 -2.441149 2.706601
H 0.791430 4.084230 -0.827624
H -3.441037 3.882406 -0.745609
H 2.805748 3.993964 0.066998
H 2.096552 -0.240304 0.041632
H 5.230264 3.582861 0.136949
H 4.535739 -0.640225 0.122741
H 6.861765 1.995507 0.207221
O -1.545619 -0.278150 2.286641
O -1.917051 -1.555074 2.823385
H -2.824978 -1.669007 2.455582

ENERGIES
SCF ENERGY: -1715.35095735
SUM OF ELECTRONIC AND ZERO-POINT ENERGIES: -1714.948512
SUM OF ELECTRONIC AND THERMAL ENERGIES: -1714.918655
SUM OF ELECTRONIC AND THERMAL ENTHALPIES: -1714.917711
SUM OF ELECTRONIC AND THERMAL FREE ENERGIES: -1715.009093

==> VITEXIN/WATER/PA/C7/RAF/C8a <==

53
XYZ-COORDINATES + ENERGIES

O	0.314531	-1.606133	1.112796
O	-1.168844	-2.379561	-2.114730
O	1.504201	-3.273354	-2.455792
O	2.867984	-4.005316	-0.003361
O	-0.238190	0.924812	0.124767
O	1.576754	-2.078296	3.575490
O	-3.378687	-2.497553	0.910153
O	-4.768215	1.988318	0.851027
O	-3.091410	3.808271	0.286481
O	5.677934	2.859489	-0.260731
C	-0.108398	-2.070965	-1.210667
C	0.907743	-3.193159	-1.165566
C	-0.727378	-1.911650	0.178374
C	1.947506	-2.922065	-0.089063
C	1.255392	-2.674253	1.248824
C	-1.797756	-0.866896	0.254903
C	2.230117	-2.240692	2.319020
C	-1.545215	0.527959	-0.231186
C	-3.140448	-1.274284	0.672857
C	-2.564156	1.517165	0.170365
C	-4.119570	-0.270662	0.817607
C	-3.820023	1.074856	0.603227
C	-2.234889	2.881145	0.088614
C	0.096170	2.236059	-0.066366
C	-0.841733	3.200705	-0.160908
C	1.550557	2.422242	-0.102834
C	2.125382	3.680527	0.101719
C	2.390382	1.327910	-0.342104
C	3.498159	3.847853	0.050661
C	3.764694	1.486423	-0.392401
C	4.317304	2.748289	-0.199472
H	0.389851	-1.148738	-1.511245
H	0.393057	-4.132200	-0.932313
H	-1.182009	-2.863756	0.456872
H	2.532178	-2.042554	-0.364975
H	0.727731	-3.583495	1.562290
H	2.704291	-1.304936	2.010482
H	2.993911	-3.001699	2.453167
H	-0.779090	-2.575684	-2.975108
H	2.101166	-4.029253	-2.464522
H	2.385169	-4.796191	0.266424
H	-5.110682	-0.545661	1.149931
H	0.881951	-1.422201	3.457435
H	-0.562568	4.230967	-0.319155
H	-4.341549	2.881500	0.666779
H	1.502881	4.537291	0.317935
H	1.965378	0.346881	-0.498845
H	3.942808	4.821429	0.212871
H	4.415431	0.644025	-0.583175
H	5.942944	3.774988	-0.113841
O	-1.397639	0.490370	-1.706949
O	-2.600573	0.016738	-2.279598
H	-2.388645	-0.932019	-2.381061

ENERGIES
SCF ENERGY: -1715.34397861
SUM OF ELECTRONIC AND ZERO-POINT ENERGIES: -1714.941176
SUM OF ELECTRONIC AND THERMAL ENERGIES: -1714.911635
SUM OF ELECTRONIC AND THERMAL ENTHALPIES: -1714.910691
SUM OF ELECTRONIC AND THERMAL FREE ENERGIES: -1715.001327

==> VITEXIN/WATER/RAF/C1' <==
54
XYZ-COORDINATES + ENERGIES

O	1.246552	1.320373	1.085382
O	2.892995	0.746949	-2.103847
O	1.492945	3.194336	-2.572105
O	0.974550	4.753306	-0.196277
O	-0.159442	-0.726400	-0.165419
O	0.732690	2.635805	3.503563
O	4.452238	-0.822501	0.689631
O	2.055644	-4.902740	0.550599
O	-0.502543	-4.754721	0.071765
O	-6.181083	0.646254	1.175454
C	1.876183	1.264724	-1.253397
C	1.879326	2.780086	-1.265635
C	2.183740	0.764038	0.159840
C	0.937443	3.329043	-0.206829
C	1.311705	2.746328	1.152330
C	2.138588	-0.735485	0.266074
C	0.349406	3.169266	2.238123
C	0.958422	-1.453105	0.098093
C	3.284760	-1.490287	0.527486
C	0.878818	-2.846177	0.185979
C	3.260991	-2.883619	0.619986
C	2.070918	-3.558072	0.453642
C	-0.392253	-3.512311	0.001496
C	-1.348734	-1.324809	-0.339619
C	-1.520184	-2.655831	-0.268989
C	-2.442869	-0.298071	-0.544563
C	-3.717101	-0.898499	-1.037195
C	-2.600714	0.512960	0.702647
C	-4.917166	-0.567062	-0.498953
C	-3.820560	0.800120	1.218180
C	-4.994527	0.293921	0.618868
H	0.895775	0.910491	-1.574725
H	2.894395	3.133537	-1.048314
H	3.185710	1.107812	0.423539
H	-0.089418	3.049891	-0.449884
H	2.333004	3.049030	1.415189
H	-0.658670	2.840134	1.973878
H	0.356979	4.251388	2.334458
H	2.720092	1.060466	-2.998082
H	1.529858	4.156427	-2.604087
H	1.868124	5.033979	0.035305
H	4.170661	-3.432919	0.823717
H	0.750532	1.676609	3.421423
H	-2.497011	-3.094074	-0.405897
H	5.166673	-1.445427	0.871050
H	1.125734	-5.193770	0.403003
H	-3.658628	-1.570181	-1.882162
H	-1.695005	0.886826	1.159076
H	-5.834433	-0.970107	-0.910991
H	-3.914166	1.412383	2.105389
H	-6.913422	0.261432	0.678542
O	-1.935726	0.684774	-1.521435
O	-1.528655	0.000506	-2.701308
H	-2.315250	0.045618	-3.263821

ENERGIES
SCF ENERGY: -1715.80041010
SUM OF ELECTRONIC AND ZERO-POINT ENERGIES: -1715.385544
SUM OF ELECTRONIC AND THERMAL ENERGIES: -1715.354955
SUM OF ELECTRONIC AND THERMAL ENTHALPIES: -1715.354011
SUM OF ELECTRONIC AND THERMAL FREE ENERGIES: -1715.447535

==> VITEXIN/WATER/RAF/C2 <==
54
XYZ-COORDINATES + ENERGIES

O	0.248011	-1.828980	1.067628
O	-1.137545	-2.373836	-2.249005
O	1.651547	-2.807955	-2.678374
O	3.108889	-3.659013	-0.334932
O	-0.395692	0.686018	0.109002
O	1.518952	-2.521930	3.459420
O	-3.408411	-2.900807	0.445261
O	-5.086472	1.511642	0.693325
O	-3.340039	3.456699	0.542380
O	5.336784	2.795116	0.677735
C	-0.120359	-2.018151	-1.318795
C	1.046945	-2.980901	-1.400748
C	-0.746459	-2.091864	0.075085
C	2.037938	-2.721975	-0.278233
C	1.310518	-2.784276	1.060695
C	-1.882015	-1.119345	0.247692
C	2.212343	-2.422147	2.217637
C	-1.688018	0.251285	0.215386
C	-3.204031	-1.563927	0.423723
C	-2.735703	1.175036	0.355897
C	-4.274087	-0.686929	0.566531
C	-4.042146	0.675105	0.539614
C	-2.459638	2.595742	0.324211
C	-0.133998	1.960811	-0.436171
C	-1.118689	2.987279	0.001470

C	1.310561	2.264379	-0.148366
C	1.701651	3.282803	0.711632
C	2.277554	1.429390	-0.711927
C	3.046826	3.478471	1.001805
C	3.618376	1.615956	-0.429755
C	3.999613	2.645649	0.428773
H	0.237572	-1.006900	-1.517078
H	0.668103	-4.005252	-1.303844
H	-1.123137	-3.106476	0.216093
H	2.485137	-1.734343	-0.404501
H	0.894764	-3.789074	1.206919
H	2.590804	-1.406909	2.072132
H	3.049808	-3.112630	2.263693
H	-0.752349	-2.346797	-3.131579
H	2.371338	-3.443566	-2.756277
H	2.748064	-4.541995	-0.189522
H	-5.279237	-1.062488	0.701855
H	0.757430	-1.934353	3.412545
H	-0.883821	4.038020	-0.067645
H	-4.344352	-3.096604	0.579894
H	-4.737453	2.429782	0.687586
H	0.971267	3.932714	1.174002
H	1.976678	0.629387	-1.375495
H	3.358317	4.271250	1.669762
H	4.375676	0.977175	-0.863059
H	5.478913	3.528919	1.287106
O	-0.340403	1.711491	-1.857607
O	-0.018015	2.892276	-2.569012
H	-0.887460	3.260035	-2.786088

ENERGIES
SCF ENERGY: -1715.81222169
SUM OF ELECTRONIC AND ZERO-POINT ENERGIES: -1715.395868
SUM OF ELECTRONIC AND THERMAL ENERGIES: -1715.365574
SUM OF ELECTRONIC AND THERMAL ENTHALPIES: -1715.364630
SUM OF ELECTRONIC AND THERMAL FREE ENERGIES: -1715.457016

==> VITEXIN/WATER/RAF/C2' <==
54
XYZ-COORDINATES + ENERGIES

O	1.643136	-1.234938	-0.962302
O	2.582914	-0.497087	2.466454
O	1.486088	-3.110924	2.696977
O	1.662970	-4.700187	0.272955
O	-0.116244	0.731620	-0.087871
O	1.641037	-2.581239	-3.427234
O	4.553967	1.134585	0.070766
O	1.920886	5.048081	-0.367675
O	-0.653925	4.734644	-0.436427
O	-5.270270	-2.820220	-0.344609
C	1.835128	-1.133734	1.437549
C	2.039781	-2.635044	1.474345
C	2.351351	-0.597015	0.102819
C	1.388510	-3.302188	0.273947
C	1.877007	-2.644351	-1.014711
C	2.215466	0.895124	-0.027813
C	1.128542	-3.151846	-2.225363
C	0.980298	1.530670	-0.118367
C	3.339321	1.726420	-0.041110
C	0.835733	2.916290	-0.236609
C	3.247313	3.114811	-0.157153
C	2.006614	3.707192	-0.254823
C	-0.486411	3.497029	-0.328421
C	-1.368619	1.241088	-0.176414
C	-1.585892	2.578874	-0.289683
C	-2.389404	0.221128	-0.156536
C	-3.825624	0.648269	-0.138993
C	-2.054537	-1.118497	-0.148253
C	-4.803688	-0.468799	-0.263721
C	-3.026966	-2.110881	-0.185108
C	-4.407275	-1.759093	-0.260751
H	0.771336	-0.910682	1.544473
H	3.116376	-2.840928	1.448596
H	3.408538	-0.857333	0.026078
H	0.304575	-3.205768	0.349033
H	2.952595	-2.826593	-1.131198
H	0.066807	-2.915800	-2.112889
H	1.248511	-4.228445	-2.308544
H	2.310192	-0.878890	3.307904
H	1.692856	-4.048331	2.775839
H	2.617825	-4.823225	0.207862
H	4.141724	3.723076	-0.166021
H	1.560639	-1.624594	-3.353265
H	-2.587796	2.973171	-0.351613
H	5.253453	1.799463	0.059635
H	0.960270	5.269597	-0.423266
H	-1.014278	-1.406959	-0.137689
H	-5.853035	-0.212473	-0.333962
H	-2.752006	-3.155420	-0.177488
H	-6.179997	-2.502752	-0.394980
H	-4.017182	1.402839	-0.905000
O	-3.993094	1.328692	1.145288
O	-5.200325	2.073004	1.093690
H	-5.838420	1.489122	1.527416

ENERGIES
SCF ENERGY: -1715.80846568
SUM OF ELECTRONIC AND ZERO-POINT ENERGIES: -1715.393227
SUM OF ELECTRONIC AND THERMAL ENERGIES: -1715.362537
SUM OF ELECTRONIC AND THERMAL ENTHALPIES: -1715.361593
SUM OF ELECTRONIC AND THERMAL FREE ENERGIES: -1715.454868

==> VITEXIN/WATER/RAF/C3 <==
54
XYZ-COORDINATES + ENERGIES

O	1.341796	-1.509644	0.910632
O	0.206400	-2.591349	-2.368406
O	2.842639	-1.669120	-2.920850
O	4.653066	-1.765290	-0.668917
O	-0.565201	0.350975	-0.011594
O	2.923781	-1.514310	3.226122
O	-1.303090	-4.244980	0.504578
O	-4.937898	-1.286236	1.075511
O	-4.440008	1.276830	0.710581
O	3.266084	5.290475	0.477105
C	0.984735	-1.821158	-1.459827
C	2.460996	-2.120278	-1.625299
C	0.548710	-2.215340	-0.048049
C	3.273265	-1.437606	-0.537505
C	2.730819	-1.839450	0.831518
C	-0.906094	-1.943524	0.222253
C	3.411509	-1.091454	1.954515
C	-1.435051	-0.659263	0.222333
C	-1.798138	-2.986996	0.508177
C	-2.789544	-0.390436	0.486189
C	-3.144451	-2.770205	0.797350
C	-3.636044	-1.482152	0.796049
C	-3.293823	0.950681	0.388047
C	-0.954583	1.660779	0.014917
C	-2.370382	1.976113	-0.238082
C	0.101947	2.601500	0.125901
C	-0.129179	3.990134	0.016751
C	1.428626	2.163501	0.349723
C	0.910215	4.891039	0.132993
C	2.464676	3.066210	0.463579
C	2.208828	4.433387	0.356764
H	0.817543	-0.755431	-1.624135
H	2.612045	-3.203664	-1.549281
H	0.731636	-3.285182	0.066459
H	3.202163	-0.354838	-0.654588
H	2.852884	-2.920831	0.971272
H	3.250251	-0.018363	1.821313
H	4.478258	-1.296770	1.939303
H	0.517593	-2.396980	-3.259350
H	3.763675	-1.912226	-3.064432
H	4.747398	-2.722918	-0.598445
H	-3.798137	-3.601786	1.022685
H	1.974163	-1.355520	3.241080
H	-1.997947	-4.876800	0.727091
H	-5.114844	-0.323231	1.048885
H	-1.124097	4.368813	-0.167047
H	1.633167	1.106964	0.440025
H	0.727179	5.954761	0.047229
H	3.477556	2.728006	0.636899
H	2.963269	6.201788	0.385983
H	-2.640666	2.969447	0.108907

O -2.555029 1.928084 -1.683135
O -3.817583 2.498823 -2.000475
H -4.394976 1.727800 -2.098856

ENERGIES
SCF ENERGY: -1715.81565938
SUM OF ELECTRONIC AND ZERO-POINT ENERGIES: -1715.399717
SUM OF ELECTRONIC AND THERMAL ENERGIES: -1715.368856
SUM OF ELECTRONIC AND THERMAL ENTHALPIES: -1715.367912
SUM OF ELECTRONIC AND THERMAL FREE ENERGIES: -1715.462101

==> VITEXIN/WATER/RAF/C3/TS <==
54
XYZ-COORDINATES + ENERGIES
O 1.791789 -1.099365 0.937638
O 1.040667 -2.004463 -2.495477
O 3.305713 -0.301900 -2.792832
O 5.085908 -0.296650 -0.486714
O -0.649801 0.110597 0.188370
O 3.244711 -0.860891 3.326226
O 0.218245 -4.489588 -0.042568
O -4.241832 -3.017345 0.589442
O -4.624789 -0.470942 0.706847
O 1.341744 6.014067 0.158560
C 1.570122 -1.198565 -1.449829
C 3.070179 -1.034535 -1.595119
C 1.283242 -1.906420 -0.125574
C 3.664624 -0.321392 -0.389676
C 3.218097 -0.996557 0.906044
C -0.179973 -2.178097 0.088965
C 3.614602 -0.192151 2.122795
C -1.121052 -1.164692 0.229111
C -0.676770 -3.486433 0.109375
C -2.490025 -1.401020 0.400613
C -2.030958 -3.772241 0.286366
C -2.932568 -2.738728 0.433319
C -3.406584 -0.302247 0.537133
C -1.457540 1.184260 0.293166
C -2.842463 1.040868 0.391806
C -0.752788 2.440378 0.250315
C -1.447721 3.651130 0.105926
C 0.649512 2.466990 0.345792
C -0.764928 4.851484 0.074080
C 1.334428 3.663590 0.316162
C 0.626137 4.858021 0.181861
H 1.101822 -0.211632 -1.452023
H 3.524476 -2.029340 -1.672450
H 1.820295 -2.858091 -0.121545
H 3.343915 0.721923 -0.390340
H 3.655994 -2.001019 0.962957
H 3.143946 0.793239 2.073890
H 4.694110 -0.070300 2.145299
H 1.267613 -1.587428 -3.333840
H 4.256587 -0.266260 -2.942266
H 5.406051 -1.206599 -0.486270
H -2.375111 -4.797538 0.301423
H 2.291339 -0.993589 3.302077
H -0.229727 -5.343232 0.002705
H -4.728089 -2.170973 0.677267
H -2.523725 3.657929 0.004947
H 1.196006 1.541459 0.454930
H -1.296561 5.787163 -0.041397
H 2.412308 3.691640 0.398923
H 0.749507 6.770978 0.073699
H -3.416065 1.849664 0.819275
O -3.462683 1.353330 -1.380105
O -4.616635 2.121367 -1.295324
H -5.324418 1.474389 -1.165537

ENERGIES
SCF ENERGY: -1715.79694071
SUM OF ELECTRONIC AND ZERO-POINT ENERGIES: -1715.382674
SUM OF ELECTRONIC AND THERMAL ENERGIES: -1715.351963
SUM OF ELECTRONIC AND THERMAL ENTHALPIES: -1715.351019
SUM OF ELECTRONIC AND THERMAL FREE ENERGIES: -1715.445427

==> VITEXIN/WATER/RAF/C3' <==
54
XYZ-COORDINATES + ENERGIES
O -1.242224 -1.666048 0.932189
O -1.965808 -1.355909 -2.614863
O 0.243537 -3.162057 -2.604017
O 0.526330 -4.695739 -0.169144
O -0.364902 0.871616 0.132261
O -1.029471 -2.972124 3.408260
O -4.735340 -0.731745 -0.446171
O -4.046321 3.919259 0.122092
O -1.591214 4.718007 0.437068
O 5.732734 -0.253239 0.804366
C -1.137492 -1.597918 -1.483784
C -0.624604 -3.024119 -1.483452
C -2.002320 -1.370247 -0.242651
C 0.094758 -3.338435 -0.182402
C -0.827313 -3.033155 0.994379
C -2.530650 0.034357 -0.142454
C -0.132826 -3.208275 2.325000
C -1.695273 1.131779 0.047053
C -3.895256 0.313359 -0.246412
C -2.155040 2.447462 0.143009
C -4.406708 1.610208 -0.158714
C -3.547219 2.669743 0.036367
C -1.213225 3.528018 0.345984
C 0.535189 1.857702 0.321974
C 0.169159 3.151885 0.445405
C 1.908330 1.331470 0.380070
C 2.965630 2.133242 0.062044
C 2.103949 -0.017442 0.780401
C 4.361498 1.623995 0.065503
C 3.398907 -0.533132 0.909364
C 4.484636 0.240830 0.612991
H -0.285597 -0.915615 -1.482437
H -1.477084 -3.706037 -1.586509
H -2.846796 -2.059768 -0.297538
H 0.996827 -2.728902 -0.107517
H -1.711496 -3.680851 0.945003
H 0.720266 -2.526680 2.378704
H 0.221204 -4.230720 2.423113
H -1.443625 -1.525718 -3.406292
H 0.529611 -4.080615 -2.650125
H -0.252660 -5.262104 -0.224900
H -5.471698 1.781757 -0.245074
H -1.371381 -2.077213 3.311731
H 0.908567 3.916170 0.627963
H -5.645604 -0.417555 -0.508729
H -3.280263 4.525840 0.257914
H 2.828343 3.154553 -0.261879
H 1.252952 -0.634013 1.021947
H 3.553173 -1.542794 1.266045
H 6.392998 0.364870 0.462084
H 5.041151 2.307528 0.582792
O 4.769557 1.657155 -1.343059
O 6.190974 1.538658 -1.390287
H 6.313514 0.687233 -1.833888

ENERGIES
SCF ENERGY: -1715.80423298
SUM OF ELECTRONIC AND ZERO-POINT ENERGIES: -1715.388759
SUM OF ELECTRONIC AND THERMAL ENERGIES: -1715.358109
SUM OF ELECTRONIC AND THERMAL ENTHALPIES: -1715.357164
SUM OF ELECTRONIC AND THERMAL FREE ENERGIES: -1715.450519

==> VITEXIN/WATER/RAF/C4 <==
54
XYZ-COORDINATES + ENERGIES
O 1.255262 -1.397069 1.020328
O 0.572446 -2.604932 -2.336688
O 3.271118 -1.723029 -2.552872
O 4.751669 -1.767542 -0.061327
O -0.525694 0.399763 -0.184161
O 2.490279 -1.286019 3.529809
O -1.280223 -4.221319 0.232133
O -5.017008 -1.329782 0.171718
O -3.540909 1.394338 -1.453617
O 3.535570 5.107323 -0.118467
C 1.226729 -1.807915 -1.356845
C 2.708709 -2.123693 -1.307515

C	0.601345	-2.150422	-0.004088
C	3.375357	-1.409797	-0.143006
C	2.637974	-1.737829	1.153096
C	-0.881251	-1.898435	0.054034
C	3.164305	-0.937948	2.322649
C	-1.435577	-0.621933	-0.019786
C	-1.784921	-2.957388	0.171899
C	-2.798536	-0.361352	0.038487
C	-3.156758	-2.746595	0.222400
C	-3.661256	-1.459140	0.151708
C	-3.268197	1.051112	-0.172386
C	-0.882295	1.692947	-0.125678
C	-2.198248	2.062509	-0.024578
C	0.245355	2.600571	-0.126840
C	0.077980	3.965921	-0.403961
C	1.533026	2.111228	0.148096
C	1.164158	4.817223	-0.403922
C	2.620783	2.957487	0.152638
C	2.436294	4.312816	-0.125655
H	1.093033	-0.747007	-1.575978
H	2.832756	-3.204831	-1.173993
H	0.776180	-3.211991	0.179639
H	3.351236	-0.333011	-0.316395
H	2.725124	-2.811469	1.361599
H	3.044641	0.128193	2.112302
H	4.218143	-1.155745	2.472281
H	0.989454	-2.428507	-3.186997
H	4.197871	-1.985564	-2.561152
H	4.811007	-2.716956	0.099215
H	-3.837632	-3.582917	0.309177
H	1.552000	-1.116072	3.395916
H	-2.489813	3.094943	0.071114
H	-2.000591	-4.858048	0.311066
H	-5.255234	-0.396933	0.287092
H	-0.898329	4.362652	-0.643392
H	1.668745	1.064182	0.373498
H	1.044212	5.869401	-0.626966
H	3.614588	2.590002	0.368848
H	3.293481	6.018357	-0.324430
O	-4.375183	1.215221	0.737289
O	-4.947374	2.499102	0.580309
H	-4.600367	2.976686	1.347442

ENERGIES
SCF ENERGY: -1715.77530153
SUM OF ELECTRONIC AND ZERO-POINT ENERGIES: -1715.359236
SUM OF ELECTRONIC AND THERMAL ENERGIES: -1715.328706
SUM OF ELECTRONIC AND THERMAL ENTHALPIES: -1715.327762
SUM OF ELECTRONIC AND THERMAL FREE ENERGIES: -1715.420525

==> VITEXIN/WATER/RAF/C4a <==
54
XYZ-COORDINATES + ENERGIES

O	0.837041	-1.534397	1.024833
O	-0.357131	-2.635423	-2.220159
O	2.469983	-2.669889	-2.574222
O	3.967187	-3.094125	-0.119046
O	-0.377810	0.687034	-0.155812
O	2.181521	-1.666696	3.485254
O	-2.472552	-3.491836	0.441447
O	-5.131422	0.427465	0.648685
O	-3.729731	2.582242	1.091291
O	4.844489	4.065570	-0.411583
C	0.563210	-2.043915	-1.312045
C	1.870815	-2.810843	-1.290561
C	-0.063569	-2.110302	0.079715
C	2.788363	-2.297558	-0.191741
C	2.049218	-2.285193	1.144875
C	-1.395259	-1.407508	0.154439
C	2.858590	-1.616478	2.231694
C	-1.515617	-0.045653	0.044667
C	-2.576944	-2.146151	0.357433
C	-2.801191	0.675268	-0.034491
C	-3.826609	-1.518471	0.528206
C	-3.943306	-0.169350	0.423925
C	-2.709921	2.045004	0.630928
C	-0.332431	1.993789	0.155769
C	-1.430537	2.659724	0.611801
C	1.003343	2.558699	-0.010988
C	1.207668	3.942806	-0.029153
C	2.107749	1.707508	-0.144839
C	2.481845	4.462492	-0.163480
C	3.384326	2.219543	-0.277116
C	3.570605	3.599902	-0.282825
H	0.755468	-1.004018	-1.584836
H	1.650869	-3.867296	-1.096149
H	-0.212524	-3.161827	0.330959
H	3.120068	-1.288127	-0.438249
H	1.804981	-3.314450	1.435258
H	3.055637	-0.579924	1.945604
H	3.803427	-2.137589	2.358877
H	0.045248	-2.628895	-3.095527
H	3.254590	-3.227758	-2.602852
H	3.709261	-3.999878	0.091101
H	-4.694608	-2.112370	0.784219
H	1.344523	-1.202076	3.382278
H	-1.350213	3.678675	0.955762
H	-3.338465	-3.886337	0.604532
H	-4.958925	1.384715	0.783542
H	0.372883	4.625094	0.044485
H	1.961562	0.638154	-0.131972
H	2.643809	5.532472	-0.183618
H	4.240321	1.565474	-0.371696
H	4.850101	5.030175	-0.387434
O	-2.937391	0.927247	-1.517929
O	-4.061128	1.758152	-1.778103
H	-3.664549	2.633152	-1.898810

ENERGIES
SCF ENERGY: -1715.78612406
SUM OF ELECTRONIC AND ZERO-POINT ENERGIES: -1715.371300
SUM OF ELECTRONIC AND THERMAL ENERGIES: -1715.340530
SUM OF ELECTRONIC AND THERMAL ENTHALPIES: -1715.339586
SUM OF ELECTRONIC AND THERMAL FREE ENERGIES: -1715.433616

==> VITEXIN/WATER/RAF/C4' <==
54
XYZ-COORDINATES + ENERGIES

O	0.945817	1.599428	1.033162
O	2.351720	1.621488	-2.315493
O	0.149136	3.399738	-2.579829
O	-0.627144	4.721242	-0.115724
O	0.431293	-0.919083	-0.051588
O	0.137747	2.615184	3.517502
O	4.702083	0.999496	0.246872
O	4.300342	-3.717135	0.214576
O	1.898970	-4.695537	0.055616
O	-5.318675	-0.333593	-1.436332
C	1.324684	1.756423	-1.340835
C	0.781537	3.171204	-1.324823
C	1.945364	1.449449	0.022621
C	-0.188400	3.367452	-0.171354
C	0.471664	2.944654	1.139229
C	2.543831	0.071783	0.099156
C	-0.504510	2.960802	2.292545
C	1.774645	-1.087042	0.044969
C	3.926573	-0.110519	0.196562
C	2.319488	-2.374471	0.085640
C	4.519013	-1.374126	0.235312
C	3.724385	-2.498638	0.179678
C	1.447945	-3.527127	0.027820
C	-0.418135	-1.975311	-0.096886
C	0.042341	-3.256186	-0.059151
C	-1.798979	-1.572206	-0.176921
C	-2.841359	-2.536116	-0.349221
C	-2.140332	-0.186400	-0.098137
C	-4.133253	-2.161484	-0.423433
C	-3.421426	0.226465	-0.165947
C	-4.556910	-0.730642	-0.320693
H	0.509347	1.059440	-1.546347
H	1.620248	3.866185	-1.199512
H	2.735966	2.180467	0.201077
H	-1.082205	2.766167	-0.344064
H	1.319273	3.608018	1.351523
H	-1.320844	2.265116	2.079971
H	-0.910736	3.960782	2.416782

H	1.984770	1.884879	-3.166568
H	-0.127713	4.321735	-2.612584
H	0.141993	5.284655	0.031982
H	5.593828	-1.471823	0.306415
H	0.514516	1.735846	3.408375
H	-0.636459	-4.092999	-0.088320
H	5.630084	0.750763	0.335580
H	3.573185	-4.382122	0.158417
H	-2.596457	-3.584232	-0.436556
H	-1.351777	0.541057	0.017490
H	-4.918469	-2.890354	-0.570765
H	-3.684287	1.274395	-0.113424
H	-5.929955	-1.042725	-1.673810
O	-5.332859	-0.524215	0.882057
O	-6.490942	-1.345448	0.827647
H	-7.153212	-0.755547	0.439819

ENERGIES
SCF ENERGY: -1715.81253474
SUM OF ELECTRONIC AND ZERO-POINT ENERGIES: -1715.396867
SUM OF ELECTRONIC AND THERMAL ENERGIES: -1715.366202
SUM OF ELECTRONIC AND THERMAL ENTHALPIES: -1715.365258
SUM OF ELECTRONIC AND THERMAL FREE ENERGIES: -1715.458671

==> VITEXIN/WATER/RAF/C5 <==
54
XYZ-COORDINATES + ENERGIES

O	0.734946	-1.607397	1.025392
O	-0.553653	-2.268080	-2.304328
O	2.251932	-2.597001	-2.669876
O	3.687390	-3.374562	-0.273217
O	-0.157488	0.811544	0.046433
O	2.025822	-2.128102	3.456607
O	-2.750626	-3.087416	0.459768
O	-4.693522	1.074634	1.227176
O	-3.318044	3.293890	0.428375
O	5.512575	3.356220	-0.323282
C	0.423510	-1.865990	-1.354255
C	1.634684	-2.776445	-1.400452
C	-0.217520	-1.982418	0.031103
C	2.595849	-2.460469	-0.265425
C	1.853786	-2.499398	1.068358
C	-1.450313	-1.138609	0.174002
C	2.721609	-2.037184	2.215820
C	-1.396357	0.290808	0.145939
C	-2.718764	-1.727022	0.361782
C	-2.491777	1.097199	0.234359
C	-3.861601	-0.984369	0.459792
C	-3.861997	0.489998	0.264817
C	-2.337219	2.532200	0.280277
C	0.046897	2.149536	0.050849
C	-0.988199	3.013221	0.174091
C	1.466607	2.486750	-0.062766
C	1.877142	3.786815	-0.370518
C	2.435584	1.498016	0.142285
C	3.222820	4.095411	-0.461951
C	3.782302	1.799357	0.053800
C	4.175018	3.100699	-0.246816
H	0.738076	-0.836974	-1.538988
H	1.295475	-3.813596	-1.294159
H	-0.494912	-3.026494	0.181942
H	3.025955	-1.469632	-0.418511
H	1.492474	-3.517842	1.255946
H	3.042834	-1.008448	2.032617
H	3.596920	-2.676187	2.293072
H	-0.138335	-2.266235	-3.173964
H	2.982302	-3.221439	-2.738811
H	3.339352	-4.263259	-0.131193
H	-4.816604	-1.462255	0.628178
H	1.239638	-1.576085	3.389657
H	-0.814145	4.077173	0.215411
H	-3.662232	-3.385294	0.564159
H	-4.554378	2.037261	1.152348
H	1.149891	4.565072	-0.553778
H	2.132350	0.490333	0.385601
H	3.542816	5.100417	-0.704712
H	4.534383	1.039533	0.217693
H	5.660682	4.288228	-0.522653
O	-4.368754	0.810691	-1.061830
O	-5.687851	0.305677	-1.190287
H	-6.226719	0.996310	-0.777861

ENERGIES
SCF ENERGY: -1715.80776961
SUM OF ELECTRONIC AND ZERO-POINT ENERGIES: -1715.391402
SUM OF ELECTRONIC AND THERMAL ENERGIES: -1715.360896
SUM OF ELECTRONIC AND THERMAL ENTHALPIES: -1715.359952
SUM OF ELECTRONIC AND THERMAL FREE ENERGIES: -1715.452710

==> VITEXIN/WATER/RAF/C5' <==
54
XYZ-COORDINATES + ENERGIES

O	-0.376554	-1.663698	1.046805
O	-1.362858	-2.042129	-2.428639
O	1.356921	-2.868658	-2.487374
O	2.344194	-3.921964	0.028723
O	-0.704102	0.905935	-0.009954
O	0.538791	-2.436833	3.593185
O	-4.013009	-2.417926	-0.056454
O	-5.322901	2.130902	0.087664
O	-3.419191	3.904984	0.112509
O	5.318994	2.606459	0.138375
C	-0.450753	-1.834407	-1.356991
C	0.575578	-2.947925	-1.299293
C	-1.262756	-1.840871	-0.061348
C	1.441177	-2.823398	-0.056712
C	0.556531	-2.738170	1.185394
C	-2.325585	-0.777789	-0.019978
C	1.362383	-2.443295	2.429320
C	-2.022070	0.580277	0.007544
C	-3.684252	-1.103433	-0.017268
C	-2.988975	1.588036	0.046171
C	-4.689650	-0.134535	0.018501
C	-4.348095	1.200140	0.051602
C	-2.581341	2.975619	0.079377
C	-0.292524	2.189406	0.031419
C	-1.165331	3.219702	0.081842
C	1.175306	2.294563	0.028572
C	1.794438	3.534187	-0.280889
C	1.923743	1.194826	0.341437
C	3.190607	3.651705	-0.242227
C	3.407810	1.219538	0.331906
C	3.966942	2.575258	0.077237
H	0.064133	-0.878663	-1.473982
H	0.048528	-3.908770	-1.264743
H	-1.742947	-2.817331	0.025769
H	2.056466	-1.925603	-0.131472
H	0.005420	-3.679049	1.306257
H	1.862528	-1.478286	2.310222
H	2.110854	-3.217312	2.574521
H	-0.850417	-2.107174	-3.242003
H	1.957561	-3.621555	-2.502939
H	1.827898	-4.735725	0.078167
H	-5.730845	-0.428319	0.019613
H	-0.133461	-1.758762	3.470472
H	-0.816883	4.238849	0.138028
H	-4.972325	-2.523361	-0.048693
H	-4.879514	3.011731	0.103386
H	1.200955	4.389825	-0.561085
H	1.460092	0.253994	0.596553
H	3.664216	4.599166	-0.466218
H	3.825592	0.784559	1.243221
H	5.648503	3.485733	-0.090836
O	3.814426	0.316896	-0.752972
O	5.099326	-0.219146	-0.469053
H	5.702247	0.480882	-0.760461

ENERGIES
SCF ENERGY: -1715.80620309
SUM OF ELECTRONIC AND ZERO-POINT ENERGIES: -1715.390931
SUM OF ELECTRONIC AND THERMAL ENERGIES: -1715.360005
SUM OF ELECTRONIC AND THERMAL ENTHALPIES: -1715.359061
SUM OF ELECTRONIC AND THERMAL FREE ENERGIES: -1715.454086

==> VITEXIN/WATER/RAF/C6 <==
54

XYZ-COORDINATES + ENERGIES

O	0.459063	-1.619058	1.083060
O	-0.779635	-2.068544	-2.300887
O	1.932597	-2.888118	-2.542989
O	3.102665	-3.894436	-0.086984
O	0.039164	0.938446	0.013127
O	1.548059	-2.352829	3.571690
O	-3.243627	-2.438575	0.249540
O	-4.552552	2.125420	0.649631
O	-2.655024	3.910384	0.572931
O	6.023148	2.584611	-0.518780
C	0.207293	-1.837297	-1.303851
C	1.240381	-2.946116	-1.300178
C	-0.503869	-1.827954	0.049703
C	2.193821	-2.798252	-0.126011
C	1.401669	-2.690958	1.175404
C	-1.576780	-0.777080	0.146833
C	2.291233	-2.375725	2.355239
C	-1.265328	0.609323	0.158406
C	-2.890234	-1.132101	0.229561
C	-2.236197	1.605606	0.314251
C	-4.018558	-0.159318	0.260391
C	-3.568919	1.236325	0.436612
C	-1.817702	2.996606	0.387769
C	0.453633	2.219984	0.054068
C	-0.418009	3.243884	0.244463
C	1.903560	2.335726	-0.107949
C	2.492628	3.551265	-0.467429
C	2.721883	1.218970	0.099982
C	3.865416	3.652694	-0.608146
C	4.094807	1.313801	-0.036340
C	4.665787	2.533333	-0.390413
H	0.706200	-0.881185	-1.474234
H	0.720262	-3.907065	-1.209248
H	-0.964725	-2.807394	0.190809
H	2.800995	-1.901950	-0.261583
H	0.861078	-3.629286	1.351171
H	2.780072	-1.412197	2.188177
H	3.049211	-3.147061	2.460163
H	-0.325538	-2.169792	-3.144681
H	2.528049	-3.643898	-2.590081
H	2.593251	-4.709288	0.002501
H	0.865208	-1.679984	3.482872
H	-0.063565	4.260363	0.311493
H	-4.204814	-2.530408	0.225918
H	-4.125865	3.016119	0.683853
H	1.883309	4.423075	-0.658997
H	2.281336	0.274575	0.384687
H	4.323158	4.591075	-0.893843
H	4.731355	0.455670	0.130839
H	6.298783	3.476570	-0.762412
H	-4.757174	-0.442986	1.014972
O	-4.702762	-0.324539	-1.034585
O	-6.080396	-0.022068	-0.878742
H	-6.125258	0.923194	-1.082666

ENERGIES
SCF ENERGY: -1715.80542745
SUM OF ELECTRONIC AND ZERO-POINT ENERGIES: -1715.389632
SUM OF ELECTRONIC AND THERMAL ENERGIES: -1715.358672
SUM OF ELECTRONIC AND THERMAL ENTHALPIES: -1715.357727
SUM OF ELECTRONIC AND THERMAL FREE ENERGIES: -1715.452828

==> VITEXIN/WATER/RAF/C6' <==
54
XYZ-COORDINATES + ENERGIES

O	-0.233325	-1.646621	1.129574
O	-1.626385	-2.200703	-2.178820
O	1.017207	-3.222085	-2.461011
O	2.192466	-4.177454	0.014221
O	-0.443847	0.886318	0.062512
O	0.868611	-2.315682	3.616654
O	-4.019693	-2.151488	0.275726
O	-4.945328	2.491423	0.167966
O	-2.918330	4.096354	0.050915
O	5.702204	1.974303	0.467007
C	-0.585245	-1.981255	-1.233478
C	0.364351	-3.161632	-1.196787
C	-1.244652	-1.820402	0.135881
C	1.364817	-3.020315	-0.062187
C	0.624665	-2.783735	1.251810
C	-2.201479	-0.659922	0.175528
C	1.572797	-2.476043	2.387555
C	-1.783828	0.667783	0.116854
C	-3.582417	-0.868842	0.228766
C	-2.666991	1.751884	0.117919
C	-4.504139	0.179688	0.225892
C	-4.052837	1.480513	0.170639
C	-2.151613	3.103507	0.066720
C	0.072931	2.138816	0.058197
C	-0.727424	3.238035	0.040028
C	1.520406	2.130945	0.090866
C	2.248567	3.268553	0.366001
C	2.218308	0.836638	-0.208861
C	3.637115	3.230437	0.458198
C	3.685695	0.851839	0.023849
C	4.339353	1.998236	0.307568
H	-0.027719	-1.077523	-1.484365
H	-0.219414	-4.075747	-1.034657
H	-1.795945	-2.737100	0.353736
H	2.031807	-2.181662	-0.266763
H	0.019802	-3.666762	1.493051
H	2.135095	-1.569209	2.147904
H	2.266723	-3.301000	2.522708
H	-1.218012	-2.331630	-3.041439
H	1.576563	-4.006142	-2.472779
H	1.627411	-4.941217	0.182719
H	-5.565175	-0.027230	0.264275
H	0.234043	-1.601909	3.494196
H	-0.303584	4.229148	0.006628
H	-4.982813	-2.172203	0.331923
H	-4.424964	3.330367	0.122139
H	1.749138	4.208827	0.551175
H	1.753394	0.016557	0.342152
H	4.193698	4.130714	0.680051
H	4.222803	-0.080254	-0.087952
H	6.021745	2.853586	0.698183
O	1.930787	0.428617	-1.582379
O	2.485097	1.386376	-2.472157
H	1.739401	1.977174	-2.650314

ENERGIES
SCF ENERGY: -1715.80849842
SUM OF ELECTRONIC AND ZERO-POINT ENERGIES: -1715.393441
SUM OF ELECTRONIC AND THERMAL ENERGIES: -1715.362723
SUM OF ELECTRONIC AND THERMAL ENTHALPIES: -1715.361779
SUM OF ELECTRONIC AND THERMAL FREE ENERGIES: -1715.454508

==> VITEXIN/WATER/RAF/C7 <==
54
XYZ-COORDINATES + ENERGIES

O	-0.139092	-1.587567	0.871546
O	-0.923354	-1.503485	-2.675465
O	1.510902	-2.970751	-2.630014
O	2.021012	-4.383503	-0.148446
O	0.183241	1.060744	-0.095676
O	0.353255	-2.721731	3.404421
O	-3.674338	-1.168154	-1.196463
O	-4.072616	3.302417	-0.055527
O	-1.826346	4.570986	0.129342
O	6.393611	1.338102	0.196494
C	-0.077459	-1.594633	-1.537824
C	0.644667	-2.927865	-1.501567
C	-0.961675	-1.490800	-0.291867
C	1.419350	-3.093827	-0.203196
C	0.491660	-2.867642	0.987715
C	-1.753025	-0.216333	-0.215797
C	1.242772	-2.852874	2.298304
C	-1.178169	1.028411	-0.109585
C	-3.248498	-0.330958	-0.153817
C	-1.915171	2.217785	-0.035210
C	-3.991484	0.960970	-0.148888
C	-3.360483	2.144527	-0.077261
C	-1.207383	3.483912	0.066078
C	0.861353	2.218249	0.014705
C	0.218569	3.410780	0.102448

C	2.307047	2.010351	0.044467
C	3.191918	3.077012	-0.142681
C	2.823693	0.727844	0.268034
C	4.558566	2.871734	-0.097308
C	4.188509	0.516238	0.318163
C	5.056104	1.590785	0.137780
H	0.657780	-0.788705	-1.542037
H	-0.098831	-3.730673	-1.571359
H	-1.661003	-2.329980	-0.304652
H	2.237854	-2.372799	-0.174099
H	-0.280071	-3.646911	1.003311
H	1.964162	-2.031496	2.288006
H	1.773258	-3.792297	2.426969
H	-0.372841	-1.604749	-3.459622
H	1.918563	-3.842955	-2.661443
H	1.321227	-5.047756	-0.172972
H	-5.071371	0.916307	-0.194654
H	-0.127911	-1.894788	3.296996
H	0.775338	4.327352	0.215805
H	-4.638608	-1.140983	-1.238564
H	-3.424255	4.041521	0.015782
H	2.820104	4.072487	-0.339987
H	2.151966	-0.104408	0.418996
H	5.245807	3.694114	-0.247882
H	4.592937	-0.470210	0.499079
H	6.892952	2.156115	0.085142
O	-3.435761	-1.023108	1.112247
O	-4.818046	-1.304819	1.272286
H	-5.121969	-0.584222	1.843056

ENERGIES
SCF ENERGY: -1715.80042997
SUM OF ELECTRONIC AND ZERO-POINT ENERGIES: -1715.385598
SUM OF ELECTRONIC AND THERMAL ENERGIES: -1715.354789
SUM OF ELECTRONIC AND THERMAL ENTHALPIES: -1715.353845
SUM OF ELECTRONIC AND THERMAL FREE ENERGIES: -1715.447651

==> VITEXIN/WATER/RAF/C8 <==
54
XYZ-COORDINATES + ENERGIES

O	0.104957	-1.651879	0.658673
O	-2.504714	-1.254552	-1.827593
O	-0.444220	-2.523698	-3.290944
O	1.344601	-4.167516	-1.705056
O	0.127284	1.051035	0.341403
O	1.841270	-2.922987	2.388402
O	-3.910518	-1.315795	1.074879
O	-4.086979	3.199275	-0.416401
O	-1.806505	4.390639	-0.846989
O	6.355965	1.157568	0.232955
C	-1.177422	-1.397601	-1.342951
C	-0.549518	-2.672972	-1.879597
C	-1.217299	-1.478629	0.181438
C	0.808089	-2.931288	-1.246339
C	0.672220	-2.905025	0.271320
C	-1.818081	-0.211727	0.837015
C	2.007661	-2.992302	0.973964
C	-1.223399	1.063661	0.341427
C	-3.310982	-0.154478	0.712611
C	-1.935875	2.169910	-0.056624
C	-4.011952	0.953922	0.348877
C	-3.352410	2.126356	-0.044980
C	-1.202498	3.355579	-0.488763
C	0.837676	2.118223	-0.064392
C	0.222750	3.260665	-0.465982
C	2.280406	1.881763	-0.000004
C	3.168517	2.680404	-0.725684
C	2.787282	0.840333	0.786613
C	4.531945	2.453882	-0.660981
C	4.149132	0.610829	0.858248
C	5.021014	1.419905	0.134425
H	-0.564766	-0.544802	-1.650460
H	-1.213007	-3.512857	-1.642435
H	-1.825906	-2.335174	0.476158
H	1.508754	-2.155668	-1.559258
H	0.013710	-3.716775	0.603455
H	2.651330	-2.180742	0.625469
H	2.481427	-3.944677	0.752295
H	-2.464075	-1.244861	-2.790445
H	-0.112724	-3.350995	-3.656616
H	0.738025	-4.874507	-1.453402
H	-5.094317	0.924546	0.334421
H	1.351997	-2.114847	2.579888
H	0.801898	4.123482	-0.755999
H	-4.870404	-1.233398	0.999971
H	-3.448220	3.907851	-0.663900
H	2.800382	3.472123	-1.362758
H	2.114607	0.213923	1.355148
H	5.221000	3.065139	-1.229294
H	4.547368	-0.187199	1.469725
H	6.855854	1.778503	-0.310170
O	-1.463468	-0.205665	2.272687
O	-1.682224	-1.479296	2.862664
H	-2.620070	-1.454574	3.102917

ENERGIES
SCF ENERGY: -1715.80464755
SUM OF ELECTRONIC AND ZERO-POINT ENERGIES: -1715.389145
SUM OF ELECTRONIC AND THERMAL ENERGIES: -1715.358698
SUM OF ELECTRONIC AND THERMAL ENTHALPIES: -1715.357754
SUM OF ELECTRONIC AND THERMAL FREE ENERGIES: -1715.450917

==> VITEXIN/WATER/RAF/C8a <==
54
XYZ-COORDINATES + ENERGIES

O	0.391238	-1.612163	1.084074
O	-1.123862	-2.365702	-2.131659
O	1.557910	-3.185384	-2.534890
O	2.987436	-3.922284	-0.120497
O	-0.264755	0.933159	0.141497
O	1.722464	-2.081876	3.511850
O	-3.212682	-2.558372	1.117203
O	-4.843675	1.877798	0.842017
O	-3.181001	3.751819	0.099819
O	5.606224	2.974558	-0.172681
C	-0.058425	-2.043512	-1.238864
C	0.986869	-3.139625	-1.232079
C	-0.657260	-1.928669	0.162597
C	2.041004	-2.859894	-0.172057
C	1.369200	-2.651374	1.182682
C	-1.753469	-0.908228	0.285037
C	2.352786	-2.204723	2.239314
C	-1.556434	0.490830	-0.215443
C	-2.990385	-1.263286	0.757253
C	-2.603428	1.472592	0.167488
C	-4.041504	-0.332633	0.888545
C	-3.823519	1.040796	0.610857
C	-2.292204	2.872343	0.001595
C	0.045432	2.241877	-0.067049
C	-0.905457	3.195678	-0.208838
C	1.492138	2.457720	-0.077229
C	2.035529	3.733943	0.105056
C	2.357516	1.373270	-0.271015
C	3.404494	3.927499	0.077488
C	3.727339	1.559318	-0.301388
C	4.250042	2.838351	-0.131069
H	0.411236	-1.103259	-1.529958
H	0.500065	-4.094695	-1.003722
H	-1.070520	-2.903731	0.425865
H	2.599524	-1.962755	-0.445465
H	0.876455	-3.581164	1.493001
H	2.792767	-1.250625	1.936425
H	3.141321	-2.944761	2.344689
H	-0.741794	-2.542929	-2.999787
H	2.166417	-3.931270	-2.570455
H	2.526839	-4.729368	0.140203
H	-5.009803	-0.648620	1.251092
H	1.012636	-1.437446	3.422960
H	-0.633802	4.224295	-0.386805
H	-4.112681	-2.664836	1.447917
H	-4.526390	2.781864	0.603396
H	1.392109	4.582985	0.287242
H	1.954534	0.380374	-0.409743
H	3.826818	4.913347	0.223556
H	4.399417	0.727055	-0.459422
H	5.854332	3.898697	-0.050939

O	-1.430600	0.478499	-1.683112
O	-2.617556	-0.036205	-2.246870
H	-2.370184	-0.977579	-2.351344

ENERGIES

SCF ENERGY: -1715.80430341
SUM OF ELECTRONIC AND ZERO-POINT ENERGIES: -1715.388201
SUM OF ELECTRONIC AND THERMAL ENERGIES: -1715.358397
SUM OF ELECTRONIC AND THERMAL ENTHALPIES: -1715.357453
SUM OF ELECTRONIC AND THERMAL FREE ENERGIES: -1715.448051
